# Mechanistic insights into chemical exchange during the signal amplification by reversible exchange sensitization of pyruvate

**DOI:** 10.1038/s41467-026-74502-1

**Published:** 2026-06-25

**Authors:** Charbel D. Assaf, Vladimir V. Zhivonitko, Amaia Vicario, Alexander A. Auer, Simon B. Duckett, Jan-Bernd Hövener, Andrey N. Pravdivtsev

**Affiliations:** 1 Section Biomedical Imaging (SBMI), Molecular Imaging North Competence Center (MOINCC), Department of Radiology and Neuroradiology, University Hospital Schleswig-Holstein, Kiel University, Kiel, Germany; 2 NMR Research Unit, Faculty of Science, University of Oulu, Oulu, Finland; 3 University of Bath, Faculty of Science, Claverton Down, Bath, UK; 4 Max-Planck-Institut für Kohlenforschung, Mülheim an der Ruhr, Germany; 5 Centre for Hyperpolarization in Magnetic Resonance (CHyM), Department of Chemistry, University of York, Heslington, UK

**Keywords:** Solution-state NMR, Chemical physics, Chemical bonding

## Abstract

Signal amplification by reversible exchange (SABRE) is a nuclear spin hyperpolarization technique in which the transient interaction of parahydrogen (pH_2_) and a target substrate with an iridium complex leads to polarization of the substrate. SABRE enables direct hyperpolarization of the substrate without chemical modification, enabling rapid polarization buildup within seconds under mild conditions. Here, we use a parahydrogen-enhanced, spin-selective nuclear magnetic resonance method to investigate pyruvate binding, which is combined with exchange-model fitting and density functional theory calculations. Our study reveals several key findings that reshape the current understanding of SABRE. First, we observe that intramolecular hydrogen exchange of the hydrides occurs faster than pyruvate or H_2_ loss. Second, we discover a distinct stable [Ir(H)_2_(IMes)(κ^1^-pyr)(DMSO)_2_] complex. Finally, the results suggest a potential role of counterions (here Na^+^) in Ir-pyruvate binding. Insights into complex kinetics and distributions as a function of temperature, [DMSO], [pyruvate], and hydrogen pressure are presented. The methods demonstrated here, exemplified by SABRE, provide a framework that is expected to guide future research in the field.

## Abstract (web summary)

The study investigates the exchange kinetics in the polarization transfer from parahydrogen to pyruvate via Ir-pyruvate complexes to pyruvate in signal amplification by reversible exchange (SABRE) hyperpolarization, and finds inconsistencies with previous structural assignments leading to a revised mechanistic understanding of SABRE exchange processes.

## Introduction

For more than a decade, [1-^13^C]pyruvate has been the most prominent tracer for hyperpolarized metabolic magnetic resonance imaging^1–3^. The reason for this is a sufficiently long polarization lifetime, a sufficiently high applicable dose, rapid in vivo metabolic transformation into lactate, alanine, bicarbonate, and CO_2_, and their association with inflammation^3^ and tumor malignancy^4,5^ as a result of the Warburg effect^6^. To achieve the necessary increase in polarization (i.e., MRI signal) of pyruvate and its metabolic products, dissolution dynamic nuclear polarization (dDNP) has emerged as the most successful translational method.^7^ This method can produce up to 60% of ^13^C polarization and deliver >10 ml, >200 millimolar concentrations of pyruvate for in vivo use following a 30–60 min polarization and dissolution process, but it requires costly equipment. However, the hyperpolarization community widely aspires to develop swifter and more cost-effective alternatives for hyperpolarizing biomolecules,^8^ with one prominent approach being based on parahydrogen^9–11^.

Parahydrogen (pH_2_)-based methods offer faster and more cost-efficient polarization. Hydrogenative parahydrogen-induced polarization (PHIP), more specifically, its side-arm hydrogenation variant (PHIP-SAH)^12–14^, has demonstrated great potential as a viable alternative to dDNP, which is underpinned by the high-yield synthesis of essential precursors^12,13,15–18^. Additionally, it has achieved high ^13^C polarization levels of pyruvate around 20%^19^. The non-hydrogenative variant of PHIP, signal amplification by reversible exchange (SABRE)^20^, offers another route to pyruvate hyperpolarization^19,21,22^. In SABRE, a ligand (e.g., pyruvate) and pH_2_ are temporarily associated with an Ir complex, whose spin-spin interactions allow converting the spin alignment of pH_2_ into the polarization of the ligand. SABRE enables direct hyperpolarization without altering the substrate, allowing rapid polarization buildup within seconds under mild conditions. However, current implementations typically achieve lower ^13^C polarization levels than dDNP and require careful control of exchange dynamics to optimize the performance. The strength of the spin-spin interaction and the lifetime of the complex play critical roles in maximizing polarization transfer^23–25^; the latter can be controlled by tuning the temperature^26^ or ligand identity^27^.

In the most common SABRE process, the exchanging ligand is exemplified by a two-electron donor, such as pyridine^28^. For a better understanding of the system, it is desirable to measure ligand loss rates for the dissociative exchange pathway following the SABRE catalysts, such as [Ir(H)_2_(IMes)(pyridine)_3_]Cl^29–31^. Some recent studies in this area have deployed in-field PHIP to improve sensitivity, selective pulses to enhance selectivity and magnetization transfer efficiency, and used long-lived ^15^N signals to measure low exchange rates^26,32–38^. Obtaining these ligand-exchange rates is valuable, as they also provide benchmarking data for the subsequent in silico optimization of hyperpolarization conditions^39,40^. However, only a few reported studies focus on determining the H_2_ exchange rate^32^, as this chemical exchange process typically occurs after substrate loss and is complicated by the role that a dihydrogen-dihydride complex plays in the exchange mechanism^29,32^. In the [Ir(H)_2_(IMes)(pyridine)_3_]Cl-type systems, it is broadly accepted that the exchange of pyridine-type ligands is dissociative (like in S_N_1), while the exchange of H_2_ that happens after pyridine loss is associative (like in S_N_2)^32,41^.

**Fig. 1:**
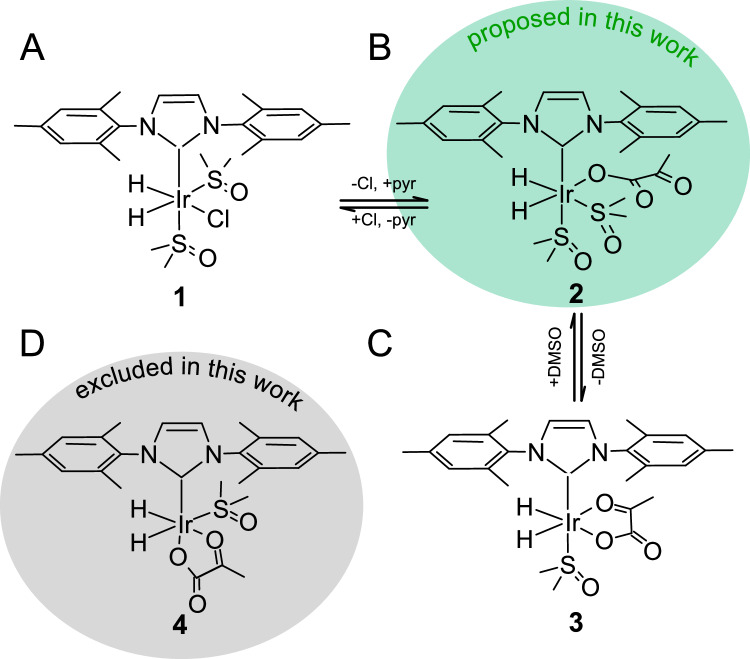
Revised speciation of Ir complexes in pyruvate SABRE. Structures of complexes **1**-**4**. Complexes **1-3** are formed when IrIMes reacts with DMSO, pyruvate, and H_2_ in methanol. **D** Previously, **4** was assumed to be populated to a high level and in exchange with **1** (**A**) and **3** (**C**); however, we show here that it is actually absent and energetically unfavorable compared to its isomer **3**, while **2** (**B**) is present instead. Therefore, **4** should be excluded from consideration for the discussed pyruvate SABRE composition.

Pyruvate (pyr), however, differs significantly from a pyridine-like ligand, as it can act as a mono- or bidentate ligand. For polarizing pyr by SABRE, though, a co-ligand, typically dimethyl sulfoxide (DMSO), is required to be present within the metal’s coordination sphere in addition to the N-heterocyclic carbenes (NHC). The need for a co-ligand and the diverse binding of pyruvate addition significantly complicates the speciation of the resulting metal complex, and several important complexes have been previously proposed to exist in solution.^22^ These included *bis*-DMSO complex [Ir(Cl)(H)_2_(DMSO)_2_(IMes)] (**1**)^42^ (Fig. 1A) and [Ir(H)_2_(κ^1^-κ^2^-pyr)(DMSO)(IMes)], which was initially proposed to exist as two isomers^22^ where pyruvate binds in the equatorial plane (**3**) (Fig. 1C) or bridges equatorial-axial sites (**4**) (Fig. 1D). Previous density functional theory (DFT) predictions^43^ indicated that isomer **4** has a Gibbs free energy 7.6 kJ/mol higher than isomer **3**. Such a significant energy difference implies much lower equilibrium concentrations of **4** than of **3**.

Herein, we show that our experimental results clearly contradict the previous assumptions, revealing significant shortcomings in the existing mechanistic model. In particular, we found that the ^1^H and ^13^C NMR signals, previously associated with complex **4** in the literature^22,27^, can grow to levels exceeding those of **3** as the DMSO concentration increases or as the temperature decreases, highlighting the significant problems in the existing mechanistic model.

Given this contradiction and the importance of hyperpolarizing pyruvate with a cost-efficient method like SABRE, we comprehensively reexamine the mechanistic transformations of the pyruvate SABRE system. Importantly, by collecting 2D NMR and chemical-exchange data, we show that the previously proposed complex **4** (Fig. 1) is not observed and should be excluded from the list of key intermediates under normal conditions. Instead, the intermediate [Ir(H)_2_(IMes)(κ^1^-pyr)(DMSO)_2_] (**2**), forms in the system. Consequently, the corresponding ^1^H and ^13^C NMR resonances previously associated with **4**, in fact, belong to complex **2**. Additionally, we observe a pronounced intramolecular hydride ligand exchange in complexes **1**-**3**, which was previously unreported but can significantly limit the observable ^13^C hyperpolarization levels. Furthermore, we present a Density Functional Theory (DFT) computational analysis of the proposed mechanism, revealing a potential role for Na^+^ interactions in influencing relative stabilities and offering an explanation for the previous misassignment.

**Fig. 2:**
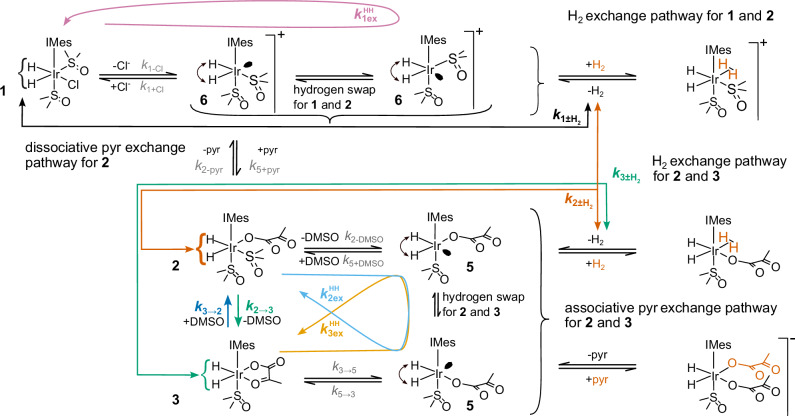
Schematic of pyruvate, DMSO, and H₂ exchange, which explains the observed experimental kinetic dependencies. Critical elements of the exchange: (1) H₂ and pyruvate exchange on a transition between **1** and **2**, where “+pyr” and “-pyr” indicate coordination from or dissociation to the solution phase; (2) intramolecular H-H exchange happens during rearrangement between **2** and **3** and **1** and **2**; (3) transformation from **3** to **2** is [DMSO] dependent. Elementary reaction steps are indicated with grey colored rates and single-barbed arrows. In contrast, transmission rates are indicated with colors and regular arrowheads. For example, transmission ${k}_{3{{{\rm{ex}}}}}^{{{{\rm{HH}}}}}$ involves multiple transformations going from **3** to **5** and back to **3**. Note that for the sake of simplicity ${k}_{2{{{\rm{ex}}}}}^{{{{\rm{HH}}}}}$ is indicated only in the cycle **2**-**5**-**2**, while it could also occur in **2**-**6**-**2**. The transmission *k*_3-pyr_ is not indicated, but it goes from **3** to **5** to the associative pyr exchange. Transmission *k*_2-pyr_ consists of two pathways, one through complex **5** (associative) and the other one through complex **6** (dissociative).

Our experimental approach leverages the fact that the hydride ligand protons (IrHH) in species **1**-**3** do not overlap in the ^1^H NMR spectra, and can therefore be targeted separately by frequency-selective polarization transfer in exchange NMR experiments^44,45^. The resulting data enable the determination of the kinetic parameters for ligand exchange in Fig. 2 with high sensitivity by using pH_2_. Previously, this sensitization method enabled the measurement of *J*-coupling constants between hydride ligands and ^13^C nuclei of bound pyruvate on SABRE complexes^43^.

Overall, we present several crucial results that necessitate reinterpretation of earlier data and suggest that further studies are needed to predict the quantitative outcomes of SABRE if an optimal solution is to be achieved.

## Results and discussion

### Reevaluation of NMR signal assignments and structures in pyruvate SABRE

**Fig. 3:**
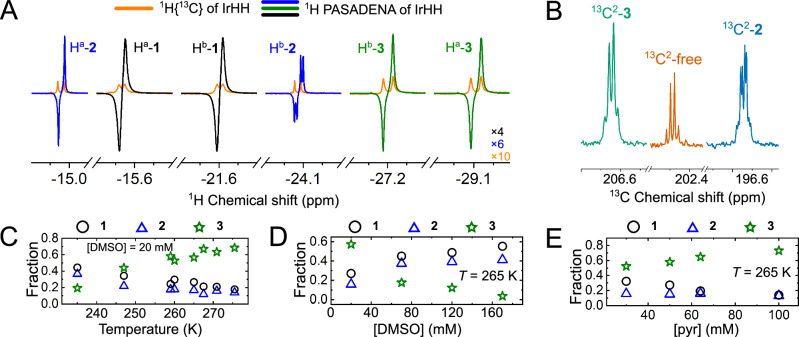
Overview of hydride resonances and concentration dependencies of dominant Ir complexes present in an IrIMes, DMSO, pyruvate, and H_2_ in methanol reaction mixture. **A** ^1^H{^13^C} NMR spectrum of thermally polarized (vermilion: multiplied by 10, ×10) and ^1^H NMR spectrum of PASADENA-polarized IrHH hydrides of **1**-**3** (blue: ×6, black: ×4, and green;  thermal spectrum was acquired with 100 scans with a receiver gain (RG) of 101, while the hyperpolarized spectrum was recorded in a single scan with an RG of 28.5). **B** Representative ^13^C spectra of free [2-^13^C]pyruvate in methanol (vermillion), coordinated to **2** (blue) and **3** (green), obtained at 265 K using the SEPP-SPINEPT-SABRE sequence. **C**–**E** Relative concentrations of hydride protons of **3** (green stars), **2** (blue triangles), and **1** (black circles) as a function of temperature (**C**, [DMSO] = 20 mM), [DMSO] (**D**, T = 265 K), or [pyr] (**E**, T = 265 K, [DMSO] = 20 mM) estimated from corresponding ^1^H NMR spectra. Note that [**3**] and [**2**] were affected differently by [DMSO], indicating different numbers of DMSO ligands. Experimental parameters: *B*_0_ = 9.4 T, 40 mM sodium pyruvate, and 4 mM [Ir(IMes)(COD)(Cl)] precatalyst dissolved in methanol-*d*_3_. Concentrations were measured using ¹H NMR at 9.4 T, with 2 scans for averaging; the tube was constantly maintained under 6.9 bar (100 psi) H_2_ pressure ([H_2_] = 23.73 mM)^52^. Data are provided as a Source Data file.

Our experiments indicate that a revised assignment of SABRE complexes is necessary. In particular, we believe that ^1^H NMR signals of hydride ligands in complex **2** at δ −14.98 and −24.09 ppm (Fig. 3A) were incorrectly ascribed to complex **4** in the literature^22,27,43,46–49^.

An important piece of evidence comes from NOE 2D NMR. We investigated a sample containing IrCl(COD)(IMes), DMSO-*h*_6,_ and [2-^13^C]pyruvate in methanol-*d*_4_ at 253 K after exposure to H_2_. ^1^H-^1^H NOE revealed cross peaks between hydride protons of **2** (δ −14.98 and −24.09 ppm) and three methyl groups of DMSO (^1H^δ 3.37, 2.98, and 2.92 ppm, ESI, Fig. S19). Due to the low hydride-ligand symmetry, a single DMSO ligand should produce two connections to the hydrides, whereas our observation of three peaks was indicative of two DMSO ligands.

To confirm this finding, we carried out two alternative experiments using [U-^13^C]DMSO and nonlabelled pyruvate: HMQC (heteronuclear multiple-quantum correlation)^50^ ^1^H-^13^C correlation 2D NMR (ESI, Fig. S20) and ^1^H-^13^C SEPP-SPINEPT (frequency-selective excitation of parahydrogen-derived PASADENA polarization followed by frequency-selectively pulsed insensitive nuclei enhanced by polarization transfer) that transfers polarization from pH_2_ to neighboring carbons^43^ (ESI, Fig. S21). Here, PASADENA refers to pH_2_ and synthesis allow dramatic enhancement of nuclear alignment^51^ experiment, where the hydrogenation takes place at high field (Fig. 3A).

Applying SEPP-SPINEPT to the hydrides of **2** yielded four hyperpolarized carbons of this isotopomer of DMSO (ESI, Fig. S21). HMQC, on the other hand, provided resonances for the neighboring methyl protons (^1H^δ-^13C^δ pairs were 3.37–48.27, 2.98–58.98, 2.92–44.19, and 3.14–50.20 ppm, ESI, Table S17). These findings confirmed the presence of two DMSO ligands and let us assign four ^13^C and ^1^H resonance pairs, establishing that the correct identity of the complex is [Ir(H)_2_(IMes)(κ^1^-pyr)(DMSO)_2_] (**2**) rather than the previously proposed [Ir(H)_2_(IMes)(κ^1^-κ^2^-pyr)(DMSO)], (**4**)^22^.

### Proposed chemical exchange model and key features of the pyruvate SABRE system

To rationalize our experimental findings, we propose the following general scheme of pyruvate SABRE, providing the reader with a basic picture of the involved processes.

As noted in the introduction, a relatively well-studied SABRE mechanism for pyridyl-type systems includes dissociative loss of pyridine and associative H_2_ exchange. Hence, by analogy, dissociative loss of DMSO and chloride in **1** and **2** (Fig. 1) is likely. However, pyruvate dissociation will be more complicated, as the reversible formation of a κ^1^-pyr intermediate from the κ^1^-κ^2^-pyr counterpart would be expected, for instance, during the interconversion of **3** and **2**. This intermediate will correspond to 16-electron [Ir(H)_2_(IMes)(κ^1^-pyr)(DMSO)] **(5)**, and likely have an octahedral geometry with inequivalent hydride ligands, where one is *trans* to a vacant site. Any H_2_ exchange is then expected to be associative, via the analogous dihydrogen-dihydride intermediate (Fig. 2).

Figure 2 shows such a hypothesized reaction pathway that allows the equilibration of **1**, **2**, and **3** through a series of dissociative reaction steps involving loss of Cl^-^ (*k*_1-Cl_), DMSO (*k*_2-DMSO_), and pyr^-^ (*k*_2-pyr_), or pyr transition from bidentate to monodentate binding (${k}_{3\to 2}$) and back (${k}_{2\to 3}$). These steps involve the 16-electron intermediates **5** (*k*_2-DMSO_, ${k}_{3\to 5}$) and [Ir(H)_2_(IMes)(DMSO)_2_]^+^ (**6,** *k*_2-pyr_ and *k*_1-Cl_). These intermediates are expected to bind DMSO, methanol, Cl^-^, pyruvate, and water. Unless complexes **1**-**3** are formed, the resulting species are unobservable in NMR because they are energetically very unfavorable. The associative bimolecular H_2_ loss occurs via binding of H_2_ to **5** and **6** intermediates (Fig. 2). We propose that these two intermediates also enable the hydride ligands to interchange positions. The corresponding, now first-order, rearrangement steps are indicated as “hydrogen swap” in Fig. 2. The observed hydrogen dissociation transmission rate for **1** is displayed as *k*_1-H2_ and involves several steps, while only the initial reagent and final product are observable.

When viewing this figure, note that elementary reaction steps (grey rates with single-barbed arrows) and transmission rates (not grey rates with regular arrowheads) are shown. Care should be taken since only overall transmission rates between reagents and products are observed, not each intermediate step.

As was noted above, based on the experimental and theoretical evidence supported by DFT calculations (ESI, Fig. S24-29), we have found that complex **4** (Fig. 1) is not observable and does not play a significant role in pyruvate SABRE. We rule it out from the list of key intermediates of this system (Fig. 2). Instead, we prove the existence of complex **2** as one of the key species for pyruvate SABRE, along with complexes **1** and **3**.

In the sections below, we evaluate the validity of the model by performing a series of exchange spectroscopy (EXSY) NMR measurements (data and experimental schemes are in Figs. 3, 5, and 6). EXSY allowed us to monitor the chemical exchange of free pyruvate, DMSO, and H_2_ with their counterparts bound to the iridium complexes. We repeated experiments with varying concentrations of [H_2_], [pyr], and [DMSO] to quantitatively probe the exchange dynamics. Then we discuss how concentrations affect the rates of chemical transformations in the context of the proposed reaction mechanisms here or elsewhere. These observations and the resulting hypothesis of pyruvate SABRE kinetics (Fig. 2) are supported by a computational study detailed below.

### Evaluation of thermodynamic properties of the pyruvate SABRE system

Temperature strongly influences the kinetic parameters and the equilibrium populations of the complexes, affecting the hyperpolarization process^26^. Moreover, it provides insights into the energetics of the intersystem conversion, which can then be compared with DFT simulations to verify the SABRE mechanism. Therefore, we measured the fractions of complexes **1**-**3** as a function of temperature by using ^1^H NMR signals of hydride ligands (Fig. 3C). These temperature dependencies were probed multiple times with freshly prepared samples and at two different research centers to ensure reproducibility. Notably, the fraction of **3** increased with increasing temperature, while the amounts of **2** and **1** decreased. This observation implies that **3** is thermodynamically less favorable than **2** and **1**, being higher in relative energy. Considering equilibrium reaction conditions for these complexes, the Gibbs free energies of **1** ← **3** and **2** ← **3** transformations were estimated using the concentration ratios $\frac{\left[2\right]}{\left[3\right]}={\left[{{{\rm{DMSO}}}}\right]{\rm{exp}}}{\left(\frac{-\varDelta {G}_{2\leftarrow 3}^{0}}{{RT}}\right)}$ and $\frac{\left[1\right]}{\left[3\right]}=\left[{{{\rm{DMSO}}}}\right]$, as detailed in (ESI, Eqs. S36 and S39). These analyses yielded $\varDelta {G}_{2\leftarrow 3}^{0}$ = $\varDelta {G}_{2}^{0}-\varDelta {G}_{3}^{0}$ = -(5.3 ± 0.14) kJ/mol and $\varDelta {G}_{1\leftarrow 3}^{0}$ = -(11.85 ± 0.51) kJ/mol at *T* = 270.9 K (ESI, Section 6).

### DMSO concentration dependency

The relative proportions of the two-DMSO complexes, **2** and **1**, increased with increasing [DMSO], while the one-DMSO complex, **3**, decreased (Fig. 3D). This behavior cannot be rationalized with the previous assignment of complexes **1**, **3**, and **4**, because, in this case, only [**1**] would increase, while [**3**] and [**4**] would relatively decrease. Thus, we further confirmed the revised identity of **2**, whose hydride ligand chemical shift signals were found not to change with increasing [DMSO] (ESI, Fig. S19). A related DMSO-dependent change in observed ^13^C hyperpolarized signal amplitudes was independently reported by Mamone et al.^46^, although without analysis of equilibrium populations or reassignment of complex identities. Given the modified structures and associated chemical exchange diagrams (Fig. 2), the dependence on [DMSO] becomes evident (ESI, Section 6).

### Pyruvate concentration dependency

A further study was then undertaken where the [pyr] was increased (Fig. 3E). The proportions of the three iridium complexes were found to depend on [pyr], with their total concentration decreasing to approximately 40% of the total iridium concentration with increasing [pyr]. This finding is in line with previous observations where the polarization yield was negatively correlated with [pyr]^49,53^.

Surprisingly, the concentration of **3** also increases relative to **2** as a function of [pyr], which was not expected based on the presented chemical model (Fig. 2). This is because, according to their chemical structures, both **2** and **3** contain one pyr ligand; hence, changes of [pyr] should not favor one of them. The observed trend, therefore, indicates that the system behaves more intricately than the simple model predicts.

### Computational study

**Fig. 4:**
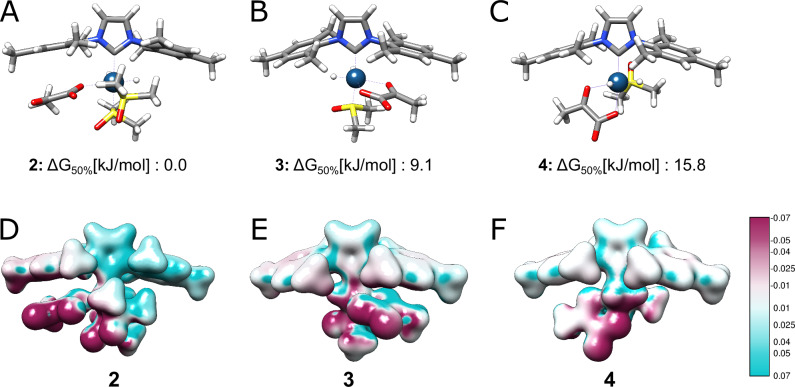
Results of DFT calculations. Optimized structures of complex 2 (**A**, **D**) and 3 (**B**, **E**) and 4 (**C**, **F**), and corresponding electrostatic potential surfaces (**C**, **D**) calculated at the B3LYP-D4/def2-tzvp CPCM(methanol) level of theory. ΔG_50%_ refers to relative Gibbs free energies for which the entropy contribution has been scaled by 0.5 to compensate for deficiencies in the description of the translational entropy correction upon DMSO dissociation.

Electronic structure calculations at the B3LYP-D4/def2-TZVP CPCM (Methanol) level of theory^54–62^ using the ORCA 6 program package^63,64^ yielded relative Gibbs free energy differences (ΔG_50%_,^65–67^ see ESI, Section 18 for details, Table S18) of +9.1 and +15.8 kJ/mol for **3** and **4** with respect to **2** (Fig. 4). Within the accuracy of the level of theory, this is consistent with the experimentally evaluated thermodynamic parameters presented above, which indicated that **2** is energetically favorable over **3** by about 5 kJ/mol.

Upon studying the relative energies and electronic structures of the complexes, we found that the electrostatic potentials of **2,** **3**, and **4** (Fig. 4) are particularly indicative of potentially strong counterion interactions. We found that their relative stabilities are strongly influenced by the explicit addition of species such as Na^+^. Note that implicit solvation models are known to exhibit larger errors in the case of strong specific interactions with solvent molecules or counterions. We also checked the impact of Na^+^ because SABRE presumably works with sodium pyruvate, as originally proposed, rather than with pyruvic acid, for which SABRE has not yet been shown to work, providing another experimental indication of the possible role Na^+^-pyr pairing plays in complex stabilization. Here, we found that the complex-counterion interaction with Na^+^ is strongest for **2** (ΔE = 144.1 kJ/mol), followed by **3** (ΔE = 116.8 kJ/mol) and **4** (ΔE = 113.0 kJ/mol), in agreement with the electrostatic potential assessment. For a series of simple adduct models (complex with Na^+^, methanol, or DMSO, ESI, Fig. S26(1),(2)), this can lead to changes in the relative free energies of more than 10 kJ/mol. While these preliminary results should not be regarded as quantitative, they hint at the great potential of this influence, and further detailed experimental and computational studies of the ions’ effects are underway in our group.

### Hyperpolarization-enhanced observation of proton exchange

To validate the revised model generated based on equilibrium data, we investigated the kinetics of chemical exchange, focusing on H₂ and pyruvate. These measurements allowed us to test the proposed mechanism and quantify the rates of exchange under varying experimental conditions.

**Fig. 5:**
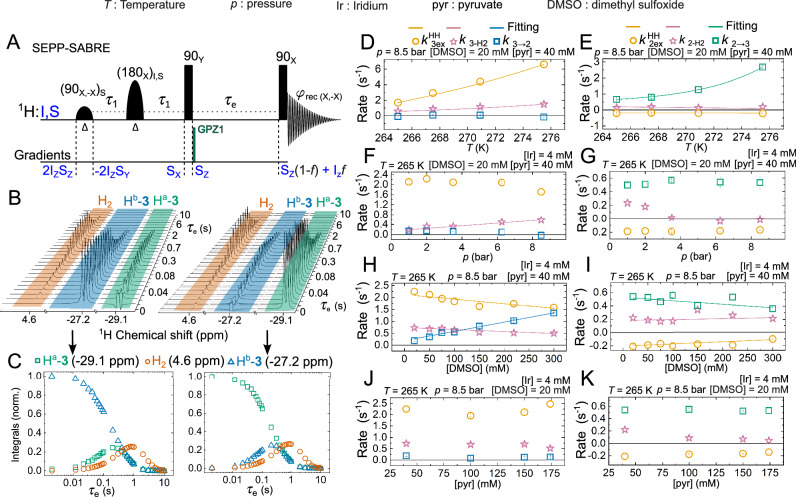
Observation of hydride chemical exchange. Using a SEPP-SABRE pulse sequence (**A**) enabled us to enhance the signals of hydride protons selectively (**B**, left: H^b^-**3** and right: H^a^-**3**) to observe the intra- and intermolecular H_2_ exchange (**C**) and extract exchange rates *k*^HH^_3ex_, *k*^HH^_2ex_ (orange, circles), *k*_3→2_ (blue, squares), *k*_3-H2_, *k*_2-H2_ (purple stars), *k*_2→3_ (green, squares), of **3** and **2** as a function of temperature (**D**, **E**), pH_2_ pressure (**F**, **G**), [DMSO] (**H**, **I**), and [pyr] (**J**, **K**) using (**3** ↔ **2**) ↔ H_2_ model as described in ESI. Note the increase of *k*_3→2_ as a function of [DMSO] and large *k*^HH^_3ex_ compared to other rates. Round shapes indicate frequency-selective Gaussian pulses with different amplitudes and fixed duration Δ = 10 ms . Indices indicate the excited nuclear spins. Rectangular pulses indicate hard pulses. Several delays were applied: ${\tau }_{1}=1/4{J}_{{IS}}$ = 10 ms to achieve the refocusing of the spins after the selective pulse; ${\tau }_{e}$ stands for the time of longitudinal magnetization evolution, which was varied to measure the exchange. During ${\tau }_{e}$, a fraction *f* of longitudinal magnetization of the spins S chemically (or through cross-relaxation) exchanges with spin-*I*. We repeated the experiment twice, changing the phase of the first selective RF pulse, *φ*_1_ = [X, −X], and the phase of the receiver φ_rec_ = [X, −X], which suppressed the background signal of spins not affected directly by the first selective pulse or indirectly via chemical exchange with this spin. A gradient pulse was applied for 2 ms with SMSQ10.100 shape at 31% amplitude. Error bars represent the standard errors of the fitted parameters obtained from nonlinear least-squares fitting using MATLAB (nlinfit), calculated from the residual covariance matrix of the fit. Solid lines represent linear or exponential fits to the data, as indicated in each panel. The data shown correspond to single representative experiments. Additional independently prepared samples were measured under the same conditions and yielded reproducible trends. Source data are provided as a Source Data file.

To study reaction kinetics, pH_2_-derived hyperpolarization was used to sensitize the associated measurements according to the PASADENA protocol. As PASADENA yields antiphase signals after a single hard pulse^51^, we used the frequency-selective excitation of PASADENA (SEPP)^44,45^ to create longitudinal polarization for the selectively excited hydride proton (e.g., ${\widehat{I}}_{z}^{{H}^{a}-\left(1\right)}$ for H^a^ of **1**) to monitor its subsequent chemical exchange. Utilization of pH_2_ and SEPP allowed us to enhance, for example, the H^a^ or H^b^ proton signals of **1** at 265 K by ~8060-fold at 9.4 T ([DMSO] = 75 mM, [Ir] = 4 mM, [pyr] = 40 mM), which dramatically accelerated data collection and proved critical for investigating complexes with low stability, e.g., **3** (the samples can degrade after preparation^68^). To improve data quality, after the SEPP pulse element (90°-${\tau }_{2}$-180°-${\tau }_{2}$, Fig. 5A), the magnetization associated with the selectively excited proton was rotated into the z-axis, and a pulsed field gradient was applied to suppress nonzero-order quantum coherences arising during the exchange encoding time ${\tau }_{e}$ (Fig. 5B, C). We refer to this experiment as SEPP-SABRE (Fig. 5A).

A simple two-step phase cycle and dephasing gradients were used to improve background signal suppression, with a 180° phase shift of the first pulse and the receiver to suppress signals unaffected by the frequency-selective first pulse (Fig. S2B). No additional filters^69^ were used to enable the observation of the early exchange, because remaining zero-quantum terms do not contribute to the total integral^37^ (Fig. 5B). Relaxation was taken into account in the data fitting process, as the *T*_1_’s of the interchanging sites may significantly differ.

Using this technique, the hydride site interchange rate, isomer interconversion rate, and any ligand loss rates were assessed as a function of the concentrations of H_2_, DMSO, chloride, and pyruvate (Fig. 5D–K).

It is worth noting some limitations of this approach. As with all EXSY-type experiments, it is not always possible to distinguish between nuclear Overhauser effect (NOE) cross-relaxation and actual chemical exchange; however, in some cases, NOE can lead to a transfer of polarization with the opposite sign, which is equivalent to a negative flux rate constant. Despite this limitation, the exchange rates obtained from the data fitting are of the correct order of magnitude, since chemical exchange processes are faster than NOE cross-relaxation in most of the cases considered here. Therefore, we did not impose any constraints on the flux rates during the fitting process. We observed a consistently negative flux-rate constant (indicative of NOE-mediated polarization transfer) during measurements of slow intramolecular magnetization exchange between two hydrides in complex **2** (Fig. 5E, G, I, K). In all other cases, the flux rates were positive in sign despite an expected but apparently weak negative cross-relaxation contribution.

### Intercomplex exchange

When the two hydride protons of **3** were selectively excited at 265 K in different experiments, at DMSO concentrations of less than 20 mM under 8.5 bar of H_2_, no exchange with **2** was observed. However, when DMSO concentrations were greater than 20 mM, the conversion of **3** to **2** became visible. Moreover, the conversion from **3** to **2** was linearly [DMSO] dependent. This flux ${k}_{3\to 2}$ is therefore approximately equal to [DMSO]×*k*_2+DMSO_, further confirming the proposed reaction scheme and indicating that we did not reach the saturation of **3** to **2** conversion and [DMSO]×*k*_2+DMSO_ ≪ ${k}_{3\to 5}$ for [DMSO] below 300 mM and T of 265 K (Fig. 5H, I).

### Hydrogen site intramolecular exchange in 3

The additional process of hydride site exchange in **3**, flux ${k}_{3{{{\rm{ex}}}}}^{{{{\rm{HH}}}}}$, was readily evident in all of the ^1^H NMR spectra shown in Fig. 5B, with directly analogous behavior seen regardless of which hydride ligand was selected, alongside the slightly slower transfer of the magnetization into that of free H_2_ (integrals shown in Fig. 5C). Moreover, the observed ${k}_{3{{{\rm{ex}}}}}^{{{{\rm{HH}}}}}$ flux decreased gradually with increasing [DMSO], which is consistent with possible interception in the κ^1^ intermediate **5** during *k*_2+DMSO_ step. Notably, the symmetric conversion observed from an excited hydride (e.g., H^a^) to both H^a^ and H^b^ within the same complex **3** or **2** indicates that the intramolecular hydride exchange is much faster than competing processes, such as DMSO binding ([DMSO]×*k*_5+DMSO_), or pyruvate rearrangement (${k}_{5\to 3}$). Furthermore, the dissociation of H_2_ via **3** occurs much faster than via **2**, and can be accelerated by increasing [H_2_] (Fig. 5F, G) or inhibited by increasing [DMSO] (Fig. 5H, I) through competitive trapping of **5**.

### Hydrogen-observed intramolecular exchange in 2

In contrast to intramolecular exchange in **3**, when the two hydride ligand signals of **2** were excited separately in two separate experiments, no hydride site interchange is evident on the same reaction timescale. Instead, we observed the buildup of negative polarization of the non-excited hydride, indicating a cross-polarization effect within the complex. Accordingly, the fitted conversion rate appears negative (Fig. 5E, G, I, K). This shows that polarization is first transferred to the other hydride (with opposite sign) before dissociation to free H_2_. A similar effect is likely present for complexes **1** and **3**, indicating that the observed exchange rates are underestimating actual exchange rates due to the intramolecular cross-relaxation rate. At the same time, the slow production of H_2_ and slightly faster conversion of **2** into **3** were evident. Under these conditions, *k*_2-DMSO_ controls access to **5** proving ${k}_{5\to 3}$ to be faster than H_2_ dissociation, *k*_2-H2_, and intracomplex exchange ${k}_{2{{{\rm{ex}}}}}^{{{{\rm{HH}}}}}$.

### H_2_ exchange

The effect of [H_2_] on these processes was evaluated by changing pH_2_ pressure over the range of 1 to 8.5 bar. This pressure change, and as a result [H_2_] change, led to a progressive increase in the degree of visible H_2_ loss, flux *k*_3-H2_ from **3** (Fig. 5F), thereby confirming that these exchange processes occur by H_2_ addition to **5** (i.e. also S_N_2 mechanism as with [Ir(H)_2_(IMes)(pyridine)_3_]Cl-type systems), accessed by κ^2^-pyr to κ^1^-pyr rearrangement. The loss of H_2_ from **2**, flux *k*_2-H2_, however, was always much lower than flux *k*_3-H2_ (Fig. 5G). The dissociation of H_2_ from **1**, flux *k*_1-H2_, however, also slightly increased with [H_2_] and was an order of magnitude faster than that of **3** (ESI, Fig. S5(6), Table S5(2)). When the hydride resonances of **2** were examined, the rates of formation of free H_2_ and of conversion to **1** were found to decrease as [H_2_] increased, indicating that suppressing the flux to **1** impacted relayed H_2_ loss via **1**.

The [DMSO]-dependency data at 8.5 bar of H_2_ revealed that the **2** to **3** conversion, flux ${k}_{2\to 3}$, was decelerated while the **3** to **2** conversion, flux ${k}_{3\to 2}$, was accelerated when [DMSO] was increased (Fig. 5H, I). There were minimal changes seen in the behavior of **1** in this case (ESI, Fig. S6). The impact of [pyr] on these changes was also evaluated for concentrations between 40 and 174 mM with 8.5 bar pH_2_. This led to a slight decrease in the rate of H_2_ liberation from **2** and **3** (Fig. 5J, K).

### Hyperpolarization-enhanced tracking of pyruvate exchange

**Fig. 6:**
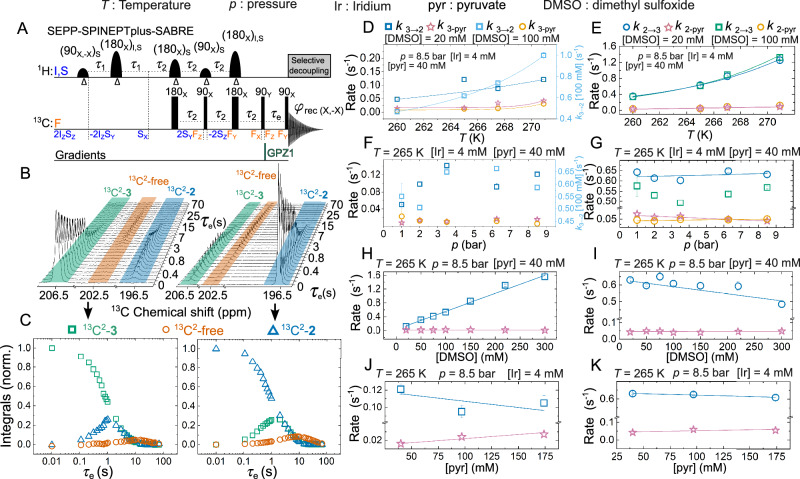
Observation of pyruvate chemical exchange. Scheme of SEPP-SPINEPTplus-SABRE spin order transfer sequence (**A**), corresponding ^13^C NMR spectra at 265 K, where selective ^13^C^2^ of **3** (B, left) and **2** (**B**, right), corresponding integral kinetics of ^13^C^2^-**3** (bluish green, squares), ^13^C^2^-free (vermillion, circles) and ^13^C^2^-**2** (blue, triangles) (**C**), and extracted from such kinetics pyruvate dissociation rates ${k}_{3-{pyr}}$_,_ and ${k}_{2-{pyr}}$, for 20 mM of DMSO (reddish purple, stars), for 100 mM of DMSO (orange, circles) exchange flux between complexes ${k}_{3\to 2}$, (blue, squares) for 20 mM of DMSO and (sky blue, squares) for 100 mM of DMSO and ${k}_{2\to 3}$ (blue, circles) for 20 mM of DMSO and (bluish green, squares) for 100 mM of DMSO as a function of temperature (**D**, **E**), pH_2_ pressure (**F**, **G**), [DMSO] (**H**, **I**), and [pyr] (**J**, **K**) by performing a global fit using a model (^13^C^2^-**3** ↔ ^13^C^2^-**2**) ↔ ^13^C^2^-free described in ESI (S10,11). The round pulses were frequency-selective (a Gaussian shape was used) with different amplitudes, and the exact duration Δ = 10 ms. Additional indices for selective pulses indicate which nuclear spins are excited. Rectangle pulses indicate the hard pulses. Several delays were applied: ${\tau }_{1}=1/4{J}_{{IS}}$ = 10 ms to achieve the refocusing of the spins after the selective pulse, ${\tau }_{2}=1/4{J}_{{SF}}$ = 70 ms in **3**^43^, and 80 ms in **2**^43^, ${\tau }_{e}$ stands for the time of longitudinal magnetization evolution, which was varied to measure the exchange. We repeated the experiment twice, changing the phase of the first selective RF pulse, *φ*_1_ = [X, −X], and the phase of the receiver φ_rec_ = [X, −X], which suppressed the background signal of spins not affected directly by the first selective pulse or via chemical or indirectly via chemical exchange with this spin. A gradient pulse was applied for 2 ms with an SMSQ10.100 shape at 31% amplitude. Error bars represent the standard errors of the fitted parameters obtained from nonlinear least-squares fitting using MATLAB (nlinfit), calculated from the residual covariance matrix of the fit. Solid lines represent linear or exponential fits to the data, as indicated in each panel. The data shown correspond to single representative experiments. Additional independently prepared samples were measured under the same conditions and yielded reproducible trends. Source data are provided as a Source Data file.

To investigate pyruvate exchange, we selectively hyperpolarized the 2-^13^C nucleus of bound pyruvate in **2** or **3** and followed the label over time. To do so, we transferred the polarization first to one of the two hydride protons H^a^ or H^b^ (using SEPP), and then to the 2-^13^C of bound pyruvate (using selective INEPT, SPINEPT, Fig. 6A)^45^. By adding two more 90° pulses and a variable evolution time ${\tau }_{e}$, we measured the exchange of pyruvate using the resulting SEPP-SPINEPTplus-SABRE sequence as a function of [DMSO], *T*, [pyr], and hydrogen pressure for complexes **2** and **3** (Fig. 6). The kinetics (exemplary Fig. 6B, C) were analyzed using a global fit with the model (^13^C^2^-**3** ↔ ^13^C^2^-**2)** ↔ ^13^C^2^-free (ESI), with the primary parameters plotted in Fig. 6: the dissociation flux rate *k*_2-pyr_, and intramolecular exchange flux rates ${k}_{3\to 2}$, and ${k}_{2\to 3}$ were defined in Fig. 2. Previously, a somewhat similar approach was proposed to transfer pH_2_ spin order to ^1^H nuclei of ligands^35^, which we cannot employ due to the too narrow chemical shift dispersion of ^1^H pyruvate signals and rapid relaxation of protons.

### Pyruvate-observed *inter*complex exchange

For the low [DMSO] of 20 mM, the flux ${k}_{3\to 2}$ was 6-8 times lower than that of flux ${k}_{2\to 3}$. At high [DMSO] (hundreds of mM), ${k}_{3\to 2}$ ≈ ${k}_{2\to 3}$ (Fig. 6H, I). Both fluxes proved to be temperature-dependent, as expected. Interestingly, flux ${k}_{3\to 2}$ grew proportionally to [DMSO], while flux ${k}_{2\to 3}$ decayed slowly because high [DMSO] prevented pyruvate from binding back to form **3**. Hence, increased [DMSO] proved to inhibit 2→3 conversion as expected through the [DMSO] dependent *k*_5+DMSO_ step. This aligned with the observations made by SEPP-SABRE applied to hydrides and supported the identity of **2**: ${k}_{2\to 3}$ and ${k}_{3\to 2}$ flux values measured by probing hydride were within error of those observed here by probing pyruvate.

The increased temperature promoted complex reactivity and hence pyruvate unhooking dissociation in **3**, or DMSO loss from **2**, thereby facilitating intracomplex exchange. The concentrations of [H_2_] and [pyr] did not significantly influence the flux of these processes: the slight decline in ${k}_{3\to 2}$ flux as a function of increased [pyr] aligned with the observed stabilization of **3** relative to **1** and **2** with an excess of pyr (Fig. 3E).

### Pyruvate dissociation rate

The observed pyruvate dissociation flux at studied temperatures was always slower than the flux of *inter*complex exchange between **2** and **3** mediated by **5**. For example, at [Ir] = 4 mM, [DMSO] = 20 mM, [pyr] = 40 mM at 265 K, the fluxes ${k}_{2-{pyr}}$ and ${k}_{3-{pyr}}$ were (0.042 ± 0.004) s^−1^, and (0.016 ± 0.001) s^−1^, respectively, whereas the interconversion fluxes were ${k}_{3\to 2}$ = (0.121 ± 0.006) s^−1^ and ${k}_{2\to 3}\,$= (0.630 ± 0.007) s^−1^.

Pyruvate exchange can go via dissociative exchange and complex **6**, or via associative exchange and complex **5**. When it goes through **5**, the complex [Ir(H)_2_(IMes)(κ^1^-pyr)_2_(DMSO)] is generated as a subsequent step; thus, the observed exchange should be [pyr]-dependent. This is in accordance with the observation of the acceleration of ${k}_{3-{pyr}}$ with the increase of [pyr] (ESI, Fig. S17G). Hence, the addition of pyr could accelerate pyr exchange. At the same time, ${k}_{2-{pyr}}$ is less impacted by [pyr] (ESI, Fig. S17H) than ${k}_{3-{pyr}}$, indicating that for **2**, the dissociative exchange has a greater contribution than the associative exchange at 265 K and [pyr] up to 175 mM.

### Rationalization of SABRE: implications of H_2_ and pyruvate exchange

A comprehensive mechanism for the complex transformations during SABRE was developed (Fig. 2), based on both experimental observation and DFT calculations. While complex **3** remains critical to this process, it is notable that the identity of the other critical complex **2** differs from the previously suggested complex **4**^22,27,43,47–49^. Electronic structure calculations indicate a strong influence of Na^+^ on the relative stability of these complexes, which might play an important role in the observed populations of pyruvate SABRE complexes.

Exchange within a complex is detrimental to SABRE polarization yield. While **3** was considered optimally suited for polarization transfer due to the associated *J*-coupling network,^43^ and was assumed to be an energetically favorable state, the loss of the oxo-κ^2^-bonding interaction, which occurs during the intramolecular hydride exchange, disrupts the critical spin-spin coupling network, thereby hindering polarization transfer. Moreover, the rapid exchange of the hydride ligands averages different *J*-coupling constants. In the extreme, the two become equal; the pH_2_ spin order cannot be converted into polarization of the target nucleus (ESI, Section 16, Fig. S22).

The need for stable complex **2**, which can be accessed via complex **3** or **1**, for optimal SABRE performance is therefore questionable. Our results suggest that both **1** and **2** negatively affect polarization transfer. Consequently, stabilizing **3**, while maintaining sufficient H_2_ exchange, appears promising for boosting pyruvate hyperpolarization. This finding suggests that designing a ligand sphere that favors complex **3**, and suppresses complexes **1** and **2**, could maximize the concentration of the active catalyst, thereby improving the target polarization. Alternatively, populating only **2** could also be potentially a remedy.

Similarly, reducing the intramolecular hydrogen exchange would also increase polarization. In fact, reduced intramolecular hydride exchange may be the reason why the highest polarization levels of SABRE were achieved at low temperatures.^19,26^ In addition, higher temperatures are required for pyruvate dissociation, in accordance with the temperature-cycling process used to deliver polarized free material.^26^

These measurements have also revealed that the **2**–**3** interconversion process also facilitates indirect H_2_ loss. This situation arises because H_2_ dissociates from **3** much faster than from **2**, and this indirect pathway contributes to the experimentally observed H_2_ loss when **2** was probed. Furthermore, the fastest observed process involved hydride site exchange in **3**, thereby confirming that the prerequisite ${k}_{5\to 3}$ is the fastest individual step. As stated, this process leads to bond rupture and, therefore, interruption of the spin-spin pathway associated with ^13^C-SABRE transfer. Thus, while rapid H_2_ exchange will be limiting SABRE, intramolecular hydride site exchange must also be considered when evaluating the potential activity of a catalyst. However, the slow process of pyruvate exchange must also be optimized. At −8 °C, there is no significant pyruvate exchange^26,70^, as shown by the fact that the observed flux ${k}_{3-{pyr}}$ is (0.005 ± 0.004) s^−1^ and ${k}_{2-{pyr}}$ = (0.031 ± 0.005) s^−1^.

Our study indicates that the current system is not yet suitable for polarizing at high pyruvate concentrations. Moreover, the refined structure for **2**, when considered alongside previous experimental observations^43^, raises the question of why the four-bond ^4^*J*_CH_ is greater than the three-bond ^3^*J*_CH_. The accuracy of DFT-calculated *J*-coupling constants to hydrides is low, making it difficult to use this metric to explain this trend.^43,47^ Future studies should therefore consider matching DFT-derived reaction paths to reaction flux to optimize outcomes. The potentially significant influence of the cation counterion also suggests that solvation will be a key parameter in improving the SABRE outcome.

## Methods

### Chemicals

Perdeuterated Ir precatalyst [Ir-*d*_22_] = [IrCl(COD)(IMes-*d*_*22*_)] was synthesized according to ref. ^30^ (IMes = 1,3-bis(2,4,6-trimethylphenyl)imidazol-2-ylidene, COD = 1,5-cyclooctadiene), sodium pyruvate-2-^13^C (^13^C-pyr, 490725, Sigma-Aldrich), dimethylsulfoxide-*d*_*6*_ (DMSO, 00905-25, Deutero GmbH), methanol-*d*_4_ (441384, Sigma-Aldrich), were used here. The perdeuterated Ir precatalyst [IrCl(COD)(IMes-*d*_22_)] was purchased from the group of Prof. S. Duckett (commercial source).

### Sample preparation

The samples were prepared by mixing 40 mM of ^13^C-pyr with 4 mM of [Ir-*d*_22_] and 20 mM of DMSO in 0.6 mL of methanol-*d*_*4*_ unless otherwise stated.

### Parahydrogen enrichment

pH_2_-enriched hydrogen gas with a 93% fraction of pH_2_ was prepared by passing high-purity hydrogen over hydrated iron(III) oxide at 25 K using a pH_2_ generator similar to the one in ref. ^71^.

### SABRE experiments

All hyperpolarization experiments were carried out on a Bruker 400 MHz WB Avance NEO spectrometer using a 5 mm BBFO probe. The NMR tube was then attached to a bubbling system (similar to the one used in Ref. ^72^) and placed inside the spectrometer. The solution was bubbled with parahydrogen-enriched H_2_ at various specified pressures, e.g., 8.5 bar. First, the sample was bubbled with pH_2_ for 30 min to activate the catalyst. One experiment consisted of a 5 s relaxation delay followed by 2 s of WALTZ-16 on the ^1^H channel, then 10 s of pH_2_ bubbling through the sample, followed by a 1.5 s delay for the bubbles to dissipate, and finally, the SOT sequence was applied. Two SOT sequences were used: SEPP-SABRE (Fig. 5A) and SEPP-SPINEPTplus-SABRE (Fig. 6A).

### Acquisition of thermally polarized NMR spectra

To acquire reference spectra for signal enhancement estimation of the ¹H NMR spectra at 9.4 T, we prepared a sample containing 4 mM of [Ir-*d*_22_], 40 mM of ¹³C^2^-pyr, and 20 mM of DMSO in 500 μL of methanol-*d*₃. We performed a zgesgp ^1^H NMR experiment that suppresses the water peak 6.58 ppm due to using a D_2_O lock, which shifts the water signal. We used 100 scans with a 90° flip angle, with RG = 101 using TR = 9 s.

For the ^13^C NMR thermally polarized NMR spectra, we used a 40 mM sample of ^13^C-pyr containing 4 mM of [Ir-*d*_22_] and 20 mM of DMSO in 0.6 mL of methanol-*d*_*4*_. We performed 600 acquisitions after a 90° flip angle at 9.4 T using TR = 130 s.

### Data processing and fitting

All NMR spectra were analyzed using the spectral data analyzing software Bruker TopSpin (4.1.4), MestReNova (14.2.2), and Origin (2021). All the kinetics profiles were analyzed using MATLAB scripts (available as supporting materials). All error margins for the fitted values are standard deviations estimated using the MATLAB nonlinear regression “*nlinfit*” function.

### Thermally polarized exchange spectroscopy

Kinetic measurements using thermal polarization were performed on an Avance III Bruker 600 MHz spectrometer equipped with a variable temperature unit. A standard EXSY NMR experiment (selnogpzs.2 Bruker experiment) was used in the measurements. The following mixing times were used in the experiments (s): 0.010, 0.020, 0.050, 0.075, 0.100, 0.150, 0.200, 0.300, 0.400, 0.500, 0.600, 0.800, 1.200, 1.600, 2.400, 3.200. The number of scans was 256. The measured EXSY curves, as well as the curves obtained from the data fitting procedures, are shown in the Fig. S8, Section 7.5, ESI. For the details of the fitting procedure, see Section 1.1, ESI.

### Computational details

All calculations have been carried out at the B3LYP-D4/def2-TZVP CPCM (methanol) level of theory^54,62^. Every geometry was fully optimized using tight convergence criteria and confirmed as a minimum structure via normal mode analysis. Note that while no extensive conformational searches have been carried out using refined search algorithms, several possible conformations of various structures have typically been screened before final optimization. For all calculations, the ORCA 6.1 program package has been used in refs. ^63,64^. If not stated otherwise, free energy corrections at 298.15 K have been computed using the standard harmonic oscillator approximation as implemented in the thermochemistry module of ORCA^73^.

## Supplementary information

Supplementary Information

Transparent Peer Review file

## Source data

Source Data

## Data Availability

The raw data of NMR experiments as well as DFT-calculated geometries generated in this study have been deposited in the Zenodo database^74^ under the accession code 10.5281/zenodo.18449765. Additional NMR datasets from the preliminary test studies are available from the corresponding author upon request. Source data for images of this article and its supplementary information are provided in the Source Data file (spreadsheet). Source data are provided with this paper.

## Code availability

All MATLAB scripts used to fit experimental kinetics are accessible via Zenodo^74^ 10.5281/zenodo.18449765.
