# Supplementary material for: Mechanistic insights into chemical exchange during the signal amplification by reversible exchange sensitization of pyruvate
Source: Nat Commun. 2026 Jun 25;17:7980. doi: 10.1038/s41467-026-74502-1 (PMC13448611; doi:10.1038/s41467-026-74502-1)
Supplement: Supplementary file 1 [file 41467_2026_74502_MOESM1_ESM.pdf]

Mechanistic Insights into Chemical Exchange during the Signal Amplification by Reversible Exchange Sensitization of Pyruvate

Charbel D. Assaf[†1], Vladimir V. Zhivonitko[*†2], Amaia Vicario[1,3], Alexander A. Auer[4], Simon B. Duckett[5], Jan-Bernd Hövener[1] and Andrey N. Pravdivtsev[*1]

†equal contribution
*corresponding authors

1 Section Biomedical Imaging (SBMI), Molecular Imaging North Competence Center (MOINCC), Department of Radiology and Neuroradiology, University Hospital Schleswig-Holstein, Kiel University, *Am Botanischen Garten 14/18*, 24118 Kiel, Germany. E-mail: andrey.pravdivtsev@rad.uni-kiel.de

2 NMR Research Unit, Faculty of Science, University of Oulu, P.O. Box 3000, Oulu, Finland. E-mail: vladimir.Zhivonitko@oulu.fi

3 University of Bath, Claverton Down, Bath, BA2 7AY, UK

4 Max-Planck-Institut für Kohlenforschung, Kaiser-Wilhelm-Platz 1, 45470 Mülheim an der Ruhr.

5 Centre for Hyperpolarization in Magnetic Resonance (CHyM), Department of Chemistry, University of York, Heslington YO10 5NY, UK

Contents:

1. Models of hydrogen exchange during SABRE S-4
   1.1. Model IrHH↔H_2 .... S-4
   1.2. Model (3↔2)↔H_2 .... S-6
2. Models of pyruvate exchange during SABRE S-10
   2.1. Model ($^{13}C^2$-3↔$^{13}C^2$-2)↔$^{13}C^2$-free S-10
3. Calibration of variable temperature NMR unit S-12
4. Signal enhancement and polarization calculations S-13
5. ^{1}H NMR Spectra of IrHH S-14
6. Analysis of chemical equilibria for 1-4 .... S-15
7. IrHH hydrogen exchange S-18
   7.1. SEPP-SABRE kinetics of IrHH hydrogens as a function of temperature S-18
      7.1.1. Complex 3 & 2 .... S-18
      7.1.2. Complex 1 .... S-20
   7.2. SEPP-SABRE kinetics of IrHH hydrogens as a function of pressure S-22
      7.2.1. Complex 3 & 2 .... S-22
      7.2.2. Complex 1 .... S-24
   7.3. SEPP-SABRE kinetics of IrHH hydrogens as a function of [DMSO] S-26
      7.3.1. Complex 3 & 2 .... S-26
      7.3.2. Complex 1 .... S-29
   7.4. SEPP-SABRE kinetics of IrHH hydrogens as a function of [pyruvate] S-30
      7.4.1. Complex 3 & 2 .... S-30
      7.4.2. Complex 1 .... S-32
   7.5 Thermal kinetics of IrHH exchange of 3 as a function of temperature S-34
8. Pyruvate exchange S-36
   8.1. SEPP-SPINEPTplus – SOT to hyperpolarize pyruvate S-36
   8.2. SEPP-SPINEPTplus-SABRE – SOT to measure pyruvate exchange S-36
   8.3. Pyruvate exchange kinetics of $^{13}C^2$ of 3 and 2 in the function of temperature with 20 mM of DMSO S-38
   8.4. Pyruvate exchange kinetics of $^{13}C^2$ of 3 and 2 in the function of temperature with 100mM of DMSO S-38
   8.5. Pyruvate exchange kinetics of $^{13}C^2$ of 3 and 2 in the function of pressure with 20 mM of DMSO S-40
   8.6. Pyruvate exchange kinetics of $^{13}C^2$ of 3 and 2 in the function of pressure with 100 mM of DMSO S-41
   8.7. Pyruvate exchange kinetics of $^{13}C^2$ of 3 and 2 in the function of DMSO S-42
   8.8. Pyruvate exchange kinetics of $^{13}C^2$ of 3 and 2 in the function of pyruvate S-43
9. Dissociation constant rates of $^{13}C^2$-3 & $^{13}C^2$-2 .... S-45

10. T_1 measurements for H_2 (4.6 ppm) and free-$^{13}C^2$ (202.5 ppm) as a function of temperature S-46
11. Exchange rates of $H^{a,b}$-1 .... S-47
12. Thermal polarized studies on complexes 1, 2 & 3 to determine their relative populations S-48
13. ^1H-^1H-NOE S-50
14. ^{13}C-DMSO-HMQC S-51
15. SEPP-SPINEPT of ^{13}C-DMSO S-52
16. Effect of intramolecular hydrogen exchange on SABRE S-53
17. Other hyperpolarized complexes S-55
18. Electronic Structure Calculations S-56
    18.1. Computational details S-56
    18.2. Additional influences on the relative stabilities of 2, 3, and 4 .... S-57
    18.3. Formation energy of complex 2 from complex 1 .... S-60
    18.4. Geometric parameters for 3 .... S-61
    18.5. Further DFT functional benchmarks S-61
19. Supplementary References S-65

1. Models of hydrogen exchange during SABRE

This section describes the kinetic models used to model ligand exchange, as described in the main text.

The first model, **IrHH↔H₂**, considers only exchange with one complex with free H_2 hydrogen.

The second model, **(3↔2)↔H₂,** considers how exchange between complexes affects H2 exchange by reference to complexes **2** and **3**.

1.1. Model IrHH↔H₂

Chemical exchange. The iridium complexes discussed feature two inequivalent hydride protons represented by IrHH. These two hydrogens can exchange within the complex, at an exchange rate $k_{\mathrm{IrH'H \to IrHH'}}$ and dissociafrom the complex at an exchange rate $k_{\mathrm{IrHH-H2}}$, both processed forming an intermediate Ir-complex. Finally, this intermediate can react with free $\mathrm{H_2}$ in the sample according to the pseudo-first-order rate constant ${k'_{\mathrm{a}}}^{\mathrm{H2}}$(valid if [$H_2$] is high and therefore invariant). Such chemical system can be represented by the following chemical equations:

$$\begin{aligned} \mathrm{H_2} &\xrightarrow{{k'_{\mathrm{a}}}^{\mathrm{H2}}} \mathrm{IrHH} \\ \mathrm{IrH'H} &\xrightarrow{k_{\mathrm{IrH'H \to IrHH'}}} \mathrm{IrHH'} \\ \mathrm{IrHH} &\xrightarrow{k_{\mathrm{IrHH-H2}}} \mathrm{H_2} \end{aligned} \quad (Eq.\ S1)$$

The apostrophe is used to highlight the exchange of the two chemically inequivalent hydrogens. Based on preserving molecularity, we consider that both the hydrides from the same complex dissociate at the same rate. In this form $1/k_{\mathrm{IrHH-H2}}$ is therefore the lifetime of the active Ir complex (IrHH) and $1/{k'_{\mathrm{a}}}^{\mathrm{H2}}$ is a characteristic time of $\mathrm{H_2}$ exchange. The chemical kinetics are given by the following kinetic equation:

$$\frac{d[\mathrm{H_2}]}{dt} = -\frac{d[\mathrm{IrHH}]}{dt} = +k_{\mathrm{IrHH-H2}}[\mathrm{IrHH}] - {k'_{\mathrm{a}}}^{\mathrm{H2}}[\mathrm{H_2}] \quad (Eq.\ S2)$$

Based on this equation, the steady-state equilibrium ratio of $[\mathrm{IrHH}]$, and $[\mathrm{H_2}]$ can be found:

$$\frac{{k'_{\mathrm{a}}}^{\mathrm{H2}}}{k_{\mathrm{IrHH-H2}}} = \frac{[\mathrm{IrHH}]}{[\mathrm{H_2}]} \quad (Eq.\ S3)$$

where [Ir] and [H_2] are the concentrations of the Ir precatalyst and natural hydrogen H_2.

Magnetization exchange (Inter and intramolecular exchange). By using Eq. S2 it is possible to derive how the evolution of magnetization, driven by relaxation and chemical exchange proceeds. The following approach is based on the Bloch-McConnell equation[1]. We define magnetization, for simplicity, as the product of polarization and concentration, which is therefore to a constant value, that does not affect to output of the calculations. The magnetization values for the constituents are therefore:

$$\begin{cases} M_{\mathrm{H_2}} = 2P_{\mathrm{H_2}}[\mathrm{H_2}] \\ M_{\mathrm{IrH^a}} = P_{\mathrm{IrH^a}}[\mathrm{IrHH}] \\ M_{\mathrm{IrH^b}} = P_{\mathrm{IrH^b}}[\mathrm{IrHH}] \\ M_{\mathrm{IrHH}} = M_{\mathrm{IrH^a}} + M_{\mathrm{IrH^b}} = \left(P_{\mathrm{IrH^a}} + P_{\mathrm{IrH^b}}\right)[\mathrm{IrHH}] \end{cases} \quad (Eq.\ S4)$$

P_{H_2} is the average polarization of two protons. The corresponding thermal equilibrium magnetization terms are $M^0_{IrH^a} = P^0[IrHH]$, $M^0_{IrH^b} = P^0[IrHH]$, $M^0_{H_2} = 2P^0[H_2]$ or $M^0_{IrHH} = M^0_{IrH^a} + M^0_{IrH^b}$, where P^0 is the thermal polarization of a ^{1}H nucleus.

The corresponding kinetic equation for magnetization within this system for IrHH, IrH^a, IrH^b, and H_2 is therefore as follows:

$$\begin{cases} \frac{dM_{H_2}}{dt} = -k'^{H2}_a M_{H_2} + k_{IrHH-H2}(M_{IrH^a} + M_{IrH^b}) - (M_{H_2} - M^0_{H_2})R_{H_2} \\ \frac{dM_{IrHH}}{dt} = +k'^{H2}_a M_{H_2} - k_{IrHH-H2} M_{IrHH} - (M_{IrHH} - M^0_{IrHH})R_{IrHH} \\ \frac{dM_{IrH^a}}{dt} = +\frac{k'^{H2}_a}{2} M_{H_2} - k_{IrHH-H2} M_{IrH^a} - k_{IrH'H \to IrHH'}(M_{IrH^a} - M_{IrH^b}) - (M_{IrH^a} - M^0_{IrH^a})R_{IrH^a} \\ \frac{dM_{IrH^b}}{dt} = +\frac{k'^{H2}_a}{2} M_{H_2} - k_{IrHH-H2} M_{IrH^b} + k_{IrH'H \to IrHH'}(M_{IrH^a} - M_{IrH^b}) - (M_{IrH^b} - M^0_{IrH^b})R_{IrH^b} \end{cases}$$

(*Eq. S5*)

An internal check is provided by the fact $\frac{dM_{IrHH}}{dt} = \frac{dM_{IrH^a}}{dt} + \frac{dM_{IrH^b}}{dt}$. Here we assumed $R_{IrH^a} \cong R_{IrH^b} \cong \frac{R_{IrH^a} + R_{IrH^b}}{2} = R_{IrHH}$, an assumption that provides a high order of accuracy. The three independent components, can be written in a matrix form as:

$$\frac{d}{dt}\begin{pmatrix} M_{IrH^a} \\ M_{IrH^b} \\ M_{H_2} \end{pmatrix} = \hat{L}\begin{pmatrix} M_{IrH^a} \\ M_{IrH^b} \\ M_{H_2} \end{pmatrix} + \begin{pmatrix} R_{IrHH} M^0_{IrH^a} \\ R_{IrHH} M^0_{IrH^b} \\ R_{H_2} M^0_{H_2} \end{pmatrix}$$

$$\hat{L} = \begin{pmatrix} -(R_{IrHH} + k_{IrHH-H2} + k_{ex}) & k_{IrH'H \to IrHH'} & \frac{k'^{H2}_a}{2} \\ k_{IrH'H \to IrHH'} & -(R_{IrHH} + k_{IrHH-H2} + k_{IrH'H \to IrHH'}) & \frac{k'^{H2}_a}{2} \\ k_{IrHH-H2} & k_{IrHH-H2} & -(R_{H_2} + k'^{H2}_a) \end{pmatrix}$$

(*Eq. S6*)

Hence, there are only five unknown parameters: $k_{IrHH-H2}$, k'^{H2}_a, $k_{IrH'H \to IrHH'}$, R_{IrHH} and R_{H_2}.

The general solution of the Bloch-McConnell equation, like (Eq. S6), is a superposition of exponentially decaying functions with decay rates equal to the eigenvalues of $\hat{L}$ plus the thermal magnetization $\vec{M}^0$ that can be calculated numerically using the following equation:

$$\vec{M}(d_{mix}) = e^{\hat{L} d_{mix}}[\vec{M}(d_{mix} = 0) - \vec{M}^0] + \vec{M}^0 \qquad (\textit{Eq. S7})$$

as a function of mixing time, d_{mix}. Here, $\vec{M}(d_{mix} = 0)$ is the initial magnetization of the system at $d_{mix} = 0$, which is, for example a result of the NMR pulse sequence preparation or spin labeling step. In case of hyperpolarization, $\vec{M}^0$ can be ignored, and the simplified solution can be calculated as follows:

$$\vec{M}(d_{mix}) = e^{\hat{L} d_{mix}} \vec{M}(d_{mix} = 0) \qquad (\textit{Eq. S8})$$

All hyperpolarization experiments were fitted using this solution with the appropriate $\vec{M}$ and $\hat{L}$ parameters as described in the corresponding model.

Magnetization exchange (Intermolecular exchange only, $M_{IrHH} = M_{IrH^a} + M_{IrH^b}$). The equations above can be significantly simplified by considering the sum of the magnetization

IrHH. Consequently, we need to consider only $\frac{dM_{\mathrm{H_2}}}{dt}$ and $\frac{dM_{\mathrm{IrHH}}}{dt}$ of Eq. S5. The resulting equations in the matrix form are:

$$\begin{cases} \mathrm{H_2} \xrightarrow{k'^{\mathrm{H2}}_{\mathrm{a}}} \mathrm{IrHH} \\ \mathrm{IrHH} \xrightarrow{k_{\mathrm{IrHH-H2}}} \mathrm{H_2} \end{cases}$$

$$\frac{d}{dt}\begin{pmatrix} M_{\mathrm{IrHH}} \\ M_{\mathrm{H_2}} \end{pmatrix} = \hat{L}\begin{pmatrix} M_{\mathrm{IrHH}} \\ M_{\mathrm{H_2}} \end{pmatrix} + \begin{pmatrix} R_{\mathrm{IrHH}} M^0_{\mathrm{IrHH}} \\ R_{\mathrm{H_2}} M^0_{\mathrm{H_2}} \end{pmatrix} \qquad (Eq.\ S9)$$

$$\hat{L} = \begin{pmatrix} -(R_{\mathrm{IrHH}} + k_{\mathrm{IrHH-H2}}) & k'^{\mathrm{H2}}_{\mathrm{a}} \\ k_{\mathrm{IrHH-H2}} & -(R_{\mathrm{H_2}} + k'^{\mathrm{H2}}_{\mathrm{a}}) \end{pmatrix}$$

This approach is convenient as it simplifies the system and allows us to readily obtain key H_2 exchange parameters, and then use them to find the rest using the model that includes inter- and intramolecular exchange.

This equation has 3 free parameters: R_{IrHH}, $k_{\mathrm{IrHH-H2}}$ and $k'^{\mathrm{H2}}_{\mathrm{a}}$ considering $R_{\mathrm{H_2}}$ is measured. When the ratio $\frac{k'^{\mathrm{H2}}_{\mathrm{a}}}{k_{\mathrm{IrHH-H2}}} = \frac{[\mathrm{IrHH}]}{[\mathrm{H_2}]}$ is measured by 1D spectroscopy at thermal equilibrium, then only 2 free parameters are left.

1.2. Model (3↔2)↔H_2

Chemical exchange. Experimentally, we observed chemical exchange between complexes **2** and **3** (**Figure 1**).

Complex **1** does not directly interconvert into **2** and **3**, but does produce free H_2; therefore, it can be treated separately using the previous model. The following model accounts for the chemical exchange of hydrogens in these two complexes.

$$\begin{cases} \mathrm{H_2} = \mathrm{H^x H^y} \xrightarrow{k'^{\mathrm{H_2}}_{3\mathrm{a}}} \frac{1}{2}\mathbf{3}^{\mathrm{xy}} + \frac{1}{2}\mathbf{3}^{\mathrm{yx}} \\ \mathrm{H_2} = \mathrm{H^x H^y} \xrightarrow{k'^{\mathrm{H_2}}_{2\mathrm{a}}} \frac{1}{2}\mathbf{2}^{\mathrm{xy}} + \frac{1}{2}\mathbf{2}^{\mathrm{yx}} \\ \mathbf{3}^{\mathrm{xy}} \xrightarrow{k^{\mathrm{H}}_{3-\mathrm{ex}}} \mathbf{3}^{\mathrm{yx}} \\ \mathbf{2}^{\mathrm{xy}} \xrightarrow{k^{\mathrm{H}}_{2-\mathrm{ex}}} \mathbf{2}^{\mathrm{yx}} \\ \mathbf{3}^{\mathrm{xy}} \xrightarrow{k_{3\to2}} \frac{1}{2}\mathbf{2}^{\mathrm{xy}} + \frac{1}{2}\mathbf{2}^{\mathrm{yx}} \\ \mathbf{2}^{\mathrm{xy}} \xrightarrow{k_{2\to3}} \frac{1}{2}\mathbf{3}^{\mathrm{xy}} + \frac{1}{2}\mathbf{3}^{\mathrm{yx}} \\ \mathbf{3}^{\mathrm{xy}} \xrightarrow{k_{3-\mathrm{H2}}} \mathrm{H_2} \\ \mathbf{2}^{\mathrm{xy}} \xrightarrow{k_{2-\mathrm{H2}}} \mathrm{H_2} \end{cases} \qquad (Eq.\ S10)$$

The model involves 8 exchange rate constants: pseudo-first-order association rate constants $k'^{\mathrm{H_2}}_{3\mathrm{a}}$, $k'^{\mathrm{H_2}}_{2\mathrm{a}}$, dissociation rate constants $k_{3-\mathrm{H_2}}$, $k_{2-\mathrm{H_2}}$ intra-complex exchange rate constants $k^{\mathrm{H}}_{3-\mathrm{ex}}$, $k^{\mathrm{H}}_{2-\mathrm{ex}}$ and inter-complex exchange rate constants $k_{3\to2}$, $k_{2\to3}$. The superscript "H" means this was measured using a ^{1}H experiment of hydride exchange, and **2** and **3** refer to the complex association, dissociation, or exchange it is related to. Here, we assume that both hydrides dissociate at the same rate under the same conditions. Finally, we also added superscripts to the complexes "xy". These labels represent the positions of two hydrides and are used here to indicate the changes in the positions of two protons. For example, the first position refers to a proton, with chemical shift H^a, and the second to a proton with chemical shift H^b in the respective complex. For H_2, both protons have the same chemical shift.

Using these chemical equations, the kinetic behavior is governed by the following equation:

$$\frac{d[\mathrm{H_2}]}{dt} = +k_{3-\mathrm{H_2}}[3] + k_{2-\mathrm{H_2}}[2] - (k_{3a}^{'\ \mathrm{H_2}} + k_{2a}^{'\ \mathrm{H_2}})[\mathrm{H_2}] \qquad \textit{(Eq. S11)}$$

From which a steady-state equilibrium can be obtained:

$$k_{3-\mathrm{H_2}}[3] + k_{2-\mathrm{H_2}}[2] = \left(k_{3a}^{'\ \mathrm{H_2}} + k_{2a}^{'\ \mathrm{H_2}}\right)[\mathrm{H_2}] \qquad \textit{(Eq. S12)}$$

Magnetization exchange (inter- and intramolecular exchange). As before, magnetization is proportional to concentration and polarization and can be defined for the constituents:

$$\begin{cases} M_{\mathrm{H_2}} = 2P_{\mathrm{H}}[\mathrm{H_2}] \\ M_{\mathbf{3}^{\mathrm{a}}} = P_{\mathbf{3}^{\mathrm{a}}}[3] \\ M_{\mathbf{3}^{\mathrm{b}}} = P_{\mathbf{3}^{\mathrm{b}}}[3] \\ M_{\mathbf{2}^{\mathrm{a}}} = P_{\mathbf{2}^{\mathrm{a}}}[2] \\ M_{\mathbf{2}^{\mathrm{b}}} = P_{\mathbf{2}^{\mathrm{b}}}[2] \\ M_{\mathbf{3}} = M_{\mathbf{3}^{\mathrm{a}}} + M_{\mathbf{3}^{\mathrm{b}}} = \left(P_{\mathbf{3}^{\mathrm{a}}} + P_{\mathbf{3}^{\mathrm{b}}}\right)[3] \\ M_{\mathbf{2}} = M_{\mathbf{2}^{\mathrm{a}}} + M_{\mathbf{2}^{\mathrm{b}}} = \left(P_{\mathbf{2}^{\mathrm{a}}} + P_{\mathbf{2}^{\mathrm{b}}}\right)[2] \end{cases} \qquad \textit{(Eq. S13)}$$

The corresponding thermal equilibrium magnetization $M_{\mathrm{H_2}}^0$, $M_{\mathbf{3}^{\mathrm{a}}}^0$, $M_{\mathbf{3}^{\mathrm{b}}}^0$, $M_{\mathbf{2}^{\mathrm{a}}}^0$, $M_{\mathbf{2}^{\mathrm{b}}}^0$, can again be obtained by substituting the polarization values with the thermal polarization P^0. Then, the corresponding kinetic equation for the magnetization of $\mathbf{3}$, $\mathbf{2}$, $\mathbf{3}^{\mathrm{a}}$, $\mathbf{3}^{\mathrm{b}}$, $\mathbf{2}^{\mathrm{a}}$, $\mathbf{2}^{\mathrm{b}}$, and H_2 is as follows:

$$\begin{cases} \frac{dM_{\mathbf{3}}}{dt} = k_{3a}^{'\ \mathrm{H_2}} M_{\mathrm{H_2}} - k_{3-\mathrm{H_2}} M_{\mathbf{3}} - k_{3\to2} M_{\mathbf{3}} + k_{2\to3} M_{\mathbf{2}} - R_{\mathbf{3}}\left(M_{\mathbf{3}} - M_{\mathbf{3}}^0\right) \\ \frac{dM_{\mathbf{2}}}{dt} = k_{2a}^{'\ \mathrm{H_2}} M_{\mathrm{H_2}} - k_{2-\mathrm{H_2}} M_{\mathbf{2}} - k_{2\to3} M_{\mathbf{2}} + k_{3\to2} M_{\mathbf{3}} - R_{\mathbf{2}}\left(M_{\mathbf{2}} - M_{\mathbf{2}}^0\right) \\ \frac{dM_{\mathbf{3}^{\mathrm{a}}}}{dt} = \frac{k_{3a}^{'\ \mathrm{H_2}}}{2} M_{\mathrm{H_2}} - k_{3-\mathrm{H_2}} M_{\mathbf{3}^{\mathrm{a}}} - k_{3-\mathrm{ex}}^{\mathrm{H}}\left(M_{\mathbf{3}^{\mathrm{a}}} - M_{\mathbf{3}^{\mathrm{b}}}\right) - k_{3\to2} M_{\mathbf{3}^{\mathrm{a}}} + \frac{k_{2\to3}}{2}\left(M_{\mathbf{2}^{\mathrm{a}}} + M_{\mathbf{2}^{\mathrm{b}}}\right) - R_{\mathbf{3}}\left(M_{\mathbf{3}^{\mathrm{a}}} - M_{\mathbf{3}^{\mathrm{a}}}^0\right) \\ \frac{dM_{\mathbf{3}^{\mathrm{b}}}}{dt} = \frac{k_{3a}^{'\ \mathrm{H_2}}}{2} M_{\mathrm{H_2}} - k_{3-\mathrm{H_2}} M_{\mathbf{3}^{\mathrm{b}}} + k_{3-\mathrm{ex}}^{\mathrm{H}}\left(M_{\mathbf{3}^{\mathrm{a}}} - M_{\mathbf{3}^{\mathrm{b}}}\right) - k_{3\to2} M_{\mathbf{3}^{\mathrm{b}}} + \frac{k_{2\to3}}{2}\left(M_{\mathbf{2}^{\mathrm{a}}} + M_{\mathbf{2}^{\mathrm{b}}}\right) - R_{\mathbf{3}}\left(M_{\mathbf{3}^{\mathrm{b}}} - M_{\mathbf{3}^{\mathrm{b}}}^0\right) \\ \frac{dM_{\mathbf{2}^{\mathrm{a}}}}{dt} = \frac{k_{2a}^{'\ \mathrm{H_2}}}{2} M_{\mathrm{H_2}} - k_{2-\mathrm{H_2}} M_{[2]^{\mathrm{a}}} - k_{2-\mathrm{ex}}^{\mathrm{H}}\left(M_{\mathbf{2}^{\mathrm{a}}} - M_{\mathbf{2}^{\mathrm{b}}}\right) - k_{2\to3} M_{\mathbf{2}^{\mathrm{a}}} + \frac{k_{3\to2}}{2}\left(M_{\mathbf{3}^{\mathrm{a}}} + M_{\mathbf{3}^{\mathrm{b}}}\right) - R_{\mathbf{2}}\left(M_{\mathbf{2}^{\mathrm{a}}} - M_{\mathbf{2}^{\mathrm{a}}}^0\right) \\ \frac{dM_{\mathbf{2}^{\mathrm{b}}}}{dt} = \frac{k_{2a}^{'\ \mathrm{H_2}}}{2} M_{\mathrm{H_2}} - k_{2-\mathrm{H_2}} M_{\mathbf{2}^{\mathrm{b}}} + k_{2-\mathrm{ex}}^{\mathrm{H}}\left(M_{\mathbf{2}^{\mathrm{a}}} - M_{\mathbf{2}^{\mathrm{b}}}\right) - k_{2\to3} M_{\mathbf{2}^{\mathrm{b}}} + \frac{k_{3\to2}}{2}\left(M_{\mathbf{3}^{\mathrm{a}}} + M_{\mathbf{3}^{\mathrm{b}}}\right) - R_{\mathbf{2}}\left(M_{\mathbf{2}^{\mathrm{b}}} - M_{\mathbf{2}^{\mathrm{b}}}^0\right) \\ \frac{dM_{\mathrm{H_2}}}{dt} = -\left(k_{3a}^{'\ \mathrm{H2}} + k_{2a}^{'\ \mathrm{H2}}\right) M_{\mathrm{H_2}} + k_{3-\mathrm{H_2}}\left(M_{\mathbf{3}^{\mathrm{a}}} + M_{\mathbf{3}^{\mathrm{b}}}\right) + k_{2-\mathrm{H_2}}\left(M_{\mathbf{2}^{\mathrm{a}}} + M_{\mathbf{2}^{\mathrm{b}}}\right) - R_{\mathrm{H_2}}\left(M_{\mathrm{H_2}} - M_{\mathrm{H_2}}^0\right) \end{cases}$$

(*Eq. S14*)

The relaxation rates of the hydrides ligands are assumed to be the same, $R_{\mathbf{3}^{\mathrm{a}}} \cong R_{\mathbf{3}^{\mathrm{b}}} \cong \frac{R_{\mathbf{3}^{\mathrm{a}}} + R_{\mathbf{3}^{\mathrm{b}}}}{2} = R_{\mathbf{3}}$ and $R_{\mathbf{2}^{\mathrm{a}}} \cong R_{\mathbf{2}^{\mathrm{b}}} \cong \frac{R_{\mathbf{2}^{\mathrm{a}}} + R_{\mathbf{2}^{\mathrm{b}}}}{2} = R_{\mathbf{2}}$ for each complex. Now, the sum of $\frac{dM_{\mathbf{2}^{\mathrm{a}}}}{dt} + \frac{dM_{\mathbf{2}^{\mathrm{b}}}}{dt}$ is $\frac{dM_{\mathbf{3}}}{dt}$, and $\frac{dM_{\mathbf{2}^{\mathrm{a}}}}{dt} + \frac{dM_{\mathbf{2}^{\mathrm{b}}}}{dt}$ is $\frac{dM_{\mathbf{2}}}{dt}$. These equations can then be written in a matrix form as:

$$\frac{d}{dt}\begin{pmatrix} M_{\mathbf{3}^{\mathrm{a}}} \\ M_{\mathbf{3}^{\mathrm{b}}} \\ M_{\mathbf{2}^{\mathrm{a}}} \\ M_{\mathbf{2}^{\mathrm{b}}} \\ M_{\mathrm{H_2}} \end{pmatrix} = \hat{L}\begin{pmatrix} M_{\mathbf{3}^{\mathrm{a}}} \\ M_{\mathbf{3}^{\mathrm{b}}} \\ M_{\mathbf{2}^{\mathrm{a}}} \\ M_{\mathbf{2}^{\mathrm{b}}} \\ M_{\mathrm{H_2}} \end{pmatrix} + \begin{pmatrix} R_{\mathbf{3}^{\mathrm{a}}} M^0_{\mathbf{3}^{\mathrm{a}}} \\ R_{\mathbf{3}^{\mathrm{b}}} M^0_{\mathbf{3}^{\mathrm{b}}} \\ R_{\mathbf{2}^{\mathrm{a}}} M^0_{\mathbf{2}^{\mathrm{a}}} \\ R_{\mathbf{2}^{\mathrm{b}}} M^0_{\mathbf{2}^{\mathrm{b}}} \\ R_{\mathrm{H_2}} M^0_{\mathrm{H_2}} \end{pmatrix} \qquad \textit{(Eq. S15)}$$

$$\hat{L} =$$

$$\begin{pmatrix} -(R_3 + k_{3-\mathrm{H_2}} + k^{\mathrm{H}}_{3-\mathrm{ex}} + k_{3\to2}) & k^{\mathrm{H}}_{3-\mathrm{ex}} & \frac{k_{2\to3}}{2} & \frac{k_{2\to3}}{2} & \frac{{k'_{3\mathrm{a}}}^{\mathrm{H2}}}{2} \\ k^{\mathrm{H}}_{3-\mathrm{ex}} & -(R_{\mathbf{3}} + k_{3-\mathrm{H_2}} + k^{\mathrm{H}}_{3-\mathrm{ex}} + k_{3\to2}) & \frac{k_{2\to3}}{2} & \frac{k_{2\to3}}{2} & \frac{{k'_{3\mathrm{a}}}^{\mathrm{H2}}}{2} \\ \frac{k_{3\to2}}{2} & \frac{k_{3\to2}}{2} & -(R_{\mathbf{2}} + k_{2-\mathrm{H_2}} + k^{\mathrm{H}}_{2-\mathrm{ex}} + k_{2\to3}) & k^{\mathrm{H}}_{2-\mathrm{ex}} & \frac{{k'_{2\mathrm{a}}}^{\mathrm{H2}}}{2} \\ \frac{k_{3\to2}}{2} & \frac{k_{3\to2}}{2} & k^{\mathrm{H}}_{2-\mathrm{ex}} & -(R_{\mathbf{2}} + k_{2-\mathrm{H_2}} + k^{\mathrm{H}}_{2-\mathrm{ex}} + k_{2\to3}) & \frac{{k'_{2\mathrm{a}}}^{\mathrm{H2}}}{2} \\ k_{3-\mathrm{H_2}} & k_{3-\mathrm{H_2}} & k_{2-\mathrm{H_2}} & k_{2-\mathrm{H_2}} & -(R_{\mathrm{H_2}} + {k'_{3\mathrm{a}}}^{\mathrm{H2}} + {k'_{2\mathrm{a}}}^{\mathrm{H2}}) \end{pmatrix}$$

Consequently, there are unknown parameters: $k_{3-\mathrm{H_2}}$, $k_{2-\mathrm{H_2}}$,${k'_{3\mathrm{a}}}^{\mathrm{H_2}}$, ${k'_{2\mathrm{a}}}^{\mathrm{H_2}}$, $k^{\mathrm{H}}_{3-\mathrm{ex}}$, $k^{\mathrm{H}}_{2-\mathrm{ex}}$, $R_{\mathbf{3}}$, $R_{\mathbf{2}}$, and $R_{\mathrm{H_2}}$. The general solution to this equation is Eq. S8.

Magnetization exchange (Intermolecular exchange only, $M_{\mathbf{3}} = M_{\mathbf{3}^{\mathrm{a}}} + M_{\mathbf{3}^{\mathrm{b}}}$, $M_{\mathbf{2}} = M_{\mathbf{2}^{\mathrm{a}}} + M_{\mathbf{2}^{\mathrm{b}}}$**)**. The equation above can be significantly simplified when we consider the magnetization of IrHH protons together. In this case, we need to consider only $\frac{dM_{\mathrm{H_2}}}{dt}$, $\frac{dM_{\mathbf{3}}}{dt}$ and $\frac{dM_{\mathbf{2}}}{dt}$ from Eq. S14. The resulting equations in the matrix form are:

$$\begin{cases} \mathrm{H_2} \xrightarrow{{k'_{3\mathrm{a}}}^{\mathrm{H_2}}} \mathbf{3} \\ \mathrm{H_2} \xrightarrow{{k'_{2\mathrm{a}}}^{\mathrm{H_2}}} \mathbf{2} \\ \mathbf{3} \xrightarrow{k_{3\to2}} \mathbf{2} \\ \mathbf{2} \xrightarrow{k_{2\to3}} \mathbf{3} \\ \mathbf{3} \xrightarrow{k_{3-\mathrm{H_2}}} \mathrm{H_2} \\ \mathbf{2} \xrightarrow{k_{2-\mathrm{H_2}}} \mathrm{H_2} \end{cases}$$

$$\frac{d}{dt}\begin{pmatrix} M_{\mathbf{3}} \\ M_{\mathbf{2}} \\ M_{\mathrm{H_2}} \end{pmatrix} = \hat{L}\begin{pmatrix} M_{\mathbf{3}} \\ M_{\mathbf{2}} \\ M_{\mathrm{H_2}} \end{pmatrix} + \begin{pmatrix} R_{\mathbf{3}} M^0_{\mathbf{3}} \\ R_{\mathbf{2}} M^0_{\mathbf{2}} \\ R_{\mathrm{H_2}} M^0_{\mathrm{H_2}} \end{pmatrix}$$

$$\hat{L} = \begin{pmatrix} -(k_{3-\mathrm{H_2}} + k_{3\to2} + R^{\mathrm{inter}}_{\mathbf{3}}) & k_{2\to3} & {k'_{3\mathrm{a}}}^{\mathrm{H_2}} \\ k_{3\to2} & -(k_{2-\mathrm{H_2}} + k_{2\to3} + R^{\mathrm{inter}}_{\mathbf{2}}) & {k'_{2\mathrm{a}}}^{\mathrm{H_2}} \\ k_{3-\mathrm{H_2}} & k_{2-\mathrm{H_2}} & -\left({k'_{3\mathrm{a}}}^{\mathrm{H_2}} + {k'_{2\mathrm{a}}}^{\mathrm{H_2}} + R_{\mathrm{H_2}}\right) \end{pmatrix}$$

(*Eq. S16*)

This is convenient as it simplifies the system, meaning some exchange parameters can be defined and used in a expanded model including inter- and intramolecular exchange. Setting $R^{\mathrm{inter}}_{\mathbf{3}} = R^{\mathrm{inter}}_{\mathbf{2}}$, and measuring $R_{\mathrm{H_2}}$, we have 7 free parameters: $k_{3\to2}$, $k_{2\to3}$, $k_{3-\mathrm{H_2}}$, $k_{2-\mathrm{H_2}}$, ${k'_{3\mathrm{a}}}^{\mathrm{H2}}$, ${k'_{2\mathrm{a}}}^{\mathrm{H2}}$ and $R^{\mathrm{inter}}_{\mathbf{3}} = R^{\mathrm{inter}}_{\mathbf{2}}$. When these parameters are obtained, we can estimate $k^{\mathrm{H}}_{3-\mathrm{ex}}$ and $k^{\mathrm{H}}_{2-\mathrm{ex}}$ using inter- and intramolecular exchange models (Eq. S15). The measured $R_{\mathrm{H_2}}$ values at different temperatures are presented in **Table S15**, and prove to be of the order 1.9 s and decrease with high temperature, reaching 1.5 s at 300 K as in Ref.[2]

Because of observable cross-relaxation effects between protons of the same complex, the obtained relaxation rates $R^{\text{inter}} = R^{\text{inter+intra}} + \sigma$ for complexes **3** and **2** (see Solomon equations for the explanation). The σ_3 and σ_2 in the inter- and intramolecular exchange model therefore is included in the observed $k_{3-\text{ex}}^{\text{H}}$ and $k_{2-\text{ex}}^{\text{H}}$ rate constants. Different R parameters were therefore used in the two models.

Additional constraints on the parameters can be set according to data from the thermal equilibrium experiments. The stationary conditions from (Eq. S13) are

$$\begin{cases} k_{3\text{a}}'^{\text{H2}}[\text{H}_2] = (k_{3-H_2} + k_{3\to 2})[3] - k_{2\to 3}[2] \\ k_{2\text{a}}'^{\text{H2}}[\text{H}_2] = (k_{2-H_2} + k_{2\to 3})[2] - k_{3\to 2}[3] \\ k_{3-\text{H}_2}[3] + k_{2-\text{H}_2}[2] = \left(k_{3\text{a}}'^{\text{H2}} + k_{2\text{a}}'^{\text{H2}}\right)[\text{H}_2] \end{cases} \qquad \textit{(Eq. S17)}$$

For $[3] = f_{3,2}[2]$, the first two equations can be divided and one obtains:

$$k_{3\text{a}}'^{\text{H}_2} = k_{2\text{a}}'^{\text{H}_2} \frac{(k_{3-\text{H}_2} + k_{3\to 2})f_{3,2} - k_{2\to 3}}{(k_{2-\text{H}_2} + k_{2\to 3}) - k_{3\to 2}f_{3,2}} \qquad \textit{(Eq. S18)}$$

Hence, when $f_{3,2} = [3]/[2]$ ratio is known, the number of parameters goes down to 6: $k_{3\to 2}$, $k_{2\to 3}$, $k_{3-\text{H}_2}$, $k_{2-\text{H}_2}$, $k_{2\text{a}}'^{\text{H}_2}$, and $R_{\mathbf{3}}^{\text{inter}} = R_{\mathbf{2}}^{\text{inter}}$ (or to 7 if $R_{\mathbf{3}}^{\text{inter}} \neq R_{\mathbf{2}}^{\text{inter}}$). Using the parameters estimated this way, one can apply them to the inter and intramolecular exchange model, which will have four free parameters: $R_{\mathbf{3}}$, $R_{\mathbf{2}}$, $k_{3-\text{ex}}^{\text{H}}$, $k_{2-\text{ex}}^{\text{H}}$.

2. Models of pyruvate exchange during SABRE

Since we observed exchange between complexes **2** and **3**, it is not reasonable to model the exchange of only one complex without the other; hence, only a model with two Ir complexes will be considered here.

2.1. Model ($^{13}C^2$-3↔$^{13}C^2$-2)↔$^{13}C^2$-free

The model is analogous to Model (**2**↔**3**)↔H_2 with only a few modifications:

$$\begin{pmatrix} \mathrm{pyr} \xrightarrow{k'^{\ \mathrm{P}}_{3\mathrm{a}}} \mathbf{3} \\ \mathrm{pyr} \xrightarrow{k'^{\ \mathrm{P}}_{2\mathrm{a}}} \mathbf{2} \\ \mathbf{3} \xrightarrow{k_{3\to 2}} \mathbf{2} \\ \mathbf{2} \xrightarrow{k_{2\to 3}} \mathbf{3} \\ \mathbf{3} \xrightarrow{k_{3\to \mathrm{pyr}}} \mathrm{pyr} \\ \mathbf{2} \xrightarrow{k_{2\to \mathrm{pyr}}} \mathrm{pyr} \end{pmatrix} \qquad \textit{(Eq. S19)}$$

Here, superscript “P” refers to the values measured by observing pyruvate exchange. The meaning of all exchange rate constants is as before. The corresponding chemical kinetic equations are

$$\begin{cases} \frac{d[1]}{dt} = -\left(k_{3\to\mathrm{pyr}} + k_{3\to 2}\right)[3] + k_{2\to 3}[2] + k'^{\ \mathrm{P}}_{3\mathrm{a}}[\mathrm{pyr}] \\ \frac{d[2]}{dt} = +k_{3\to 2}[3] - \left(k_{2\to\mathrm{pyr}} + k_{2\to 3}\right)[2] + k'^{\ \mathrm{P}}_{2\mathrm{a}}[\mathrm{pyr}] \\ \frac{d[\mathrm{pyr}]}{dt} = +k_{3\to\mathrm{pyr}}[3] + k_{2\to\mathrm{pyr}}[2] - (k'^{\ \mathrm{P}}_{3\mathrm{a}} + k'^{\ \mathrm{P}}_{2\mathrm{a}})[\mathrm{pyr}] \end{cases} \qquad \textit{(Eq. S20)}$$

Corresponding steady-state concentrations of $[\mathrm{pyr}]$ and catalysts are:

$$\begin{cases} k'^{\ \mathrm{P}}_{3\mathrm{a}}[\mathrm{pyr}] = \left(k_{3\to\mathrm{pyr}} + k_{3\to 2}\right)[3] - k_{2\to 3}[2] \\ k'^{\ \mathrm{P}}_{2\mathrm{a}}[\mathrm{pyr}] = \left(k_{2\to\mathrm{pyr}} + k_{2\to 3}\right)[2] - k_{3\to 2}[3] \\ k_{3\to\mathrm{pyr}}[3] + k_{2\to\mathrm{pyr}}[2] = \left(k'^{\ \mathrm{P}}_{3\mathrm{a}} + k'^{\ \mathrm{P}}_{2\mathrm{a}}\right)[\mathrm{pyr}] \end{cases} \qquad \textit{(Eq. S21)}$$

For $[3] = f_{3,2}[2]$, the first two equations can be divided, and one obtains:

$$k'^{\ \mathrm{P}}_{3\mathrm{a}} = k'^{\ \mathrm{P}}_{2\mathrm{a}} \frac{(k_{3\to\mathrm{pyr}}+k_{3\to 2})f_{3,2} - k_{2\to 3}}{(k_{2\to\mathrm{pyr}}+k_{2\to 3}) - k_{3\to 2}f_{3,2}} \qquad \textit{(Eq. S22)}$$

The magnetization is proportional to concentration and polarization, and for the discussed compounds is:

$$\begin{cases} M_{\mathbf{3}} = P_{\mathbf{3}}[\mathbf{3}] \\ M_{\mathbf{2}} = P_{\mathbf{2}}[\mathbf{2}] \\ M_{\mathrm{pyr}} = P_{\mathrm{pyr}}[\mathrm{pyr}] \end{cases} \qquad \textit{(Eq. S23)}$$

The corresponding thermal equilibrium magnetization $M^0_{\mathbf{3}}$, $M^0_{\mathbf{2}}$, or $M^0_{[\mathrm{pyr}]}$ can be obtained by substituting the polarization values with the thermal polarization P^0. Then, the corresponding kinetic equations for magnetization of **3**, **2**, and pyr are as follows:

$$\begin{cases} \frac{dM_3}{dt} = +{k'_{3a}}^{\mathrm{P}} M_{\mathrm{pyr}} - \left(k_{3\to\mathrm{pyr}} + k_{3\to2}\right) M_{\mathbf{3}} + k_{2\to3} M_{\mathbf{2}} - \left(M_{\mathbf{3}} - M_{\mathbf{3}}^0\right) R_{\mathbf{3}} \\ \frac{dM_2}{dt} = +{k'_{2a}}^{\mathrm{P}} M_{\mathrm{pyr}} + k_{3\to2} M_{\mathbf{3}} - \left(k_{2\to\mathrm{pyr}} + k_{2\to3}\right) M_{\mathbf{2}} - \left(M_{\mathbf{2}} - M_{\mathbf{2}}^0\right) R_{\mathbf{2}} \\ \frac{dM_{\mathrm{pyr}}}{dt} = -\left({k'_{3a}}^{\mathrm{P}} + {k'_{2a}}^{\mathrm{P}}\right) M_{\mathrm{pyr}} + k_{3\to\mathrm{pyr}} M_{\mathbf{3}} + k_{2\to\mathrm{pyr}} M_{\mathbf{2}} - \left(M_{\mathrm{pyr}} - M_{\mathrm{pyr}}^0\right) R_{\mathrm{pyr}} \end{cases} \quad \textit{(Eq S24)}$$

In a matrix form, this can be written as follows:

$$\frac{\mathrm{d}}{\mathrm{dt}} \begin{pmatrix} M_{\mathbf{3}} \\ M_{\mathbf{2}} \\ M_{\mathrm{pyr}} \end{pmatrix} = \hat{L} \begin{pmatrix} M_{\mathbf{3}} \\ M_{\mathbf{2}} \\ M_{\mathrm{pyr}} \end{pmatrix} + \begin{pmatrix} R_{\mathbf{3}} M_{\mathbf{3}}^0 \\ R_{\mathbf{2}} M_{\mathbf{2}}^0 \\ R_{\mathrm{pyr}} M_{\mathrm{pyr}}^0 \end{pmatrix}$$

$$\hat{L} = \begin{pmatrix} -\left(R_{\mathbf{3}} + k_{3\to\mathrm{pyr}} + k_{3\to2}\right) & k_{2\to3} & {k'_{3a}}^{\mathrm{P}} \\ k_{3\to2} & -\left(R_{\mathbf{2}} + k_{2\to\mathrm{pyr}} + k_{2\to3}\right) & {k'_{2a}}^{\mathrm{P}} \\ k_{3\to\mathrm{pyr}} & k_{2\to\mathrm{pyr}} & -\left(R_{\mathrm{pyr}} + {k'_{3a}}^{\mathrm{P}} + {k'_{2a}}^{\mathrm{P}}\right) \end{pmatrix} \quad \textit{(Eq S25)}$$

Consequently, there are unknown parameters: $k_{3\to\mathrm{pyr}}$, $k_{2\to\mathrm{pyr}}$, ${k'_{3a}}^{\mathrm{P}}$, ${k'_{2a}}^{\mathrm{P}}$, $k_{2\to3}$, $k_{3\to2}$, $R_{\mathbf{3}}$, $R_{\mathbf{2}}$, and R_{pyr}. Setting $R_{\mathbf{3}} = R_{\mathbf{2}}$ and measuring R_{pyr} for pyruvate in H_2 bubbled methanol without the catalyst (**Table S15**), leaves only 7 independent parameters. When the ratio $f_{3,2} = [3]/[2]$, the number of parameters goes down to 6 using Eq. S25.

3. Calibration of variable temperature NMR unit

We calibrated the variable temperature unit (VTU) of the NMR spectrometer using a method sample, of observing the chemical shift separation Δ between its OH and CH_3 resonances (calibration sample temperature method provided by Bruker). We prepared a 600 µL of 4:96 mixture of isotopically unlabeled methanol and methanol-d_4 and measured its ^{1}H NMR spectra at different temperatures. Then, we used the following two equations to calculate the actual temperature of the sample, as in the Bruker Instruments Manual for VT-Calibration:

1) For values between 230 – 270 K:

$$T = (3.92 - \Delta)/0.008 \quad (Eq.\ S26)$$

2) For more accurate values between 270 – 300 K, we used this equation:

$$T = (4.109 - \Delta)/0.008708 \quad (Eq.\ S27)$$

Table S1. The user set the nominal NMR temperature, and the calibrated temperature was calculated using Eqs. S26 and S27. Deviation indicates the relative deviation between the calibrated and the nominal temperatures.

Nominal NMR *T* (K)	Calibrated *T* (K)	Deviation (%)
260	257.5	-0.96
263	260	-1.14
267	265	-0.75
270	267.5	-0.92
273	270.9	-0.77
277	275.5	-0.54
280	277.8	-0.79
283	281.2	-0.62
288	287	-0.35

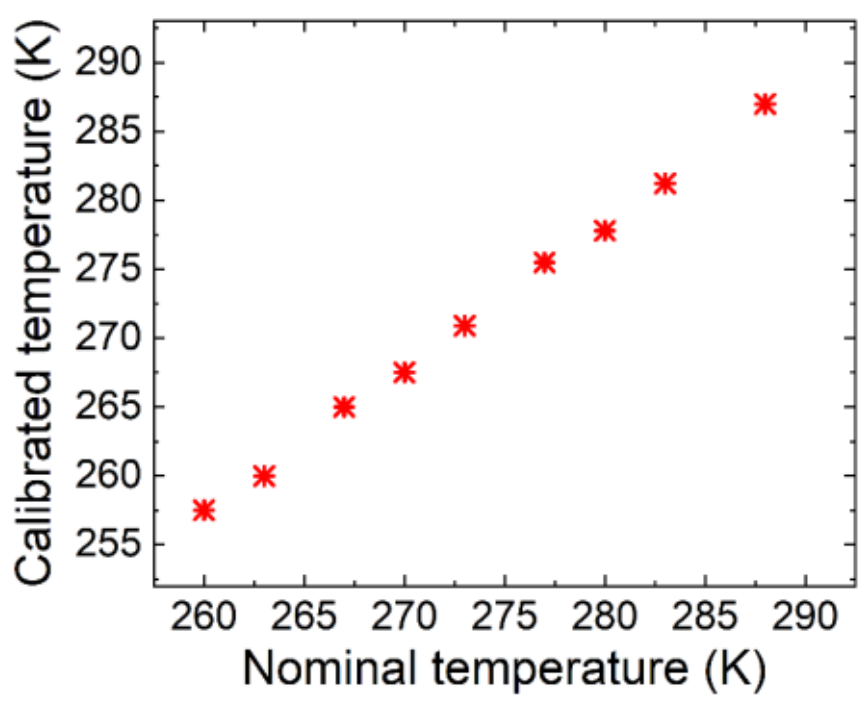

Figure S1: Calibrated temperature measured from chemical shift difference of methanol peaks, Δ, as a function of nominal temperature. Source data are provided as a Source Data file.

4. Signal enhancement and polarization calculations

Signal enhancement and polarization

The enhancement factor ε of substrates was calculated using the signal intensities of the spectra of thermally polarized and hyperpolarized solutions, taking into account the differences in acquisition parameters. The following formula was used when the signals had net magnetization:

$$\varepsilon = \frac{I^{\mathrm{HP}}}{I^{\mathrm{TP}}} \times \frac{\sin\left(\alpha^{\mathrm{TP}}\right)}{\sin\left(\alpha^{\mathrm{HP}}\right)} \times \frac{RG^{\mathrm{TP}}}{RG^{\mathrm{HP}}} \times \frac{NS^{\mathrm{TP}}}{NS^{\mathrm{HP}}} \qquad \textit{(Eq. S28)}$$

where I^{HP} is the integral of the hyperpolarized signal and I^{TP} is the integral of the thermally polarized signal of the same species. NS^{TP} and NS^{HP} are the numbers of scans for the thermally polarized and hyperpolarized samples, and α^{TP} and α^{HP} are the excitation angles of RF pulses used to acquire the corresponding spectra. RG^{TP} and RG^{HP} are values of linear receiver gain.

In the case of ^{1}H PASADENA polarization, the integral of only one ^{1}H proton was considered after the application of the SEPP SOT; the corresponding excitation angle is $\sin\left(\alpha^{\mathrm{HP}}\right) = 1$.

To compare the enhancements at 9.4 T more easily, the enhancement factors ε were converted to a polarization level, P, using Eq. S28, where, γ is the gyromagnetic ratio, B_0 is the detection field, T is the temperature, $\hbar$ is the reduced Planck's constant, and k_B is Boltzmann's constant.

$$P = \varepsilon \frac{\gamma B_0 \hbar}{2\, k_{\mathrm{B}} T} = \varepsilon P^{\mathrm{TP}} \qquad \textit{(Eq. S29)}$$

The example thermal polarization values at 9.4 T and 300 K are $P^{\mathrm{TP}}({}^{1}\mathrm{H}) \cong 3.6 \cdot 10^{-5}$ and $P^{\mathrm{TP}}({}^{13}\mathrm{C}) \cong 8.05 \cdot 10^{-6}$.

Line-enhancement in PASADENA: In the case of the ^{1}H PASADENA experiment, we integrated separately two lines of the antiphase PASADENA spectrum of a given ^{1}H resonance and then subtracted them (or added together absolute values) which gave us an integral over the PASADENA line, I^{PASADENA}, of one ^{1}H spin. This value is very close to the integral over the spectral line when it is given in amplitude mode. Since two IrHH protons are polarized, we used an average integral value for each complex: $I_{\mathrm{avg}}^{\mathrm{PASADENA}} = 0.5(I_{\mathrm{H^a}}^{\mathrm{PASADENA}} + I_{\mathrm{H^b}}^{\mathrm{PASADENA}})$. We used the modification of Eq. S28 to calculate the signal enhancement as

$$\varepsilon^{PASADENA} = \frac{I_{\mathrm{avg}}^{\mathrm{PASADENA}}}{I^{\mathrm{TP}}} \times \frac{\sin\left(\alpha^{\mathrm{TP}}\right)}{\sin\left(2\alpha^{\mathrm{HP}}\right)} \times \frac{RG^{\mathrm{TP}}}{RG^{\mathrm{HP}}} \times \frac{NS^{\mathrm{TP}}}{NS^{\mathrm{HP}}} \qquad \textit{(Eq. S30)}$$

Note that here, the angle dependence is $\sin\left(2\alpha^{\mathrm{HP}}\right)$, hence for $\alpha^{\mathrm{HP}} = 45^{\circ}$, it is 1 (maximum signal in the PASADENA experiment). To calculate the polarization of PASADENA two spin order, $\hat{I}_{\mathrm{z}}^{\mathrm{H^a}} \hat{I}_{\mathrm{z}}^{\mathrm{H^b}}$, one should use the following equation:

$$P_{\mathrm{zz}} = 2\varepsilon^{PASADENA} P^{\mathrm{TP}} \qquad \textit{(Eq. S31)}$$

The additional factor of 2 reflects that the PASADENA signal is measured with a 45° pulse so ½ of the signal results relative to a spin with 100% net magnetization. Derivation of the net and PASADENA signals is discussed in the SI of Ref.[3]. When $P_{\mathrm{zz}} = 1$ is reached, it means that 100% pH_2 was used, and there were no losses in spin order during the experiment.

5. ^{1}H NMR Spectra of IrHH

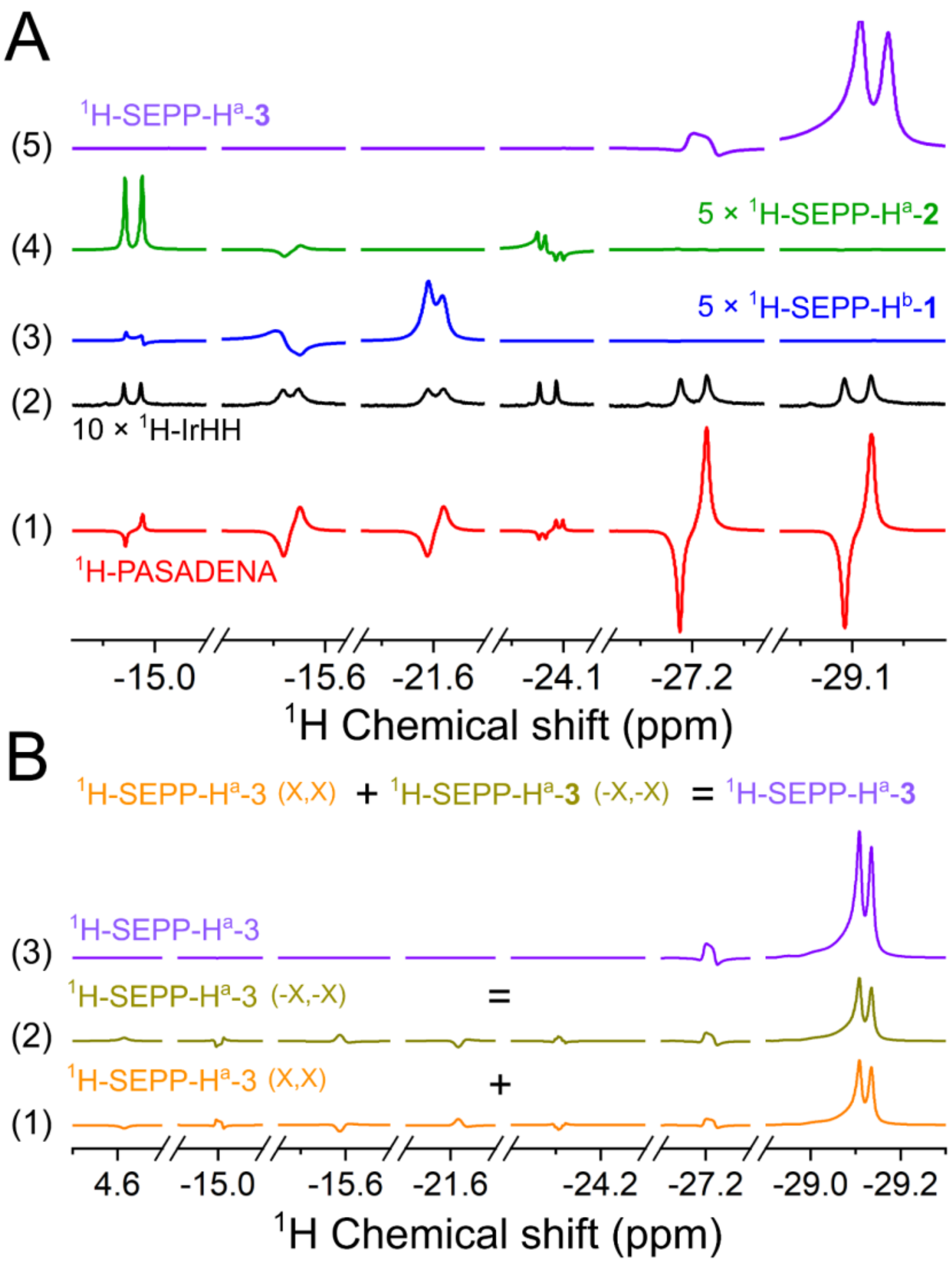

Figure S2: ^{1}H NMR spectra of IrHH protons. (A.1)^{1}H-PASADENA (NS = 1, $\alpha = 45^{o}$) (red),(A. 2) ^{1}H thermally polarized spectrum (NS = 100, TR = 9 s, $\alpha = 90^{o}$) (black), (A. 3) ^{1}H-SEPP of H^{b}-**1** (-21.59 ppm, t_1 = 26 ms, t_2 = 0 ms, Δ = 10 ms) (blue), (A. 4) ^{1}H-SEPP of H^{a}-**2** (-14.98 ppm, t_1 = 26 ms, t_2 = 0 ms, Δ = 10 ms) (green), and (A. 5) ^{1}H-SEPP of H^{a}-**3** (-29.1 ppm, t_1 = 23.8 ms, t_2 = 0 ms, Δ = 10 ms) (purple). (B. 1) ^{1}H-SEPP of H^{a}-**3** (-29.1 ppm, t_1 = 23.8 ms, t_2 = 0 ms, Δ = 10 ms) with phase (X, X) of the first 90° pulse and recording phase (orange). (B. 2) ^{1}H-SEPP of H^{a}-**3** (-29.1 ppm, t_1 = 23.8 ms, t_2 = 0 ms, Δ = 10 ms) with phase (-X, -X) of the first 90° pulse and recording phase (dark yellow). (B. 1) ^{1}H-SEPP of H^{a}-**3** (-29.1 ppm, t_1 = 23.8 ms, t_2 = 0 ms, Δ = 10 ms) after 2 scans (B.1) + (B.2) (purple). Sample compositions and conditions: [Ir] = 4mM, [2-^{13}C-pyr] = 40mM, [DMSO] = 20 mM, in methanol-d_4 at 8.5 bar and 265 K, with 10 s bubbling time, and a settling time of 1.5 s. Achieved signal enhancements and polarization for SEPP spectra are 1995, 8.28% (A. 3), 1053, 4.36% (A. 4), 2723, 11.3% (A. 5). Achieved signal enhancements and polarization in PASADENA spectrum are 8058, 66.89% **3**, 2320, 19.26% **2**, 5717, 47.45% **1**. Source data are provided as a Source Data file.

6. Analysis of chemical equilibria for 1-4

2-3 and 3-4: To assess the connection between equilibrium concentrations of complexes **2**, **3**, and **4**, it is convenient to consider the following thermodynamic equilibria with the corresponding equilibrium constants $K_{\{2\leftarrow3\}}$ and $K_{\{4\leftarrow3\}}$:

$$\mathbf{3} + \text{DMSO} \overset{K_{\{2\leftarrow3\}}}{\rightleftharpoons} \mathbf{2} \qquad (Eq.\ S32)$$

where

$$K_{\{2\leftarrow3\}} = \frac{[\mathbf{2}]}{[\mathbf{3}][\text{DMSO}]} \Rightarrow \frac{[\mathbf{2}]}{[\mathbf{3}]} = K_{\{2\leftarrow3\}}[\text{DMSO}] \qquad (Eq.\ S33)$$

And

$$\mathbf{3} \overset{K_{\{4\leftarrow3\}}}{\rightleftharpoons} \mathbf{4} \qquad (Eq.\ S34)$$

Where

$$K_{\{4\leftarrow3\}} = \frac{[\mathbf{4}]}{[\mathbf{3}]} \qquad (Eq.\ S35)$$

The obtained ratios of equilibrium concentrations reveal a clear difference since the [**2**]/[**3**] ratio is dependent on the equilibrium concentration of DMSO (see Eq. S33), whereas the [**4**]/[**3**] ratio must be DMSO concentration independent (see Eq. S35). The latter conclusion comes from the fact that complexes **3** and **4** are structural isomers, and their equilibrium concentration ratio is defined solely by the thermodynamic chemical potential of only these two species. In other words, since there are no other species involved, [**4**]/[**3**] becomes concentration independent.

Since the analysis of concentration dependencies for **2** and **3** shown in **Figure 3D** of the main text demonstrates an apparent increase of [**2**] and decrease of [**3**] with the total DMSO concentration (**3** → **2** transformation), it provides additional evidence that the hydride signals at -14.93 and -24.02 ppm in ^{1}H NMR belong to complex **2** because it matches expectation on the DMSO concentration dependence for the [**2**]/[**3**] ratio (Eq. S33). If those resonances were to belong to **4**, the complex concentration ratio would remain unchanged, as discussed above.

Moreover, the dependence of the kinetic rate for the **3**→**2** transformation on [DMSO] shown in **Figure 5H** supports the hydride signal assignment of **2,** since this process will also be DMSO concentration dependent according to the equilibrium Eq. S32, whereas such a kinetic rate would be concentration independent in the case of equilibrium Eq. S34.

To determine the standard Gibbs free energy change for the **2**←**3** transition, we assumed that

$$K_{\{2\leftarrow3\}} = \frac{[\mathbf{2}]}{[\mathbf{3}][\text{DMSO}]} = \mathrm{e}^{-\frac{\Delta G^0_{2\leftarrow3}}{RT}}. \qquad (Eq.\ S36)$$

where, $\Delta G^0_{\mathbf{2}\leftarrow\mathbf{3}} = \Delta G^0_{\mathbf{2}} - \Delta G^0_{\mathbf{3}}$. Rearranging this equation and assuming $\Delta G^0_{2\leftarrow3} = \Delta H^0_{2\leftarrow3} - T\Delta S^0_{2\leftarrow3}$ gives:

$$\ln\left(\frac{[\mathbf{2}]}{[\mathbf{3}][\text{DMSO}]}\right) = -\frac{\Delta H^0_{2\leftarrow3}}{R} \times \frac{1}{T} + \frac{\Delta S^0_{2\leftarrow3}}{R} \qquad (Eq.\ S37)$$

A plot of $\ln\left(\frac{[\mathbf{2}]}{[\mathbf{3}][\mathrm{DMSO}]}\right)$ as a function of $\frac{1}{T}$ yields a straight line, where the slope corresponds to $-\frac{\Delta H^0_{2\leftarrow3}}{R}$ and the constant term to $\frac{\Delta S^0_{2\leftarrow3}}{R}$. Utilizing the equilibrium concentrations from **Table S2,** this linearization equation was used to fit experimental dependency (**Figure S3B**) and obtain $\Delta H^0_{2\leftarrow3}$, $\Delta S^0_{2\leftarrow3}$, and $\Delta G^0_{2\leftarrow3}$ in **Table S3**.

1-3: Similarly, the transformation from **3** to **1** can be analyzed using the following equation,

$$\mathbf{3} + \mathrm{DMSO} + \mathrm{Cl}^- \overset{K_{\{\mathbf{1}\leftarrow\mathbf{3}\}}}{\rightleftharpoons} \mathbf{1} + \mathrm{pyr} \quad (Eq.\ S38)$$

leading to

$$K_{\{\mathbf{1}\leftarrow\mathbf{3}\}} = \frac{[\mathbf{1}][\mathrm{pyr}]}{[\mathbf{3}][\mathrm{DMSO}][\mathrm{Cl}^-]} \Rightarrow \frac{[\mathbf{1}]}{[\mathbf{3}]} = K_{\{\mathbf{1}\leftarrow\mathbf{3}\}}\frac{[\mathrm{DMSO}][\mathrm{Cl}^-]}{[\mathrm{pyr}]} \quad (Eq.\ S39)$$

Performing the necessary rearrangements and assuming $\Delta G^0_{1\leftarrow3} = \Delta H^0_{1\leftarrow3} - T\Delta S^0_{1\leftarrow3}$ gives:

$$\ln\left(\frac{[\mathbf{1}][\mathrm{pyr}]}{[\mathbf{3}][\mathrm{DMSO}][\mathrm{Cl}^-]}\right) = -\frac{\Delta H^0_{1\leftarrow3}}{R} \times \frac{1}{T} + \frac{\Delta S^0_{1\leftarrow3}}{R}. \quad (Eq.\ S40)$$

Utilizing the equilibrium concentrations from **Table S2,** this linearization equation was used to fit experimental dependency (**Figure S3C**) and obtain $\Delta H^0_{1\leftarrow3}$, $\Delta S^0_{1\leftarrow3}$, and $\Delta G^0_{1\leftarrow3}$ in **Table S3**.

Table S2: Equilibrium concentrations of complexes 1-9 were estimated from the NMR spectra (**Figure S3A**) measured at different temperatures. Nominal concentrations of constituents $[\mathrm{DMSO}]^0$ = 20 mM, $[\mathrm{pyr}]^0$ = 40 mM, $[\mathrm{Ir}]^0$ = 4 mM, and $[\mathrm{Cl}^-]^0$ = 4 mM, and their corresponding estimated equilibrium concentrations [DMSO], [pyr] and $[\mathrm{Cl}^-]$.

C, mM	**1**	**2**	**3**	**7**	**8**	**9**	-25.84	-26.47	[DMSO]	[pyr]	$[\mathrm{Cl}^-]$
DMSO	2	2	1	1	2	2	X	X			
Cl	1	0	0	0	0	0	X	X			
Pyr	0	1	1	0	0	0	X	X			
220 K	0.79	1.51	0.1	0.27	0.83	0.05	0.15	0.24	12.76	38.3	3.20
235 K	1.16	1.02	0.51	0.08	0.77	0.06	0.18	0.26	12.92	38.4	2.84
247 K	1.13	0.81	0.98	0.03	0.72	0.02	0.10	0.23	13.30	38.2	2.87
259 K	0.77	0.5	1.48	0.13	0.78	0.02	0.11	0.21	13.89	38.1	3.22
260 K	0.58	0.31	1.19	0.90	0.77	0.05	0.08	0.18	14.24	38.5	3.42
265 K	0.45	0.24	1.06	1.19	0.75	0.08	0.09	0.20	14.38	38.6	3.54
267.5 K	0.35	0.21	1.13	1.27	0.72	0.05	0.087	0.23	14.63	38.7	3.65
270.9 K	0.30	0.20	1.13	1.42	0.66	0.10	0.07	0.22	14.64	38.7	3.70
275.5 K	0.22	0.15	1.16	1.50	0.64	0.12	0.09	0.24	14.77	38.7	3.78

The equilibrium concentrations (**Table S2**) were used together with Eq S37 and S40 to estimate $\Delta G^0_{2\leftarrow3}$ and $\Delta G^0_{1\leftarrow3}$ (**Figure S3B,C**).

Table S3: Fitted from temperature dependences, $\Delta H^0_{2\leftarrow3}$ = -(39 ± 2) kJ/mol, $\Delta S^0_{2\leftarrow3}$ = -(126 ± 9) J/(mol K) for transition **2-3** and $\Delta H^0_{1\leftarrow3}$ = -(39 ± 2) kJ/mol $\Delta S^0_{1\leftarrow3}$ = -(99 ± 9) J/(mol K) for transition **1-3**. Here for example $\Delta G^0_{2\leftarrow3} = \Delta G^0_{\mathbf{2}} - \Delta G^0_{\mathbf{3}}$.

	Experimental	
Temperature (K)	$\Delta G^0_{1\leftarrow 3}$ (J/mol)	$\Delta G^0_{2\leftarrow 3}$ (J/mol)
235	-15430	-9864
247	-14236	-8351
259	-13042	-6839
260	-12942	-6713
265	-12445	-6083
267.5	-12196	-5767
270.9	-11858	-5339
275.5	-11400	-4759

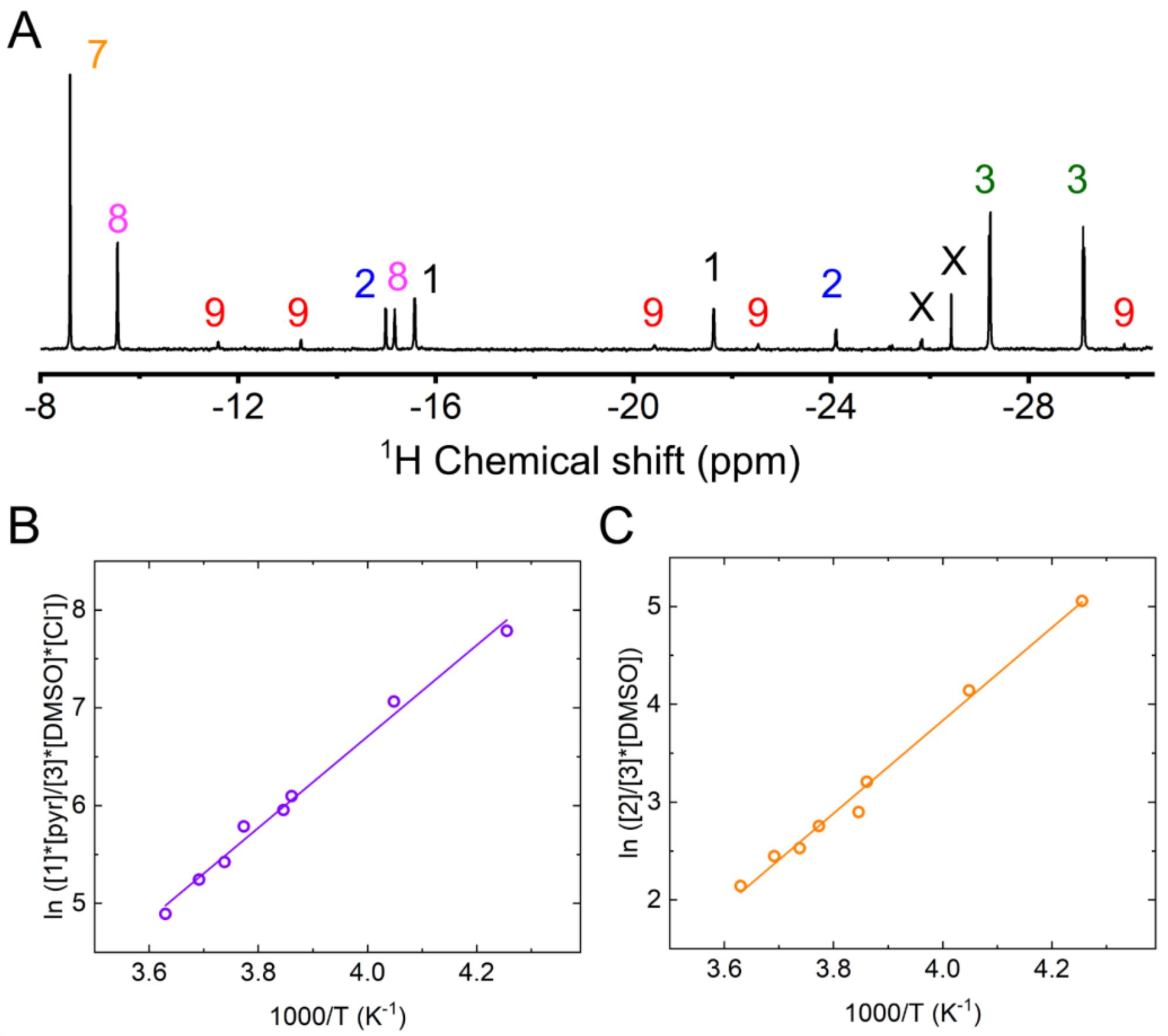

Figure S3: (A) ^{1}H NMR spectrum of the hydride region (negative chemical shifts) for the SABRE-pyruvate system showing peak assignments corresponding to iridium complexes **1-3** and **7-9**. These complexes were assigned in[4]. The peaks at -25.84 ppm and -26.47 ppm have not been structurally assigned but play no role in the observed kinetic exchange profile. (B) The Van't Hoff plot of ln([1][pyr]/[3][DMSO][Cl⁻]) versus 1000/T based on the thermodynamic equilibrium between complexes [1] and [3] (Eq. S40). The slope and intercept of the linear fit yield the enthalpy ($\Delta H^0_{1\leftarrow 3}$) and entropy ($\Delta S^0_{1\leftarrow 3}$) changes, respectively. (C) The Van't Hoff plot of ln([2]/[3][DMSO]) versus 1000/T, corresponding to the equilibrium between complexes [2] and [3] (Eq. S37). $\Delta H^0_{2\leftarrow 3}$ and $\Delta S^0_{2\leftarrow 3}$ were determined from the slope and intercept of the linear fit. Source data are provided as a Source Data file.

7. IrHH hydrogen exchange

7.1. SEPP-SABRE kinetics of IrHH hydrogens as a function of temperature

Sample: 4 mM of [Ir-d_{22}], 40 mM of ^{13}C-pyr, and 20 mM of DMSO in 600 µL of methanol-d_4.

Experiment: In all the experiments below, we used a 9.4 T NMR system, pH_2 at 8.5 bar pressure, and a SEPP-SABRE sequence for ^{1}H hyperpolarization of the selected proton. The temperature varied between 260 K and 275.5 K. We performed SEPP-SABRE on 6 hydrogens H^a and H^b of all 3 complexes. Consequently, 2 sets of data were measured for each complex. Data showed an exchange of hydrogens within complexes **3** and **2**. The hyperpolarized selected proton of **3** and **2** showed no exchange with the hydrogens of **1**; selection of 1 also did not show conversion to **3** and **2**.

Simulations: To streamline the analysis, we fitted **3** and **2** together using model (**3**↔**2**)↔H_2, and **1** separately using model IrHH↔H_2. The scripts were written in MATLAB, and global fitting was used when possible.

7.1.1. Complex 3 & 2

Experiment: For each temperature, 4 sets of data were measured. Set 1 - hyperpolarization of H^a-**3** at -29.1 ppm using SEPP-SABRE and following exchanging with H^a-**3** at -27.2 ppm, H^a-**2** at -14.98 ppm, H^b-**2** at -24.08 ppm, and free H_2 at 4.6 ppm (**Figure S4-A, E**). Set 2 - hyperpolarization of H^b-**3** using SEPP-SABRE and following exchanging with H^a-**3** , H^a-**2** , H^b-**2** , and H_2 (**Figure S4-B, F**). Set 3 - hyperpolarization of H^a-**2** using SEPP-SABRE and following exchanging with H^b-**2**, H^a-**3** , H^b-**3** , and H_2 (**Figure S4-C, G**). Set 4 - hyperpolarization of H^b-**2** using SEPP-SABRE and following exchanging with H^a-**2**, H^a-**3**, H^b-**3** , and H_2 (**Figure S4-D, H**). R_{H2} was measured for different temperatures (**Table S15**).

Simulations:

To streamline the analysis, we employed a global fitting approach for model (**3**↔**2**)↔H_2, consisting of two steps of fitting, using MATLAB scripts to simultaneously fit all parameters across 4 sets of data.

Across all 4 sets and for each temperature, we share ten parameters: 2 dissociation rate constants $k_{3-\mathrm{H}_2}$, and $k_{2-\mathrm{H}_2}$, two inter-complex exchange rate constants $k_{3\to2}$, $k_{2\to3}$, two exchange constants $k^{\mathrm{HH}}_{3-\mathrm{ex}}$, and $k^{\mathrm{HH}}_{2-\mathrm{ex}}$, two association rate constants ${k'_{3\mathrm{a}}}^{\mathrm{H}}$ and ${k'_{2\mathrm{a}}}^{\mathrm{H}}$, and two distinct relaxation exchanges $R_{\mathbf{3}}$, $R_{\mathbf{2}}$. 2 steps of fitting were followed to get these parameters. Step 1: kinetics of H^a-**3**, H^b-**3**, and H^a-**2**, H^b-**2** were summed together having 3 kinetics within 1 set: H^a-**3** + H^b-**3**, H^a-**2** + H^b-**2**, and H_2. Then, a global fit was applied using Eq. S16 of model (**3**↔**2**)↔H_2 (Intermolecular exchange) allows us to get, as explained in **section 1.2**, 6 out of 10 parameters $k_{3\to2}$, $k_{2\to3}$, k_{3-H_2}, $k_{2-\mathrm{H}_2}$, ${k'_{3\mathrm{a}}}^{\mathrm{H}}$ and ${k'_{2\mathrm{a}}}^{\mathrm{H}}$. Step 2: We used 4 sets of data, each set containing 5 kinetics: H^a-**3**, H^b-**3**, H^a-**2**, H^b-**2**, and H_2. Then, a global fit was applied using Eq. S15 (inter and intramolecular exchange) to find the rest of the parameters $k^{\mathrm{HH}}_{3-\mathrm{ex}}$, $k^{\mathrm{HH}}_{2-\mathrm{ex}}$, $R_{\mathbf{3}}$, and $R_{\mathbf{2}}$. The reported errors are the results of such fits using MATLAB's nonlinear regression function “nlinfit”.

The fitting script and integrals are available in Zenodo https://doi.org/10.5281/zenodo.18449765.

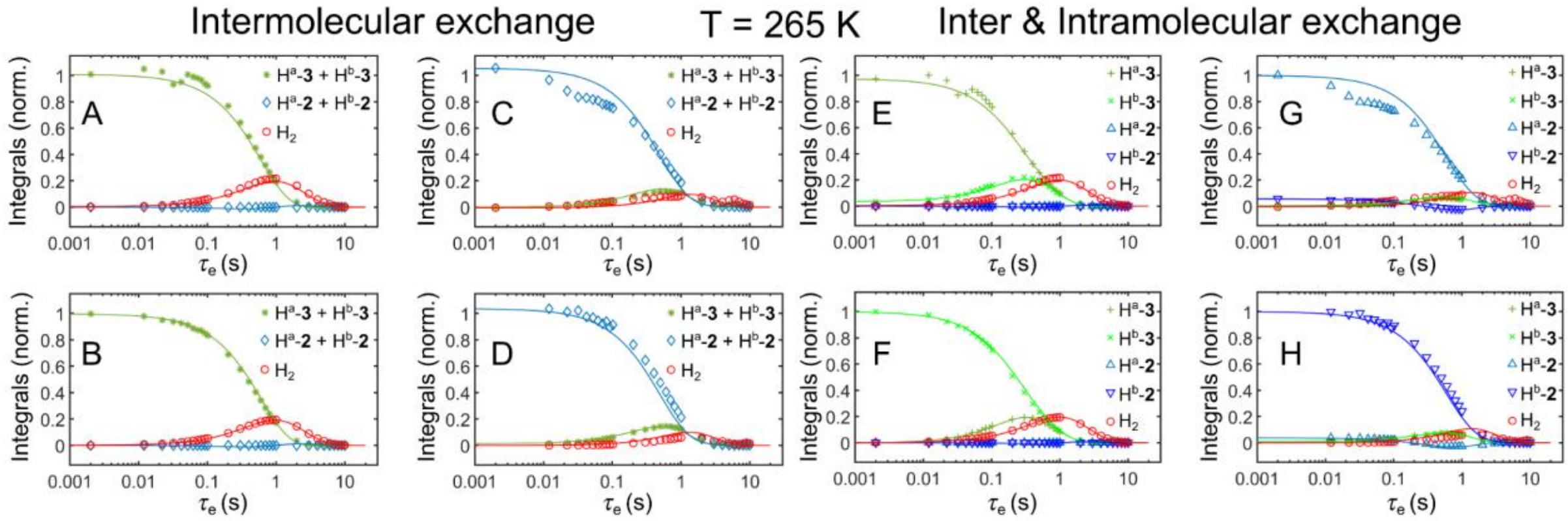

Figure S4(1): Normalized integrals of 3 and 2 SEPP-SABRE kinetics at 265 K. ^{1}H NMR integrals of the hydride at -29.2 ppm (H^a-**3**, green plus), at -27.2 ppm (H^b-**3**, green cross), and 4.6 ppm (H_2, red circle) at 265 K. The summed integrals of **3**'s hydrogens (H^a-**3** + H^b-**3**, green star), and of **2**'s hydrogens (H^a-**2** + H^b-**2**, blue diamond) are used for the intermolecular exchange. The curves are fitted using a global fit of the model (**3**↔**2**)↔H_2 (A, B, C, D, Intermolecular exchange), and (E, F, G, H, Inter and Intramolecular exchange). Estimated exchange rate constants, dissociations, associations, and relaxation-exchanges are given in **Table S4(1).**

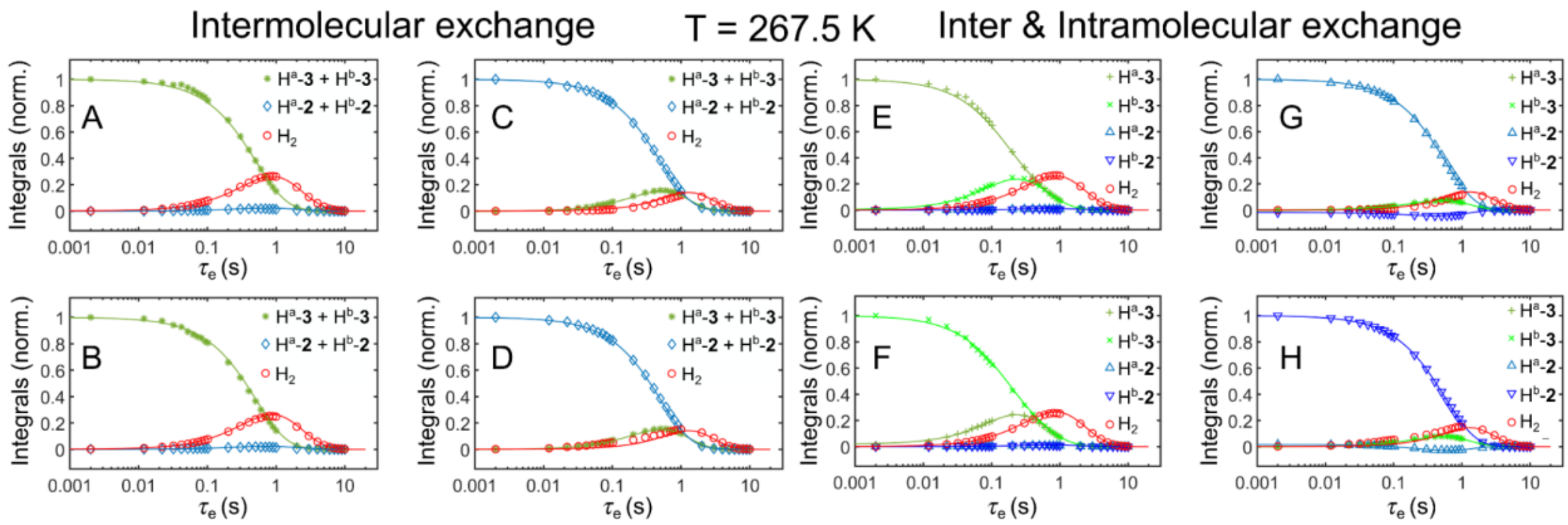

Figure S4(2): Normalized integrals of 3 and 2 SEPP-SABRE kinetics at 267.5 K. ^{1}H NMR integrals of the hydride at -29.2 ppm (H^a-**3**, green plus), at -27.2 ppm (H^b-**3**, green cross), and 4.6 ppm (H_2, red circle) at 267.5 K. The summed integrals of **3**'s hydrogens (H^a-**3** + H^b-**3**, green star), and of **2**'s hydrogens (H^a-**2** + H^b-**2**, blue diamond) are used for the intermolecular exchange. The curves are fitted using a global fit the model (**3**↔**2**)↔H_2 (A, B, C, D, Intermolecular exchange), and (E, F, G, H, Inter and Intramolecular exchange). Estimated exchange rate constants, dissociations, associations, and relaxation-exchanges are given in **Table S4(1).**

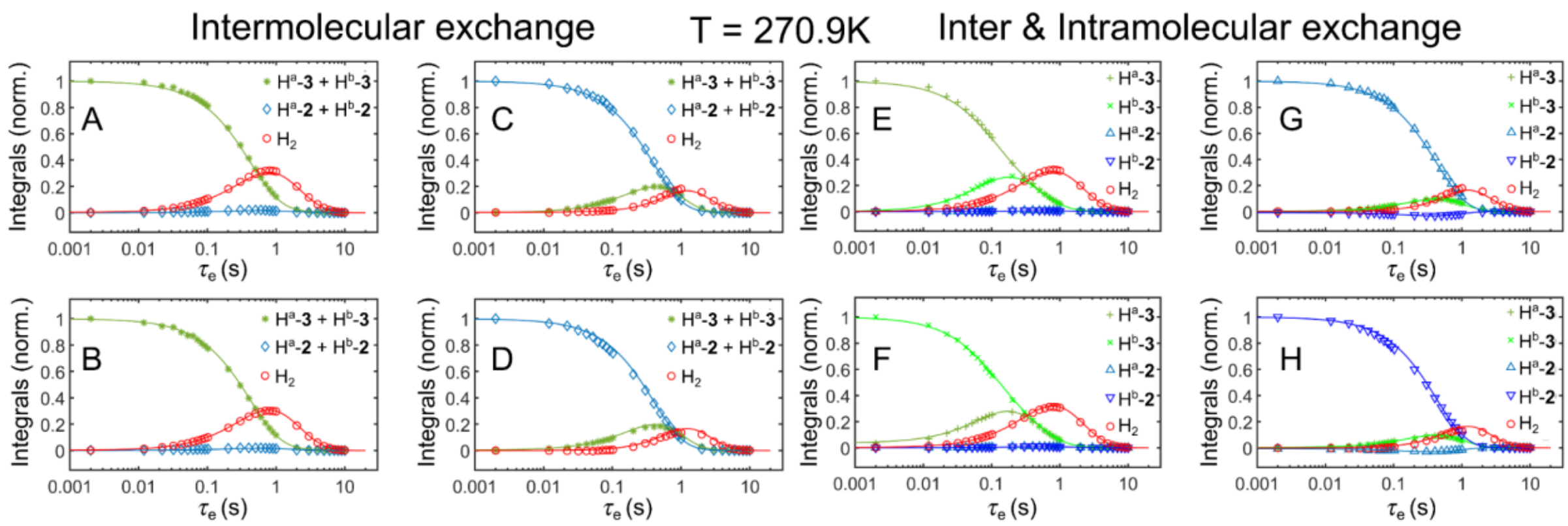

Figure S4(3): Normalized integrals of 3 and 2 SEPP-SABRE kinetics at 270.9 K. ^{1}H NMR integrals of the hydride at -29.2 ppm (H^a-**3**, green plus), at -27.2 ppm (H^b-**3**, green cross), and 4.6 ppm (H_2, red circle) at 270.9 K. The summed integrals of **3**'s hydrogens (H^a-**3** + H^b-**3**, green star), and of **2**'s hydrogens (H^a-**2** + H^b-**2**, blue diamond) are used for the intermolecular exchange. The curves are fitted using a global fit of the model (**3**↔**2**)↔H_2 (A, B, C, D, Intermolecular exchange), and (E, F, G, H, Inter and Intramolecular exchange). Estimated exchange rate constants, dissociations, associations, and relaxation-exchanges are given in **Table S4(1).**

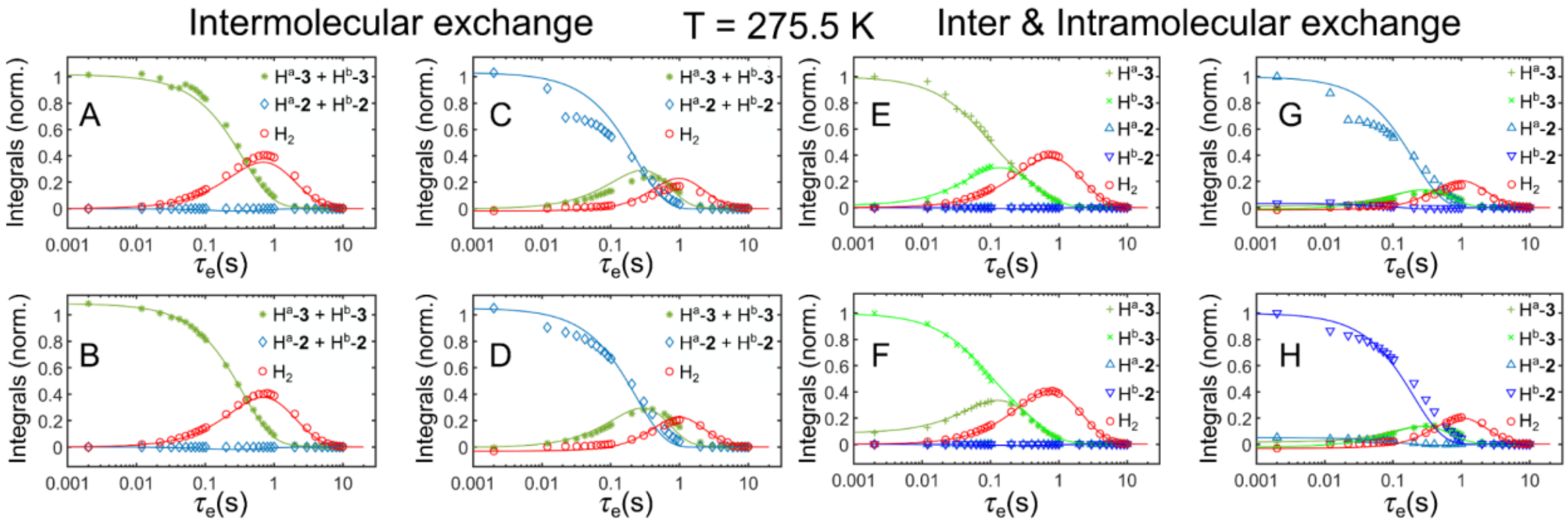

Figure S4(4): Normalized integrals of 3 and 2 SEPP-SABRE kinetics at 275.5 K. ^{1}H NMR integrals of the hydride at -29.2 ppm (H^a-**3**, green plus), at -27.2 ppm (H^b-**3**, green cross), and 4.6 ppm (H_2, red circle) at 275.5 K. The summed integrals of **3**'s hydrogens (H^a-**3** + H^b-**3**, green star), and of **2**'s hydrogens (H^a-**2** + H^b-**2**, blue diamond) are used for the intermolecular exchange. The curves are fitted using a global fit of the model (**3**↔**2**)↔H_2 (A, B, C, D, Intermolecular exchange), and (E, F, G, H, Inter and Intramolecular exchange). Estimated exchange rate constants, dissociations, associations, and relaxation-exchanges are given in **Table S4(1).**

Table S4(1). Fitting parameters of **3** and **2** SEPP-SABRE kinetics for each temperature using a 2-step fitting process of model (**3**↔**2**)↔H_2. Parameters are defined in section 2.

Temp (K)	k_{3-H_2} (s^{-1})	k_{2-H_2} (s^{-1})	$k_{3\to 2}$ (s^{-1})	$k_{2\to 3}$ (s^{-1})	k_{3-ex}^{HH} (s^{-1})	k_{2-ex}^{HH} (s^{-1})	$k'^{\,H}_{3a}$ (s^{-1})	$k'^{\,H}_{2a}$ (s^{-1})	R_3 (s^{-1})	R_2 (s^{-1})	R_{H2} (s^{-1})
**265**	0.562 ± 0.031	0.141 ± 0.024	-0.091 ± 0.041	0.665 ± 0.033	1.695 ± 0.064	-0.202 ± 0.024	-0.162 ± 0.12	0.191 ± 0.115	1.136 ± 0.031	1.054 ± 0.036	0.518 ± 0.02
**267.5**	0.868 ± 0.012	0.189 ± 0.009	0.090 ± 0.014	0.798 ± 0.012	2.928 ± 0.029	-0.182 ± 0.008	0.033 ± 0.03	0.094 ± 0.028	1.014 ± 0.010	0.948 ± 0.012	0.531 ± 0.02
**270.9**	1.148 ± 0.013	0.126 ± 0.010	0.083 ± 0.016	1.270 ± 0.015	4.401 ± 0.036	-0.191 ± 0.010	0.089 ± 0.03	0.053 ± 0.025	1.065 ± 0.010	1.131 ± 0.014	0.526 ± 0.02
**275.5**	1.484 ± 0.062	0.196 ± 0.065	-0.163 ± 0.089	2.679 ± 0.103	6.607 ± 0.204	-0.219 ± 0.09	0.117 ± 0.14	-0.021 ± 0.12	1.122 ± 0.038	2.330 ± 0.106	0.549 ± 0.02

*The negative values of k_{2-ex}^{HH} are reflective of the NOE contribution dominating under limited/zero exchange.

7.1.2. Complex 1

Sample: 4 mM of [Ir-*d*$_{22}$], 40 mM of ^{13}C-pyr, and 20 mM of DMSO in 600 µL of methanol-*d*$_4$.

Experiment: In all the experiments below, we used a 9.4 T NMR system, pH_2 at 8.5 bar pressure, and a SEPP-SABRE sequence for ^{1}H hyperpolarization of the selected proton. For each temperature, 2 sets of data were measured. Set 1 - hyperpolarization of H^a-**1** at -15.56 ppm using SEPP-SABRE and following exchanging with H^b-**1** at -21.59 ppm, and free H_2 at 4.6 ppm (**Figure S4(5)-top**). Set 2 - hyperpolarization of H^b-**1** using SEPP-SABRE and following exchanging with H^a-**1**, and H_2 (**Figure S4(5)-bottom**). R_{H2} was measured for different temperatures (**Table S15**).

Simulations:

To streamline the analysis, we employed a global fitting approach for model IrHH↔H_2 using MATLAB scripts to fit all parameters simultaneously using 2 sets of kinetics. Across both kinetics and for each temperature, we share 5 parameters: the dissociation rate constant k_{1-H_2}, exchange constant k_{1-ex}^{HH}, association rate constant $k'^{\,H}_{1a}$, natural H_2 relaxation exchange R_{H_2} and relaxation exchange R_{IrHH}. The reported errors are the results of such fits using MATLAB's nonlinear regression function "nlinfit". The fitting script, together with integrals, is available in Zenodo DOI: https://doi.org/10.5281/zenodo.18449765.

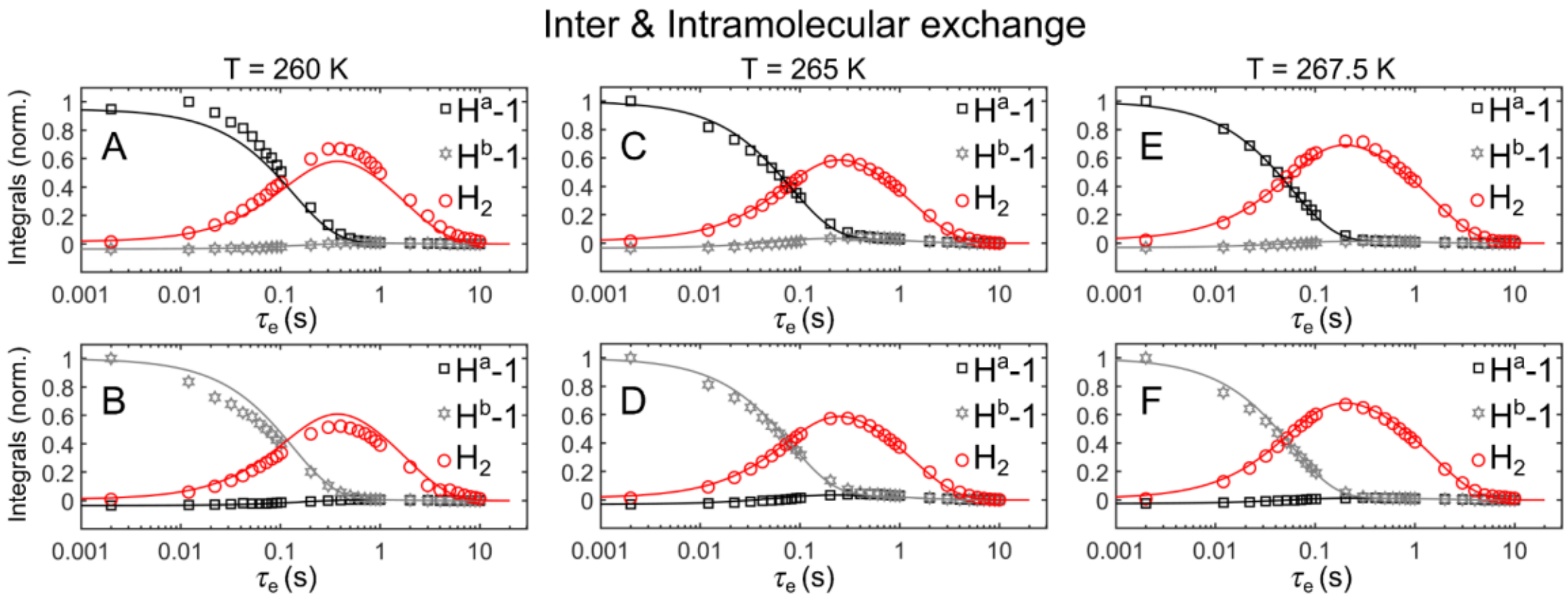

Figure S4(5): Normalized integrals of 1 SEPP-SABRE kinetics. ^{1}H NMR integrals of the hydride at -14.98 ppm (H^a-**1**, black square), at -21.59 ppm (H^b-**1**, grey star), and 4.6 ppm (H_2, red circle), at the nominal temperatures of 260 K (A, B), 265 K (C, D), and 267.5 K (E, F). The curves are fitted using the model IrHH↔H_2 (Inter and Intramolecular exchange). Estimated exchange rate constants, dissociations, associations, and relaxation-exchanges are given in **Table S4(2)**.

Table S4(2).. Fitting parameters of **1** SEPP-SABRE kinetics for each temperature using model IrHH↔H_2. Parameters are defined in section 2

**Temp (K)**	k_{1-H_2} (s^{-1})	$k_{1-\mathrm{ex}}^{\mathrm{HH}}$ (s^{-1})	$k_{1a}'^{\mathrm{H}}$ (s^{-1})	R_{IrHH} (s^{-1})	R_{H2} (s^{-1})
**260**	5.757 ± 0.226	-0.034 ± 0.225	0.153 ± 0.148	1.644 ± 0.211	0.515 ± 0.02
**265**	8.530 ± 0.098	0.779 ± 0.099	1.129 ± 0.074	2.505 ± 0.084	0.518 ± 0.02
**267.5**	13.444 ± 0.119	0.298 ± 0.125	0.652 ± 0.064	3.083 ± 0.107	0.531 ± 0.02

*The negative value here corresponds to very low or zero flux within the error bars.

7.2. SEPP-SABRE kinetics of IrHH hydrogens as a function of pressure

Sample: 4 mM of [Ir-*d*$_{22}$], 40 mM of ^{13}C-pyr, and 20 mM of DMSO in 600 µL of methanol-*d*$_4$.

Experiment: In all the experiments below, we used a 9.4 T NMR system, at 265 K, and a SEPP-SABRE sequence for ^{1}H hyperpolarization of the selected proton. For each pressure, 4 sets of data were measured. Set 1 - hyperpolarization of H^a-**3** at -29.1 ppm using SEPP-SABRE and following exchanging with H^b-**3** at -27.2 ppm, Ha-2 at -14.98 ppm, H^b-**2** at -24.08 ppm, and free H_2 at 4.6 ppm (**Figure S5-A, E**). Set 2 - hyperpolarization of H^b-**3** using SEPP-SABRE and following exchanging with H^a-**3**, H^a-**2**, H^b-**2**, and H_2 (**Figure S5-B, F**). Set 3 - hyperpolarization of H^a-**2** using SEPP-SABRE and following exchanging with H^b-**2**, H^a-**3**, H^b-**3**, and H_2 (**Figure S5-C, G**). Set 4 - hyperpolarization of H^b-**2** using SEPP-SABRE and following exchanging with H^a-**2**, H^a-**3**, H^b-**3**, and H_2 (**Figure S5-D, H**). R_{H2} was measured for different temperatures (**Table S15**).

7.2.1. Complex 3 & 2

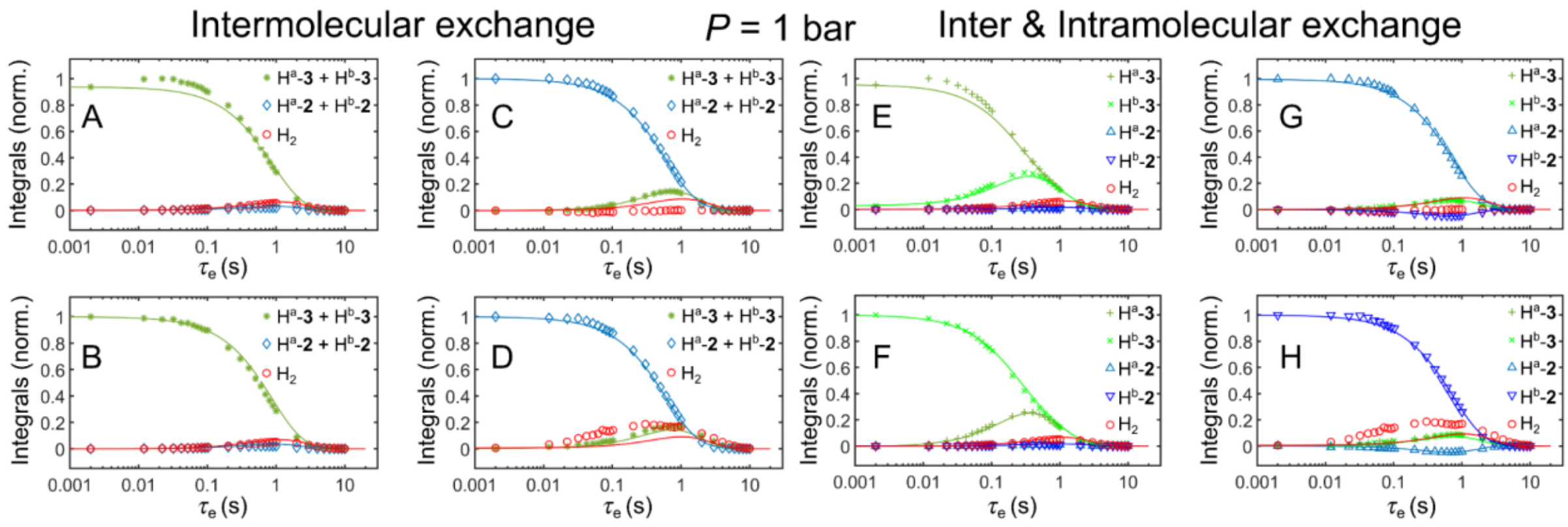

Figure S5(1): Normalized integrals of 3 and 2 SEPP-SABRE kinetics at 1 bar. ^{1}H NMR integrals of the hydride at -29.2 ppm (H^a-**3**, green plus), at -27.2 ppm (H^b-**3**, green cross), and 4.6 ppm (H_2, red circle) at 265 K. The summed integrals of **3**'s hydrogens (H^a-**3** + H^b-**3**, green star), and of **2**'s hydrogens (H^a-**2** + H^b-**2**, blue diamond) are used for the intermolecular exchange. The curves are fitted using a global fit of the model (**3**↔**2**)↔H_2 (A, B, C, D, Intermolecular exchange), and (E, F, G, H, Inter and Intramolecular exchange). Estimated exchange rate constants, dissociations, associations, and relaxation-exchanges are given in **Table S5(1).**

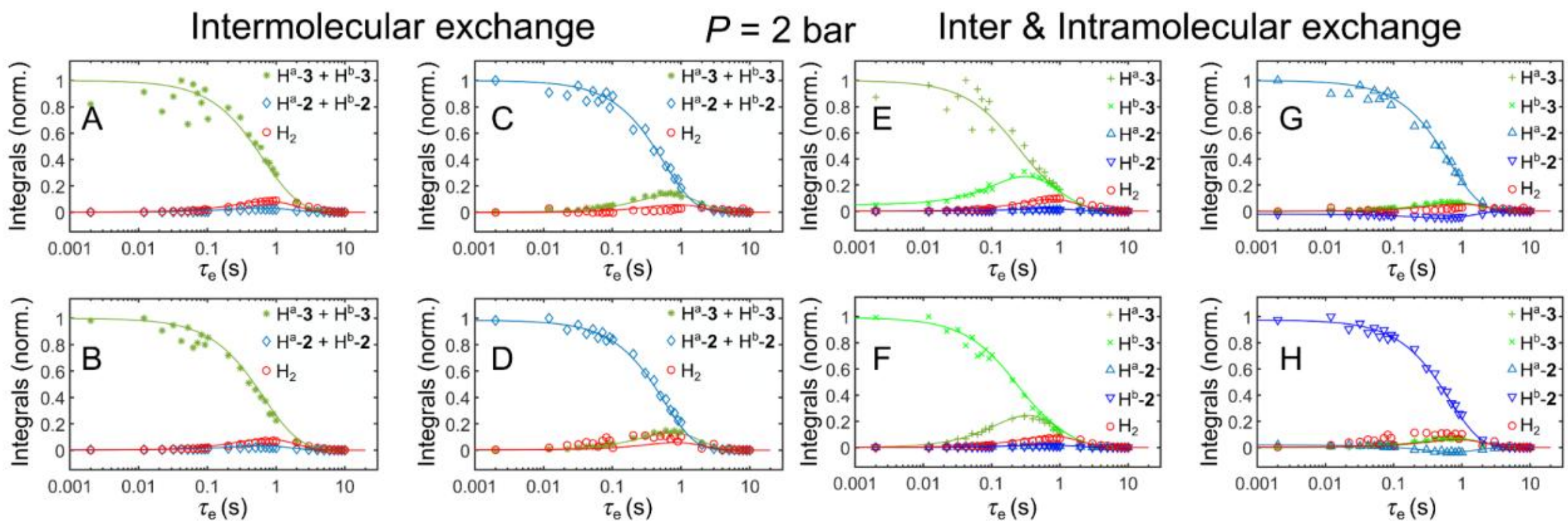

Figure S5(2): Normalized integrals of 3 and 2 SEPP-SABRE kinetics at 2 bar. ^{1}H NMR integrals of the hydride at -29.2 ppm (H^a-**3**, green plus), at -27.2 ppm (H^b-**3**, green cross), and 4.6 ppm (H_2, red circle) at 265 K. The summed integrals of **3**'s hydrogens (H^a-**3** + H^b-**3**, green star), and of **2**'s hydrogens (H^a-**2** + H^b-**2**, blue diamond) are used for the intermolecular exchange. The curves are fitted using a global fit of the model (**3**↔**2**)↔H_2 (A, B, C, D, Intermolecular exchange), and (E, F, G, H, Inter and Intramolecular exchange). Estimated exchange rate constants, dissociations, associations, and relaxation-exchanges are given in **Table S5(1).**

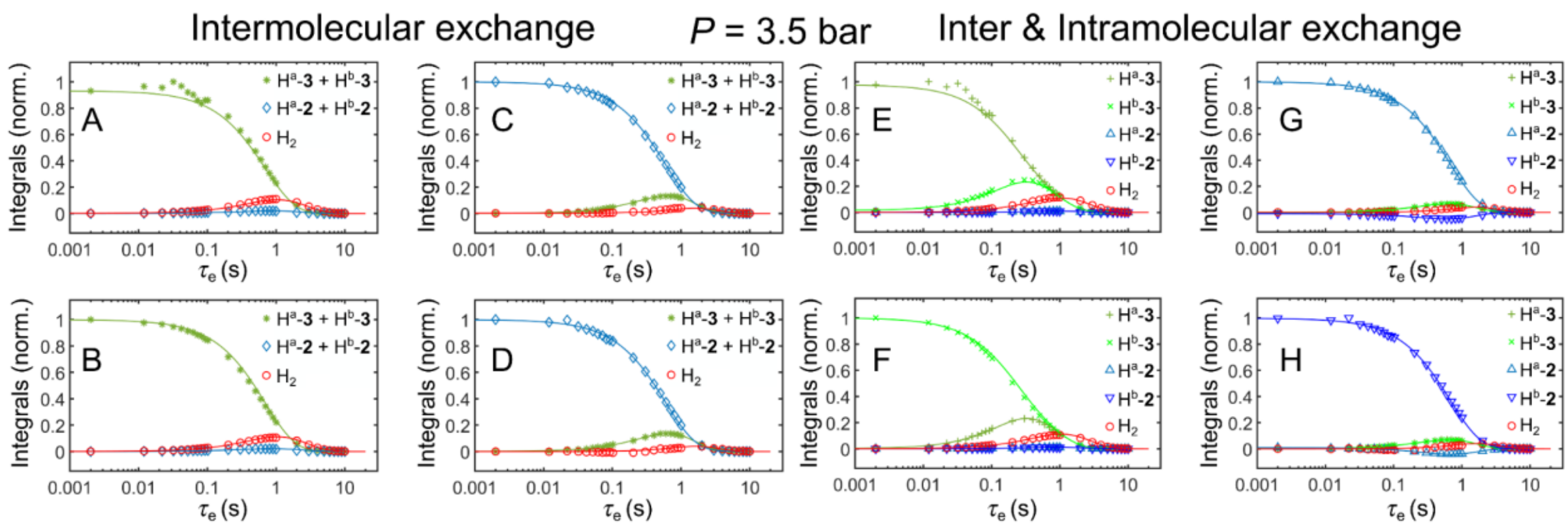

Figure S5(3): Normalized integrals of 3 and 2 SEPP-SABRE kinetics at 3.5 bar. ^{1}H NMR integrals of the hydride at -29.2 ppm (H^a-3, green plus), at -27.2 ppm (H^b-3, green cross), and 4.6 ppm (H_2, red circle) at 265 K. The summed integrals of **3**'s hydrogens (H^a-**3** + H^b-**3**, green star), and of **2**'s hydrogens (H^a-**2** + H^b-**2**, blue diamond) are used for the intermolecular exchange. The curves are fitted using a global fit of the model (**3**↔**2**)↔H_2 (A, B, C, D, Intermolecular exchange), and (E, F, G, H, Inter and Intramolecular exchange). Estimated exchange rate constants, dissociations, associations, and relaxation-exchanges are given in **Table S5(1).**

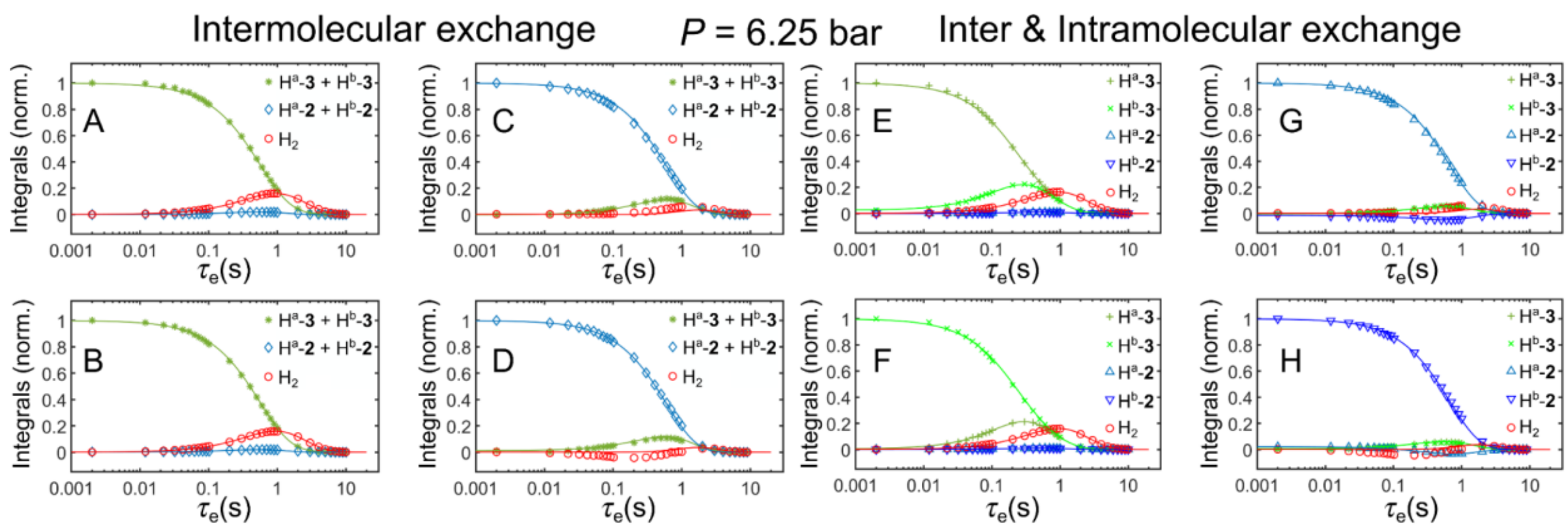

Figure S5(4): Normalized integrals of 3 and 2 SEPP-SABRE kinetics at 6.25 bar. ^{1}H NMR integrals of the hydride at -29.2 ppm (H^a-**3**, green plus), at -27.2 ppm (H^b-**3**, green cross), and 4.6 ppm (H_2, red circle) at 265 K. The summed integrals of **3**'s hydrogens (H^a-**3** + H^b-**3**, green star), and of **2**'s hydrogens (H^a-**2** + H^b-**2**, blue diamond) are used for the intermolecular exchange. The curves are fitted using a global fit of the model (**3**↔**2**)↔H_2 (A, B, C, D, Intermolecular exchange), and (E, F, G, H, Inter and Intramolecular exchange). Estimated exchange rate constants, dissociations, associations, and relaxation-exchanges are given in **Table S5(1).**

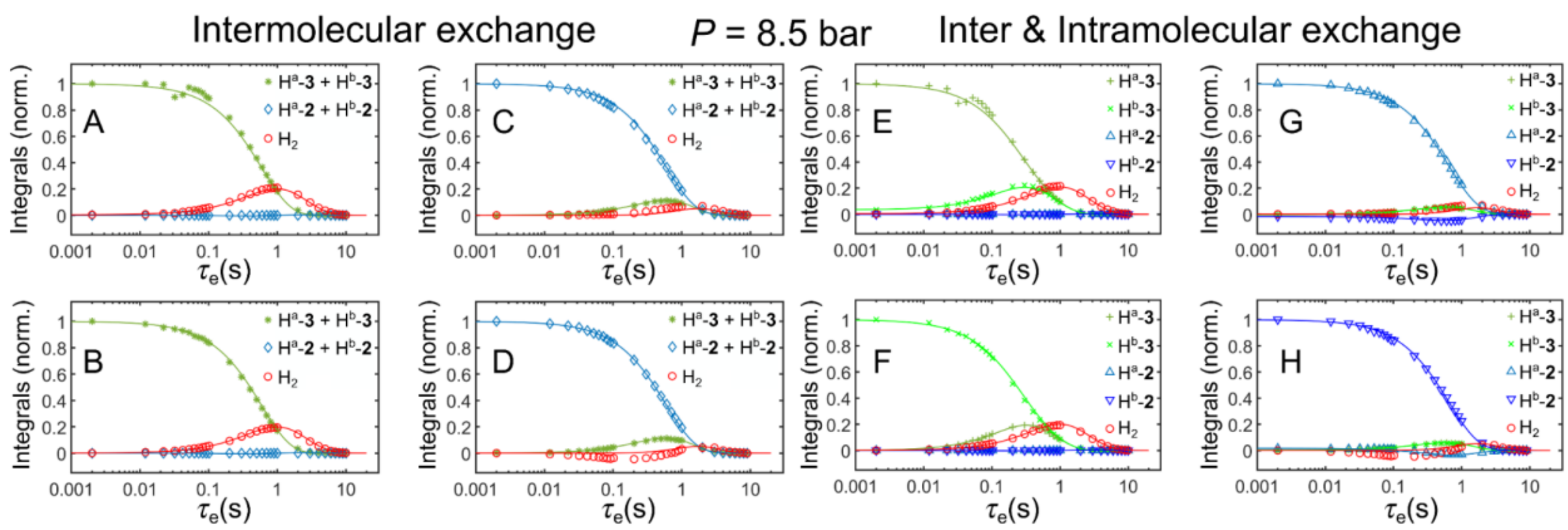

Figure S5(5): Normalized integrals of 3 and 2 SEPP-SABRE kinetics at 8.5 bar. ^{1}H NMR integrals of the hydride at -29.2 ppm (H^a-**3**, green plus), at -27.2 ppm (H^b-**3**, green cross), and 4.6 ppm (H_2, red circle) at 265 K. The summed integrals of **3**'s hydrogens (H^a-**3** + H^b-**3**, green star), and of **2**'s hydrogens (H^a-**2** + H^b-**2**, blue diamond) are used for the intermolecular exchange. The curves are fitted using a global fit of the model (**3**↔**2**)↔H_2 (A, B, C, D, Intermolecular exchange), and (E, F, G, H, Inter and Intramolecular exchange). Estimated exchange rate constants, dissociations, associations, and relaxation-exchanges are given in **Table S5(1).**

Table S5(1). Fitting parameters of **3** and **2** SEPP-SABRE kinetics for each pressure using 2 steps fitting process of model (**3**↔**2**)↔H_2. Parameters are defined in section 2.

Press (bar)	k_{3-H_2} (s^{-1})	k_{2-H_2} (s^{-1})	$k_{3\to 2}$ (s^{-1})	$k_{2\to 3}$ (s^{-1})	$k_{3-\mathrm{ex}}^{\mathrm{HH}}$ (s^{-1})	$k_{2-\mathrm{ex}}^{\mathrm{HH}}$ (s^{-1})	$k'_{3\mathrm{a}}{}^{\mathrm{H}}$ (s^{-1})	$k'_{2\mathrm{a}}{}^{\mathrm{H}}$ (s^{-1})	R_3 (s^{-1})	R_2 (s^{-1})	R_{H2} (s^{-1})
**1**	0.175 ± 0.026	0.234 ± 0.030	0.134 ± 0.031	0.497 ± 0.035	2.109 ± 0.067	-0.188 ± 0.020	0.218 ± 0.232	0.105 ± 0.247	0.942 ± 0.025	0.790 ± 0.030	0.518 ± 0.02
**2**	0.319 ± 0.055	0.181 ± 0.042	0.147 ± 0.053	0.509 ± 0.046	2.230 ± 0.078	-0.184 ± 0.024	0.997 ± 0.466	-0.053 ± 0.403	0.969 ± 0.028	1.000 ± 0.037	0.518 ± 0.02
**3.5**	0.317 ± 0.008	0.014 ± 0.006	0.119 ± 0.010	0.569 ± 0.008	2.088 ± 0.024	-0.191 ± 0.008	0.202 ± 0.061	-0.055 ± 0.054	1.063 ± 0.010	1.094 ± 0.012	0.518 ± 0.02
**6.25**	0.493 ± 0.087	-0.032 ± 0.060	0.095 ± 0.107	0.539 ± 0.085	2.079 ± 0.025	-0.177 ± 0.008	0.139 ± 0.448	-0.032 ± 0.363	1.161 ± 0.011	1.177 ± 0.012	0.518 ± 0.02
**8.5**	0.578 ± 0.082	-0.010 ± 0.059	-0.032 ± 0.101	0.532 ± 0.084	1.710 ± 0.029	-0.163 ± 0.010	-0.049 ± 0.333	0.060 ± 0.269	1.139 ± 0.014	1.178 ± 0.016	0.518 ± 0.02

7.2.2. Complex 1

Sample: 4 mM of [Ir-*d*$_{22}$], 40 mM of ^{13}C-pyr, and 20 mM of DMSO in 600 µL of methanol-*d*$_4$.

Experiment: In all the experiments below, we used a 9.4 T NMR system, at 265 K, and a SEPP-SABRE sequence for ^{1}H hyperpolarization of the selected proton. For each pressure, 2 sets of data were measured. Set 1 - hyperpolarization of H^a-**1** at -15.56 ppm using SEPP-SABRE and following exchanging with H^b-**1** at -21.59 ppm, and free H_2 at 4.6 ppm (**Figure S5(6)-top**). Set 2 - hyperpolarization of H^b-**1** using SEPP-SABRE and following exchange with H^a-**1**, and H_2 (**Figure S5(6)-bottom**). R_{H2} was measured for different temperatures (**Table S15**).

Simulations:

To streamline the analysis, we employed a global fitting approach for model IrHH↔H_2 using MATLAB scripts to fit all parameters simultaneously using 2 sets of kinetics. Across both kinetics and for each temperature, we share 5 parameters: the dissociation rate constant $k_{1-\mathrm{H}_2}$, exchange constant $k_{1-\mathrm{ex}}^{\mathrm{HH}}$, association rate constant $k'_{1\mathrm{a}}{}^{\mathrm{H}}$, natural H_2 relaxation exchange R_{H_2} and relaxation exchange $R_{\mathbf{IrHH}}$. The reported errors are the results of such fits using MATLAB's nonlinear regression function "nlinfit". The fitting script, together with integrals, is available in Zenodo DOI: https://doi.org/10.5281/zenodo.18449765.

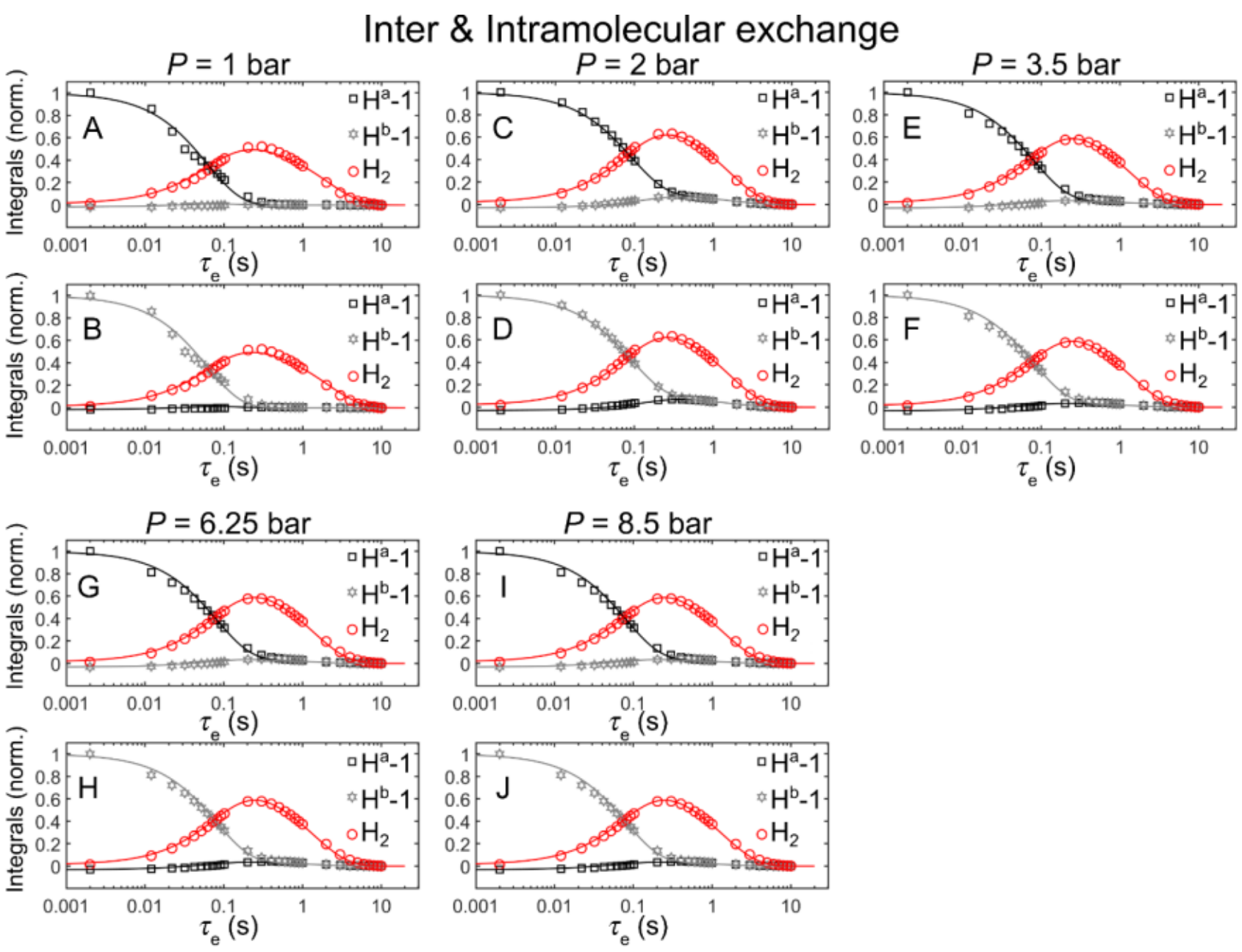

Figure S5(6): Normalized integrals of SEPP-SABRE kinetics. ^{1}H NMR integrals of the hydride at -14.98 ppm (H^a-**1**, black square), at -21.59 ppm (H^b-**1**, grey star), and 4.6 ppm (H_2, red circle), at the pressure of 1 bar (A, B),

2 bar (C, D), 3.5 bar (E, F), 6.25 bar (G, H), and 8.5 bar (I, J). The curves are fitted using the model IrHH↔H_2 (Inter and Intramolecular exchange). Estimated exchange rate constants, dissociations, associations, and relaxation-exchanges are given in **Table S5(2)**.

Table S5(2). Fitting parameters of **1** SEPP-SABRE kinetics for each pressure using model IrHH↔H_2. Parameters are defined in section 2.

**Back pressure Bubbling pressure**	k_{1-H_2} (s^{-1})	$k_{1-\mathrm{ex}}^{\mathrm{HH}}$ (s^{-1})	${k'_{1\mathrm{a}}}^{\mathrm{H}}$ (s^{-1})	R_{IrHH} (s^{-1})	R_{H2} (s^{-1})
**5 PSI 1 bar**	8.578 ± 0.147	0.680 ± 0.218	-0.066 ± 0.041	7.089 ± 0.202	0.518 ± 0.02
**20 PSI 2 bar**	8.634 ± 0.053	0.378 ± 0.044	2.198 ± 0.045	0.979 ± 0.021	0.518 ± 0.02
**40 PSI 3.5 bar**	8.579 ± 0.101	0.790 ± 0.102	1.119 ± 0.077	2.518 ± 0.087	0.518 ± 0.02
**75 PSI 6.25 bar**	9.216 ± 0.090	0.347 ± 0.103	0.305 ± 0.047	3.217 ± 0.094	0.518 ± 0.02
**100 PSI 8.5 bar**	9.317 ± 0.096	0.353 ± 0.104	0.339 ± 0.052	2.848 ± 0.096	0.518 ± 0.02

7.3. SEPP-SABRE kinetics of IrHH hydrogens as a function of [DMSO]

Sample: 4 mM of [Ir-d_{22}], 40 mM of ^{13}C-pyr, and a variable amount of DMSO between 20 and 300 mM in 600 µL of methanol-d_4.

Experiment: In all the experiments below, we used a 9.4 T NMR system, pH_2 at 8.5 bar pressure, 265 K, and a SEPP-SABRE sequence for ^{1}H hyperpolarization of the selected proton. For each concentration, 4 sets of data were measured. Set 1 - hyperpolarization of H^a-**3** at -29.1 ppm using SEPP-SABRE and following exchanging with H^b-**3** at -27.2 ppm, H^a-**2** at -14.98 ppm, H^b-**2** at -24.08 ppm, and free H_2 at 4.6 ppm (**Figure S6-A, E**). Set 2 - hyperpolarization of H^b-**3** using SEPP-SABRE and following exchanging with H^a-**3**, H^a-**2**, H^b-**2**, and H_2 (**Figure S6-B, F**). Set 3 - hyperpolarization of H^a-**2** using SEPP-SABRE and following exchanging with H^b-**2**, H^a-**3**, H^b-**3**, and H_2 (**Figure S6-C, G**). Set 4 - hyperpolarization of H^b-2 using SEPP-SABRE and following exchanging with H^a-**2**, H^a-**3**, H^b-**3**, and H_2 (**Figure S6-D, H**). R_{H2} was measured for different temperatures (**Table S15**).

7.3.1. Complex 3 & 2

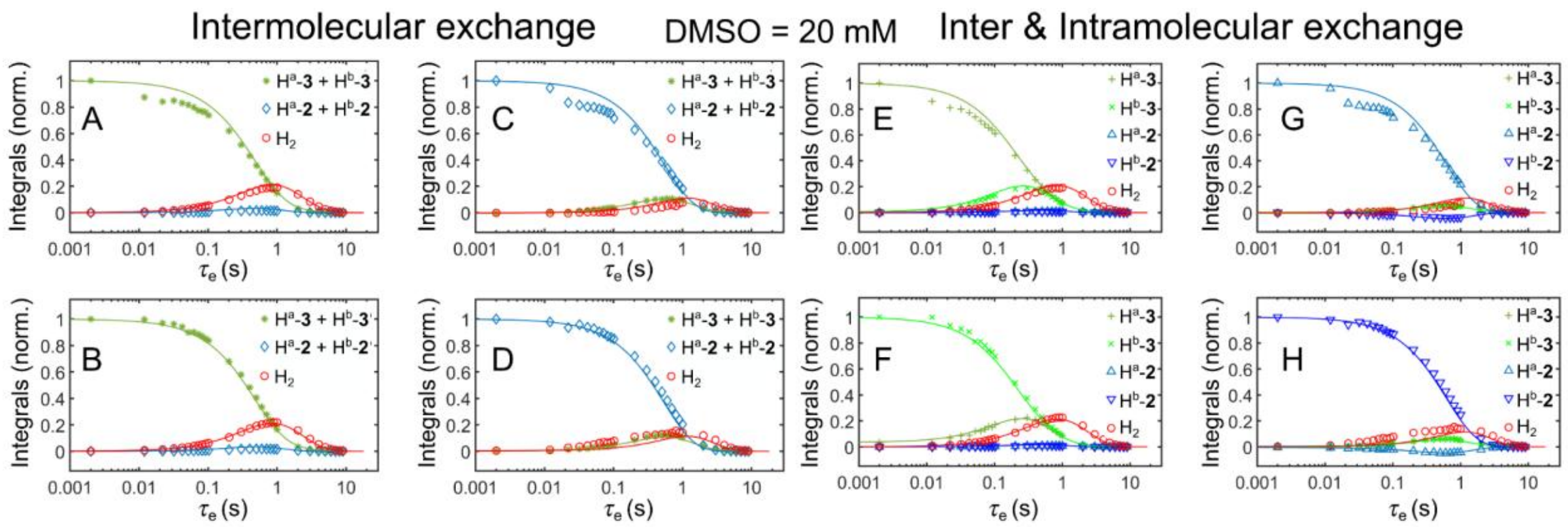

Figure S6(1): Normalized integrals of 3 and 2 SEPP-SABRE kinetics for 20 mM. ^{1}H NMR integrals of the hydride at -29.2 ppm (Ha-**3**, green plus), at -27.2 ppm (H^b-**3**, green cross), and 4.6 ppm (H_2, red circle) at 265 K. The summed integrals of **3**'s hydrogens (H^a-**3** + H^b-**3**, green star), and of **2**'s hydrogens (H^a-**2** + H^b-**2**, blue diamond) are used for the intermolecular exchange. The curves are fitted using a global fit of the model (**3**↔**2**)↔H_2 (A, B, C, D, Intermolecular exchange), and (E, F, G, H, Inter and Intramolecular exchange). Estimated exchange rate constants, dissociations, associations, and relaxation-exchanges are given in **Table S6(1).**

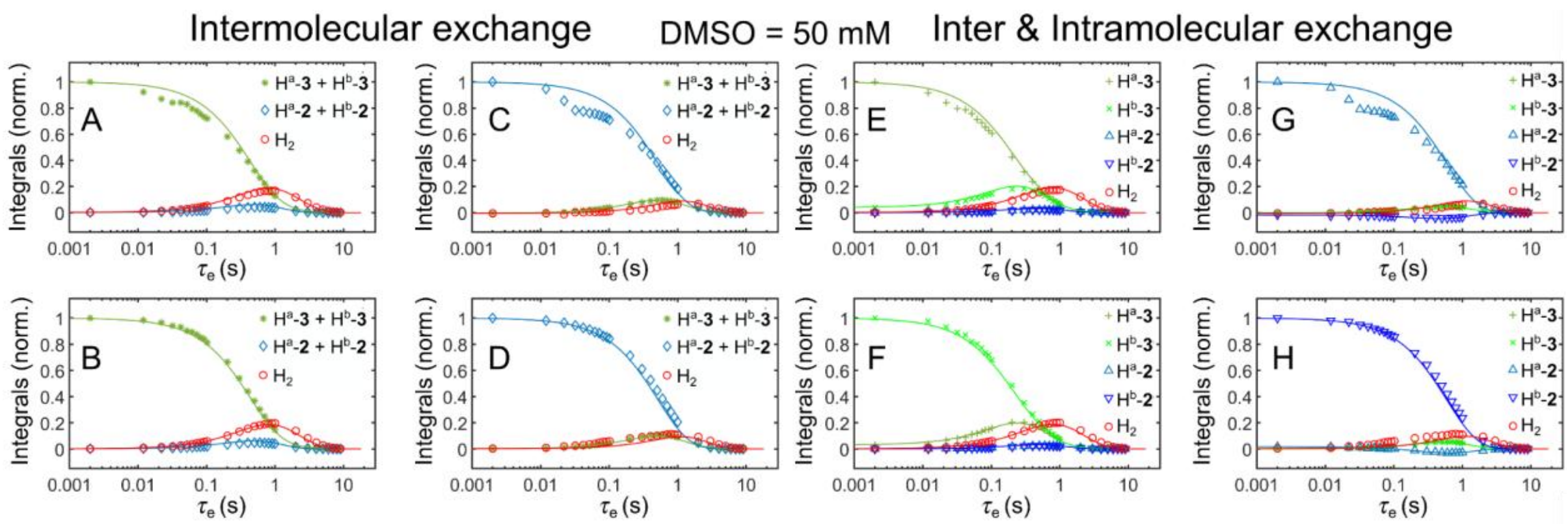

Figure S6(2): Normalized integrals of 3 and 2 SEPP-SABRE kinetics for 50 mM. ^{1}H NMR integrals of the hydride at -29.2 ppm (H^a-**3**, green plus), at -27.2 ppm (H^b-**3**, green cross), and 4.6 ppm (H_2, red circle) at 265 K. The summed integrals of **3**'s hydrogens (H^a-**3** + H^b-**3**, green star), and of **2**'s hydrogens (H^a-**2** + H^b-**2**, blue diamond) are used for the intermolecular exchange. The curves are fitted using a global fit of the model (**3**↔**2**)↔H_2 (A, B, C, D, Intermolecular exchange), and (E, F, G, H, Inter and Intramolecular exchange). Estimated exchange rate constants, dissociations, associations, and relaxation-exchanges are given in **Table S6(1).**

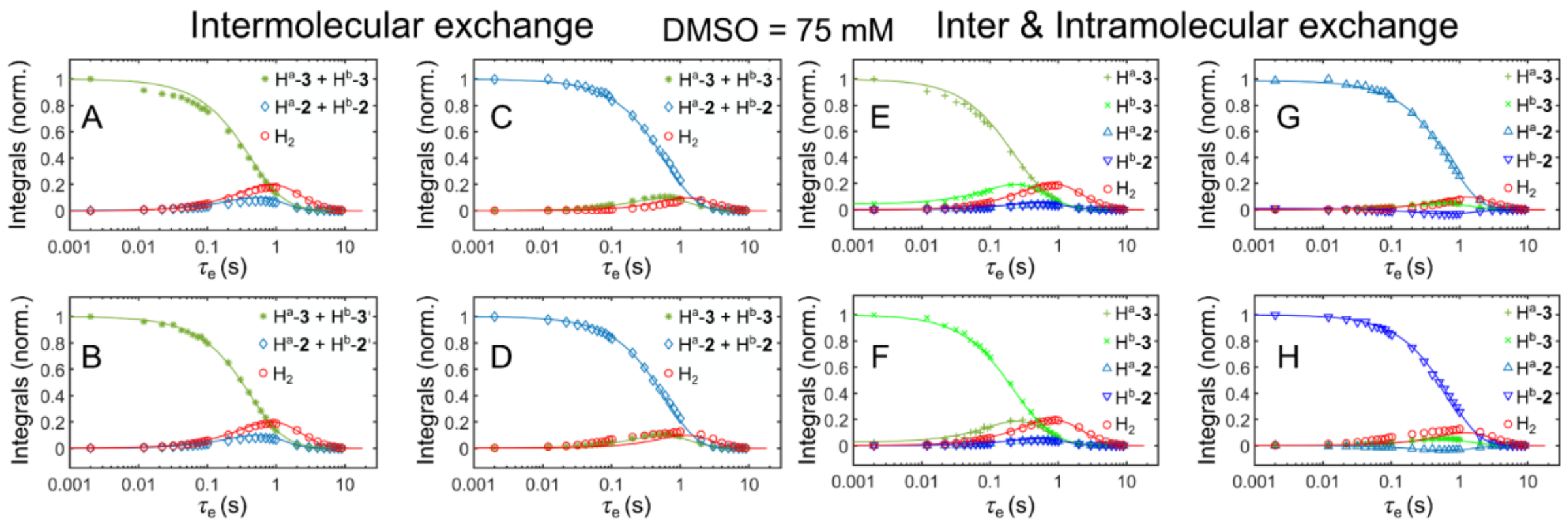

Figure S6(3): Normalized integrals of 3 and 2 SEPP-SABRE kinetics for 75 mM. ^{1}H NMR integrals of the hydride at -29.2 ppm (H^a-**3**, green plus), at -27.2 ppm (H^b-**3**, green cross), and 4.6 ppm (H_2, red circle) at 265 K. The summed integrals of **3**'s hydrogens (H^a-**3** + H^b-**3**, green star), and of **2**'s hydrogens (H^a-**2** + H^b-**2**, blue diamond) are used for the intermolecular exchange. The curves are fitted using a global fit of the model (**3**↔**2**)↔H_2 (A, B, C, D, Intermolecular exchange), and (E, F, G, H, Inter and Intramolecular exchange). Estimated exchange rate constants, dissociations, associations, and relaxation-exchanges are given in **Table S6(1).**

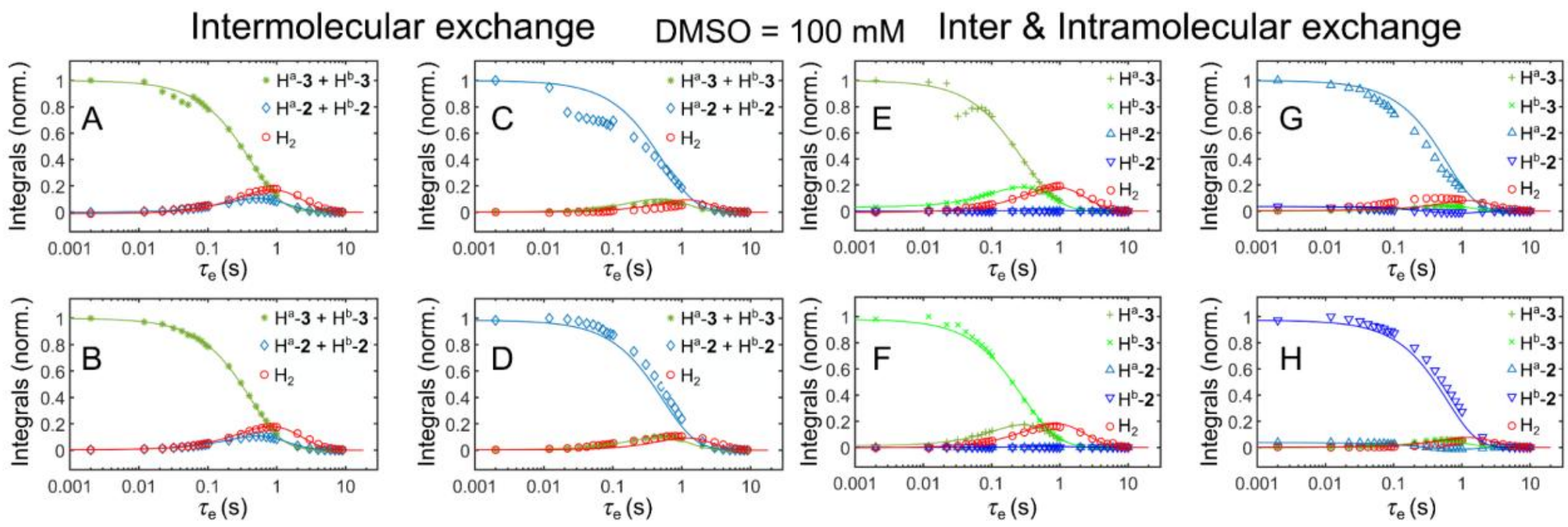

Figure S6(4): Normalized integrals of 3 and 2 SEPP-SABRE kinetics for 100 mM. ^{1}H NMR integrals of the hydride at -29.2 ppm (H^a-**3**, green plus), at -27.2 ppm (H^b-**3**, green cross), and 4.6 ppm (H_2, red circle) at 265 K. The summed integrals of **3**'s hydrogens (H^a-**3** + H^b-**3**, green star), and of **2**'s hydrogens (H^a-**2** + H^b-**2**, blue diamond) are used for the intermolecular exchange. The curves are fitted using a global fit of the model (**3**↔**2**)↔H_2 (A, B, C, D, Intermolecular exchange), and (E, F, G, H, Inter and Intramolecular exchange). Estimated exchange rate constants, dissociations, associations, and relaxation-exchanges are given in **Table S6(1).**

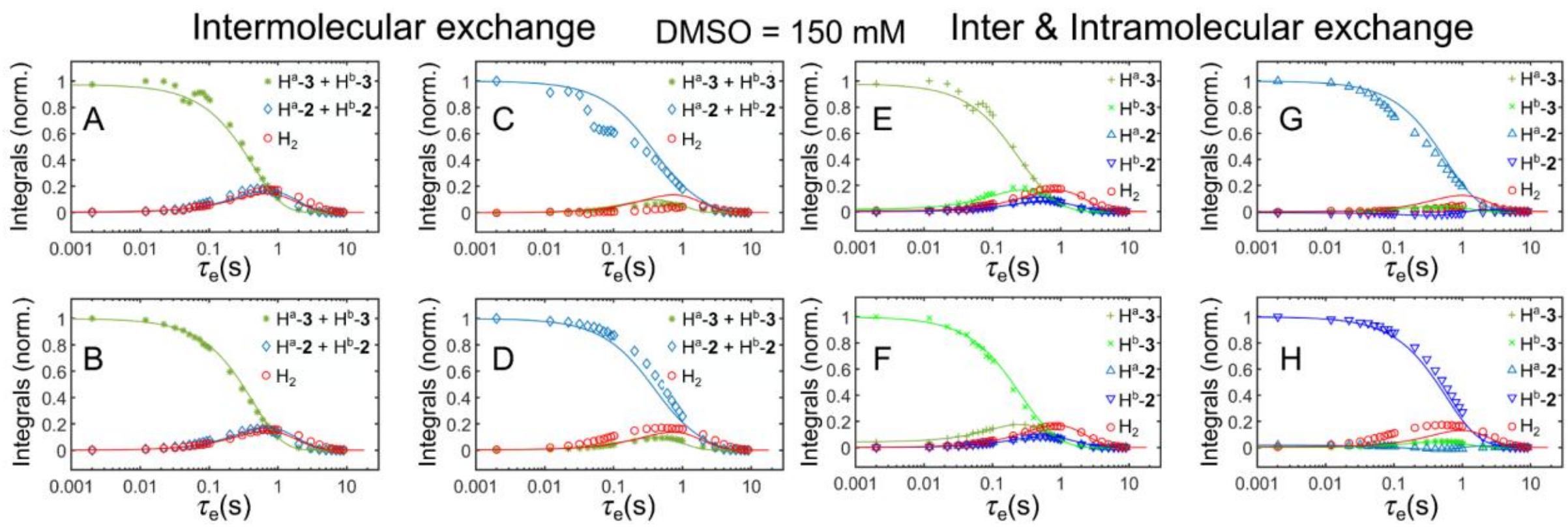

Figure S6(5): Normalized integrals of 3 and 2 SEPP-SABRE kinetics for 150 mM. ^{1}H NMR integrals of the hydride at -29.2 ppm (H^a-**3**, green plus), at -27.2 ppm (H^b-**3**, green cross), and 4.6 ppm (H_2, red circle) at 265 K. The summed integrals of **3**'s hydrogens (H^a-**3** + H^b-**3**, green star), and of **2**'s hydrogens (H^a-**2** + H^b-**2**, blue diamond) are used for the intermolecular exchange. The curves are fitted using a global fit of the model (**3**↔**2**)↔H_2 (A, B, C, D, Intermolecular exchange), and (E, F, G, H, Inter and Intramolecular exchange). Estimated exchange rate constants, dissociations, associations, and relaxation-exchanges are given in **Table S6(1).**

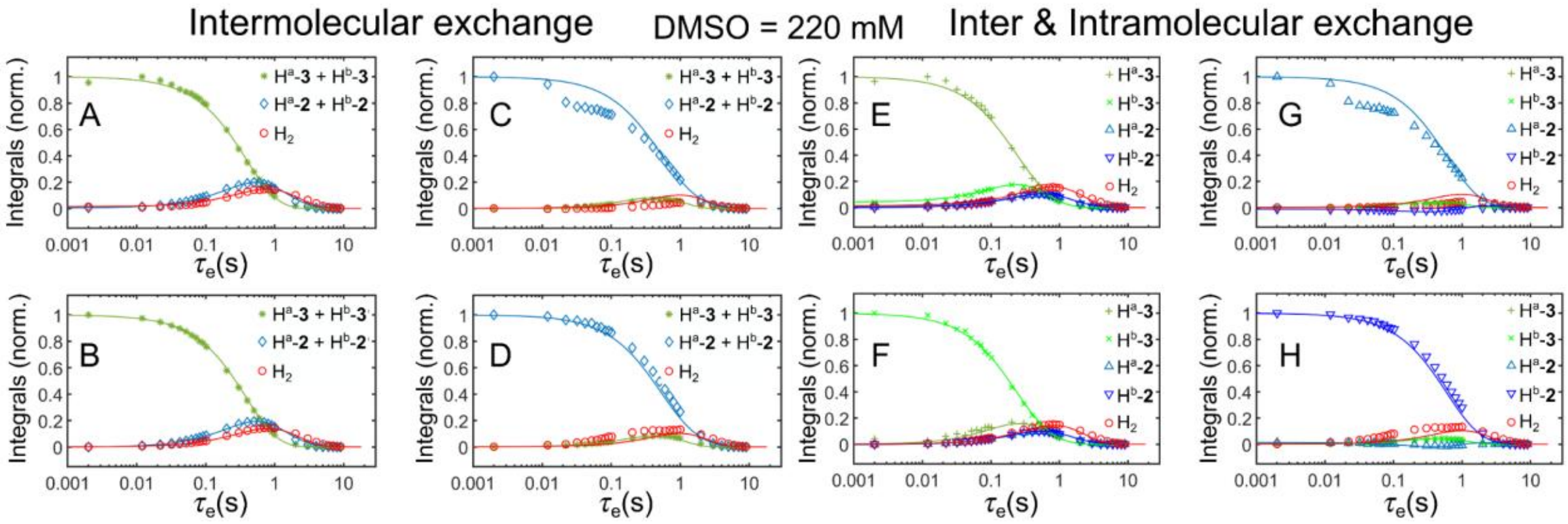

Figure S6(6): Normalized integrals of 3 and 2 SEPP-SABRE kinetics for 220 mM. ^{1}H NMR integrals of the hydride at -29.2 ppm (H^a-**3**, green plus), at -27.2 ppm (H^b-**3**, green cross), and 4.6 ppm (H_2, red circle) at 265 K. The summed integrals of **3**'s hydrogens (H^a-**3** + H^b-**3**, green star), and of **2**'s hydrogens (H^a-**2** + H^b-**2**, blue diamond) are used for the intermolecular exchange. The curves are fitted using a global fit of the model (**3**↔**2**)↔H_2 (A, B, C, D, Intermolecular exchange), and (E, F, G, H, Inter and Intramolecular exchange). Estimated exchange rate constants, dissociations, associations, and relaxation-exchanges are given in **Table S6(1).**

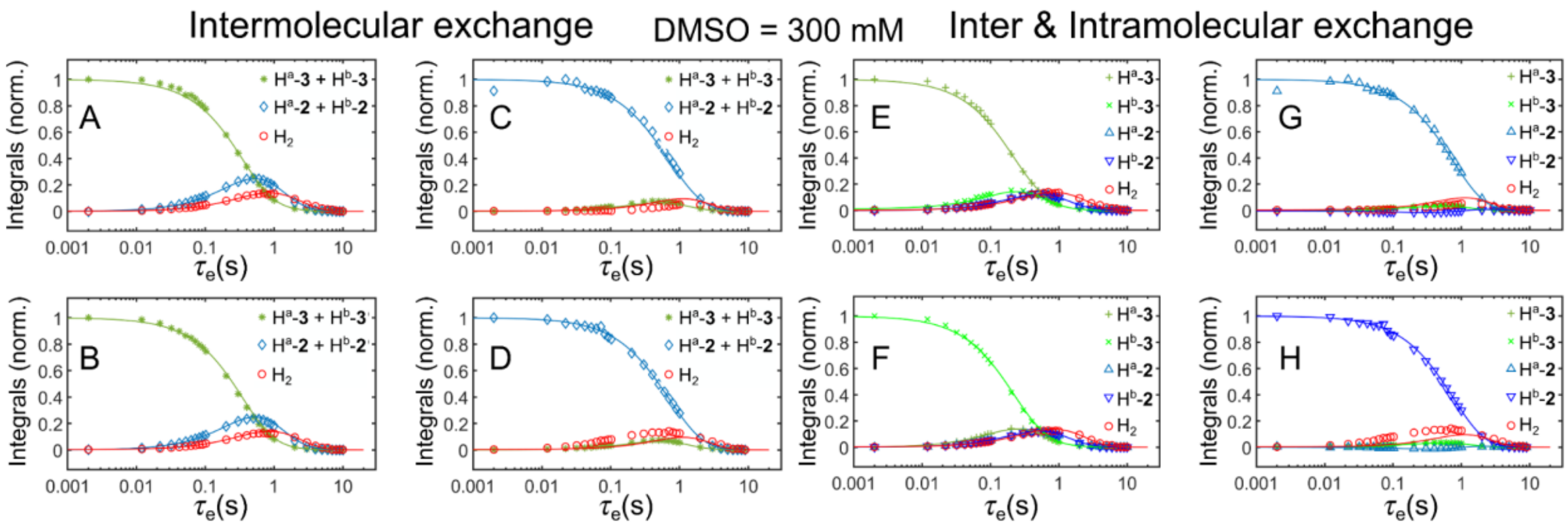

Figure S6(7): Normalized integrals of 3 and 2 SEPP-SABRE kinetics for 300 mM. ^{1}H NMR integrals of the hydride at -29.2 ppm (H^a-**3**, green plus), at -27.2 ppm (H^b-**3**, green cross), and 4.6 ppm (H_2, red circle) at 265 K. The summed integrals of **3**'s hydrogens (H^a-**3** + H^b-**3**, green star), and of **2**'s hydrogens (H^a-**2** + H^b-**2**, blue diamond) are used for the intermolecular exchange. The curves are fitted using a global fit of the model (**3**↔**2**)↔H_2 (A, B, C, D, Intermolecular exchange), and (E, F, G, H, Inter and Intramolecular exchange). Estimated exchange rate constants, dissociations, associations, and relaxation-exchanges are given in **Table S6(1).**

Table S6(1). Fitting parameters of **3** and **2** SEPP-SABRE kinetics for each concentration using a 2-step fitting process of model (**3**↔**2**)↔H_2. Parameters are defined in section 2.

[DMSO] (mM)	k_{3-H} (s^{-1})	k_{2-H_2} (s^{-1})	$k_{3\to2}$ (s^{-1})	$k_{2\to3}$ (s^{-1})	k^{HH}_{3-ex} (s^{-1})	k^{HH}_{2-ex} (s^{-1})	k'^{H}_{3a} (s^{-1})	k'^{H}_{2a} (s^{-1})	R_3 (s^{-1})	R_2 (s^{-1})	R_{H2} (s^{-1})
**20**	0.726 ± 0.04	0.220 ± 0.028	0.185 ± 0.047	0.540 ± 0.039	2.248 ± 0.080	-0.209 ± 0.023	-0.162 ± 0.12	-0.046 ± 0.114	1.168 ± 0.036	1.056 ± 0.036	0.518 ± 0.02
**50**	0.678 ± 0.04	0.186 ± 0.029	0.344 ± 0.051	0.527 ± 0.041	2.115 ± 0.081	-0.189 ± 0.024	0.248 ± 0.163	-0.023 ± 0.137	1.287 ± 0.040	1.161 ± 0.037	0.518 ± 0.02
**75**	0.701 ± 0.02	0.167 ± 0.014	0.525 ± 0.026	0.463 ± 0.021	1.949 ± 0.039	-0.170 ± 0.010	0.280 ± 0.081	-0.077 ± 0.065	1.154 ± 0.020	0.951 ± 0.015	0.518 ± 0.02
**100**	0.634 ± 0.05	0.172 ± 0.036	0.552 ± 0.066	0.559 ± 0.054	1.837 ± 0.103	-0.203 ± 0.033	0.021 ± 0.15	0.190 ± 0.190	1.103 ± 0.055	1.153 ± 0.050	0.518 ± 0.02
**150**	0.540 ± 0.04	0.345 ± 0.034	0.799 ± 0.056	0.409 ± 0.047	1.631 ± 0.077	-0.178 ± 0.025	-0.298 ± 0.181	0.276 ± 0.106	0.938 ± 0.045	1.044 ± 0.036	0.518 ± 0.02
**220**	0.513 ± 0.05	0.262 ± 0.034	1.025 ± 0.062	0.534 ± 0.049	1.738 ± 0.085	-0.192 ± 0.025	-0.433 ± 0.224	0.758 ± 0.219	0.963 ± 0.051	1.067 ± 0.037	0.518 ± 0.02
**300**	0.498 ± 0.02	0.213 ± 0.016	1.350 ± 0.031	0.362 ± 0.025	1.586 ± 0.043	-0.103 ± 0.011	0.039 ± 0.127	0.211 ± 0.112	1.007 ± 0.029	0.894 ± 0.015	0.518 ± 0.02

*The negative values of k^{HH}_{2-ex} are reflective of the NOE contribution dominating under limited/zero exchange. Other negative values correspond to very low or zero flux within the error bars.

7.3.2. Complex 1

Sample: 4 mM of [Ir-d_{22}], 40 mM of ^{13}C-pyr, and a variable amount of DMSO between 20 and 300 mM in 600 µL of methanol-d_4.

Experiment: In all the experiments below, we used a 9.4 T NMR system, at 265 K, and a SEPP-SABRE sequence for ^{1}H hyperpolarization of the selected proton. For each pressure, 2 sets of data were measured. Set 1 - Hyperpolarization of H^a-**1** at -15.56 ppm using SEPP-SABRE, followed by exchange with H^b-**1** at -21.59 ppm, and free H_2 at 4.6 ppm (**Figure S6(8)-top**). Set 2 - Hyperpolarization of H^b-**1** using SEPP-SABRE and subsequent exchange with H^a-**1** and H_2 (**Figure S6(8)-Bottom**). R_{H2} was measured for different temperatures (**Table S15**).

Simulations: To streamline the analysis, we employed a global fitting approach for model IrHH↔H_2 using MATLAB scripts to fit all parameters simultaneously using 2 sets of kinetics. Across both kinetics and for each temperature, we share 5 parameters: the dissociation rate constant $k_{1-\mathrm{H_2}}$, exchange constant $k_{1-\mathrm{ex}}^{\mathrm{HH}}$, association rate constant ${k'_{1a}}^{\mathrm{H}}$, natural H_2 relaxation exchange $R_{\mathrm{H_2}}$ and relaxation exchange R_{IrHH}. The reported errors are the results of such fits using MATLAB's nonlinear regression function "nlinfit". The fitting script, together with integrals, are available in Zenodo DOI: https://doi.org/10.5281/zenodo.18449765.

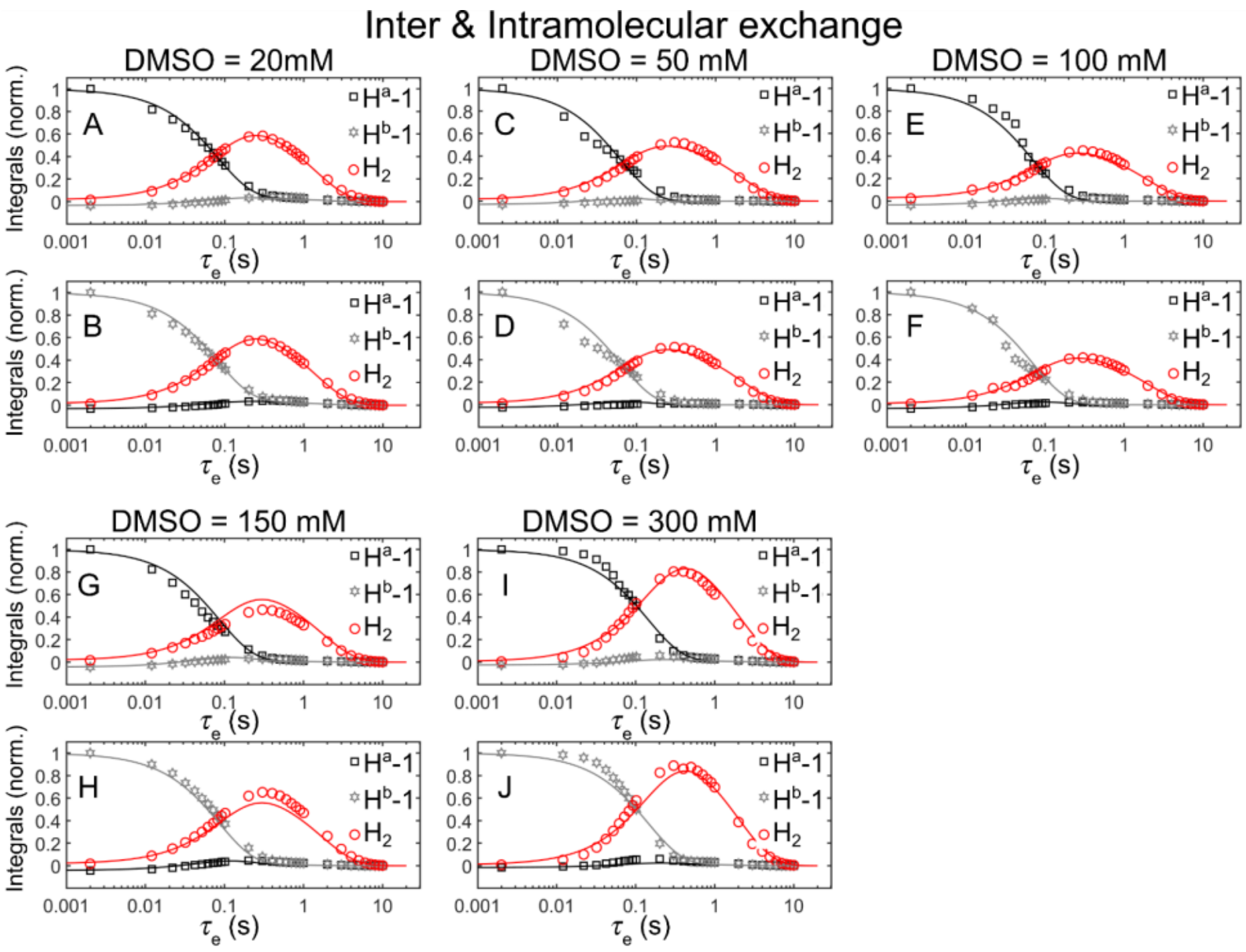

Figure S6(8): Normalized integrals of SEPP-SABRE kinetics. ^{1}H NMR integrals of the hydride at -14.98 ppm (H^a-**1**, black square), at -21.59 ppm (H^b-**1**, grey star), and 4.6 ppm (H_2, red circle), of 20 mM (A, B), 50 mM (C, D), 100 mM (E, F), 150 mM (G, H), and 300 mM (I, J). The curves are fitted using the model IrHH↔H_2 (Inter and Intramolecular exchange). Estimated exchange rate constants, dissociations, associations, and relaxation-exchanges are given in **Table S6(2).**

Table S6(2). Fitting parameters of **1** SEPP-SABRE kinetics for each DMSO concentration using model IrHH↔H_2. Parameters are defined in section 2

**[DMSO] (mM)**	k_{1-H_2} (s^{-1})	$k_{1-\mathrm{ex}}^{\mathrm{HH}}$ (s^{-1})	${k'_{1a}}^{\mathrm{H}}$ (s^{-1})	R_{IrHH} (s^{-1})	R_{H2} (s^{-1})
**20**	8.530 ± 0.098	0.779 ± 0.099	1.129 ± 0.074	2.505 ± 0.084	0.518 ± 0.02

**50**	7.982 ± 0.201	1.431 ± 0.312	-0.129 ± 0.055	6.580 ± 0.273	0.518 ± 0.02
**100**	6.193 ± 0.174	1.354 ± 0.279	-0.053 ± 0.060	6.778 ± 0.269	0.518 ± 0.02
**150**	6.787 ± 0.230	1.623 ± 0.296	0.271 ± 0.133	3.033 ± 0.255	0.518 ± 0.02
**300**	6.834 ± 0.171	0.471 ± 0.160	0.150 ± 0.087	0.477 ± 0.074	0.518 ± 0.02

7.4. SEPP-SABRE kinetics of IrHH hydrogens as a function of [pyruvate]

Sample: 4 mM of [Ir-d_{22}], 20 mM of DMSO, and a variable amount of ^{13}C-pyr between 40 and 174 mM in 600 µL of methanol-d_4.

Experiment: In all the experiments below, we used a 9.4 T NMR system, pH_2 at 8.5 bar pressure, 265 K, and a SEPP-SABRE sequence for ^{1}H hyperpolarization of the selected proton. For each concentration of pyruvate, 4 sets of data were measured. Set 1 - hyperpolarization of H^a-3 at -29.1 ppm using SEPP-SABRE and following exchanging with H^b-**3** at -27.2 ppm, H^a-**2** at -14.98 ppm, H^b-**2** at -24.08 ppm, and free H_2 at 4.6 ppm (**Figure S7-A, E**). Set 2 - hyperpolarization of H^b-**3** using SEPP-SABRE and following exchanging with H^a-**3**, H^a-**2**, H^b-**2**, and H_2 (**Figure S7-B, F**). Set 3 - hyperpolarization of H^a-**2** using SEPP-SABRE and following exchanging with H^b-**2**, H^a-**3**, H^b-**3**, and H_2 (**Figure S7-C, G**). Set 4 - hyperpolarization of H^b-**2** using SEPP-SABRE and following exchanging with H^a-**2**, H^a-**3**, H^b-**3**, and H_2 (**Figure S7-D, H**). R_{H2} was measured for different temperatures (**Table S15**).

7.4.1. Complex 3 & 2

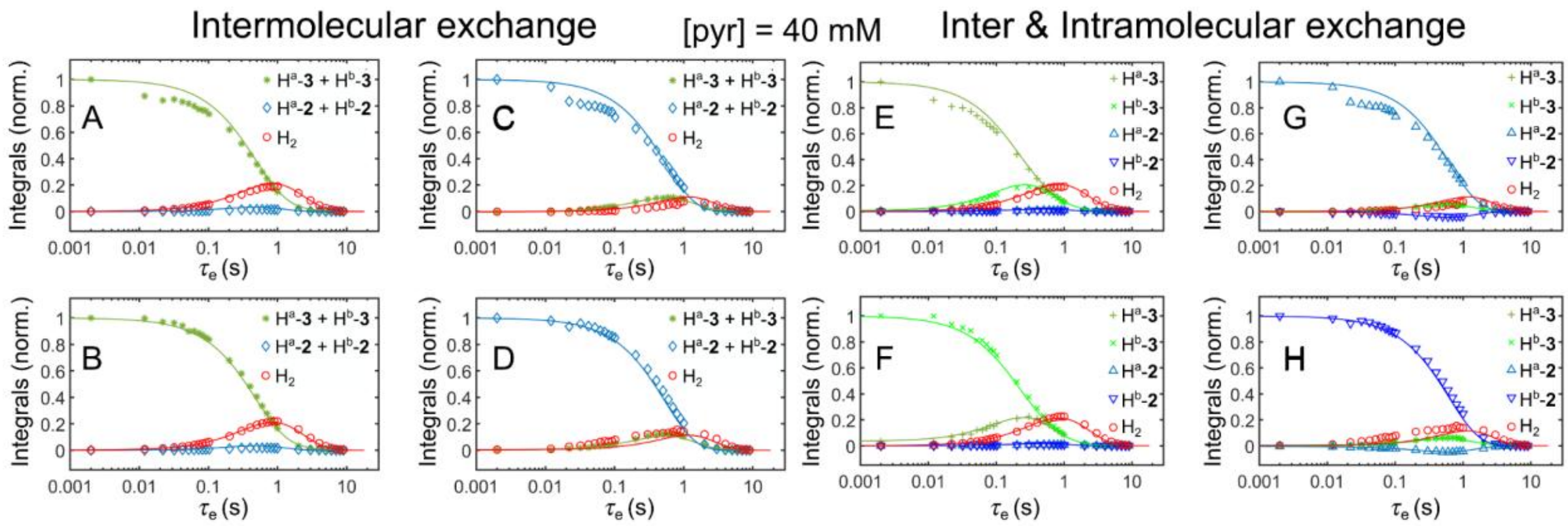

Figure S7(1): Normalized integrals of 3 and 2 SEPP-SABRE kinetics for 40 mM. ^{1}H NMR integrals of the hydride at -29.2 ppm (H^a-**3**, green plus), at -27.2 ppm (H^b-**3**, green cross), and 4.6 ppm (H_2, red circle) at 265 K. The summed integrals of **3**'s hydrogens (H^a-**3** + H^b-**3**, green star) and of **2**'s hydrogens (H^a-**2** + H^b-**2**, blue diamond) are used for the intermolecular exchange. The curves are fitted using a global fit of the model (**3**↔**2**)↔H_2 (A, B, C, D, Intermolecular exchange), and (E, F, G, H, Inter and Intramolecular exchange). Estimated exchange rate constants, dissociations, associations, and relaxation-exchanges are given in **Table S7(1).**

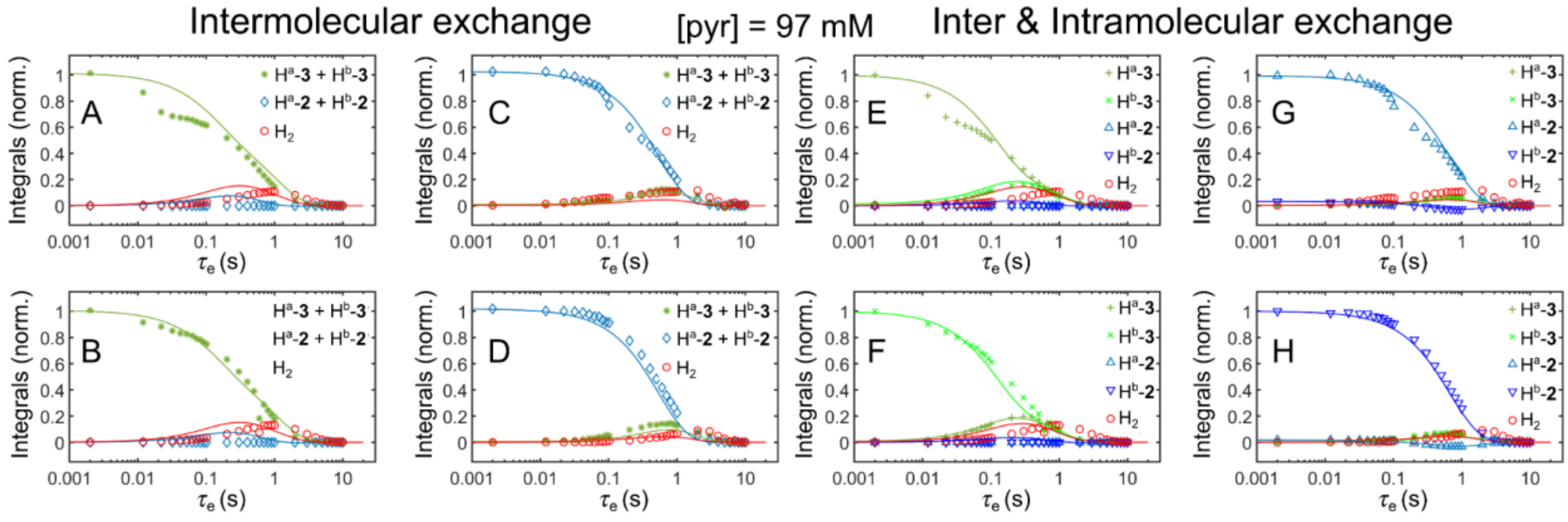

Figure S7(2): Normalized integrals of 3 and 2 SEPP-SABRE kinetics for 100 mM. ^{1}H NMR integrals of the hydride at -29.2 ppm (H^a-**3**, green plus), at -27.2 ppm (H^b-**3**, green cross), and 4.6 ppm (H_2, red circle) at 265 K. The summed integrals of **3**'s hydrogens (H^a-**3** + H^b-**3**, green star), and of **2**'s hydrogens (H^a-**2** + H^b-**2**, blue diamond) are used for the intermolecular exchange. The curves are fitted using a global fit of the model (**3**↔**2**)↔H_2 (A, B, C, D, Intermolecular exchange), and (E, F, G, H, Inter and Intramolecular exchange). Estimated exchange rate constants, dissociations, associations, and relaxation-exchanges are given in **Table S7(1).**

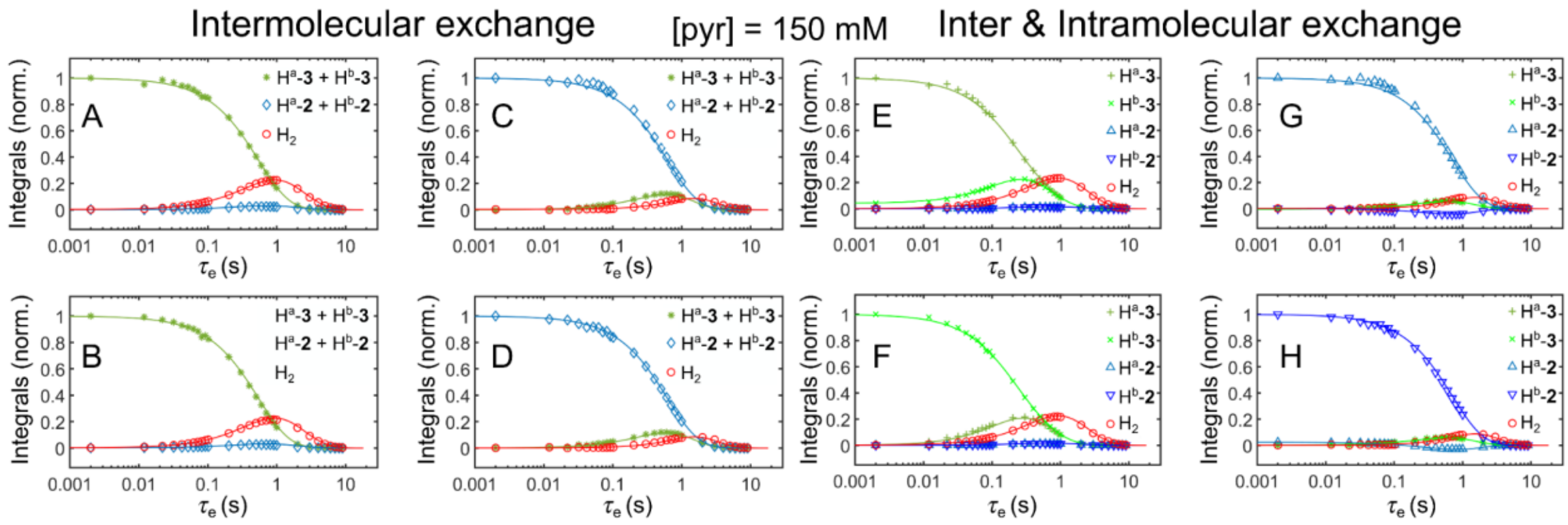

Figure S7(3): Normalized integrals of 3 and 2 SEPP-SABRE kinetics for 150 mM. ^{1}H NMR integrals of the hydride at -29.2 ppm (H^a-**3**, green plus), at -27.2 ppm (H^b-**3**, green cross), and 4.6 ppm (H_2, red circle) at 265 K. The summed integrals of **3**'s hydrogens (H^a-**3** + H^b-**3**, green star), and of **2**'s hydrogens (H^a-**2** + H^b-**2**, blue diamond) are used for the intermolecular exchange. The curves are fitted using a global fit of the model (**3**↔**2**)↔H_2 (A, B, C, D, Intermolecular exchange), and (E, F, G, H, Inter and Intramolecular exchange). Estimated exchange rate constants, dissociations, associations, and relaxation-exchanges are given in **Table S7(1).**

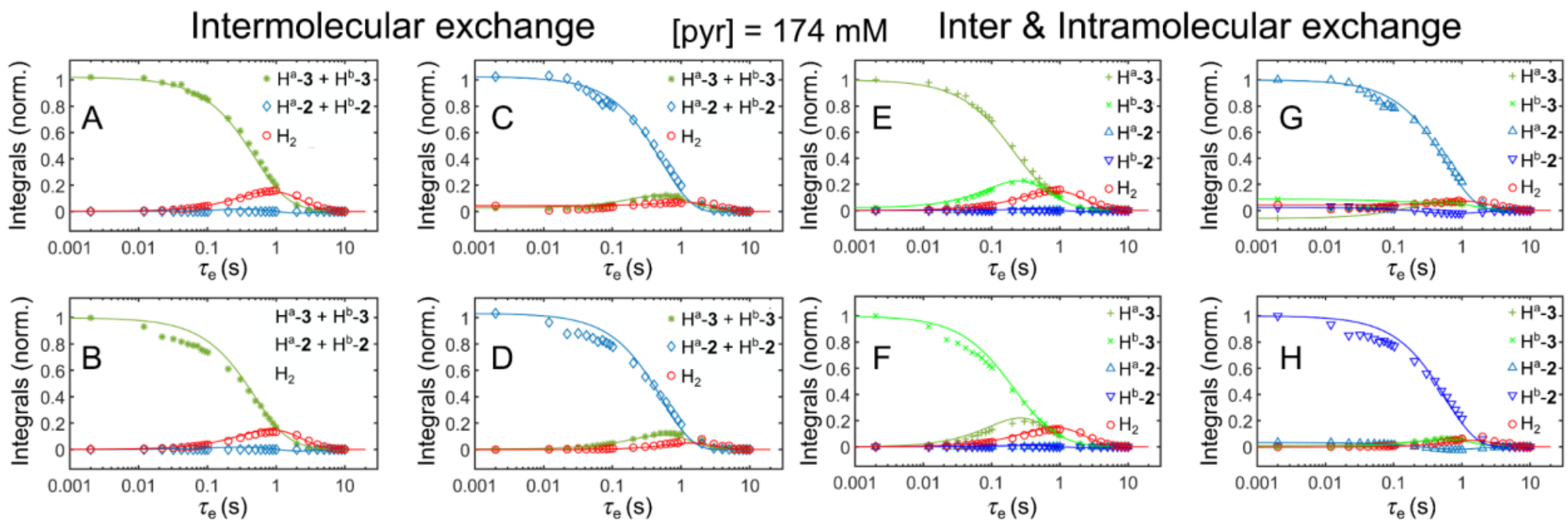

Figure S7(4): Normalized integrals of 3 and 2 SEPP-SABRE kinetics for 174 mM. ^{1}H NMR integrals of the hydride at -29.2 ppm (H^a-**3**, green plus), at -27.2 ppm (H^b-**3**, green cross), and 4.6 ppm (H_2, red circle) at 265 K. The summed integrals of **3**'s hydrogens (H^a-**3** + H^b-**3**, green star), and of **2**'s hydrogens (H^a-**2** + H^b-**2**, blue diamond) are used for the intermolecular exchange. The curves are fitted using a global fit of the model (**3**↔**2**)↔H_2 (A, B, C, D, Intermolecular exchange), and (E, F, G, H, Inter and Intramolecular exchange). Estimated exchange rate constants, dissociations, associations, and relaxation-exchanges are given in **Table S7(1).**

Table S7(1). Fitting parameters of **3** and **2** SEPP-SABRE kinetics for each concentration using 2 steps fitting process of model (**3**↔**2**)↔H_2. Parameters are defined in section 2.

[pyr] (mM)	k_{3-H} (s^{-1})	k_{2-H_2} (s^{-1})	$k_{3\to2}$ (s^{-1})	$k_{2\to3}$ (s^{-1})	k_{3ex}^{HH} (s^{-1})	k_{2ex}^{HH} (s^{-1})	$k_{3a}'^{H}$ (s^{-1})	$k_{2a}'^{H}$ (s^{-1})	R_3 (s^{-1})	R_2 (s^{-1})	R_{H2} (s^{-1})
**40**	0.726 ± 0.04	0.220 ± 0.028	0.185 ± 0.047	0.540 ± 0.039	2.248 ± 0.080	-0.209 ± 0.023	-0.162 ± 0.12	-0.046 ± 0.114	1.168 ± 0.036	1.056 ± 0.036	0.518 ± 0.02
**100**	0.683 ± 0.02	0.088 ± 0.011	0.075 ± 0.019	0.553 ± 0.015	1.950 ± 0.036	-0.176 ± 0.011	0.014 ± 0.053	0.074 ± 0.043	0.997 ± 0.017	1.022 ± 0.017	0.518 ± 0.02
**150**	0.701 ± 0.01	0.071 ± 0.007	0.122 ± 0.012	0.527 ± 0.009	2.109 ± 0.024	-0.165 ± 0.007	0.015 ± 0.034	0.073 ± 0.027	1.010 ± 0.010	0.965 ± 0.010	0.518 ± 0.02
**174**	0.515 ± 0.03	0.046 ± 0.023	0.132 ± 0.041	0.534 ± 0.033	2.470 ± 0.079	-0.141 ± 0.024	0.579 ± 0.179	-0.331 ± 0.142	1.334 ± 0.034	1.275 ± 0.036	0.518 ± 0.02

7.4.2. Complex 1

Sample: 4 mM of [Ir-*d*$_{22}$], 20 mM of DMSO, and a variable amount of ^{13}C-pyr between 40 and 174 mM in 600 µL of methanol-*d*$_4$.

Experiment: In all the experiments below, we used a 9.4 T NMR system, pH_2 at 8.5 bar pressure, 265 K, and a SEPP-SABRE sequence for ^{1}H hyperpolarization of the selected proton. For each temperature, 2 sets of data were measured. Set 1 - hyperpolarization of Ha-1 at -15.56 ppm using SEPP-SABRE and following exchanging with H^b-1 at -21.59 ppm, and free H_2 at 4.6 ppm (**Figure S7(5)-top**). Set 2 - hyperpolarization of H^b-1 using SEPP-SABRE and following the exchange with H^a-1, and H_2 (**Figure S7(5)-bottom**). R_{H2} was measured for different temperatures (**Table S15**).

Simulations:

To streamline the analysis, we employed a global fitting approach for model IrHH↔H_2 using MATLAB scripts to fit all parameters simultaneously using 2 sets of kinetics. Across both kinetics and for each temperature, we share 5 parameters: the dissociation rate constant k_{1-H_2}, exchange constant k_{1-ex}^{HH}, association rate constant $k_{1a}'^{H}$, natural H_2 relaxation exchange R_{H_2} and relaxation exchange R_{IrHH}. The reported errors are the results of such fits using MATLAB's nonlinear regression function "nlinfit". The fitting script, together with integrals, is available in Zenodo DOI: https://doi.org/10.5281/zenodo.18449765.

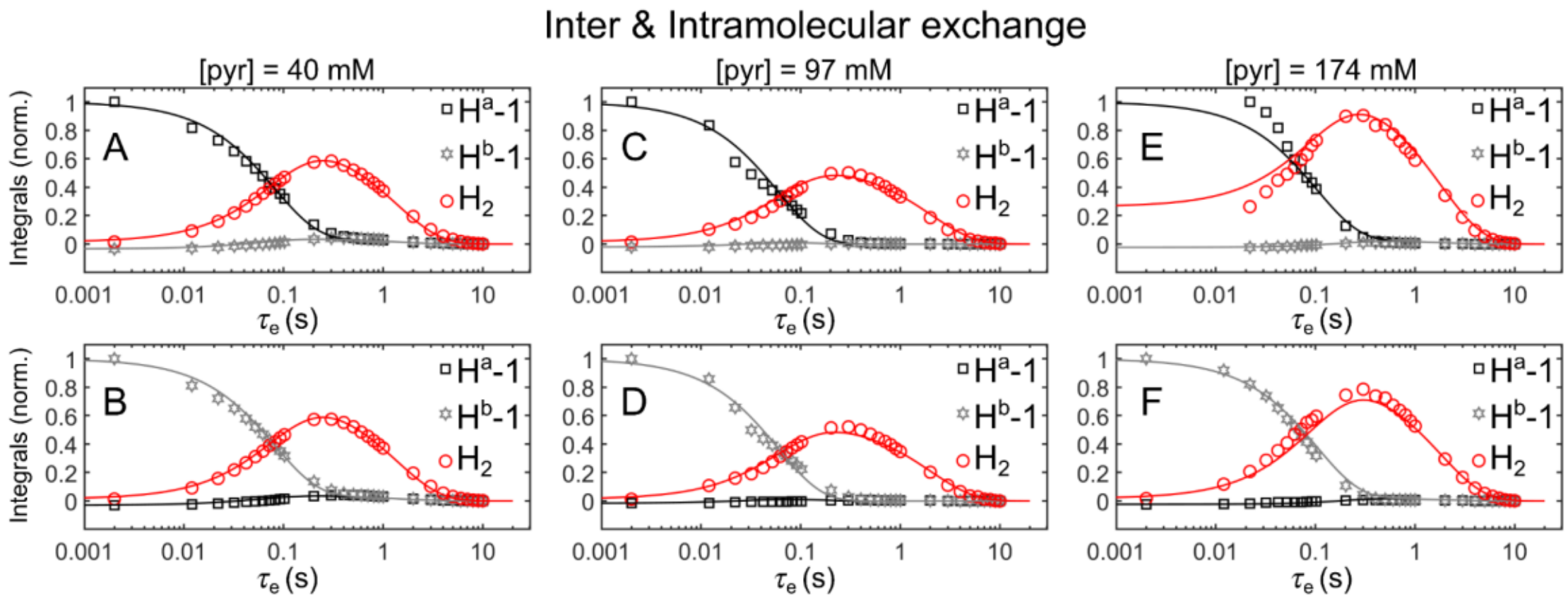

Figure S7(5): Normalized integrals of 1 SEPP-SABRE kinetics. ^{1}H NMR integrals of the hydride at -14.98 ppm (H^a-**1**, black square), at -21.59 ppm (H^b-**1**, grey star), and 4.6 ppm (H_2, red circle), of pyruvate concentration of 40 mM (A, B), 97 mM (C, D), and 174 mM (E, F). The curves are fitted using the model IrHH↔H_2 (Inter and Intramolecular exchange). Estimated exchange rate constants, dissociations, associations, and relaxation-exchanges are given in **Table S7(2).**

Table S7(2). Fitting parameters of **1** SEPP-SABRE kinetics for each pyruvate concentration using model IrHH↔H_2. Parameters are defined in section 2

**pyr (mM)**	k_{1-H_2} (s^-1^)	$k_{1-\mathrm{ex}}^{\mathrm{HH}}$ (s^-1^)	${k'_{1\mathrm{a}}}^{\mathrm{H}}$ (s^-1^)	R_{IrHH} (s^-1^)	R_{H2} (s^-1^)
**40**	8.530 ± 0.098	0.779 ± 0.099	1.129 ± 0.074	2.505 ± 0.084	0.518 ± 0.02
**97**	8.556 ± 0.165	0.735 ± 0.249	-0.071 ± 0.044	7.451 ± 0.231	0.518 ± 0.02
**174**	8.509 ± 0.268	0.457 ± 0.156	-0.152 ± 0.234	1.153 ± 0.184	0.518 0.02

7.5 Thermal kinetics of IrHH exchange of 3 as a function of temperature

Sample: 4 mM of [Ir], 40 mM of ^{13}C-pyr, and 20 mM of DMSO in 600 µL of methanol-d_4 at 6 bar.

Experiments:

To monitor the hydride ligand exchange pathways, selective ^{1}H exchange spectroscopy (SEXSY)[5,6] was used (**Figure S8**, **SI**). Due to a poor signal-to-noise ratio, each measurement required four hours of data acquisition (selnogpzs.2 experiment, 256 scans, 2-second repetition time, 16 kinetic time points; see also **Section 3 in SI**). This unsatisfactory situation stemmed from the relatively weak thermally derived ^{1}H NMR signals of IrHH hydrides at the typical ~1 mM iridium complex concentration used. Although magnetization transfer and species interchange were evident in the resulting NMR spectra, the inherent limitations in the accuracy of this technique, despite its ability to encode effects up to and including the rate-limiting step, rendered it insufficient for producing complete insights.

Results:

This section aims to show thermal exchange kinetics measured by varying temperatures from 247 K to 264 K. Selnogpzs.2 experiment was used, which is a 1D NOESY pulse sequence using a selective refocusing shaped pulse (see above references and also **Section 3 in SI**). The curves are fitted using the model IrHH↔H_2 exchange model described in **Section 1.1 of the SI**. **Figure S8** shows the experimental points and the fitting results, whereas **Table S8** presents the obtained reaction kinetic rates.

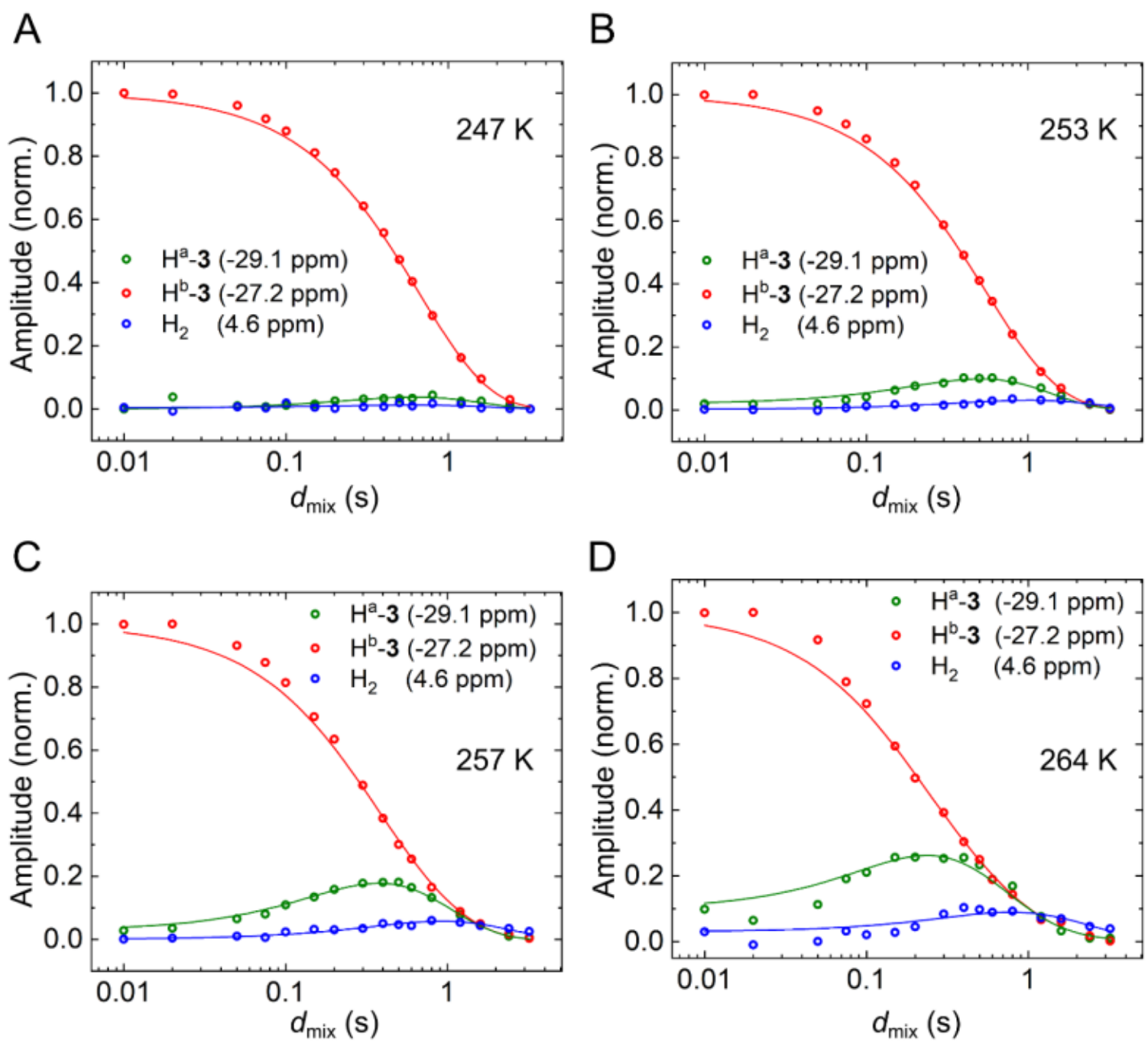

Figure S8: Normalized integrals of selnogpzs.2 thermal kinetics. ^{1}H NMR integrals of the hydride at -29.2 ppm (Ha, green), at -27.2 ppm (Hb, red), and 4.6 ppm (H_2, blue) at 247 K (A), 253 K (B), 257 K (C), and 264 K (D). The curves are fitted using the model IrHH↔H_2. Estimated exchange rate constants, dissociations, associations, and relaxation-exchanges are given in **Table S8**. Source data are provided as a Source Data file.

Table S8: Fitting parameters selnogpzs.2 thermal kinetics for each temperature.

Temperature (K)	$k_{3\text{-H2}}$ (s^{-1})	$k_{3-\mathrm{ex}}^{\mathrm{HH}}$ (s^{-1})	${k'_{3a}}^{\mathrm{H}}$ (s^{-1})	R_{H2} (s^{-1})	$R_{\mathrm{Hab\text{-}3}}$ (s^{-1})
**247**	0.04 ± 0.03	0.168 ± 0.02	-1.201 ± 1.927	1.29 ± 1.20	1.305 ± 0.03
**253**	0.08 ± 0.01	0.45 ± 0.01	-0.152 ± 0.452	0.77 ± 0.5	1.31 ± 0.03
**257**	0.166 ± 0.02	1.175 ± 0.04	-0.267 ± 0.365	1.00 ± 0.4	1.316 ± 0.02
**264**	0.20 ± 0.04	2.30 ± 0.15	0.659 ± 0.558	0.01 ± 0.54	1.606 ± 0.09

8. Pyruvate exchange

One hypothesis is that the dissociation of the pyruvate from the SABRE complex can associate again in different positions, influencing a change of both hydride' chemical shifts at -29.1 ppm and -27.2 ppm. Therefore, we measured pyruvate's exchange rates to understand the mechanism of exchange. We used the SEPP-SPINEPTplus-SABRE pulses sequence, which consists of three parts:

8.1. SEPP-SPINEPTplus – SOT to hyperpolarize pyruvate

First part frequency-selective excitation of parahydrogen-derived polarization SOT sequence (SEPP) SOT converts the PASADENA two-spin order into the magnetization of one of them. In the second part, insensitive nuclei enhanced by polarization transfer (INEPT) with selective pulses (SP) with an additional 180º pulse on both channels (SPINEPTplus) to transfer polarization from the proton to the targeted nuclei to have a net magnetization on the bound ^{13}C-pyruvate (**Figure S9 A**).

The first part, τ_1 and τ_2 delays of SEPP were optimized previously by Assaf et al.[7]. As for the second part SPINEPTplus, we performed temperature dependence experiments from 260 K to 275.5 K as a function of τ_3. Polarization is maximal when the timing is properly set for τ_3 (**Figure S9 B**). The main reason for polarization loss is the increase in the total duration of the pulse sequence, and with high temperatures, the dissociation rate increases.

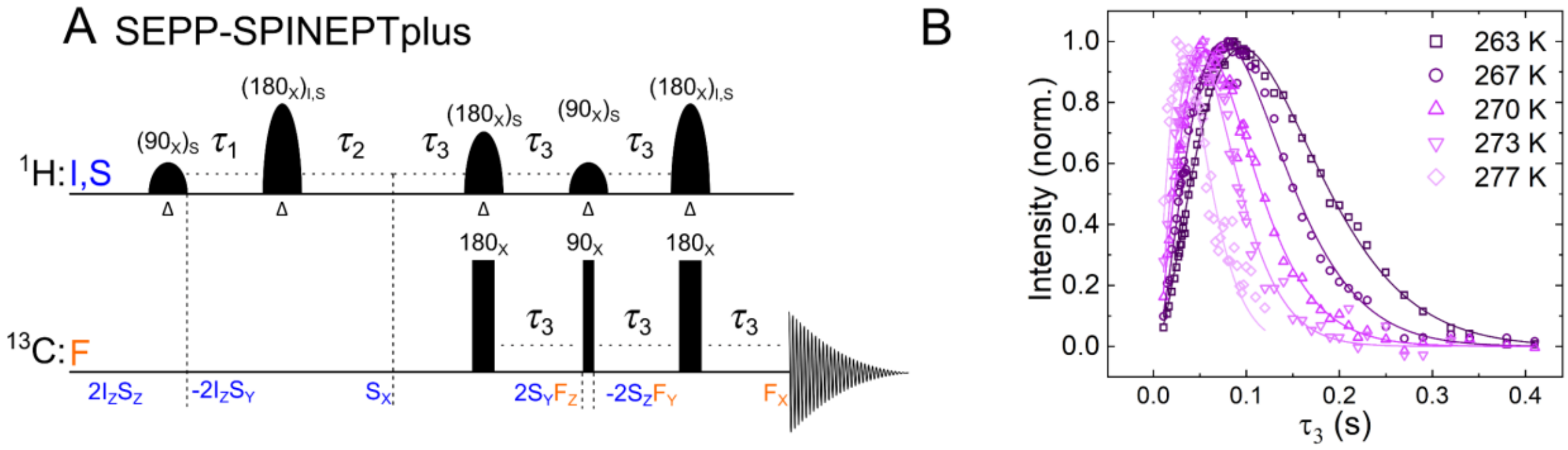

Figure S9: Scheme of SEPP-SPINEPT+ pulse sequence (A) and dependence of the SEPP-SPINEPT+ enhanced NMR signal of 2-^{13}C-ePyr (-29.1 ppm ^{1}H of **3** was selectively excited with SEPP-SPINEPTplus) on the inter-pulse delay τ_3 at different temperature (B): 263 K (squares), 267 K (circles); 270 K (up-triangles), 273 K (down-triangles), 277 K (diamond). Lines represent the global fitting by a sinus decay function $y0+A\times\sin(2\pi J\tau_3)^2 \times \exp(-4R\tau_3)$. The *J*-coupling was treated as a shared parameter across all datasets, while the relaxation rates *R* were fitted independently for each temperature. This yielded a *J*-coupling value of 0.61 ± 0.15 Hz. The extracted relaxation rates *R* exhibit a clear temperature dependence, with values of 5.0 ± 0.16 s^{-1}, 6.43 ± 0.14 s^{-1}, 8.6 ± 0.13 s^{-1}, 10.56 ± 0.13 s^{-1}, and 15.5 ± 0.2 s^{-1}, respectively. Previously, this *J* value was estimated to be 0.9 ± 0.1 Assaf et al. (43AssafJPCL). Source data are provided as a Source Data file.

8.2. SEPP-SPINEPTplus-SABRE – SOT to measure pyruvate exchange

After SEPP-SPINEPTplus, we have a net magnetization on the ^{13}C-ePyr. Then, the magnetization of all spins is rotated, and a dephasing gradient is applied (**Figure S10**). In the ideal case, this would destroy the magnetization of all nuclear spins, leaving only z-magnetization of the excited carbon. Then, after mixing time, τ_e, and subsequent excitation, all exchanging species can be observed (**Figure S11,12,13,14**).

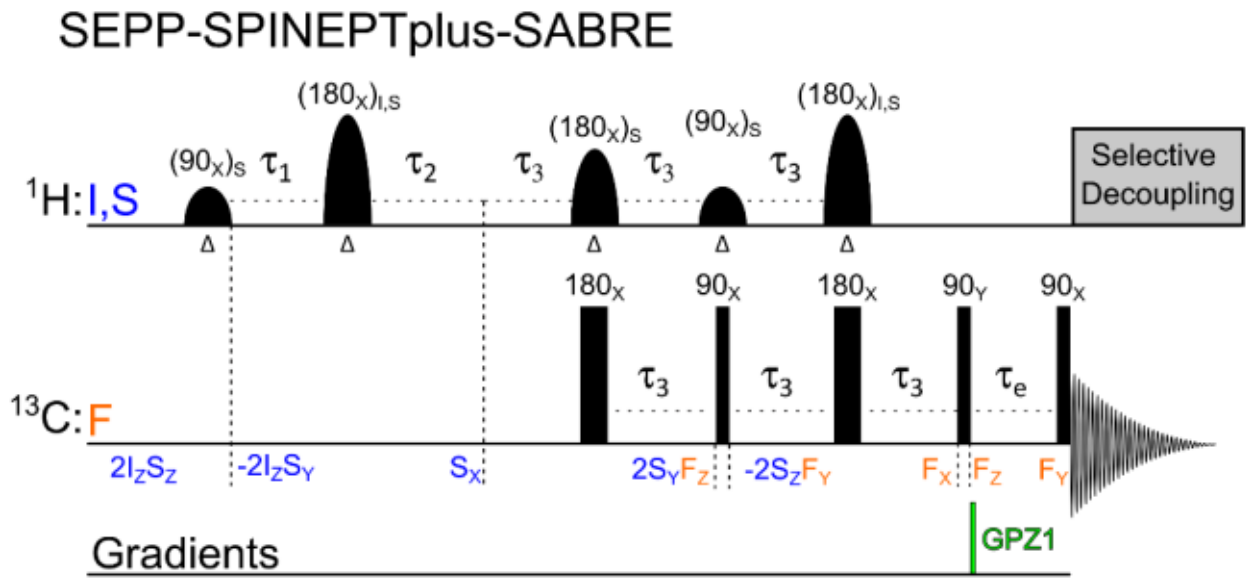

Figure S10: Scheme of SEPP-SPINEPTplus-SABRE pulse sequence. During acquisition, a selective decoupling {^{1}H} at 2.36 ppm was added to decouple the methyl group's interaction. GPZ1 is a gradient pulse of 2 ms with SMSQ10.100 shape at 31%.

These exchange rates were measured as a function of temperature, bubbling pressure, and concentrations of DMSO and pyruvate. During these measurements, the dissociation of the bound pyruvate doesn't take a direct path to the free form; however, an intermediate exchange happens before total dissociation. We observed an exchange between $^{13}C^2$-pyr **3** at 206.59 ppm and $^{13}C^2$-pyr **2** at 196.86 ppm, followed by dissociation to $^{13}C^2$-free at 202.5 ppm. We fitted the data using the carbon exchange model (**$^{13}C^2$-3 ↔$^{13}C^2$-2)↔$^{13}C^2$-free.**

8.3. Pyruvate exchange kinetics of $^{13}C^2$ of 3 and 2 in the function of temperature with 20 mM of DMSO

Sample: 4 mM of [Ir-*d22*], 40 mM of ^{13}C-pyr, and 20 mM of DMSO in 600 µL of methanol-*d4*.

Experiment: In all the experiments below, we used a 9.4 T NMR system, pH_2 pressure of 8.5 bar, and a SEPP-SPINEPTplus-SABRE sequence for ^{13}C hyperpolarization of the selected carbon. We experimented twice: first by hyperpolarizing the $^{13}C^2$-pyr of **3**, and observing its transition to **2** and then to free pyruvate **(Figure S11-top)**. In the second experiment, we hyperpolarized the $^{13}C^2$-pyr of **2** and tracked its conversion to **3** before dissociation **(Figure S11-bottom)**. The temperature was varied from 260K to 270.9 K.

Simulations: These experiments provided two sets of data, which we analyzed using a global fit with the model **($^{13}C^2$-3 ↔$^{13}C^2$-2)↔$^{13}C^2$-free**, as explained in Section 2, to extract the relevant kinetic parameters. R_{pyr} was measured for the free carbon.

Results:

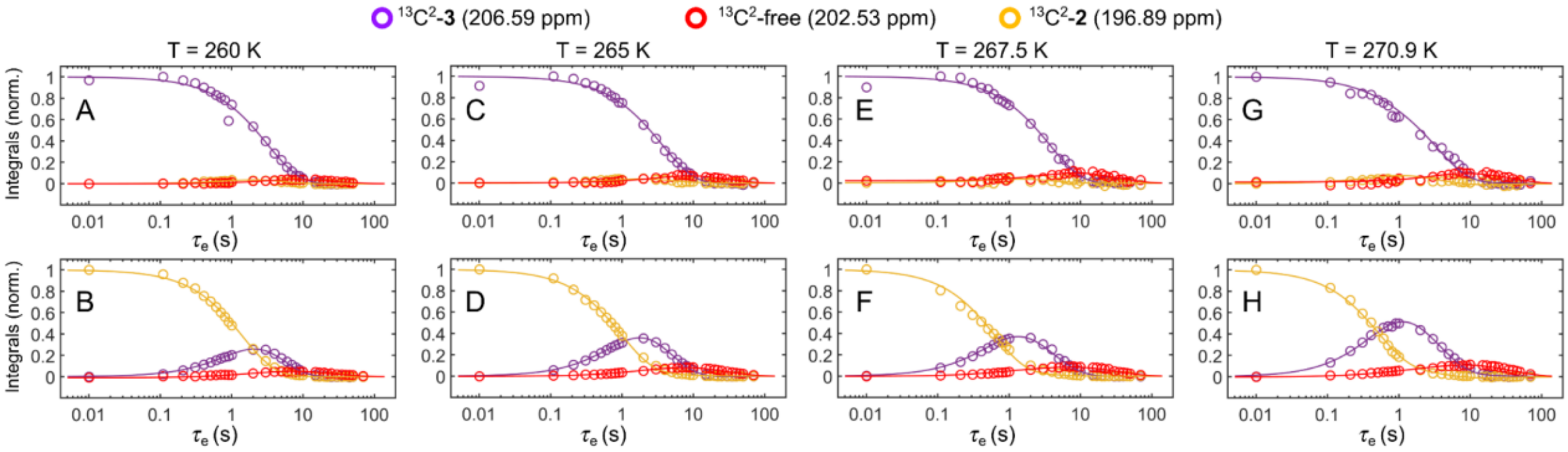

Figure S11: Normalized integrals of SEPP-SPINEPTplus-SABRE kinetics. ^{13}C NMR integrals of the equatorial $^{13}C^2$-epyr **3** at 206.59 ppm (purple), free $^{13}C^2$-free at 202.53 ppm (red), and equatorial $^{13}C^2$-ePyr **2** at 196.89 ppm (orange) at the nominal temperatures of 260 K (A,B), 265 K (C,D), 267.5 K (E,F), and 270.9 K (G,H). The curves are fitted using model **($^{13}C^2$-3 ↔$^{13}C^2$-2)↔$^{13}C^2$-free**. Estimated exchange rate constants, dissociations, associations, and relaxation-exchanges are given in **Table S9**.

Table S9. Fitting parameters of SEPP-SPINEPTplus-SABRE kinetics of both experiments for each temperature using model ($^{13}C^2$-**3** ↔$^{13}C^2$-**2**)↔$^{13}C^2$-free. Parameters are defined in section 2.

Temp (K)	Fig	$k_{3-pyr'}$ (s^{-1})	$k_{2-pyr'}$ (s^{-1})	k_{3-2} (s^{-1})	k_{2-3} (s^{-1})	$k_a'^{\mathrm{P[3]}}$ (s^{-1})	$k_a'^{\mathrm{P[2]}}$ (s^{-1})	R_3 (s^{-1})	R_2 (s^{-1})	R_{pyr} (s^{-1})
**260**	A&B	0.014 ± 0.002	0.034 ± 0.004	0.049 ± 0.007	0.350 ± 0.009	0.019 ± 0.042	-0.014 ± 0.043	0.270 ± 0.006	0.312 ± 0.011	0.034 ± 0.004
**265**	C&D	0.016 ± 0.001	0.042 ± 0.004	0.121 ± 0.006	0.630 ± 0.007	0.025 ± 0.026	-0.019 ± 0.027	0.208 ± 0.004	0.467 ± 0.009	0.033 ± 0.003
**267.5**	E&F	0.034 ± 0.003	0.087 ± 0.009	0.089 ± 0.013	0.849 ± 0.018	-0.002 ± 0.031	0.015 ± 0.030	0.210 ± 0.008	0.611 ± 0.022	0.029 ± 0.002
**270.9**	G&H	0.042 ± 0.004	0.119 ± 0.012	0.121 ± 0.008	1.251 ± 0.021	0.008 ± 0.032	0.041 ± 0.039	0.198 ± 0.010	0.470 ± 0.049	0.029 ± 0.003

8.4. Pyruvate exchange kinetics of $^{13}C^2$ of 3 and 2 in the function of temperature with 100mM of DMSO

Sample: 4 mM of [Ir-*d22*], 40 mM of ^{13}C-pyr, and 100 mM of DMSO in 600 µL of methanol-*d4*.

Experiment: In all the experiments below, we used a 9.4 T NMR system, pH_2 pressure of 8.5 bar, and a SEPP-SPINEPTplus-SABRE sequence for ^{13}C hyperpolarization of the selected carbon. We experimented twice: first by hyperpolarizing the $^{13}C^2$-pyr of **3**, and observing its transition to **2** and then to free pyruvate **(Figure S12-top)**. In the second

experiment, we hyperpolarized the $^{13}C^2$-pyr of **2** and monitored its conversion to **3** prior to dissociation **(Figure S12-bottom)**. The temperature was varied from 260K to 270.9 K.

Simulations: These experiments provided two sets of data, which we analyzed using a global fit with the model **($^{13}C^2$-3 ↔$^{13}C^2$-2)↔$^{13}C^2$-free**, as explained in Section 2, to extract the relevant kinetic parameters. R_{pyr} was measured for the free carbon.

Results: Increasing of temperature increases dissociation and exchange rates.

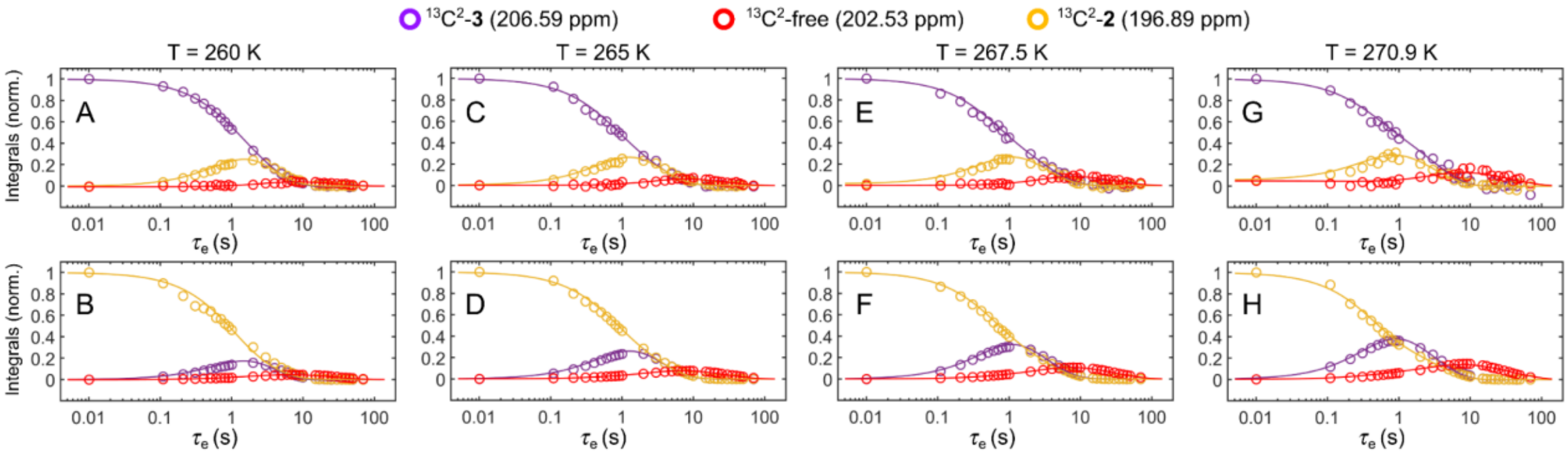

Figure S12: Normalized integrals of SEPP-SPINEPTplus-SABRE kinetics. ^{13}C NMR integrals of the equatorial $^{13}C^2$-epyr **3** at 206.59 ppm (purple), free $^{13}C^2$-free at 202.53 ppm (red), and equatorial $^{13}C^2$-epyr **2** at 196.89 ppm (orange) at the nominal temperatures of 260 K (A, B), 265 K (C, D), 267.5 K (E, F), and 270.9 K (G, H). The curves are fitted using model **($^{13}C^2$-3 ↔$^{13}C^2$-2)↔$^{13}C^2$-free**. Estimated exchange rate constants, dissociations, associations, and relaxation-exchanges are given in **Table S10**.

Table S10. Fitting parameters of SEPP-SPINEPTplus-SABRE kinetics of both experiments for each temperature using model ($^{13}C^2$-**3** ↔$^{13}C^2$-**2**)↔$^{13}C^2$-free. Parameters are defined in section 2.

**Temp (K)**	**Fig**	$k_{3-\mathrm{pyr}}$, (s^{-1})	$k_{2-\mathrm{pyr}}$, (s^{-1})	$k_{3\to2}$ (s^{-1})	$k_{2\to3}$ (s^{-1})	$k'^{\,\mathrm{P}}_{3\mathrm{a}}$ (s^{-1})	$k'^{\,\mathrm{P}}_{2\mathrm{a}}$ (s^{-1})	R_3 (s^{-1})	R_2 (s^{-1})	R_{pyr} (s^{-1})
**260**	A&B	0.010 ± 0.004	0.031 ± 0.005	0.482 ± 0.013	0.338 ± 0.012	-0.106 ± 0.069	0.113 ± 0.070	0.172 ± 0.013	0.468 ± 0.014	0.034 ± 0.004
**265**	C&D	0.010 ± 0.005	0.048 ± 0.005	0.618 ± 0.014	0.614 ± 0.014	-0.020 ± 0.052	0.025 ± 0.053	0.305 ± 0.013	0.284 ± 0.013	0.033 ± 0.003
**267.5**	E&F	0.013 ± 0.006	0.071 ± 0.006	0.723 ± 0.018	0.890 ± 0.019	0.020 ± 0.048	-0.021 ± 0.05	0.339 ± 0.015	0.221 ± 0.017	0.029 ± 0.002
**270.9**	G&H	0.031 ± 0.009	0.089 ± 0.012	0.931 ± 0.036	1.319 ± 0.040	-0.022 ± 0.065	0.017 ± 0.071	0.362 ± 0.025	0.173 ± 0.032	0.029 ± 0.003

8.5. Pyruvate exchange kinetics of $^{13}C^2$ of 3 and 2 in the function of pressure with 20 mM of DMSO

Sample: 4 mM of [Ir-d_{22}], 40 mM of ^{13}C-pyr, and 20 mM of DMSO in 600 µL of methanol-d_4.

Experiment: In all the experiments below, we used a 9.4 T NMR system at 265 K, and a SEPP-SPINEPTplus-SABRE sequence for ^{13}C hyperpolarization of the selected carbon. We experimented twice: first by hyperpolarizing the $^{13}C^2$-pyr of **3**, and observing its transition to **2** and then to free pyruvate **(Figure S13-top)**. In the second experiment, we hyperpolarized the $^{13}C^2$-pyr of **2** and tracked its conversion to **3** before dissociation **(Figure S13-bottom)**. The pressure was varied from 1 bar to 8.5 bar.

Simulations: These experiments provided two sets of data, which we analyzed using a global fit with the model **($^{13}C^2$-3 ↔$^{13}C^2$-2)↔$^{13}C^2$-free**, as explained in Section 2, to extract the relevant kinetic parameters. R_{pyr} was measured for the free carbon.

Results:

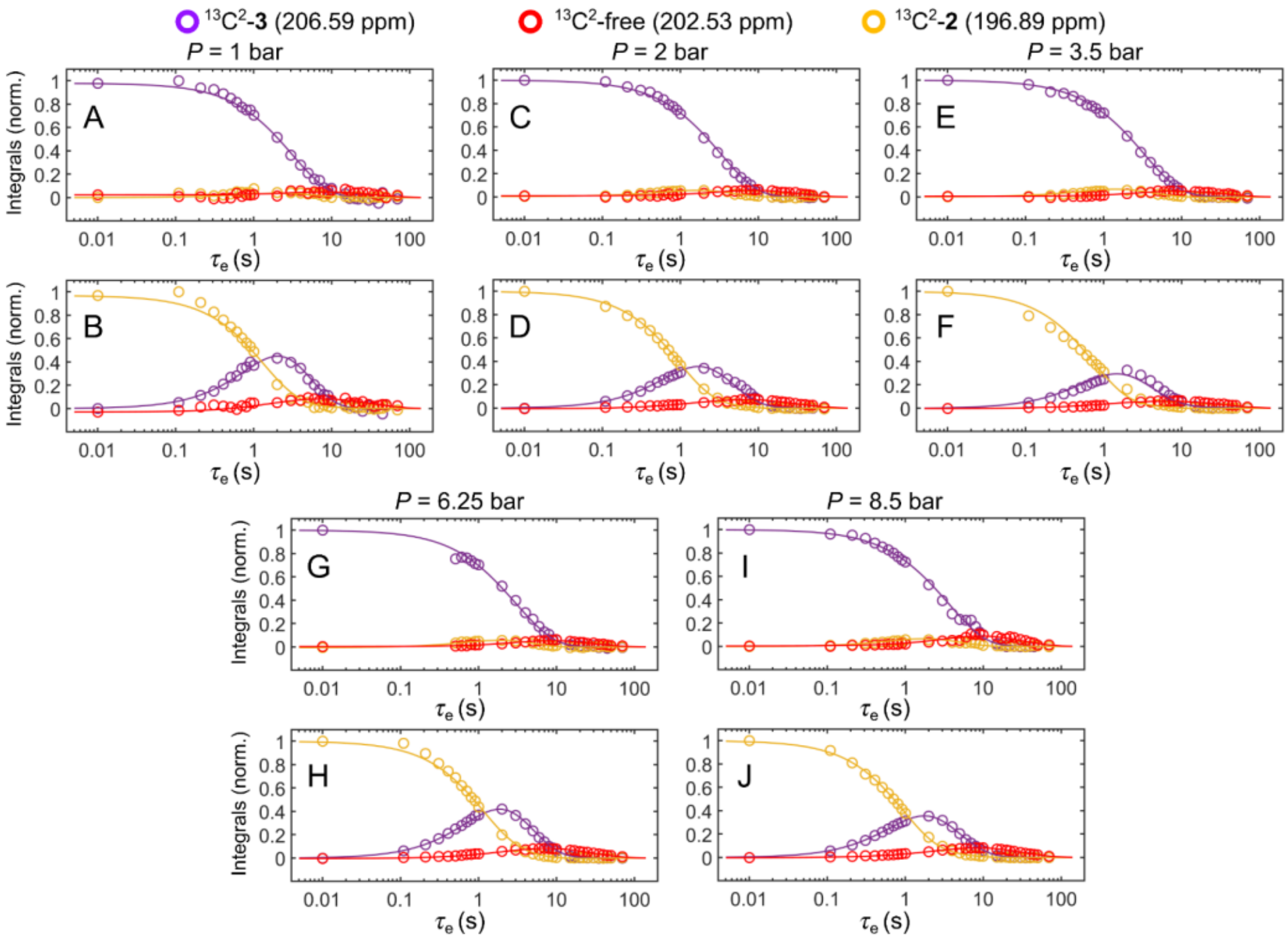

Figure S13: Normalized integrals of SEPP-SPINEPTplus-SABRE kinetics. ^{13}C NMR integrals of the equatorial $^{13}C^2$-epyr **3** at 206.59 ppm (purple), free $^{13}C^2$-free at 202.53 ppm (red), and equatorial $^{13}C^2$-epyr **2** at 196.89 ppm (orange) at 1 bar (A, B), 2 bar (C, D), 3.5 bar (E, F), 6.25 bar (G, H), and 8.5 bar (I, J) at 265 K. The curves are fitted using model **($^{13}C^2$-3↔$^{13}C^2$-2)↔$^{13}C^2$-free**. Estimated exchange rate constants, dissociations, associations, and relaxation-exchanges are given in **Table S11**.

Table S11. Fitting parameters of SEPP-SPINEPTplus-SABRE kinetics of both experiments for each pressure using model ($^{13}C^2$-**3** ↔$^{13}C^2$-**2**)↔$^{13}C^2$-free. Parameters are defined in section 2.

**Back pressure Bubbling pressure**	**Fig**	$k_{3-\mathrm{pyr}}$ (s^{-1})	$k_{2-\mathrm{pyr}}$ (s^{-1})	$k_{3\to2}$ (s^{-1})	$k_{2\to3}$ (s^{-1})	$k'^{\,\mathrm{P}}_{3a}$ (s^{-1})	$k'^{\,\mathrm{P}}_{2a}$ (s^{-1})	R_3 (s^{-1})	R_2 (s^{-1})	R_{pyr} (s^{-1})
**5 PSI 1 bar**	A,B	0.008 ± 0.0023	0.074 ± 0.007	0.050 ± 0.0011	0.642 ± 0.015	-0.047 ± 0.050	0.050 ± 0.056	0.287 ± 0.007	0.120 ± 0.016	0.033 ± 0.003
**20 PSI 2 bar**	C,D	0.014 ± 0.002	0.059 ± 0.004	0.098 ± 0.006	0.613 ± 0.008	0.041 ± 0.027	-0.042 ± 0.028	0.243 ± 0.004	0.329 ± 0.010	0.033 ± 0.003

**40 PSI 3.5 bar**	E,F	0.011 ± 0.003	0.064 ± 0.007	0.141 ± 0.012	0.602 ± 0.015	0.094 ± 0.045	-0.100 ± 0.046	0.212 ± 0.009	0.617 ± 0.020	0.033 ± 0.003
**75 PSI 6.25 bar**	G,H	0.017 ± 0.002	0.049 ± 0.004	0.092 ± 0.007	0.647 ± 0.009	0.059 ± 0.032	-0.058 ± 0.033	0.275 ± 0.005	0.126 ± 0.010	0.033 ± 0.003
**100 PSI 8.5 bar**	I,J	0.016 ± 0.001	0.042 ± 0.004	0.121 ± 0.006	0.630 ± 0.007	0.025 ± 0.026	-0.019 ± 0.027	0.208 ± 0.004	0.467 ± 0.009	0.033 ± 0.003

8.6. Pyruvate exchange kinetics of $^{13}C^2$ of 3 and 2 in the function of pressure with 100 mM of DMSO

Sample: 4 mM of [Ir-d_{22}], 40 mM of ^{13}C-pyr, and 100 mM of DMSO in 600 µL of methanol-d_4

Experiment: In all the experiments below, we used a 9.4 T NMR system at 265 K, and a SEPP-SPINEPTplus-SABRE sequence for ^{13}C hyperpolarization of the selected carbon. We experimented twice: first by hyperpolarizing the $^{13}C^2$-pyr of **3**, and observing its transition to **2** and then to free pyruvate **(Figure S14-top)**. In the second experiment, we hyperpolarized the $^{13}C^2$-pyr of **2** and tracked its conversion to **3** before dissociation **(Figure S14-bottom)**. The pressure was varied from 1 bar to 8.5 bar.

Simulations: These experiments provided two sets of data, which we analyzed using a global fit with the model **($^{13}C^2$-3 ↔$^{13}C^2$-2)↔$^{13}C^2$-free**, as explained in Section 2, to extract the relevant kinetic parameters. R_{pyr} was measured for the free carbon.

Results:

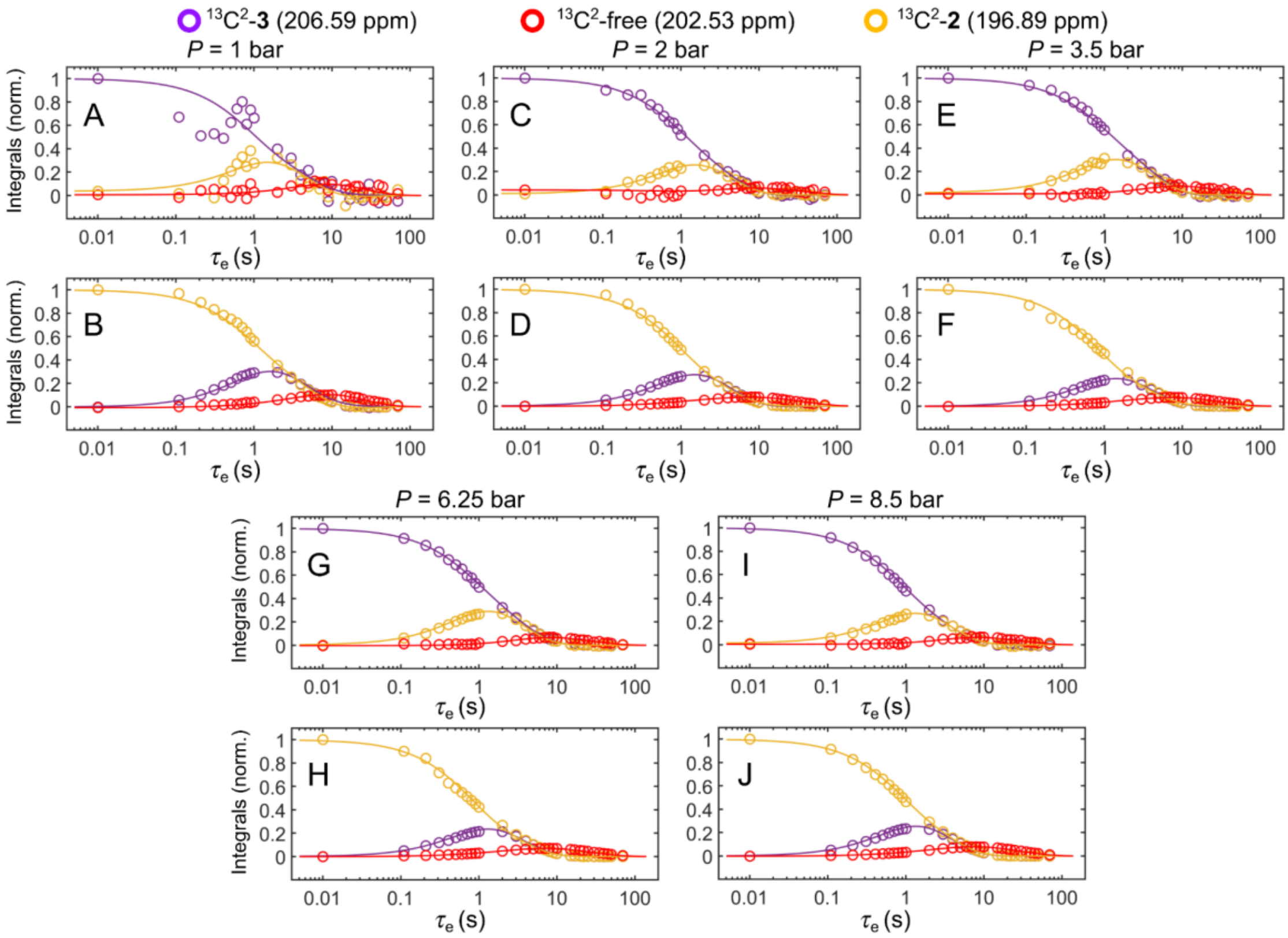

Figure S14: Normalized integrals of SEPP-SPINEPTplus-SABRE kinetics. ^{13}C NMR integrals of the equatorial $^{13}C^2$-epyr **3** at 206.59 ppm (purple), free $^{13}C^2$-fpyr at 202.53 ppm (red), and equatorial $^{13}C^2$-epyr **2** at 196.89 ppm (orange) at 1 bar (A, B), 2 bar (C, D), 3.5 bar (E, F), 6.25 bar (G, H), and 8.5 bar (I, J) at 265 K. The curves are fitted using model **($^{13}C^2$-3↔$^{13}C^2$-2)↔$^{13}C^2$-free**. Estimated exchange rate constants, dissociations, associations, and relaxation-exchanges are given in **Table S12**.

Table S12. Fitting parameters of SEPP-SPINEPTplus-SABRE kinetics of both experiments for each pressure using model ($^{13}C^2$-**3** ↔$^{13}C^2$-**2**)↔$^{13}C^2$-free. Parameters are defined in section 2.

Back pressure Bubbling pressure	Fig	$k_{3-\text{pyr}}$ (s^{-1})	$k_{2-\text{pyr}}$ (s^{-1})	$k_{3\to2}$ (s^{-1})	$k_{2\to3}$ (s^{-1})	$k'_{3a}{}^{P}$ (s^{-1})	$k'_{2a}{}^{P}$ (s^{-1})	R_3 (s^{-1})	R_2 (s^{-1})	R_{pyr} (s^{-1})
**5 PSI 1 bar**	A,B	0.023 ± 0.020	0.045 ± 0.019	0.522 ± 0.050	0.576 ± 0.051	0.177 ± 0.135	-0.169 ± 0.137	0.278 ± 0.043	0.117 ± 0.044	0.033 ± 0.003
**20 PSI 2 bar**	C,D	0.013 ± 0.004	0.041 ± 0.004	0.505 ± 0.010	0.531 ± 0.011	-0.048 ± 0.036	0.050 ± 0.038	0.215 ± 0.009	0.271 ± 0.010	0.033 ± 0.003
**40 PSI 3.5 bar**	E,F	0.010 ± 0.004	0.049 ± 0.005	0.650 ± 0.007	0.493 ± 0.007	-0.057 ± 0.034	0.085 ± 0.046	0.058 ± 0.010	0.454 ± 0.012	0.033 ± 0.003
**75 PSI 6.25 bar**	G,H	0.012 ± 0.003	0.047 ± 0.004	0.663 ± 0.009	0.537 ± 0.009	0.004± 0.031	0.001 ± 0.032	0.130 ± 0.008	0.442 ± 0.010	0.033 ± 0.003
**100 PSI 8.5 bar**	I,J	0.006 ± 0.003	0.051 ± 0.003	0.587 ± 0.007	0.567 ± 0.007	-0.004 ± 0.025	0.012 ± 0.026	0.297 ± 0.007	0.268 ± 0.007	0.033 ± 0.003

8.7. Pyruvate exchange kinetics of $^{13}C^2$ of 3 and 2 in the function of DMSO

Sample: 4 mM of [Ir-d_{22}], 40 mM of ^{13}C-pyr, and a variable amount of DMSO between 20 and 300 mM in 600 μL of methanol-d_4.

Experiment: In all the experiments below, we used a 9.4 T NMR system at 265 K, pH_2 pressure of 8.5 bar, and a SEPP-SPINEPTplus-SABRE sequence for ^{13}C hyperpolarization of the selected carbon. We experimented twice: first by hyperpolarizing the $^{13}C^2$-pyr of **3**, and observing its transition to **2** and then to free pyruvate **(Figure S15-top)**. In the second experiment, we hyperpolarized the $^{13}C^2$-pyr of **2** and tracked its conversion to **3** before dissociation **(Figure S15-bottom)**.

Simulations: These experiments provided two sets of data, which we analyzed using a global fit with the model **($^{13}C^2$-3 ↔$^{13}C^2$-2)↔$^{13}C^2$-free**, as explained in Section 2, to extract the relevant kinetic parameters. R_{pyr} was measured for the free carbon.

Results:

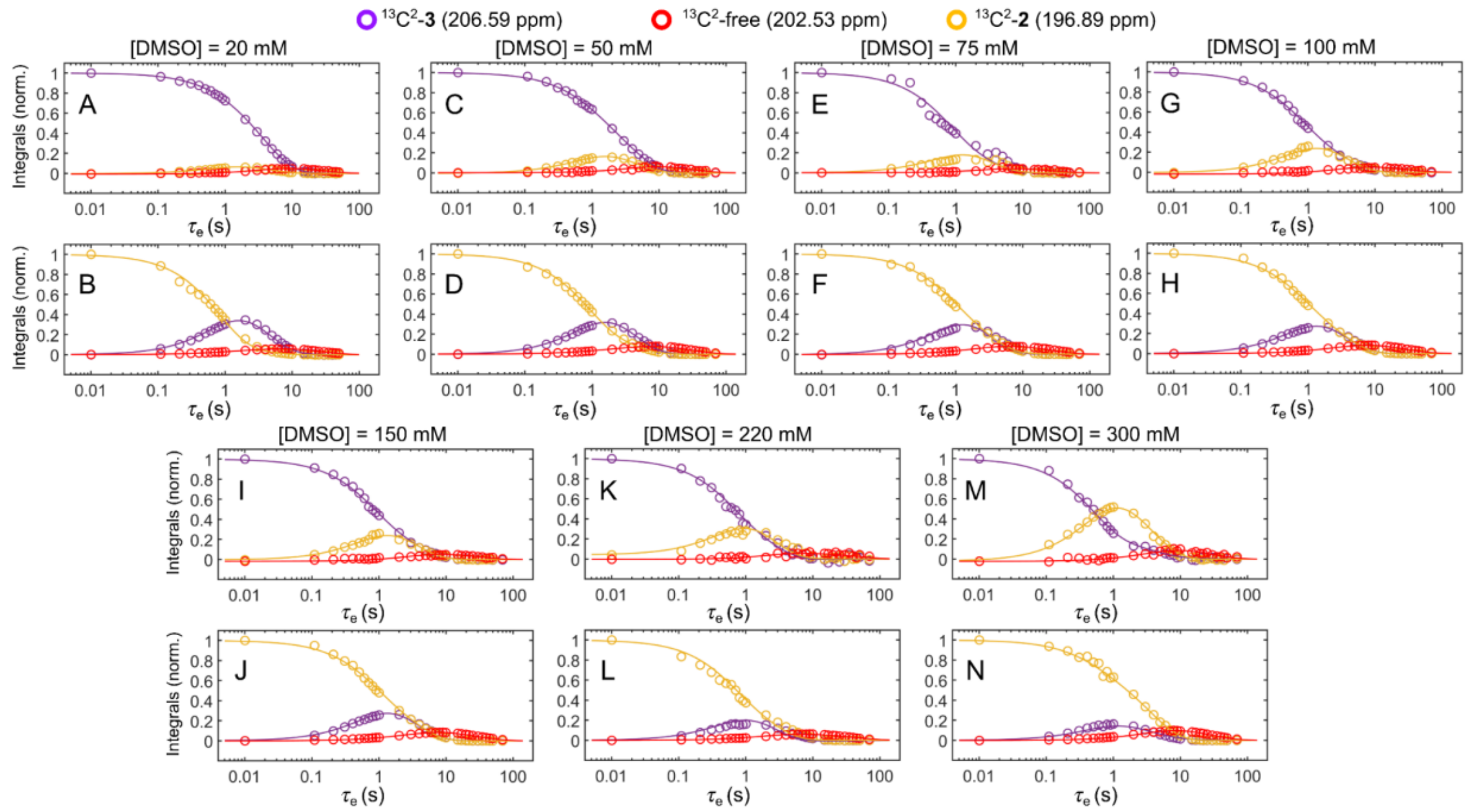

Figure S15: Normalized integrals of SEPP-SPINEPTplus-SABRE kinetics. ^{13}C NMR integrals of the equatorial $^{13}C^2$-epyr **3** at 206.59 ppm (purple), free $^{13}C^2$-fpyr at 202.53 ppm (red), and equatorial $^{13}C^2$-epyr **2** at 196.89 ppm (orange) for 20 mM of [DMSO] (A, B), 50 mM (C, D), 75mM (E, F), 100 mM (G, H), 150 mM (I, J), 220 mM (K, L),

and 300 mM (M, N) at 265 K. The curves are fitted using model **($^{13}C^2$-3↔$^{13}C^2$-2)↔$^{13}C^2$-free**. Estimated exchange rate constants, dissociations, associations, and relaxation-exchanges are given in **Table S13**.

Table S13. Fitting parameters of SEPP-SPINEPTplus-SABRE kinetics of both experiments for each concentration using model ($^{13}C^2$-**3** ↔$^{13}C^2$-**2**)↔$^{13}C^2$-free. Parameters are defined in section 2.

DMSO (mM)	Fig	$k_{3-pyr'}$ (s^{-1})	$k_{2-pyr'}$ (s^{-1})	$k_{3\to2}$ (s^{-1})	$k_{2\to3}$ (s^{-1})	k'^{P}_{3a} (s^{-1})	k'^{P}_{2a} (s^{-1})	R_3 (s^{-1})	R_2 (s^{-1})	R_{pyr} (s^{-1})
**20**	A,B	0.016 ± 0.001	0.042 ± 0.004	0.121 ± 0.006	0.630 ± 0.007	0.025 ± 0.026	-0.019 ± 0.027	0.208 ± 0.004	0.467 ± 0.009	0.033 ± 0.003
**50**	C,D	0.012 ± 0.001	0.049 ± 0.002	0.311 ± 0.005	0.596 ± 0.006	0.012 ± 0.018	-0.012 ± 0.019	0.213 ± 0.004	0.314 ± 0.006	0.033 ± 0.003
**75**	E,F	0.012 ± 0.009	0.048 ± 0.009	0.414 ± 0.023	0.650 ± 0.026	-0.049 ± 0.082	0.06 ± 0.085	0.593 ± 0.025	0.070 ± 0.024	0.033 ± 0.003
**100**	G,H	0.022 ± 0.003	0.040 ± 0.004	0.533 ± 0.009	0.607 ± 0.010	0.024 ± 0.034	-0.02 ± 0.034	0.383 ± 0.009	0.188 ± 0.009	0.033 ± 0.003
**150**	I,J	0.020 ± 0.010	0.040 ± 0.009	0.849 ± 0.031	0.595 ± 0.028	-0.155 ± 0.114	0.144 ± 0.114	0.338 ± 0.030	0.489 ± 0.026	0.033 ± 0.003
**220**	K,L	0.012 ± 0.013	0.045 ± 0.009	1.308 ± 0.036	0.592 ± 0.028	-0.317± 0.113	0.320 ± 0.114	0.132 ± 0.031	0.418 ± 0.020	0.033 ± 0.003
**300**	M,N	0.007 ± 0.009	0.048 ± 0.004	1.556 ± 0.022	0.483 ± 0.015	0.000 ± 0.049	0.000 ± 0.050	0.477 ± 0.021	0.209 ± 0.009	0.033 ± 0.003

8.8. Pyruvate exchange kinetics of $^{13}C^2$ of 3 and 2 in the function of pyruvate

Sample: 4 mM of [Ir-d_{22}], 20 mM of DMSO, and a variable amount of ^{13}C-pyr between 40 and 174 mM in 600 µL of methanol-d_4.

Experiment: In all the experiments below, we used a 9.4 T NMR system at 265 K, pH_2 pressure of 8.5 bar, and a SEPP-SPINEPTplus-SABRE sequence for ^{13}C hyperpolarization of the selected carbon. We experimented twice: first by hyperpolarizing the $^{13}C^2$-pyr of **3**, and observing its transition to **2** and then to free pyruvate **(Figure S16-top)**. In the second experiment, we hyperpolarized the $^{13}C^2$-pyr of **2** and tracked its conversion to **3** before dissociation **(Figure S16-bottom)**.

Simulations: These experiments provided two sets of data, which we analyzed using a global fit with the model **($^{13}C^2$-3 ↔$^{13}C^2$-2)↔$^{13}C^2$-free**, as explained in Section 2, to extract the relevant kinetic parameters. R_{pyr} was measured for the free carbon.

Results:

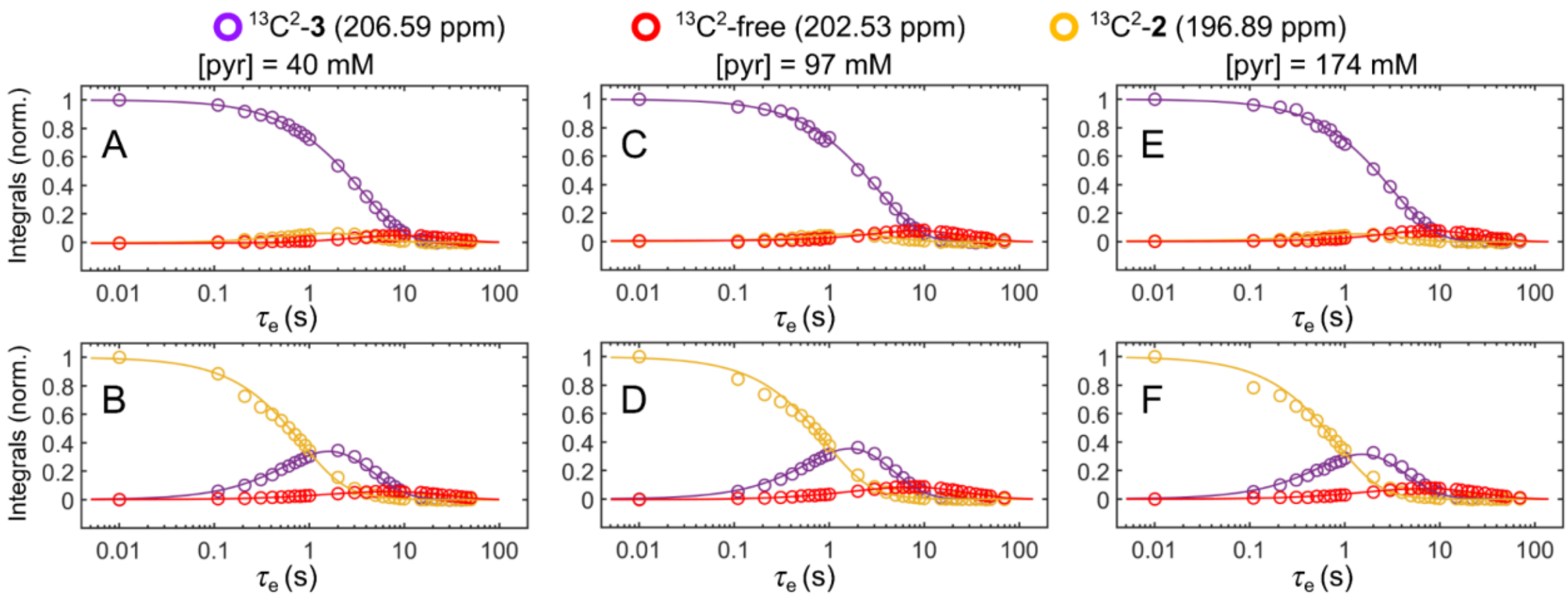

Figure S16: Normalized integrals of SEPP-SPINEPTplus-SABRE kinetics. ^{13}C NMR integrals of the equatorial $^{13}C^2$-epyr **3** at 206.59 ppm (purple), free $^{13}C^2$-fpyr at 202.53 ppm (red), and equatorial $^{13}C^2$-epyr **2** at 196.89 ppm (orange) for 40 mM of pyruvate (A, B), 97 mM (C, D), and 174 mM (E, F). The curves are fitted using model **($^{13}C^2$-3↔$^{13}C^2$-2)↔$^{13}C^2$-free**. Estimated exchange rate constants, dissociations, associations, and relaxation-exchanges are given in **Table S14**.

Table S14. Fitting parameters of SEPP-SPINEPTplus-SABRE kinetics of both experiments for each concentration using model ($^{13}C^2$-**3** ↔$^{13}C^2$-**2**)↔$^{13}C^2$-free. Parameters are defined in section 2.

Pyr (mM)	Fig	$k_{3-\mathrm{pyr}}$, (s-1)	$k_{2-\mathrm{pyr}}$, (s-1)	$k_{3\to2}$ (s-1)	$k_{2\to3}$ (s-1)	$k'^{\,\mathrm{P}}_{3a}$ (s-1)	$k'^{\,\mathrm{P}}_{2a}$ (s-1)	R_3 (s-1)	R_2 (s-1)	R_{pyr} (s-1)
**40**	A&B	0.016 ± 0.001	0.042 ± 0.004	0.121 ± 0.006	0.630 ± 0.007	0.025 ± 0.026	-0.019 ± 0.027	0.208 ± 0.004	0.467 ± 0.009	0.033 ± 0.003
**97**	E&F	0.024 ± 0.002	0.059 ± 0.004	0.095 ± 0.007	0.627 ± 0.009	0.014 ± 0.024	-0.011 ± 0.025	0.240 ± 0.005	0.348 ± 0.011	0.033 ± 0.003
**174**	G&H	0.027 ± 0.003	0.054 ± 0.006	0.105 ± 0.009	0.608 ± 0.011	0.046 ± 0.029	-0.041 ± 0.03	0.237 ± 0.007	0.505 ± 0.015	0.033 ± 0.003

9. Dissociation constant rates of $^{13}C^2$-3 & $^{13}C^2$-2

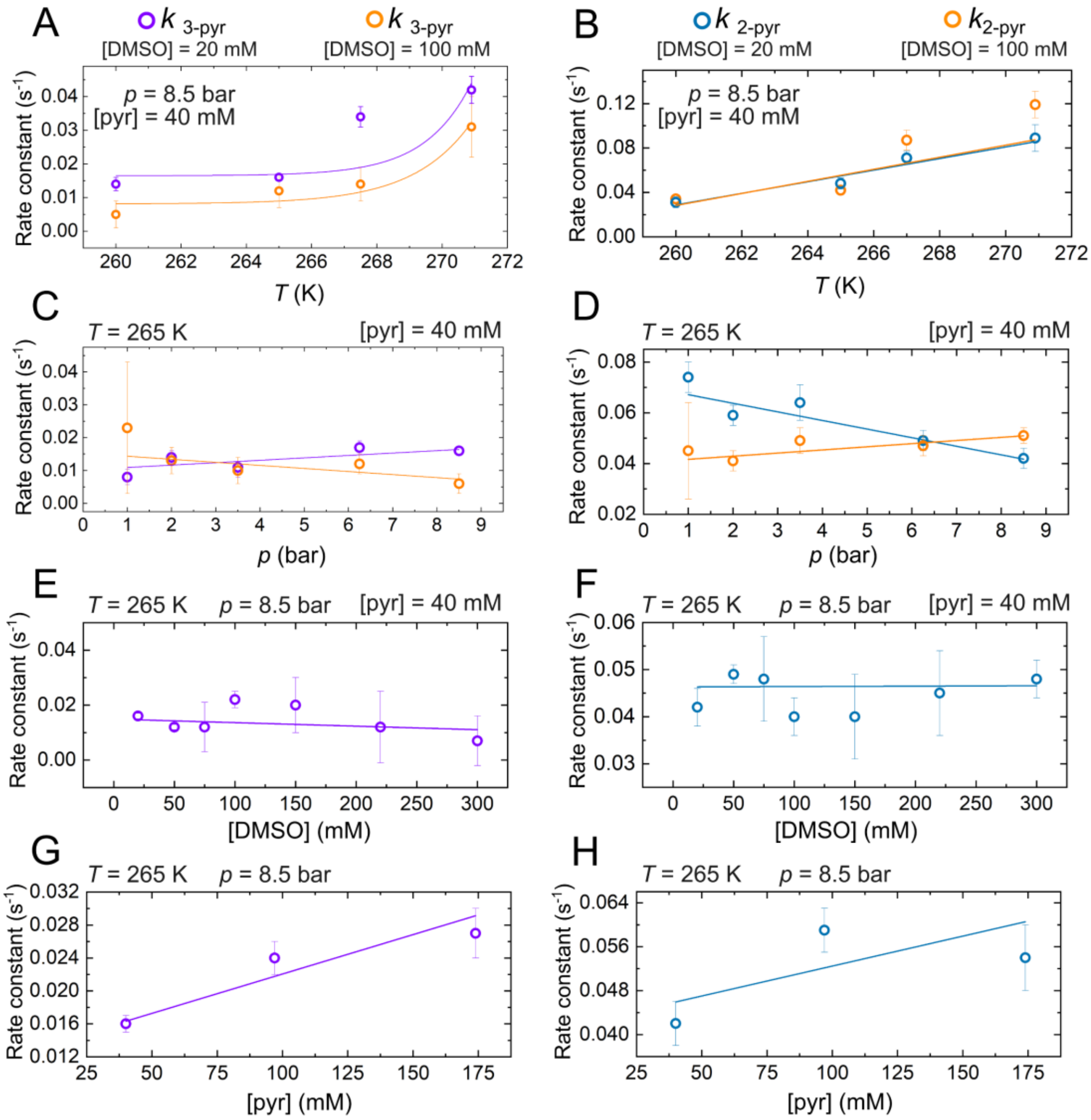

Figure S17: Dissociation rate of $^{13}C^2$ of 3 and 2. Dissociation constant rates $k_{3-\mathrm{pyr}}$, and $k_{2-\mathrm{pyr}}$, of **tables S7-12** are plotted as a function of temperature (A, B), [DMSO] (C, D), pH$_2$ pressure (E, F), and [pyr] (G, H) by performing a global fit of the model IrHH↔H$_2$. $k_{3-\mathrm{pyr}}$ dissociation constant rate of $^{13}C^2$-**3** (purple) with 20 mM of DMSO in the sample and 100 mM of DMSO (orange). $k_{2-\mathrm{pyr}}$ dissociation constant rate of $^{13}C^2$-**2** (blue) with 20 mM of DMSO in the sample and 100 mM of DMSO (orange). Error bars represent the standard errors of the fitted parameters obtained from nonlinear least-squares fitting of corresponding kinetics using MATLAB (nlinfit), calculated from the residual covariance matrix of the fit. Source data are provided as a Source Data file.

10. T_1 measurements for H_2 (4.6 ppm) and free-$^{13}C^2$ (202.5 ppm) as a function of temperature

Sample: 60 mM of ^{13}C-pyr, in 400 μL of methanol-d_4.

Experiment: We performed an inversion recovery experiment to measure the T1 of the system's free hydrogen and free carbon, thereby reducing the number of fitting parameters in the models. The sample was bubbled with H_2 for 30 min at 8.5 bar to ensure that no more oxygen remained, which might affect the relaxation time. The sample was maintained at a pressure of 8.5 bar throughout the measurement.

Results:

Table S15: T_1 measurements of H_2 and free-$^{13}C^2$.

Temperature (K)	T_1 of H_2 (s)	T_1 of Free-$^{13}C^2$ (s)
260	1.94 ± 0.07	29.24 ± 1.28
265	1.93 ± 0.07	30.27 ± 1.35
267.5	1.88 ± 0.06	34.02 ± 1.26
270.9	1.90 ± 0.03	34.34 ± 1.74
275.5	1.82 ± 0.06	35.17 ± 1.28

11. Exchange rates of $H^{a,b}$-1

Sample: In this section, 4 sets of experiments were performed. In the case of [DMSO] and [pyr] experiments, the concentration of a key component was systematically varied.

1. **Temperature and pressure experiments (A, C):** 4 mM of [Ir-*d*22], 20 mM of DMSO, and 40 mM ^{13}C-pyr in 600 µL of methanol-*d*3.
2. **[DMSO] experiments (B):** 4 mM of [Ir-*d*22], a variable amount of DMSO, and 40 mM ^{13}C-pyr in 600 µL of methanol-*d*3.
3. **[pyr] experiments (D):** 4 mM of [Ir-*d*22], 20 mM of DMSO, and a variable amount of ^{13}C-pyr in 600 µL of methanol-*d*3.

Experiment: We used a 9.4 T NMR system at 265 K, and a pH_2 pressure of 8.5 bar.

Results: we plotted the exchange rate k_{1ex}^{HH} and dissociation rate $k_{1-\mathrm{H2}}$ of **tables 4(2), 5(2), 6(2) and 7(2)**.

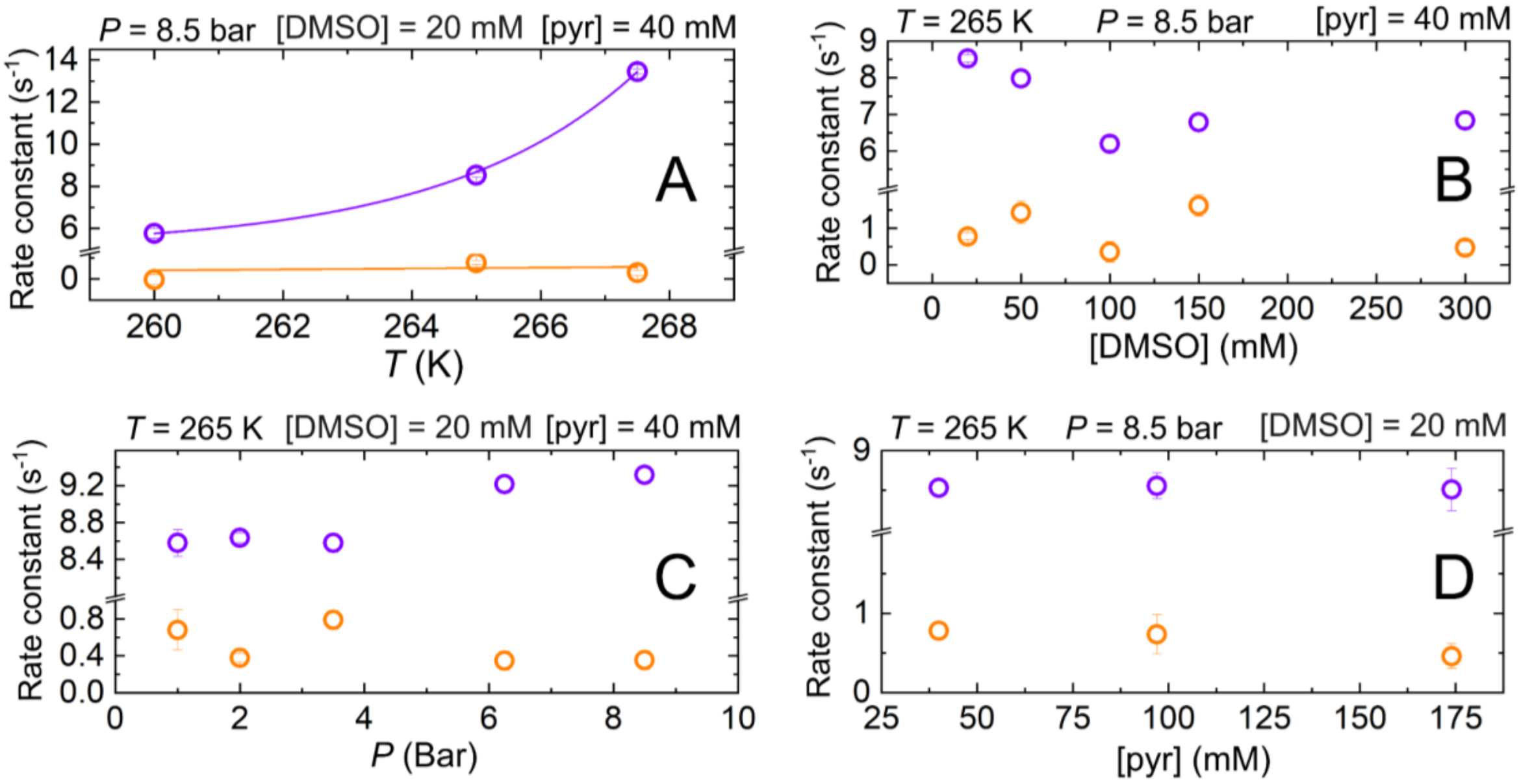

Figure S18: IrHH exchange rates of $H^{a,b}$-1. Extracted parameters as a function of temperature (A), [DMSO] (B), pH_2 pressure (C), and [pyr] (D) by performing a global fit of the model IrHH↔H_2. Both hydrogens can exchange within the complex, with the exchange rate k_{1ex}^{HH} (orange), dissociate from the complex with the exchange rate $k_{1-\mathrm{H2}}$ (purple). Error bars represent the standard errors of the fitted parameters obtained from nonlinear least-squares fitting using MATLAB (nlinfit), calculated from the residual covariance matrix of the fit. Source data are provided as a Source Data file.

12. Thermal polarized studies on complexes 1, 2 & 3 to determine their relative populations

Sample: In this section, three sets of experiments were performed. In each case, the concentration of a key component was systematically varied.

1. **Temperature experiments:** 4 mM of [Ir-d_{22}], 20 mM of DMSO, and 40 mM ^{13}C-pyr in 600 µL of methanol-d_3.
2. **[DMSO] experiments:** 4 mM of [Ir-d_{22}], a variable amount of DMSO, and 40 mM ^{13}C-pyr in 600 µL of methanol-d_3.
3. **[pyr] experiments:** 4 mM of [Ir-d_{22}], 20 mM of DMSO, and a variable amount of ^{13}C-pyr in 600 µL of methanol-d_3.

Experiment: We used a 9.4 T NMR system at 265 K, a pH_2 pressure of 8.5 bar, and 100 scans of a ^{1}H thermal spectrum using the "zgesgp" sequence to suppress the water signal.

Table S16(1) Normalized integrals of IrHH resonances of complexes **1**, **2**, and **3** as a function of temperature with 20 mM of DMSO and 40 mM of pyruvate. All data were normalized, and relative to the total hydride signal at the maximum of all complexes.

Temp (K)	**3**	**2**	**1**	**1 + 2 + 3**	All complexes
260	0.37724	0.12694	0.21128	0.71546	1
265	0.39852	0.11821	0.18681	0.70353	0.94847
267.5	0.32135	0.05676	0.10184	0.47995	0.88735
270.9	0.37841	0.09491	0.12507	0.59838	0.84191
275.5	0.36409	0.07416	0.09432	0.53257	0.76438

Table S16(2) Normalized integrals of IrHH resonances of complexes **1**, **2**, and **3** as a function of DMSO concentrations at 265 K. All data were normalized to the maximum of all complexes.

[DMSO] (mM)	**3**	**2**	**1**	**1 + 2 + 3**	All complexes
20	0.34179	0.09387	0.1612	0.59686	0.91268
70	0.1189	0.25328	0.30509	0.67727	0.9604
120	0.08108	0.26103	0.32552	0.66763	0.98339
170	0.02448	0.28346	0.3804	0.68834	1

Table S16(3) Normalized integrals of IrHH lines of complexes **1**, **2**, and **3** as a function of pyruvate concentrations at 265 K. All data were normalized to the maximum of all complexes.

[pyr] (mM)	**3**	**2**	**1**	**1 + 2 + 3**	All complexes
30	0.388	0.09647	0.1414	0.62596	1
50	0.36278	0.09605	0.11784	0.57668	0.98863
64	0.33224	0.07449	0.09774	0.50447	0.93334
100	0.27734	0.08418	0.11759	0.47911	0.88515

Table S16(4) Normalized integrals of IrHH lines of complexes **1**, **2**, and **3** as a function of temperature from 220 K to 259 K with 20 mM of DMSO and 40 mM of pyruvate. All data were normalized to the maximum of all complexes. All these experiments were done in Finland.

Temp (K)	**3**	**2**	**1**	**1 + 2 + 3**	All complexes

220	0.05608	0.43972	0.23668	0.73248	1
235	0.13281	0.25325	0.30696	0.69301	0.91001
247	0.24789	0.12268	0.19273	0.5633	0.69963
259	0.35108	0.10711	0.14667	0.60487	0.84764

All experiments in **Table S16(1-3)** were conducted in Kiel, Germany, and experiments in **Table S16(4)** were conducted in Oulu, Finland. The data collected by both groups show consistent and continuous trends: we measured in the temperature range of 260-275.5 K, while the other set of measurements was performed between 220-260 K. The overlapping pattern across both datasets supports the validity and continuity of our results. Specifically, the integrals of complex **3** increase as a function of temperature, while the integrals corresponding to complexes **1** and **2** decrease accordingly.

13. ^{1}H-^{1}H-NOE

Sample: 4 mM of [Ir-d_{22}], 20 mM of DMSO, and 40 mM ^{13}C-pyr in 600 µL of methanol-d_4.

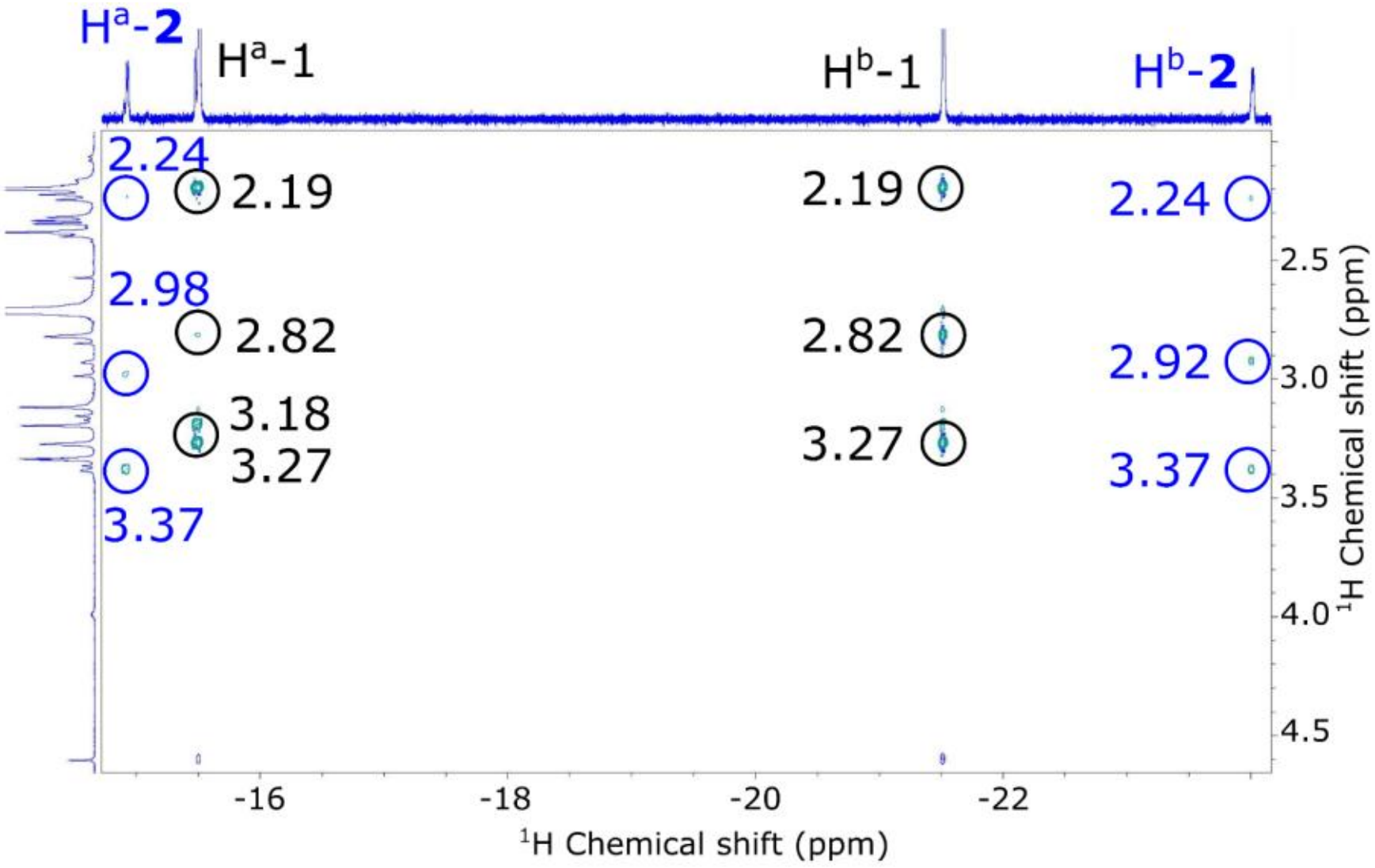

Figure S19: ^{1}H-^{1}H NOESY experiment. The experiment was conducted at 243 K, revealing interactions between the IrHH hydrides of **1** and **2** and the DMSO and pyruvate hydrogens.

14. ^{13}C-DMSO-HMQC

Sample: [Ir] = 4.9 mM, [not labeled Pyr] = 51 mM, [^{13}C-DMSO]=20 mM, Meod-d_3 = 400 uL.

Experiment: ^{1}H-^{13}C HMQC at 263K, at 8.5 bar of normal hydrogen. The J interaction was set to 10 Hz, ns = 45 scans, aq = 0.81 s, d1 = 3 s. estimated T_1 = 1 s.

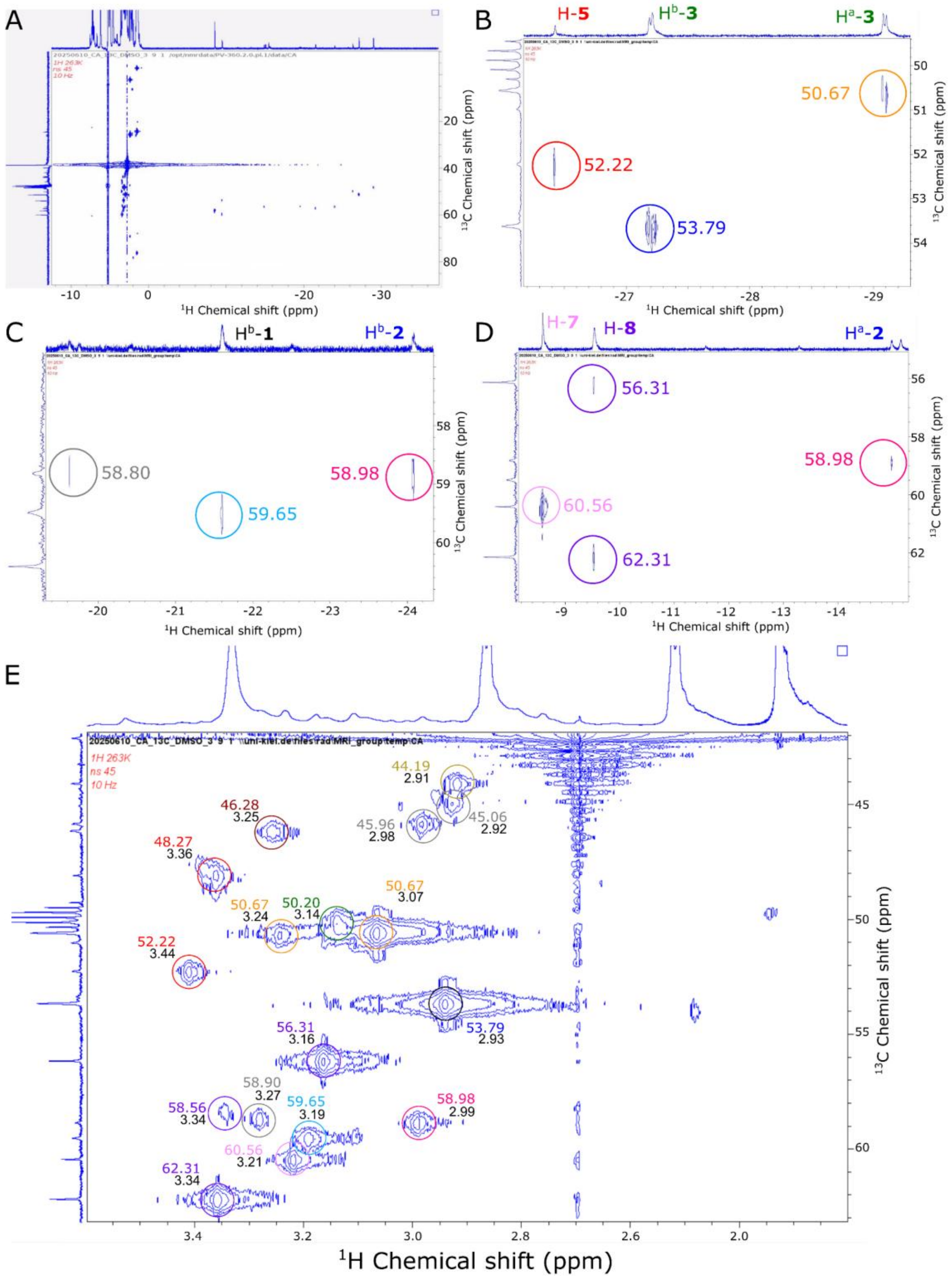

Figure S20: ^{1}H-^{13}C HMQC spectra optimized for a 10 Hz. ^{1}H-^{13}C J coupling recorded in methanol-d_3 at 263 K.

15. SEPP-SPINEPT of ^{13}C-DMSO

Sample: [Ir] = 4.9 mM, [not labeled Pyr] = 51 mM, [^{13}C-DMSO]=20 mM, Meod-d_3 = 400 uL.

Experiment: We applied the SEPP-SPINEPT pulse sequence to detect the ^{1}H-^{13}C interactions between the hydride ligands and ^{13}C-labeled DMSO in each complex. This experiment aimed to confirm the presence of two DMSO molecules in complex **2**.

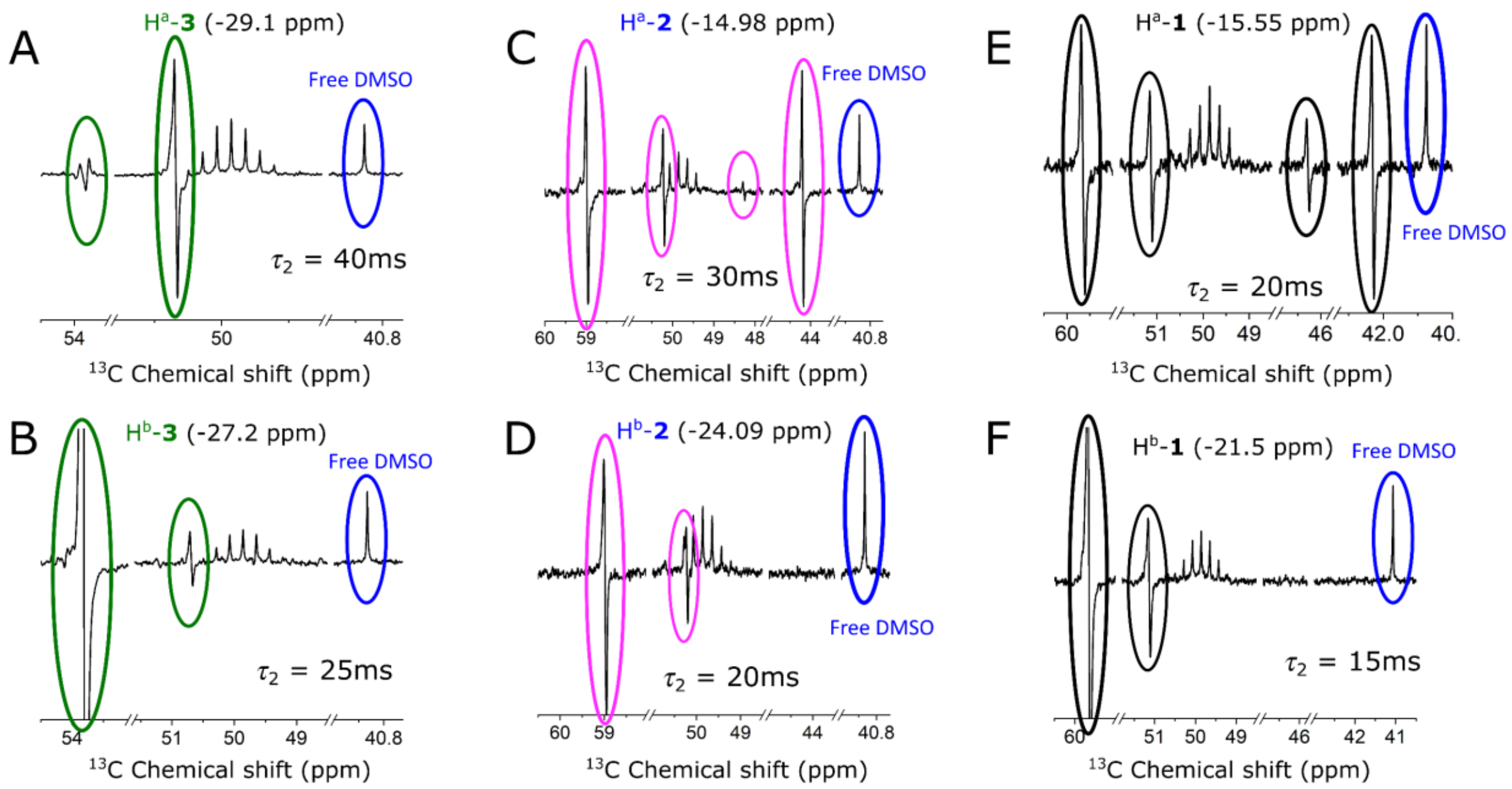

Figure S21: Phased NMR spectra at a maximum of polarization of the absolute intensity ^{13}C NMR signals of bound DMSO after SEPP-SPINEPT at 267K. (A) This NMR spectrum shows polarization transferred from H^{a}-**3** (-29.1 ppm) to the bound ^{13}C-DMSO of **3,** while (B) shows polarization transferred from H^{b}-**3** (-27.2 ppm) to the bound ^{13}C-DMSO of **3.** (C) The spectrum shows polarization transferred from H^{a}-**2** (-14.98 ppm) to the bound ^{13}C-DMSO of **2,** and (D) shows polarization transferred from H^{b}-**2** (-24.09 ppm) to the bound ^{13}C-DMSO of **2.** (E) The spectrum shows polarization transferred from H^{a}-**1** (-15.55 ppm) to the bound ^{13}C-DMSO of **1,** and (F) shows polarization transferred from H^{b}-**1** (-21.5 ppm) to the bound ^{13}C-DMSO of **1.** The peak at 41 ppm corresponds to free DMSO in the sample. The multiplet (septet) at 49.86 ppm is assigned to the methanol.

Table S17: NMR resonances of **2**. The resonance labels correspond to those shown in **Figure S19-21.**

Methanol-d_4 at 267K				
**Interaction Number**	**^{1}H (ppm)**	**^{13}C (ppm)**	**^{1}H (ppm) DMSO**	**Type of interaction**
1	-14.98	58.98	2.99	IrHH-DMSO 1
2	-14.98	50.20	3.14	IrHH-DMSO 2
3	-14.98	48.27	3.37	IrHH-DMSO 3
4	-14.98	44.19	2.91	IrHH-DMSO 4
5	-24.09	58.98	2.99	IrHH-DMSO 1
6	-24.09	50.20	3.14	IrHH-DMSO 2

16. Effect of intramolecular hydrogen exchange on SABRE

Effect of intramolecular hydrogen exchange on SLIC-SABRE efficacy: The SLIC-SABRE experiment is the most efficient for SABRE polarization of pyruvate at low field reported to date[8,9]. Neglecting pyruvate exchange at low temperatures, and considering only hydrogen exchange (as previously reported in[8]) means pH_2 exchange should occur at an optimum rate (in this case, approximately 1-10 s^{-1}), for more rapid exchange, there is insufficient time to transfer polarization, while for too slow an exchange, relaxation destroys the spin order of pH_2. Intramolecular exchange of two hydride protons will reduce the polarization level (**Figure S22**). This is because for efficient polarization transfer, the scalar couplings between pH_2 and the target must be different, and these average under exchange, so polarization transfer slows down, and efficacy drops.

The simulations were achieved using the following model. To simulate the SABRE experiment, we used the formalism developed for the linear exchange model in[8,10]. Note the critical difference: we assume that only H_2 exchanges, while the substrate stays bound to the Ir-complex. This is a typical case for **3** at low temperatures. These assumptions can be expressed with the following model chemical reaction

$$\mathrm{IrHHPyr} \xrightarrow{\mathrm{H_2\ to\ pH_2\ exchange},\ k_\mathrm{d}} \mathrm{IrH'H'Pyr}$$

$$\mathrm{IrH'HPyr} \xrightarrow{\mathrm{intramolecular\ H_2\ exchange},\ k_\mathrm{ex}} \mathrm{IrHH'Pyr}$$

where $\mathrm{IrHHPyr}$ states for the active SABRE complex and prime symbol ($'$) is used to distinguish hydrogens before and after exchange. For this chemical exchange, the following generalized Liouville-von Neuman equation has to be solved numerically:

$$\frac{d\hat{\rho}_{\mathrm{IrHHPyr}}}{dt} = \left[\hat{\hat{L}}_{\mathrm{IrHHPyr}} + k_\mathrm{ex}\left(\hat{\hat{Q}}^{\mathrm{HH}}_{\mathrm{H1\leftrightarrow H2}} - \hat{\hat{1}}_{\mathrm{IrHHPyr}}\right) + k_\mathrm{d}\left(\hat{\hat{D}}^{\mathrm{H_2^*}}_{\mathrm{IrPyr}}\widehat{\widehat{Tr}}^{\mathrm{HH}}_{\mathrm{IrHHPyr}} - \hat{\hat{1}}_{\mathrm{IrHHPyr}}\right)\right]\hat{\rho}_{\mathrm{IrHHPyr}}$$

Here $\hat{\rho}_{\mathrm{IrHHPyr}}$ is the density matrix for the active Ir-complex with H_2 and pyruvate (Iyr), $\hat{\hat{L}}$ is the corresponding Liouville superoperator, $\hat{\hat{1}}$ is a unitary superoperator, $\hat{\hat{D}}^{\mathrm{H_2^*}}_{\mathrm{IrPyr}}$ is the superoperator of the direct product that adds pH_2 to IrPyr and $\widehat{\widehat{Tr}}^{\mathrm{HH}}_{\mathrm{IrHHPyr}}$ is a superoperator that removes HH from $\mathrm{IrHHPyr}$, $\hat{\hat{Q}}^{\mathrm{HH}}_{\mathrm{H1\leftrightarrow H2}}$ is a super operator that exchanges two protons.

For example, for k_d = 10 s^{-1}, predicted polarization is falling from 67.2% to 43.7% (or by 35%) when intramolecular exchange increases from 0 to 10 s^{-1}. For 3, the $k^{\mathrm{HH}}{}_{\mathrm{3ex}}$ ranged between 2 and 7 s^{-1} in the studied temperature range.

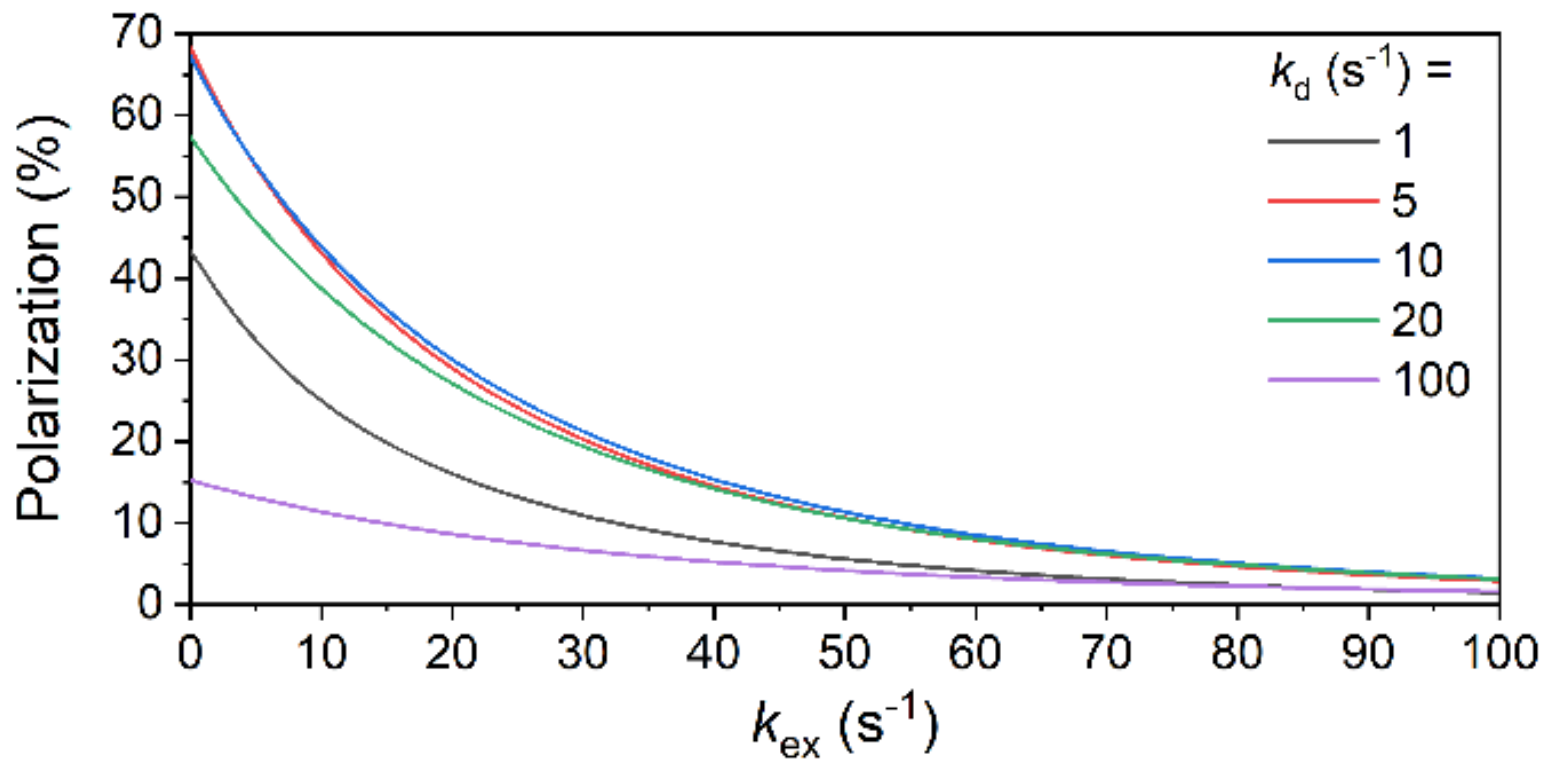

Figure S22. SLIC-SABRE [1-^{13}C]pyruvate polarization as a function of intramolecular hydrogen exchange. Intramolecular exchange is always detrimental to polarization transfer. *Simulation parameters*: three spin systems consisting of two protons and one ^{13}C resembling 1-^{13}C of pyruvate. T_1 of ^{1}H was 1 s, T_1 of ^{13}C was 25 s, chemical shifts were -27.2 ppm and -29.1 ppm for protons and 164 ppm for ^{13}C, and *J* coupling constants between protons and carbon were -0.55, 0.014 Hz, and between protons -10.48 Hz. Simulations were performed

for a 50 μT magnetic field using the SLIC pulse with an amplitude of 1 μT along the X axis in the rotating frame of reference, resonant with ^{13}C, for 60 s. Polarization along the X axis in the rotation frame of reference was generated by SLIC and is displayed here.

17. Other hyperpolarized complexes

Sample: 4 mM of [Ir-d_{22}], 300 mM of DMSO, and 40 mM ^{13}C-pyr in 600 µL of methanol-d_4.

Experiment: We used a 9.4 T NMR system at 265 K, a pH_2 pressure of 8.5 bar, and a SEPP-SABRE sequence for ^{1}H hyperpolarization of the selected proton. Set 1 - hyperpolarization of H^a-**1** at -15.56 ppm using SEPP-SABRE, and following exchanging with H^b-**1** at -21.59 ppm, and free H_2 at 4.6 ppm (**Figure S16-left**). Set 2 - hyperpolarization of H^b-**1** using SEPP-SABRE and following exchange with H^a-**1**, and H_2 (**Figure S16-right**).

Hydrogen's exchange of **1** using [DMSO] = 300 mM

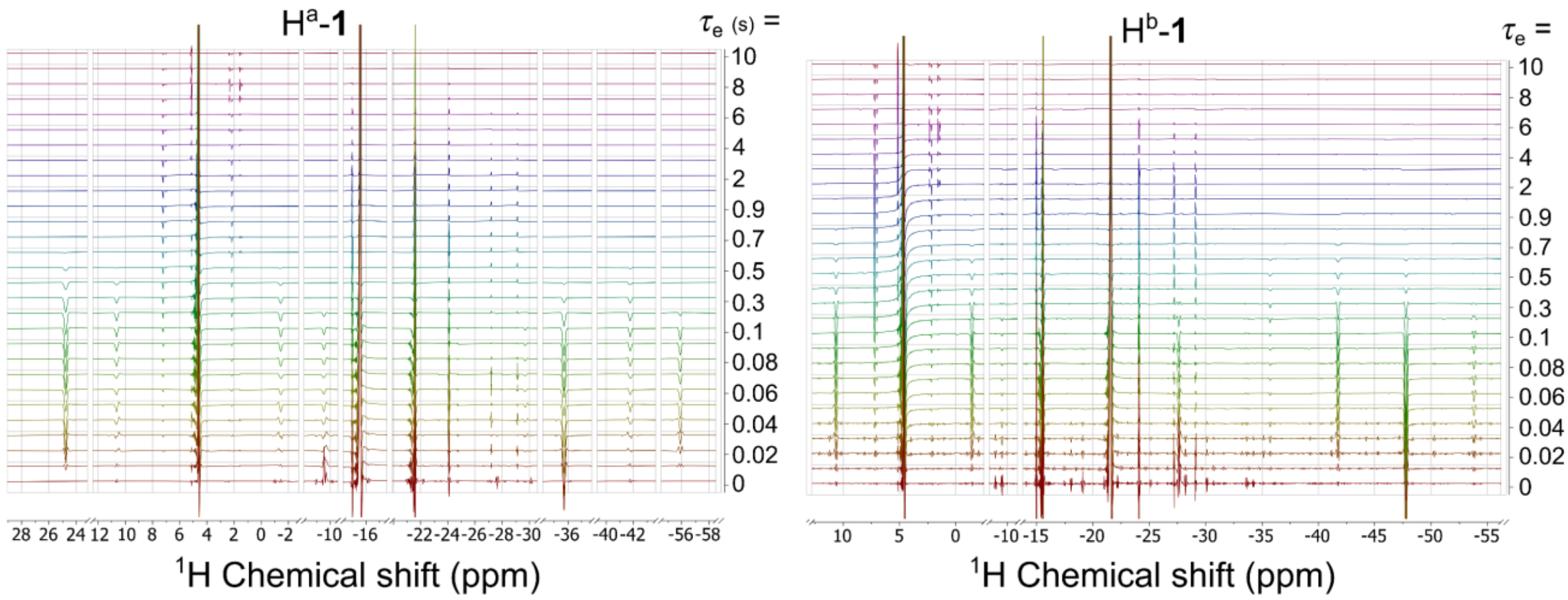

Figure S23: Observation of hydride chemical exchange of $H^{a,b}$-1. ^{1}H NMR spectra at 265 K where selective pulse excites H^a-**1** (left) and H^b-**1** (right) using SEPP-SABRE pulse sequence. During the measurement of hydrogen exchange of $H^{a,b}$-**1** in the presence of 300 mM of DMSO, an unexpected polarization transfer from hydrogens of **1** to different complexes.

18. Electronic Structure Calculations

18.1. Computational details

If not mentioned otherwise, all calculations have been carried out at the B3LYP-D4/def2-TZVP CPCM (methanol) level of theory[11–19]. Every geometry was fully optimized using tight convergence criteria and confirmed as a minimum structure via normal mode analysis. Note that while no extensive conformational searches have been carried out using refined search algorithms, several possible conformations of various structures have typically been screened before final optimization. For all calculations, the ORCA 6.1 program package has been used in[20,21]. If not stated otherwise, free energy corrections at 298.15K have been computed using the standard harmonic oscillator approximation as implemented in the thermochemistry module of ORCA[1].

Structure and Energetics of Compounds 2-4

In a first series of calculations, the structures of compounds **2** to **4** were optimized, and relative Gibbs free energies were compared with implicit solvent inclusion using CPCM. Note that energy values are given in kJ/mol.

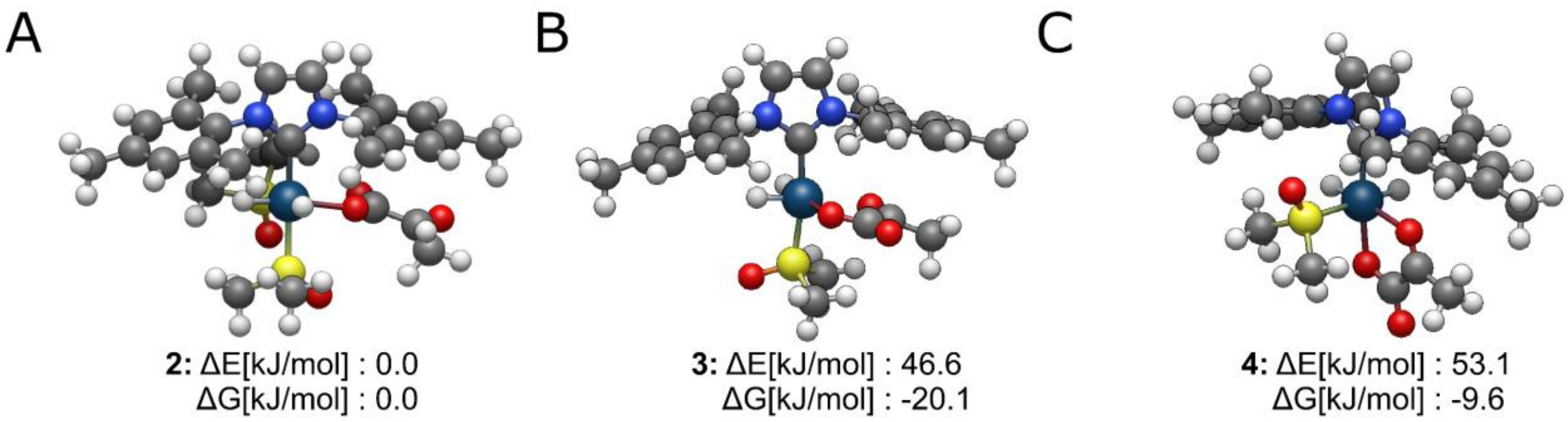

Figure S24: Optimized structures and relative free energies of complexes **2**-**4** obtained at the B3LYP-D4/def2-TZVP CPCM (methanol) level of theory. Note that for the relative energy of **2**, the free energy of DMSO has been computed at the same level of theory. For reference, free energies are shown in the table S21.

From the resulting free energies, the pyruvate complex **3** is the lowest energy structure - in contrast to what is found in experiment ($\Delta G^0_{2\leftarrow 3}$ = -5.3 kJ/mol in favor of **2**) - and the most stable compared to the other species, implying that **2** and **4** should not be observed at all (see ΔG in **Fig. S24**). For the electronic energies (**ΔE** in **Fig. S24**), however, **2,** which contains an additional DMSO, appears much more stable than **3** and **4**. This situation is often observed for Gibbs free energies of non-unimolecular reactions in solution. In the literature, it is well documented that in these cases the common entropy corrections contained in Gibbs free energies strongly overestimate the translational entropy[22]. We will refer to this computational scheme as **CS1**.

Therefore, two additional computational schemes (**CS2** and **CS3**) have been applied in computing the relative free energies of **2**, **3,** and **4,** following the suggestions of Falivene et al.[22]

CS2: We computed the $\Delta G_{50\%}$ values, in which the entropy correction for all species was scaled by 0.5[23,24].

CS3: We computed the ΔG_{assoc} values, for which the standard entropy corrections were used, but the dissociating DMSO molecule was kept associated with the reacting complex in the second coordination sphere (see Fig. S25 for geometries). For the latter, translational entropy corrections mostly cancel.

The results with respect to compound **2** are shown in **Table S18**.

Table S18: Relative free energies in kJ/mol of compounds **3** and **4** with respect to compound **2**. Three computational schemes are compared. (**CS1**) ΔG denotes standard free energy corrections. For (**CS2**) $\Delta G_{50\%}$ the entropy correction has been scaled by 0.5. (**CS3**) $\Delta G_{assoc.}$ corresponds to the relative free energies of 3 and 4 encounter complexes with DMSO (see Fig. S25).

Compound	**ΔG**	**$\Delta G_{50\%}$**	**$\Delta G_{assoc.}$**
**3**	-20.1	9.1	15.1
**4**	-9.6	15.8	23.5

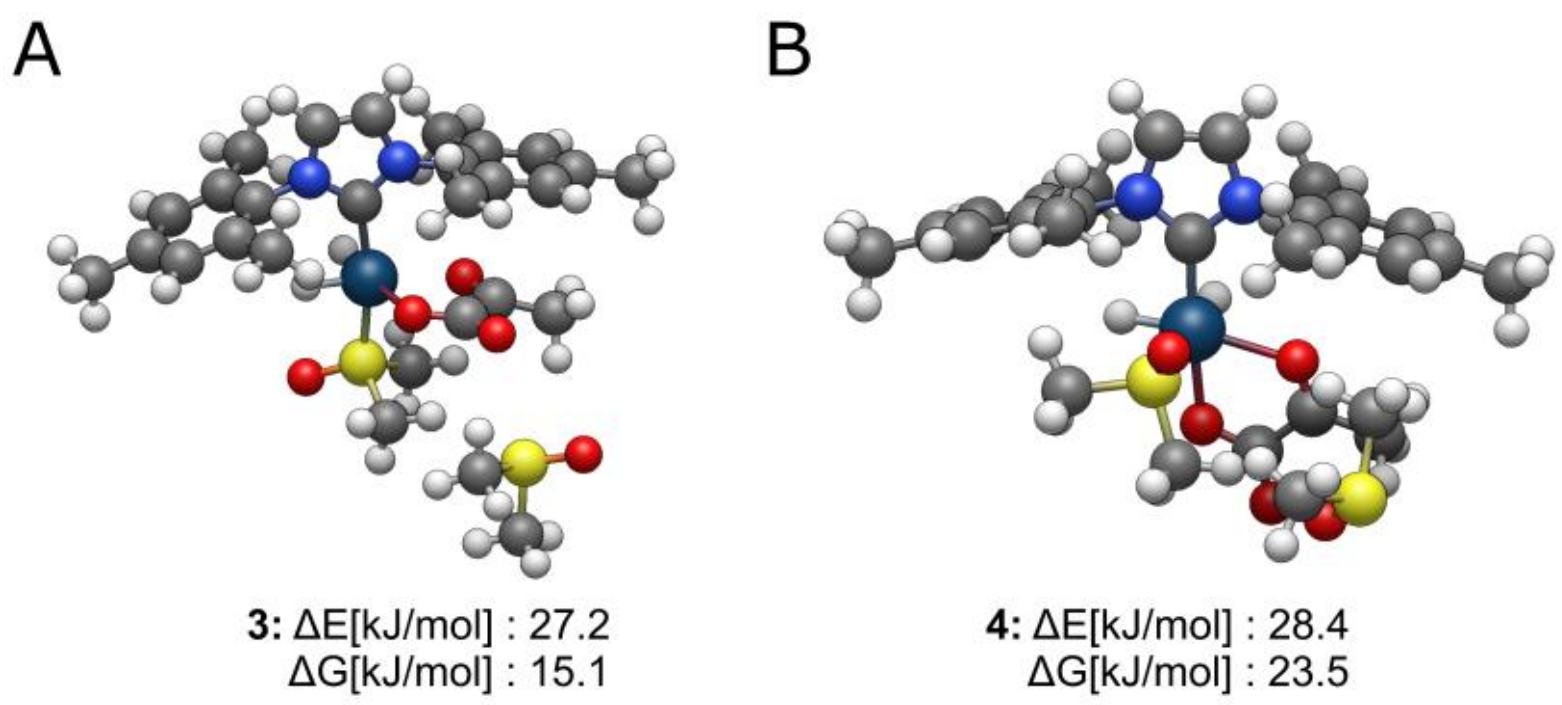

Figure S25: Geometries of compounds **3** and **4** optimized, including one weakly bound DMSO molecule in the second coordination sphere. Relative free energies and energies given in kJ/mol with respect to compound **2** (see **Fig. S24**).

As shown by the resulting Gibbs free energies (**Table S18**), the corrected values no longer favor the dissociation of DMSO from **2**. This clearly shows that the entropy corrections are a source of error in the computed results. For the discussion in the main manuscript, we have opted to report the $\Delta G_{50\%}$ values: $\Delta G_{50\%}$ and $\Delta G_{assoc.}$ clearly favor **2** over **3** by at least 9 kJ/mol, while **4** is unfavorable compared to **3** by at least 7 kJ/mol. These results are consistent with the fact that **2** is observed depending on the DMSO concentration and that it is unlikely that **4** is observed.

18.2. Additional influences on the relative stabilities of 2, 3, and 4

Experimental results indicate that, under certain conditions (for example, variation in [pyr], **Fig. 3E**), the relative concentrations of **2** and **3** can change, prompting additional computations of factors that may affect the balance between the two conformers. In this respect, it is noteworthy that sodium pyruvate is used in the experiment and has not been accounted for in calculations to date. An inspection of the electrostatic potentials (**Fig. 4**) already hints at another vital component, namely Na^+.

These electrostatic potentials suggest that the different compounds and conformers may exhibit strong stabilizing interactions with cations. The structures resemble a negatively charged pocket that facilitates explicit ion interactions. Consequently, all structures were reoptimized in the presence of Na^+, incorporating implicit solvation. As the measurements

were carried out in methanol, we assessed a variety of adducts of **2**, **3,** and **4** with Na^+, DMSO, and methanol (**Fig. S26**; the list of structures is not exhaustive).

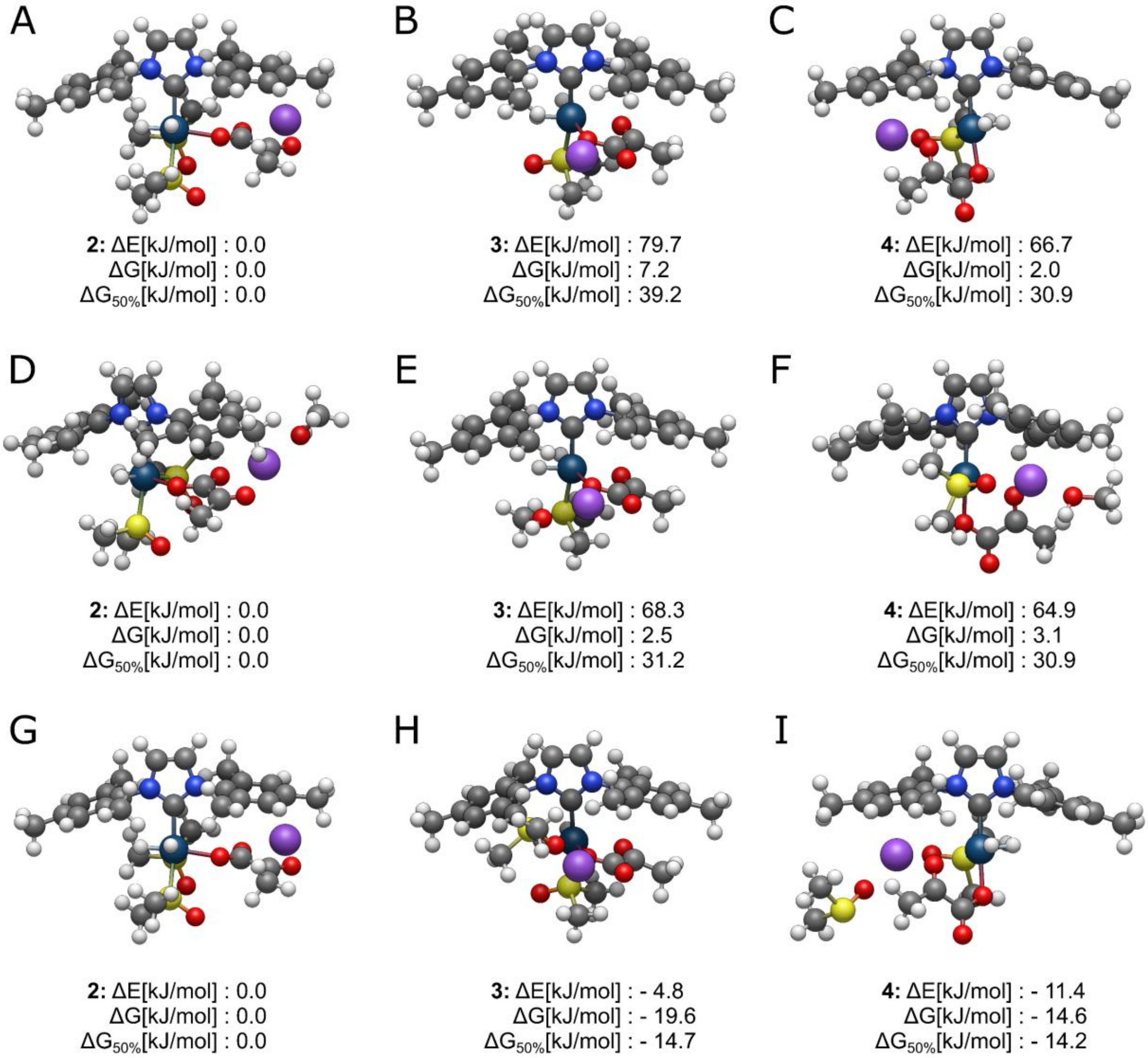

Figure S26(1): Optimized structures and relative free energies of complexes **2**-**4,** assuming sodium pyruvate yields the corresponding Na^+ adducts. Top row: (A, B, C) Na^+ adducts, middle row: (D, E, F) adducts with additional explicit solvent molecule (methanol), bottom row: (G, H, I) systems with DMSO in 2nd coordination sphere for **3** and **4**. See corresponding Lewis structures on Fig. S26(2).

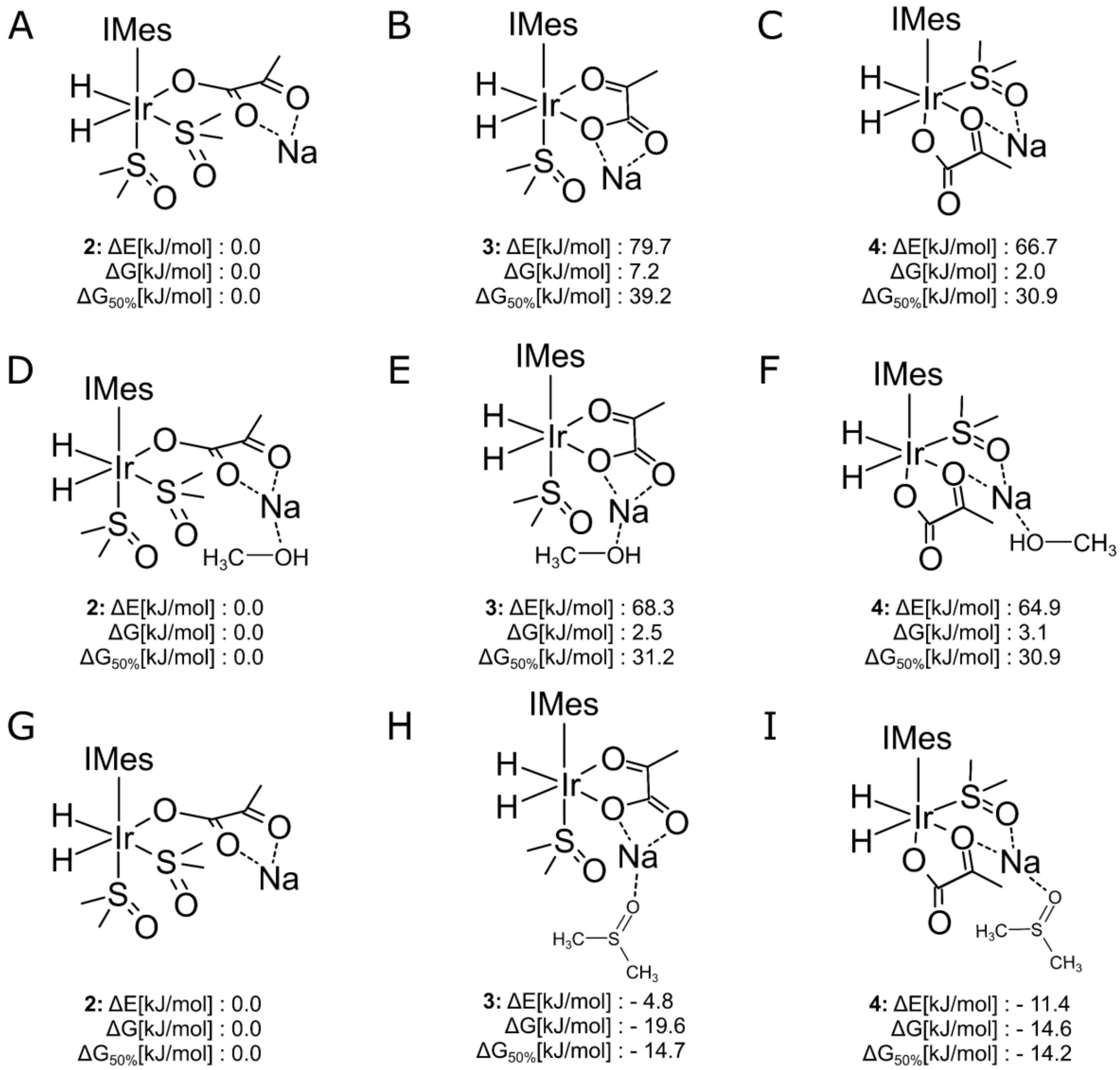

Figure S26(2): Lewis structures of the optimized structures and relative free energies of complexes **2-4,** assuming sodium pyruvate yields the corresponding Na^+ adducts. Top row: (A, B, C) Na^+ adducts, middle row: (D, E, F) adducts with additional explicit solvent molecule (methanol), bottom row: (G, H, I) systems with DMSO in 2nd coordination sphere for **3** and **4**.

While these preliminary results on a narrow set of possible structures are far from quantitative, they indicate some important influences. While without the counter ion, **2** is favorable over **3** by about 16 kJ/mol (see middle column **Table S18**), with inclusion of the counter ion (see **Figure S26** top row), the difference increases to almost 40 kJ/mol (**Fig S26** top, using $\Delta G_{50\%}$ here). For the difference between **3** and **4**, compound **3** is favorable in energy by almost 7 kJ/mol without a counterion; with Na^+, this difference is reversed to more than -8 kJ/mol in favor of **4**. Note that the presence of Na^+ also slightly changes the minimum energy structures as it perturbs the balance of electrostatic repulsion and attraction in the ligands.

However, when including further species that are present in the solvent into the model (**Fig. S26**, middle and bottom), it becomes clear that the computed trends are very model-dependent. While **3** and **4** are always predicted to be close in energy, and for the majority of models, both are less stable than **2**, a more complete description of the effect of intermolecular interactions beyond implicit solvation is required for a more quantitative analysis. Nevertheless, the results still present a strong indication that the presence of counterions actually influences the stability of different species in the equilibrium in different ways, and

might be applied to bias the system in a desired direction. Further studies are currently the focus of work in our group.

18.3. Formation energy of complex 2 from complex 1

From the experimental results, it can be assumed that **2** is formed from **1** after pyruvate dissociation and Cl addition. However, as their stoichiometries differ by the Cl^- anion (and DMSO, which, however, is neutral), computed relative energies are much less reliable, as the common solvent models do not describe ions that undergo ion pair interactions very accurately. Note that, like in the previous section, this section is meant to assess possible influences and sources of error in the computational numbers, rather than derive accurate numbers.

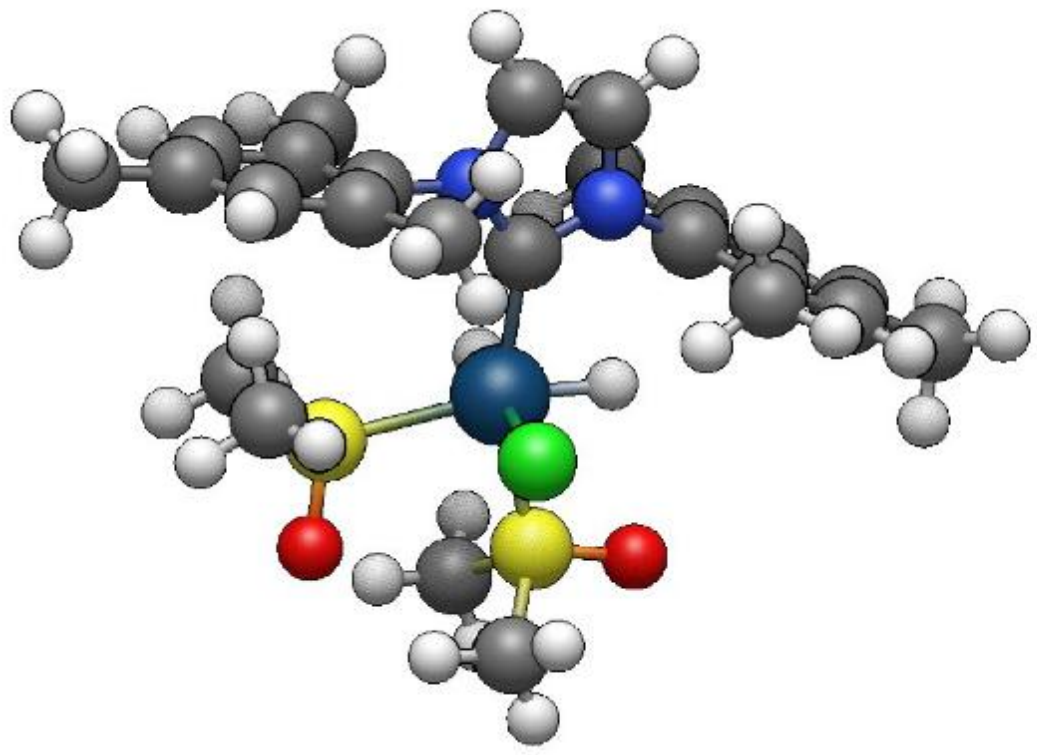

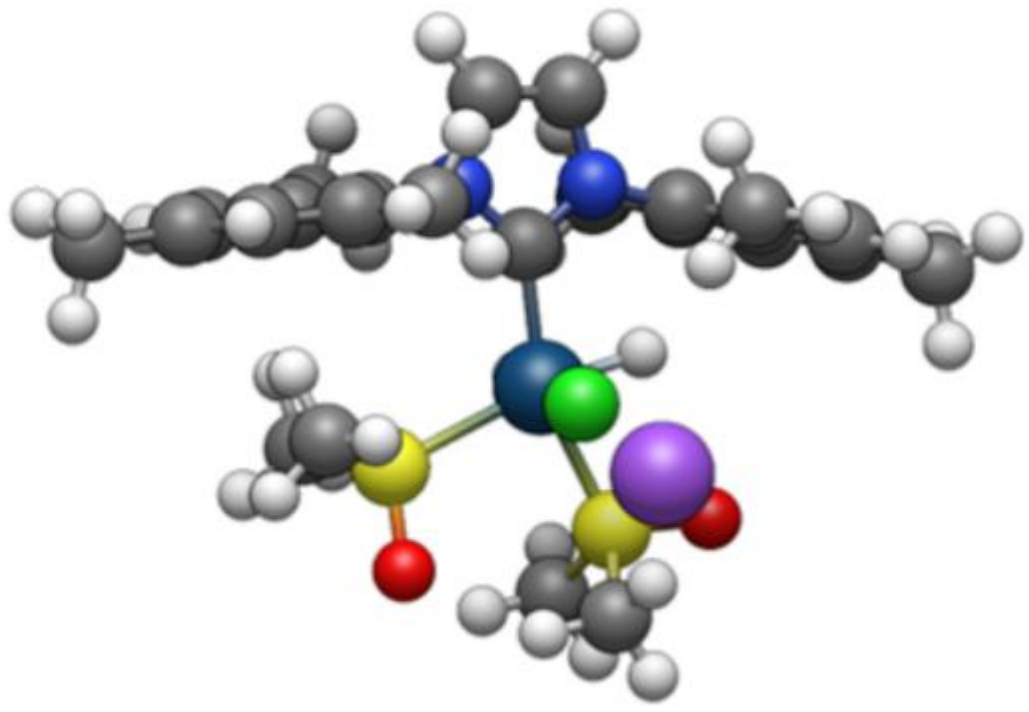

Figure S27: Optimized structures of complex **1** without (left), and with (right) Na^+ counterion.

To do so, we have computed the relative free energy differences using the computed energies (including CPCM for methanol) of sodium pyruvate, the chloride anion, the sodium cation, a sodium chloride ion pair, and the Na^+ educts (after preliminary screening of low energy conformers) of **1** and **3**, e.g. ΔG(**3**←**1**) = ΔG(**3**) - ΔG(**1**), at 298.15 K **(Table S19**), where **1** = $[IrIMes(DMSO)_2(HH)Cl]$ and **3** = [IrIMesDMSO(pyr)(HH)]. From these numbers, it becomes obvious that a broad range of relative energies is obtained depending on how the reference energies are chosen.

In the extreme case of using Na^+ and pyr^- for the educts and NaCl for the product side, **3** is more stable by 150 kJ/mol. If ions as reference systems are avoided, the relative energies change in favor of the educt side (like for [**3**+DMSO+NaCl ← [1+(Na pyr)]]), while the presence of an ion pair of **1** with Na^+ fully favors the educt side (as for [(**3** Na^+)+DMSO+NaCl] ← [(**1** Na^+)+(Na pyr)]). Hence, reliable predictions for the relative free energies of **1** can only be made if more information about the final state of the ions can be obtained. This might include anything from precipitation of NaCl to strong interactions with other ions in solution.

Table S19: Relative free energy differences (ΔG, 298.15 K) for the interconversion between complexes **1** and **3** under different ionic environments.

	$\Delta G^{298.15}$ (kJ/mol)
[**3**+DMSO+Cl^-] ← [**1**+pyr^-]	21.8
[**3**+DMSO+NaCl] ← [**1**+(Na pyr)]	-2.0
[**3**+DMSO+NaCl] ← [**1**+pyr^-+Na^+]	-150.7
[(**3** Na^+)+DMSO+NaCl] ← [(**1** Na^+)+(Na pyr)]	51.0

[(**3** Na^+)+DMSO+Cl^-] ← [(**1** Na^+)+pyr^-]	74.9
[(**3** Na^+)+DMSO+Cl^-] ← [**1**+(Na pyr)]	53.6
[(**3** Na^+)+DMSO+NaCl] ← [**1**+(Na pyr)+Na^+]	-118.9

18.4. Geometric parameters for 3

The structure of **3** calculated with B3LYP-D4/def2-TZVP CPCM(methanol) agrees with the structures and properties reported in Ref.[25] obtained at the ω-B97XD(PCM) / LANL2TZ(Ir) 6-311G(d.p) level of theory (**Fig. S28**).

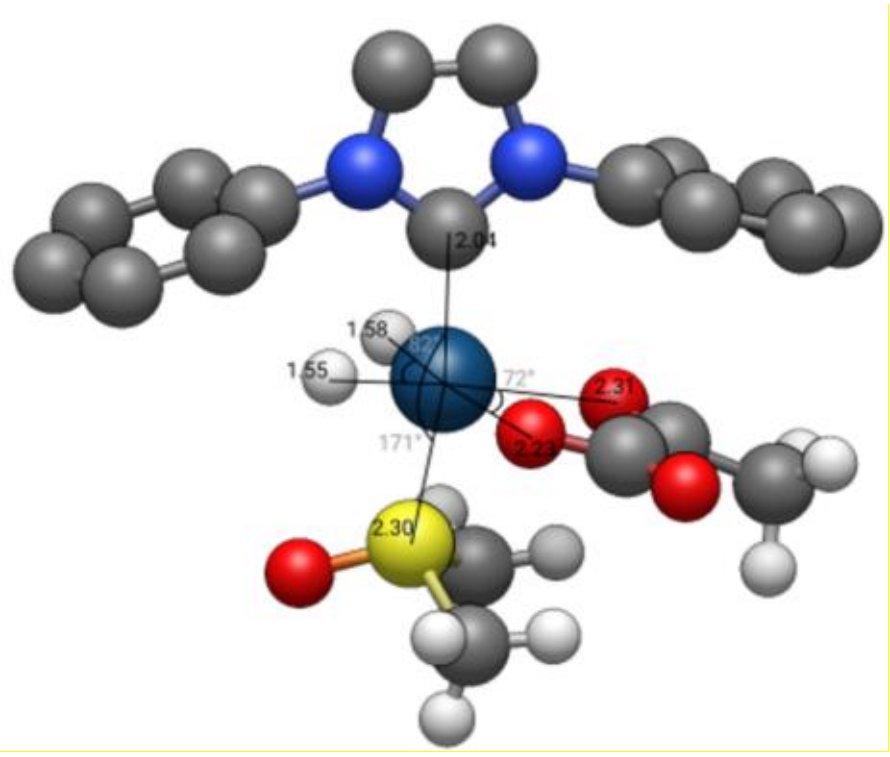

Figure S28: Geometrical parameters of **3** without the addition of Na^+ computed at the B3LYP-D4/def2-TZVP CPCM(methanol) level of theory.

18.5. Further DFT functional benchmarks

To assess the error introduced by DFT, the energies and energy difference between compounds **3** and **4** have been computed using single-point energy calculations using PBE, PBE0, TPSS, ωB97x, and B3LYP functionals [26–29] and B3LYP-D4/def2-SVP CPCM (methanol) optimized geometries with D4 dispersion correction and implicit solvation (**Table S20**, upper part).

The results indicate that, for these systems, typical DFT errors of 5 kJ/mol are observed, ensuring that the relative energies of different species are reproduced well, regardless of the functional chosen.

Comparing single-point calculations at the B3LYP-D4/def2-TZVP CPCM(methanol) geometry to the relative energies obtained from full optimizations using DFT-D4/def2-TZVP CPCM(methanol) (DFT = PBE, PBE0, TPSS, ωB97x, and B3LYP) shows that deviations are mostly due to the functional in the energy calculations and not so much due to differences in the structures (**Table S20**, lower part). Furthermore, B3LYP appears to accurately represent the range of results obtained. Also, the trend that **4** is always above **3** in energy is robust throughout the different results.

Table S20: Single-point energies (absolute electronic energies [a.u.]) and energy differences (ΔE[kJ/mol]) between compounds **3** and **4** calculated using different DFT functionals with B3LYP geometry (upper part, see text) and geometries optimized using the different functionals (lower part).

	PBE	PBE0	TPSS	WB97X	B3LYP
Geometry: B3LYP-D4/def2-TZVP CPCM					

Single point energy: DFT-D4/def2-TZVP CPCM					
E(3)	-1923.701306930	-1923.77022309	-1925.879486509	-1925.146623401	-1924.707791733
E(4)	-1923.699563319	-1923.76765087	-1925.877799114	-1925.143430419	-1924.705312465
ΔE [kJ/mol]	4.6	6.8	4.4	8.4	6.5
Geometries optimized at the corresponding DFT-D4/def2-TZVP CPCM level of theory					
E(3)	-1923.703282727	-1923.76936534	-1925.880269513	-1925.144541356	-1924.707791733
E(4)	-1923.704852988	-1923.77189298	-1925.881960145	-1925.147787817	-1924.705312465
ΔE [kJ/mol]	4.1	6.6	4.4	8.5	6.5

Relaxed H-H distance potential energy curve scan

To assess the intramolecular dynamics of the hydrogens in the system, a relaxed surface scan for the H-H bond distance was carried out for **3** (see **Figure S29**). Note that the equilibrium distance between the hydrogen atoms is 2.05 Å. From this plot, it becomes evident that the potential of the H atoms moving in is, as expected, very shallow. Within 5-10 kJ/mol distance changes of almost 0.3 Å are possible, indicating a strong quantum nature in the dynamics of the hydrogen degrees of freedom.

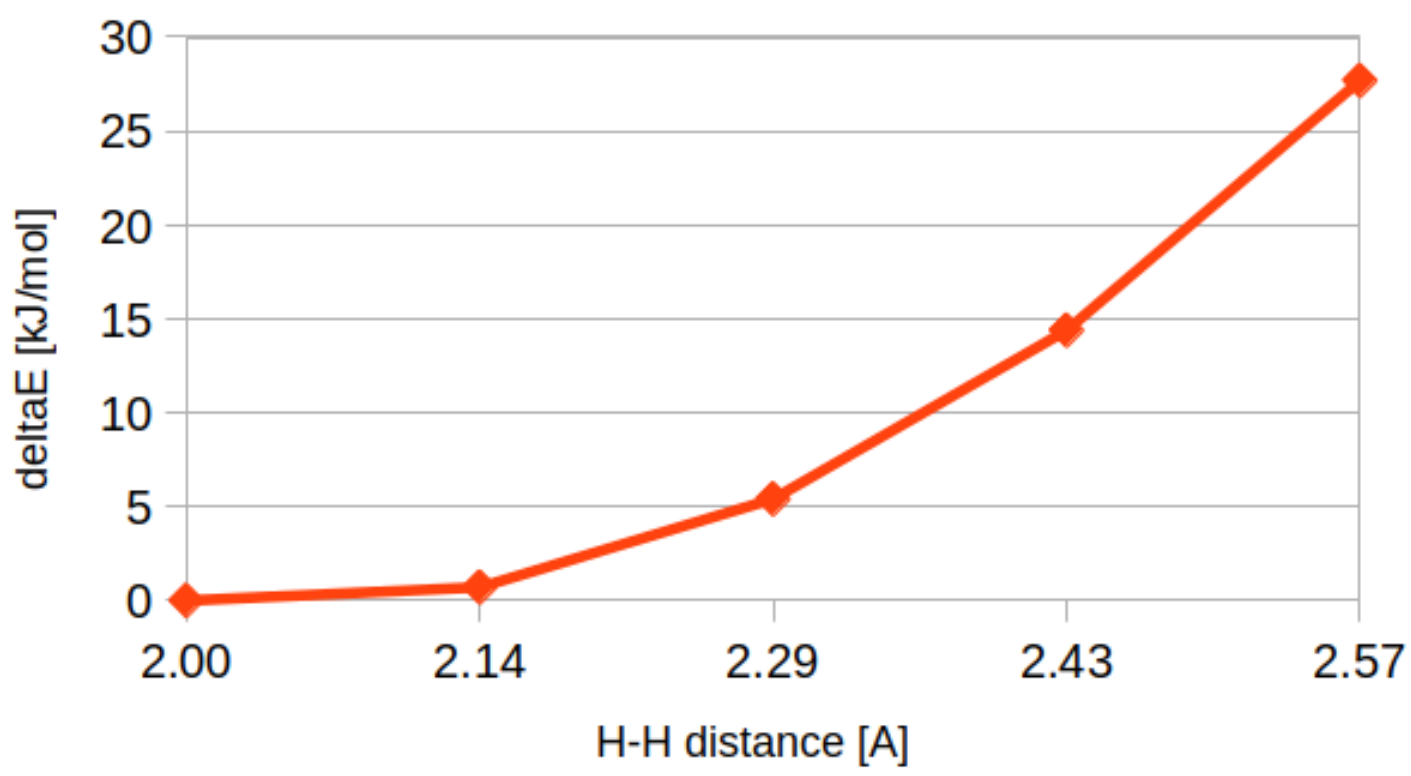

Figure S29: Relaxed H-H distance scan at the B3LYP-D4/def2-SVP CPCM(methanol) level of theory.

Sample input files

Standard DFT geometry optimization and frequency calculation:

```
!B3LYP D4 RIJCOSX def2-TZVP def2/J CPCM(Methanol) TIGHTOPT FREQ
```

Reference energies

For all relative Gibbs free energy calculations, the following reference systems have been used:

Table S21: Reference systems for all relative Gibbs free energy calculations.

	G [a.u.]
H_2	-1.17554706
DMSO	-553.12635508
MeOH	-115.69137577
pyruvate anion	-341.91993197
pyruvate protonated	-342.36424947
chloride anion	-460.336856781981
water	-76.43352387
sodium chloride (Na^+Cl^-) model	-622.55046007
sodium pyruvate	-504.1244614

sodium cation	-162.147901128498

Note: XYZ coordinates of selected compounds are given as a separate zip file as part of the Zenodo DOI: https://doi.org/10.5281/zenodo.18449765.

19. Supplementary References

(1) McConnell, H. M. Reaction Rates by Nuclear Magnetic Resonance. *The Journal of Chemical Physics* **1958**, *28* (3), 430–431. https://doi.org/10.1063/1.1744152.

(2) Schmidt, A. B.; Wörner, J.; Pravdivtsev, A.; Knecht, S.; Scherer, H.; Weber, S.; Hennig, J.; Von Elverfeldt, D.; Hövener, J. Lifetime of *Para* Hydrogen in Aqueous Solutions and Human Blood. *ChemPhysChem* **2019**, *20* (19), 2408–2412. https://doi.org/10.1002/cphc.201900670.

(3) Pravdivtsev, A. N.; Kozinenko, V. P.; Hövener, J.-B. Only Para-Hydrogen Spectroscopy (OPSY) Revisited: In-Phase Spectra for Chemical Analysis and Imaging. *J. Phys. Chem. A* **2018**, *122* (45), 8948–8956. https://doi.org/10.1021/acs.jpca.8b07459.

(4) Cowley, M. J.; Adams, R. W.; Atkinson, K. D.; Cockett, M. C. R.; Duckett, S. B.; Green, G. G. R.; Lohman, J. A. B.; Kerssebaum, R.; Kilgour, D.; Mewis, R. E. Iridium N-Heterocyclic Carbene Complexes as Efficient Catalysts for Magnetization Transfer from *Para* -Hydrogen. *J. Am. Chem. Soc.* **2011**, *133* (16), 6134–6137. https://doi.org/10.1021/ja200299u.

(5) Kessler, H.; Oschkinat, H.; Griesinger, C.; Bermel, W. Transformation of Homonuclear Two-Dimensional NMR Techniques into One-Dimensional Techniques Using Gaussian Pulses. *Journal of Magnetic Resonance (1969)* **1986**, *70* (1), 106–133. https://doi.org/10.1016/0022-2364(86)90366-5.

(6) Bauer, C.; Freeman, R.; Frenkiel, T.; Keeler, J.; Shaka, A. J. Gaussian Pulses. *Journal of Magnetic Resonance (1969)* **1984**, *58* (3), 442–457. https://doi.org/10.1016/0022-2364(84)90148-3.

(7) Assaf, C. D.; Gui, X.; Auer, A. A.; Duckett, S. B.; Hövener, J.-B.; Pravdivtsev, A. N. J Coupling Constants of <1 Hz Enable 13C Hyperpolarization of Pyruvate via Reversible Exchange of Parahydrogen. *J. Phys. Chem. Lett.* **2024**, *15* (5), 1195–1203. https://doi.org/10.1021/acs.jpclett.3c02980.

(8) Pravdivtsev, A. N.; Buckenmaier, K.; Kempf, N.; Stevanato, G.; Scheffler, K.; Engelmann, J.; Plaumann, M.; Koerber, R.; Hövener, J.-B.; Theis, T. LIGHT-SABRE Hyperpolarizes 1-^{13} C-Pyruvate Continuously without Magnetic Field Cycling. *J. Phys. Chem. C* **2023**, *127* (14), 6744–6753. https://doi.org/10.1021/acs.jpcc.3c01128.

(9) Schmidt, A. B.; Eills, J.; Dagys, L.; Gierse, M.; Keim, M.; Lucas, S.; Bock, M.; Schwartz, I.; Zaitsev, M.; Chekmenev, E. Y.; Knecht, S. *Over 20% 13C Hyperpolarization for Pyruvate Using Deuteration and Rapid SLIC-SABRE in Mictrotesla Fields*; preprint; Chemistry, 2023. 10.1021/acs.jpclett.3c00707.

(10) Knecht, S.; Pravdivtsev, A. N.; Hövener, J.-B.; Yurkovskaya, A. V.; Ivanov, K. L. Quantitative Description of the SABRE Process: Rigorous Consideration of Spin Dynamics and Chemical Exchange. *RSC Adv.* **2016**, *6* (29), 24470–24477. https://doi.org/10.1039/C5RA28059A.

(11) Becke, A. D. A New Mixing of Hartree–Fock and Local Density-Functional Theories. *The Journal of Chemical Physics* **1993**, *98* (2), 1372–1377. https://doi.org/10.1063/1.464304.

(12) Weigend, F. Accurate Coulomb-Fitting Basis Sets for H to Rn. *Phys. Chem. Chem. Phys.* **2006**, *8* (9), 1057. https://doi.org/10.1039/b515623h.

(13) Izsák, R.; Neese, F. An Overlap Fitted Chain of Spheres Exchange Method. *The Journal of Chemical Physics* **2011**, *135* (14). https://doi.org/10.1063/1.3646921.

(14) Weigend, F.; Ahlrichs, R. Balanced Basis Sets of Split Valence, Triple Zeta Valence and Quadruple Zeta Valence Quality for H to Rn: Design and Assessment of Accuracy. *Phys. Chem. Chem. Phys.* **2005**, *7* (18), 3297. https://doi.org/10.1039/b508541a.

(15) Lee, C.; Yang, W.; Parr, R. G. Development of the Colle-Salvetti Correlation-Energy Formula into a Functional of the Electron Density. *Phys. Rev. B* **1988**, *37* (2), 785–789. https://doi.org/10.1103/PhysRevB.37.785.

(16) Garcia-Ratés, M.; Neese, F. Effect of the Solute Cavity on the Solvation Energy and Its Derivatives within the Framework of the Gaussian Charge Scheme. *J Comput Chem* **2020**, *41* (9), 922–939. https://doi.org/10.1002/jcc.26139.

(17) Garcia-Ratés, M.; Neese, F. Efficient Implementation of the Analytical Second Derivatives of Hartree–Fock and Hybrid DFT Energies within the Framework of the Conductor-like Polarizable Continuum Model. *J Comput Chem* **2019**, *40* (20), 1816–1828. https://doi.org/10.1002/jcc.25833.

(18) Andrae, D.; Häußermann, U.; Dolg, M.; Stoll, H.; Preuß, H. Energy-Adjustedab Initio Pseudopotentials for the Second and Third Row Transition Elements. *Theoret. Chim. Acta* **1990**, *77* (2), 123–141. https://doi.org/10.1007/bf01114537.

(19) Caldeweyher, E.; Bannwarth, C.; Grimme, S. Extension of the D3 Dispersion Coefficient Model. *J. Chem. Phys.* **2017**, *147* (3), 034112. https://doi.org/10.1063/1.4993215.

(20) Neese, F. Software Update: The ORCA Program System—Version 6.0. *WIREs Comput Mol Sci* **2025**, *15* (2). https://doi.org/10.1002/wcms.70019.

(21) Neese, F. The ORCA Program System. *WIREs Comput Mol Sci* **2012**, *2* (1), 73–78. https://doi.org/10.1002/wcms.81.

(22) Falivene, L.; Barone, V.; Talarico, G. Unraveling the Role of Entropy in Tuning Unimolecular vs. Bimolecular Reaction Rates: The Case of Olefin Polymerization Catalyzed by Transition Metals. *Molecular Catalysis* **2018**, *452*, 138–144. https://doi.org/10.1016/j.mcat.2018.04.012.

(23) Garza, A. J. Solvation Entropy Made Simple. *J. Chem. Theory Comput.* **2019**, *15* (5), 3204–3214. https://doi.org/10.1021/acs.jctc.9b00214.

(24) Yu, Z.-X.; Houk, K. N. Intramolecular 1,3-Dipolar Ene Reactions of Nitrile Oxides Occur by Stepwise 1,1-Cycloaddition/Retro-Ene Mechanisms. *J. Am. Chem. Soc.* **2003**, *125* (45), 13825–13830. https://doi.org/10.1021/ja0376487.

(25) Mascitti, B. B.; Zanoni, G.; De Biasi, F.; Rastrelli, F.; Saielli, G. Predicting the NMR Chemical Shifts of Hydrides in SABRE-Active Ir Complexes by Relativistic DFT. *Phys. Chem. Chem. Phys.* **2025**. https://doi.org/10.1039/d5cp01214g.

(26) Perdew, J. P.; Chevary, J. A.; Vosko, S. H.; Jackson, K. A.; Pederson, M. R.; Singh, D. J.; Fiolhais, C. Atoms, Molecules, Solids, and Surfaces: Applications of the Generalized Gradient Approximation for Exchange and Correlation. *Phys. Rev. B* **1992**, *46* (11), 6671–6687. https://doi.org/10.1103/physrevb.46.6671.

(27) Perdew, J. P.; Burke, K.; Ernzerhof, M. Generalized Gradient Approximation Made Simple. *Phys. Rev. Lett.* **1996**, *77* (18), 3865–3868. https://doi.org/10.1103/PhysRevLett.77.3865.

(28) Perdew, J. P.; Ernzerhof, M.; Burke, K. Rationale for Mixing Exact Exchange with Density Functional Approximations. *The Journal of Chemical Physics* **1996**, *105* (22), 9982–9985. https://doi.org/10.1063/1.472933.

(29) Chai, J.-D.; Head-Gordon, M. Long-Range Corrected Hybrid Density Functionals with Damped Atom–Atom Dispersion Corrections. *Phys. Chem. Chem. Phys.* **2008**, *10* (44), 6615. https://doi.org/10.1039/b810189b.
