# Supplementary material for: Mechanistic insights into chemical exchange during the signal amplification by reversible exchange sensitization of pyruvate
Source: Nat Commun. 2026 Jun 25;17:7980. doi: 10.1038/s41467-026-74502-1 (PMC13448611; doi:10.1038/s41467-026-74502-1)
Supplement: Supplementary file 2 [file 41467_2026_74502_MOESM2_ESM.pdf]

nature portfolio

Peer Review File

Mechanistic Insights into Chemical Exchange during the Signal Amplification by Reversible Exchange Sensitization of Pyruvate

Corresponding Author: Dr Andrey Pravdivtsev

This file contains all reviewer reports in order by version, followed by all author rebuttals in order by version.

Attachments originally included by the reviewers as part of their assessment can be found at the end of this file.

Version 0:

Reviewer comments:

Reviewer #1

(Remarks to the Author)
Assaf et al present an investigation of the reaction pathways of the iridium catalyst during the preparation of 13C-hyperpolarized [1-13C]-pyruvate using parahydrogen during SABRE, which is a cost-effective method for enhancing in vivo metabolic MRI. They question the accepted assignment of the 1H hydride signals of the complex and propose a new assignment. Based on this assignment, they monitor in detail the conversion of the complex, including intramolecular exchange. To do so, they introduce a new NMR-based method to monitor specific pathways. Their results suggest that the complex which was thought to be necessary for SABRE might in fact be detrimental for SABRE efficiency. They conclude on the possible implications of their results for the future optimization of SABRE.

While the results are interesting and certainly useful for the future optimization of SABRE, the weakness of the paper is that it does not give a clear account of what is the state of the art and the established understanding of the SABRE mechanism vs the methods introduced by the authors and the mechanism they propose.

The results are convincing, but in some places, they feel like a collection of observations rather than like a compelling story following a logical course. It makes it hard to follow and not suitable for a large audience. Furthermore, in the current state, the manuscript shows how this study advances the understanding of SABRE hyperpolarization but the impact for a broader readership, as required by Nature Communications, is not clear.

I believe the manuscript could be suitable for Nature Communications after major revisions to make it more accessible and easier to read and to highlight how it advances science in a broader way than only for the parahydrogen community.

Here are some examples where I felt a need for more clarity in what is established vs new.
• The abstract states “we introduce a parahydrogen-enhanced, spin-selective NMR method to investigate pyruvate binding”, but the introduction does not present what methods were previously established and what is the novelty of the introduced methods.
• Paragraph starting with “Given the importance of this area, this paper reports […]”: This paragraph which summarizes the main findings of the paper could be clearer. The sentence "It was found that the NMR signals proposed to be associated with 4 [...]" is a surprising way to start the description of the results. As reader, I want to hear what methods were used/what new methods were introduced, and the key findings they allowed to reach. In the current state, things feel are mixed and blurred.
• First section of results and discussion: it is not clear from reading the text if the scheme in Figure 1 exists in the literature or is proposed by the authors. The authors should clarify it and, if it exists in the literature, references must be added.
• I have nothing against the use of the passive form in general but here it often makes it unclear what are previously established findings/hypotheses and findings/hypotheses of the papers.

This manuscript is presumably meant for a large audience as it is submitted to Nat. Comms. However, the authors mention specialized NMR experiments without even specifying the acronyms. If the paper is to be published in Nat. Comms., the

acronyms must be detailed. Furthermore, a few words explaining what information each experiment brings would make the paper much more approachable (in particular for EXSY).

Detailed comments

Introduction

- “Has proven” appears three times in the intro and feels heavy
- Because the introduction focuses precisely on the production of hyperpolarized pyruvate for in vivo MRI, a few words comparing the performance (polarization, concentration, purity, preparation time) of the different methods (dDNP, PHIP, SABRE) would be welcome.
- “However, the hyperpolarization community widely aspires to create swifter and more cost-effective alternatives to hyperpolarize biomolecules using parahydrogen” This statement sounds a little overstretched. Parahydrogen is not the only way the hyperpolarization community seeks to make the hyperpolarization of biomolecules more widespread or faster. This statement should be toned down, for example saying “one route is SABRE…”
- “In this case, the measurement of ligand loss rates for the dissociative exchange pathway that SABRE catalysts like $[Ir(H)_2(IMes)(pyridine)_3]Cl$ follow is standard[28–30].” By "standard", do the authors mean that it's easy/routinely done?
- “[…] as this process typically happens after substrate loss and is complicated by the role a dihydrogen-dihydride complex plays in the exchange mechanism[28,31].” polarization or molecular exchange?
- “[…] which is proposed to exist as two isomers where pyruvate binds in the equatorial plane (3) or bridges equatorial-axial sites (4)” Would be good to make it clear in which reference this hypothesis was proposed. 39? 21?
- “Previous DFT predictions indicated a 7.6 kJ/mol higher Gibbs free energy for 4 than for 3, which is higher than the experimentally observed equilibrium would suggest.” This argument is difficult to follow as is: equilibrium between which states?
- “[…] $[Ir(H)_2(IMes)(Cl)(\kappa 1\text{-pyr})(DMSO)_2]$, (2), forms instead of 4 (Fig. 1), as previously assumed.” A reference is needed

Results

Chemical exchange models. Several Key features of the pyruvate SABRE system:

- “Several” in the title is superfluous
- “Hence, dissociative loss of DMSO and chloride in 1 and 2 (Fig. 1) is likely.” I understand that the authors are transposing a well-established mechanism of pyridyl-type systems to the pyruvate case. If correct, this could be made clearer.
- “A role for complex 4 was ruled out through detailed experimental and theoretical evidence, as described later.” This is an example where the passive form gets in the way of understanding. Furthermore, the authors are already ruling out a mechanism at the point where they are setting up the work hypothesis.
- Paragraph staring with “The corresponding bimolecular ligand rebinding is indicated […]” This paragraph is confusing: it states that figure 2 shows some rebinding steps but then each listed element is specified "not shown".
- “The required associative bimolecular H_2 loss […]” Required for what?
- “To evaluate the merits of this proposed model, a series of experimental EXSY-type measurements […]” This section presents the proposed mechanisms, but this part of the paragraph mentions the experiments undertaken to study of the mechanism. This makes the story hard to follow.
- Figure 2: the arrows in the conversion from and to 1 shows +pyr and -pyr by pyruvate isn't present in the product and reagent
- Figure 2: The arrows leading to H_2 (from 2 and 3) do not follow the same logic as the rest of the figure. Instead of linking complexes, they link a complex to H_2 and the complex disappears. Although I think I understand the point the authors are making, I find this is a little confusing.

Reevaluation of the SABRE complex structures and NMR signal assignment

- “Previously, the ^1H chemical shifts of (here) complex 2 were associated with com-plex 4 instead[26,40,43–46].” Hard to follow. What signals? I suppose the authors should refer to Fig 3a. Then the rest of the paragraph mixes established facts that the authors challenge, conclusions drawn by the authors, and some results. The structure should be clearer. What is most troubling is that conclusions are drawn before the results are even stated. Then the first results that are mentioned are not even in the main text but it the SI.
- “Among others, we used NOE of two hydride ligands that […]” We are finally getting to actual results. However, they are mentioned in an intro-like fashion, with no mention of a figure. Regarding NOE, was this H-H NOE? How was the NOE experiment used to draw the authors’ conclusion. Looking at Figure S19 (and not S20), I understand that the presence of a cross-peak in the ^1H-^1H-NOE spectrum led the authors to their conclusion. This should be clearly stated.
- “At the same time, connections between the hydrides and Me groups of the IMes ligand were also ex-pected.” And so what? Expected by who? This sentence and the rest of the paragraph are unclear.
- “The hydride connections to DMSO signals at 3.37 ppm, 2.98 ppm, and 2.92 ppm […]”. The hydride connections of what complex?

Temperature dependency

- Why are the authors undertaking this measurement series? The section should start with a direction. Otherwise, it feels like an internal report-like list of observations. This applies to other sections.

DMSO concentration dependency

- “[…] 1, 3, and 4 are considered to be the pri-mary complexes in SABRE, […]” I don't understand the word "primary" in this context, but it might be a lack of knowledge of mine

- “This data highlights why polarization of high [pyr] could be a challenge.” is or could be? Are the others referring to a known challenge? If so, they should add a references

Computational study
- “[…] in agreement with our findings” The section on T dependence gives value for 2<-3 and 1<-3, while the DFT results are for 2<-3 and 2<-4. I don't understand the comparison that is made between DFT and experimental results. A table would help.
- “In addition to this fact, SABRE works only with sodium pyruvate and not with pyruvic acid, which is another clear indication of the critical role Na+-pyr--pairing plays in complex stabilization.” Add reference to literature or data or specify "not shown"

Hyperpolarization-enhanced observation of proton exchange
- “We acknowledge that a Thrippleton-Keeler zero-quantum filter based on a chirp pulse with a gradient […]” Do I understand correctly that Fig 5A shows a pulse sequence that was not used? That's a surprising choice. I think it would be more appropriate to show in the figure the exact pulse sequence that used.
- “It is worth noting some limitations of this approach. As with all EXSY-like experiments, one cannot distin-guish between NOE cross-relaxation and chemical exchange;” It is positive that the authors state this limitation. However, they should probably discuss how this affects the interpretation of the data (and possibly refer the reader to the SI).

Hydrogen site intramolecular exchange
- “readily evident in all of these NMR spectra” What are “these”?
- “The fact that we always observed uniform conversion from, e.g., Ha excited state to Ha and Hb of the same complex 3 or complex 2 indicates that kHHex flux >>[DMSO]*k2+DMSO, and, k3-κ1-κ2-pyr.” This sentence is very hard to follow

Hydrogen-observed intramolecular exchange in 2
- “we observed the polarization buildup of the negative sign” Polarization buildup of what?

Reviewer #2

(Remarks to the Author)
Assaf and colleagues describe a study of the kinetics of iridium complex formation with hydrogen and pyruvate used in SABRE hyperpolarization of 13C-labelled pyruvate. With extensive characterization of the dependence on temperature and concentration and of the interconversion of the various complex complexes, this study sheds fresh light on the mechanisms of this promising hyperpolarization technology and points to potential routes for its improvement.

Overall, the manuscript is well written and clearly presented. My main concern is that the data presented consists of many details that, while very important for the SABRE community, may be lost on the general readership of Nature Communications. It may potentially find a more receptive audience in a specialized magnetic resonance or chemistry journal.

In any case, there are also several apparent errors or omissions that should be addressed:

(page 6) Figure 4. The depicted calculated structure of complex 2 appears to only have one DMSO ligand and may therefore be incorrect. (The optimized structure of complex 2 in Fig. S26 does however correspond more closely to the structure for 2 shown in Figs 1 & 2.)

(page 3) "...transition from bidentate to monodentate binding (k3-κ1-κ2-pyr)" does not agree with the definitions in Figure 2. Please either swap the bi- and mono- or change the rate to k3-κ2-κ1-pyr.

(page 6) "We acknowledge that a Thrippleton-Keeler zero-quantum filter based on a chirp pulse with a gradient (Fig. 5A) (ref61) could be used to improve coherence selection efficiency further". Fig. 5A appears to show such a filter with a chirp pulse and simultaneous GPZ0 gradient in the SEPP-SABRE sequence. Is the depicted sequence correct, and was a Thrippleton-Keeler indeed used?

A similar effect of [DMSO] on the formation of complexes 2 and 3 has recently been described in Mamone et al. (doi:10.1039/D5CP01773D), albeit with the old assignment of complex 4 for complex 2. It would be worth acknowledging and citing this prior work.

While evidence is shown to support complex 2 containing two DMSO ligands, does the 1-13C pyruvate chemical shift of complex 2 also support this new assignment of complex 2 instead of complex 4? Given that complex 2/4 has been assigned by the 13C chemical shift, it would further strengthen the new assignment.

While Na+ is attributed to have a substantial effect on the relative free energies of the complexes, is there any experimental evidence for this beyond the fact that SABRE works with sodium pyruvate but not with pyruvic acid?

Minor points:
(page 1) The abbreviation pH2 is introduced without being defined

(page 4) "were revealed using HMQC for DMSO with naturally abundant 13C (ESI, Fig. S19)". Fig. S19 shows a 1H-1H NOESY spectrum rather than HMQC.

(page 4) A character, presumably a lowercase delta to indicate chemical shift, is not displayed properly.

(page 5) "The role of Na+ ... which is described in the next chapter". Perhaps "section" is more appropriate than "chapter".

(page 7) "...rate of conversion to 1 were found to reduce as [H2] increased...". "to decrease" or the like instead of "to reduce"

(page 8) "Interestingly, flux $k_{3\rightarrow 2}$ growed rapidly with [DMSO] ". "grew", or better, "increased" instead of "growed"

Reviewer #3

(Remarks to the Author)
In this article, the authors report novel understanding of an iridium SABRE catalyst's role in hyperpolarization of pyruvate through a combination of thermal equilibrium NMR experiments, hyperpolarized NMR, and computational studies using DFT calculations. In their hyperpolarization experiments, the authors use frequency-selective excitation sequences to perform chemical exchange measurements of protons. The manuscript reports a number of new findings. The authors convincingly show that a previously-described catalyst species is in fact not present in solution, and provide a wealth of new data on ligand and complex exchange dynamics. The authors performed electronic structure calculations using the ORCA 6 program package to support their experimental findings. These computational studies have revealed the importance of sodium ions for catalyst stability, which was not previously known. A key conclusion of this work is that the catalyst system, while effective, is not suitable for polarizing high concentrations of pyruvate. This is an important revelation, since the goal in many hyperpolarization experiments and applications is to maximize the concentration of hyperpolarized target in solution.

The only weakness is that the new knowledge that has been acquired is not then applied to improve the polarization level or concentration of polarized pyruvate. For example, by use of alternative ligands, or inclusion of additional/alternative counterions for catalyst stability. No concrete suggestions are given, and it is left up to future studies to optimize these outcomes. Nevertheless, this is a fantastic piece of work and I congratulate the authors on this carefully-conducted and thorough study that will be important for the research field.

I have a few specific suggestions for the authors to consider:

The authors show that there is rapid intramolecular hydride exchange in complex 3, and in the conclusions they say that rapid exchange of the hydride ligands averages J-couplings hinders polarization transfer in complex 3. But Fig. S21 that is pointed to, does not seem to show that. If the authors have quantitative measurements of intramolecular hydride exchange (Fig. 5), is it not possible to quantitatively predict the effect on pyruvate 13C polarization via simulations? Showing that this mechanism limits pyruvate polarization would be an important result in the manuscript, and if it is a negligible effect, that would still be worth showing.

The title is over-reaching and vague, and should be modified: this article has certainly shed new light on this catalyst and provides important new results, but the title should reflect more precisely the work reported in the manuscript.

In the "DMSO concentration dependency" subsection, I found the discussion regarding the "revised identity of 2" to be confusing, and this pops up a few times in the paper. As I understand, the identity of complex 2 is not revised, but rather the authors have determined its existence, and excluded the existence of complex 4.

In the "Pyruvate concentration dependency" subsection the authors say that the increasing concentration of 3 relative to 2 is not expected from the chemical model, but it's not obvious to me why that is. Further clarification would be helpful here.

The authors state that complex 3 has low stability in solution because the sample degrades, but data is not included to show this. In parallel, they have shown that complex 3 is the most important catalyst species for polarization of pyruvate, so quantification of its degradation would be useful data to include.

Version 1:

Reviewer comments:

Reviewer #1

(Remarks to the Author)
I wish to thank the authors for taking my feedback into account. I found the manuscript significantly improved. The motivation is clearer and the introduction reads better in the new version.

However, I still believe that the second half of the results section is not properly justified. The reported data are surely relevant and useful for the parahydrogen community, but how do they contribute to the story of this paper? As an example,

the section on H2 exchange starts with “The effect of [H2] on these processes was evaluated over the range of 1 to 8.5 bar.” Why was this measurement performed? How does it contribute to reshaping the understanding of SABRE? Although the manuscript was improved, the question of why a measurement is needed still needs to be answered in multiple places.

Detailed comments
- The choice of tenses of verbs needs to be improved. A common choice is the present tense in the introduction and methods and the past tense in the results. In the current manuscript, it goes back and forth between tenses creating a confusion regarding where the authors describe their overall methods, past results, and their current results. Example: In the introduction paragraph that starts with “Given the significant level of contradiction and the importance of […]”, the authors use “we have shown” and “we observe”. I believe that the present tense would make it clearer to the reader that this summarizes the findings of this paper.

- “However, previous density functional theory (DFT) predictions indicated that isomer 4 has a Gibbs free energy 7.6 kJ/mol higher than isomer 344. Such a significant energy difference implies much lower equilibrium concentrations of 4 than of 3, contradicting our experimental observations.” Am I correct in saying that the authors state a contradiction with results they haven’t presented yet? This makes this paragraph difficult to follow.

- “The SEPP-SPINEPT applied to hydrides of 2 yielded four hyperpolarized carbons of this isotopomer of DMSO” I see more than 4 hyperpolarized 13C signals in the SI figure

- “The observed hydrogen dissociation transmission rate for 1 is displayed as k1-H2 and involves several steps, while only the initial and final steps are observable.” Initial reagent and final product might be easier to understand.

(Remarks on code availability)
The link does not contain any code. In contains experimental data (together with pulse sequence files). It does not contain a readme file.

Reviewer #2

(Remarks to the Author)
The authors have addressed my comments, and, in addressing the critique of Reviewer 1, the manuscript is much improved in its presentation and narrative structure. Some of the changes should, however, be further revised to make the manuscript suitable for publication.

- The revised title makes less sense than before. With "Reshaping ... exchange", how does one reshape exchange? Perhaps the comment from Reviewer 3 can again be taken in consideration to reformulate a title that more accurately summarizes the work and its significance.

- The revised Figure 2 is busier and less clear than the prior version. The additional species shown are helpful for understanding the various pathways, but less so for the the long coloured arrows and especially the crowding. Perhaps more space can be used and/or the repetitive presence of the IMes ligand omitted or abbreviated to make the key pathways easier for the reader to understand.

Additionally, should complex 6 have a net positive charge, and should the unnumbered complex with two pyruvates have a net negative charge?

"Single-barbed", "equilibrium" or "half-headed", rather than "harpoon-like", are more conventional descriptions of the arrows for reversible chemical reactions. (both in Fig. 2 caption and main text)

Minor points:
- page 7: "dissociation of H2 via 3 occurs much faster than via 2, and can be inhibited by increasing [DMSO] or accelerated by increasing [H2] through competitive trapping of 5 (Fig. 5H, I)." Should that be Fig. 5F, rather than 5I?

The revised text also contains somes minor grammatical or syntactical errors, but the following may particularly cause some confusion:

- page 2: "which was initially proposed22 and then followed (e.g., recently43) to exist as two isomers". "followed ... to exist" sounds very strange; perhaps "and then followed (e.g., recently43)" can be removed if that citation is not essential here.

- page 7: "Utilization of pH2 and SEPP allowed us to enhance, for example, Ha or Hb proton signals of 1 at 265 K by ~8060-fold at 9.4 T ([DMSO]=75 mM, [Ir] = 4 mM, [pyr] = 40 mM), dramatically accelerating data collection proved to be critical because of the low stability of 3 (the samples can degrade after preparation65)." Run-on sentence.

- page 7: "No additional filters66 were used to enable the observation of the early exchange, and because remaining zero-quantum terms do not contribute to the total integral37 (Fig. 5B)." Sentence fragment.

- page 9: "...was also quantified at high [DMSO] and again very close to the value measured via pyruvate polarization." – ...and was again very close...?

- page 9: "...exchange between 2 and 3, is mediated by 5, is much slower..." - ...and is much slower...?

(Remarks on code availability)

Reviewer #3

(Remarks to the Author)
The authors have addressed all of my suggestions and I recommend publication.

(Remarks on code availability)

Reviewer #4

(Remarks to the Author)
This work of Dr. Pravdivtsev and co-workers presents new insights on the formation of the iridium complex used in SABRE hyperpolarization of 13C-labelled pyruvate through NMR experiments and DFT calculations. Two key points of this study are the characterization of a key intermediate of the reaction pathway and the elucidation of the role of a sodium counterion in the stabilization of the Iridium complex intermediates in the reaction media. The overall manuscript is well written, and the findings are presented with clarity.
I focused my attention on the DFT results presented in this study, as the NMR experiments go beyond my expertise and have been thoroughly evaluated by the previous reviewers. The used computational method (B3Lyp-D4/de2tzvp) is adequate (and properly cited) and the general approach is suitable for the scope of the presented work, consisting in the calculation and comparison of Gibbs free energy of different structures to provide insight on the most stable species. Within the scope of this study, the presented DFT results play a key role in supporting the experimental observations. It is required for the computational data to be thorough and robust in order to assure the presented results are not derived from artifacts of the computational model, especially taken into account the explicit inclusion of Na+ in the model presented in this study. For this reason, I strongly believe that some additional calculations are required and that this work can become suitable for publication in Nature Communications if the following points are addressed:

1. In the computational study section of the main text, the authors start by comparing the relative Gibbs free energies of structures 2, 3 and 4:
"Computational study: Electronic structure calculations at the B3LYP-D4/def2-TZVP CPCM(Methanol) level of theory54–62 yielded relative Gibbs free energy differences of +7.2 and +21.5 kJ/mol for 3 and 4 with respect to 2 (Fig. 4 and ESI, Section 18; using the ORCA 6 program package63,64, see ESI for details"
However, in this section the authors do not present the relative energies of complexes 2, 3 and 4 shown in Figure 1. These energies correspond to the results obtained after the inclusion of a sodium ion in the model, which is presented in Figure S26 of the ESI. One issue is that the adaptation of the computational model by inclusion of a sodium ion is only discussed in detail in the ESI, and these structures containing a sodium ion are not shown at all in Figure 4. Another is that the relative energies of the structures presented in Figure 4-A,B are only mentioned in the figure's caption and not properly discussed in the main text. Therefore, the obtained results should be presented with more clarity and Figure 4 should be modified to show the structures matching the "Gibbs free energy differences of +7.2 and +21.5 kJ/mol for 3 and 4 with respect to 2" described in the text.

2. In Figure 4, the presented electrostatic potentials (computed for C and D) must contain the corresponding colour-coded scale. This scale is also missing in Figure S25 in the ESI

3. In the "Electronic Structure Calculations" of the ESI, there is no mention of conformational search by the authors. While the studied Iridium complexes might be somewhat rigid, the explicit inclusion of Na+ in the computational model requires a conformational search of the system, so the most stable interaction of the Iridium complex with the cation can be obtained as a global minimum (which could require the rotation of Ir-S(DMSO), Ir–O(pyr) and C–C(pyr) bonds).

4. The authors present in the ESI a comparison of Gibbs free energies between 2, 3 and 4 (Figure S24) and the observation that 4 is less stable than 3 is in line with the cited literature (ref. 44). However, the authors discuss that 2 is 20.1 kJ/mol less stable than 3 (and is also less stable than 4), contrarily to experimental findings. I believe the relative instability of 2 can be over-estimated in this case. While 3 and 4 contain a single DMSO ligand, 2 contains two DMSO ligands. As a result, the presented energies are obtained by comparing G(2) to G(3 or 4) + G(free DMSO molecule). This approach tends to over-estimate the entropy of the free DMSO molecule. I would therefore strongly suggest the authors to compute the structures of 3 and 4 with a second explicit DMSO molecule interacting in the second coordination sphere of the complex. This adaptation of the computational model would allow a direct comparison between G(2) and G(3 with a second explicit DMSO) and can decrease the initially obtained 20.1kJ/mol difference.

5. The tables in this section of the ESI are not labelled.

6. Chemical drawings should be included in the ESI figures of this section. Especially due to the bulkiness of the IMes ligand, 3D structures alone are not enough to clearly show the metal-ligand bonds, namely in figures S26, which includes

Na+.

7. The XYZ coordinates of the computed structures are available in the domain presented in the ESI (https://doi.org/10.5281/zenodo.16746641). However, this webpage references an outdated title of the current manuscript and JACS as the source journal. I would like to remind the authors to update the details of this page.

8. A functional benchmark is included in the ESI. However, since all other calculations were performed with def2-TZVP basis set, I don't understand why the authors chose to decrease the level of theory to a double-zeta basis set for this study. Also, performing single-point energy calculations with a different functional than the one used for the optimization is often not recommended. This is because the minimum energy structure can be significantly different from functional to functional, especially when considering: a) functionals that belong to different classes (in this benchmark: GGA, hybrid GGA and meta-GGA), b) hybrid functionals that contain different contributions of HF% in the exchange or present range-correction; c) the studied systems contain transition metal complexes. Therefore, I would recommend the authors to either re-compute the benchmark by optimizing structures 3 and 4 with the tested functionals (instead of computing a single point on a structure optimized with B3LYP-D4/def2-SVP) or remove this section entirely. I also noticed that the XZY coordinates of the structures of 3 and 4 used for this benchmark are not included in the available DFT data

9. Finally, the introduction of Na+ in the model must be further explored with computations. It was observed by the authors in the section "Formation energy of complex 2 from complex 1" of the ESI that computing the Gibbs free energy difference between 2 and 1 is an issue due to the stabilization of Cl-, which needs to dissociate from 1. In the table below Figure S27, it is even possible to observe large discrepancies of computed relative energies, depending on the computational model and, in particular, how the Cl- is stabilized. For this reason, I find surprising that the authors did not apply the same approach to the inclusion of Na+ in the computational model.
The results presented in the main text show that A is 7.2 kJ/mol more stable than B and are computed as shown in Scheme 1 (provided as attachment).
While examining the computed 3D structures, it is possible to observe that the form of chelation of sodium is different from A to B. As a result, the established O–Na bonds present a length 2.20 and 2.30 Å in A, while in B they are longer (both O–Na bonds in B are 2.31 Å) and therefore correspond to weaker interactions, leading to a weaker stabilization of the sodium ion. Given that this energy comparison is a key argument for the proposed characterization of complex 2 within this work, it is imperative to show strong evidence that the relative stability of A is obtained from a significant comparison to B and not due to an artifact of a computational model that is unable to promote the stabilization of the introduced cation.
Therefore, the computations including the sodium cation must be expanded to consider further stabilization of this species. If the trend in relative energies remains (2 being more stable than 3), the computational results would indeed be strong evidence for both the presented assignment of 2 as a key species in the SABRE reactivity and the role of sodium in the system.
I would like to provide the authors with two suggestions on how to approach the stabilization of the sodium ion. As I mentioned in point 4, B has a fewer DMSO molecule than A in the model, which could easily coordinate to the sodium ion. As such, one possible approach would be to consider the interaction of the dissociated DMSO with sodium, as depicted in Scheme 2 (provided as attachment).
Alternatively, the solvent (MeOH) can be included explicitly in the computational model to account for the interactions with Na+, as shown in Scheme 3 (provided as attachment).

(Remarks on code availability)

Version 2:

Reviewer comments:

Reviewer #1

(Remarks to the Author)
I thank the authors for taking my comments into account. I believe the manuscript is improved and can be published as is.

(Remarks on code availability)

Reviewer #2

(Remarks to the Author)
The further revised manuscript is significantly improved, and the authors have addressed the changes I suggested and points raised. The critiques of Reviewer #4 on the computational data raise further questions about its reliability, and in my opinion the role of the Na+ cation is best addressed with experimental evidence, as alluded to in the original round of review. With the various caveats now noted in the computational study section, it is more suitable for publication in its current form.

One minor comment concerning the revised text for Figure 3: "The spectra were hyperpolarized using the SEPP-SPINEPT-SABRE sequence." Perhaps that can be rephrased, unless it really was the spectra and not the samples that were

hyperpolarized.

(Remarks on code availability)

Reviewer #4

(Remarks to the Author)
The authors addressed my main concerns regarding the viability of the computational model by performing additional calculations and I am pleased to see that the new results reveal a much clearer agreement with experiment. The presented computational data no longer requires an explicit interaction with Na+ to achieve a match between the relative Gibbs free energies of intermediate species and the experimental observations. I believe the manuscript can be accepted after the authors adress the following minor details:
-In Figure 4, the size of iridium nucleus is inconsistent in B (very small in comparison to A and C)
-In the main text, I would suggest rephrasing “Within the error bars of DFT” to “within the accuracy of the level of theory” or “computational method”, since error bars were not explicitly calculated for this system.
-In the Figure S26 of the ESI I would insist on the authors to either include the Lewis structures of each computed complex or to explicitly display in the 3D figure the bonds to Na so the reader can easily see the different considered interactions.
-Table S20 of the ESI must contain a typo: the entries of E3 and E4, reoptimized at the DFT-D4/def2-TZVP CPCM level of theory in the column of B3LYP (last column, last 3 rows) have to be exactly the same as the ones in the same column, first 3 rows: -1924.707791733 for E3 and -1924.705312465 for E4.

(Remarks on code availability)

Open Access This Peer Review File is licensed under a Creative Commons Attribution 4.0 International License, which permits use, sharing, adaptation, distribution and reproduction in any medium or format, as long as you give appropriate credit to the original author(s) and the source, provide a link to the Creative Commons license, and indicate if changes were made.
In cases where reviewers are anonymous, credit should be given to 'Anonymous Referee' and the source.
The images or other third party material in this Peer Review File are included in the article’s Creative Commons license, unless indicated otherwise in a credit line to the material. If material is not included in the article’s Creative Commons license and your intended use is not permitted by statutory regulation or exceeds the permitted use, you will need to obtain permission directly from the copyright holder.
To view a copy of this license, visit https://creativecommons.org/licenses/by/4.0/

Comments of the reviewers and corresponding answers

Reviewer: 1

Assaf et al present an investigation of the reaction pathways of the iridium catalyst during the preparation of ^{13}C-hyperpolarized [1-^{13}C]-pyruvate using parahydrogen during SABRE, which is a cost-effective method for enhancing in vivo metabolic MRI. They question the accepted assignment of the 1H hydride signals of the complex and propose a new assignment. Based on this assignment, they monitor in detail the conversion of the complex, including intramolecular exchange. To do so, they introduce a new NMR-based method to monitor specific pathways. Their results suggest that the complex, which was thought to be necessary for SABRE might in fact be detrimental for SABRE efficiency. They conclude on the possible implications of their results for the future optimization of SABRE.

While the results are interesting and certainly useful for the future optimization of SABRE, the weakness of the paper is that it does not give a clear account of what is the state of the art and the established understanding of the SABRE mechanism vs the methods introduced by the authors and the mechanism they propose.

The results are convincing, but in some places, they feel like a collection of observations rather than like a compelling story following a logical course. It makes it hard to follow and not suitable for a large audience. Furthermore, in the current state, the manuscript shows how this study advances the understanding of SABRE hyperpolarization but the impact for a broader readership, as required by Nature Communications, is not clear.

I believe the manuscript could be suitable for Nature Communications after major revisions to make it more accessible and easier to read and to highlight how it advances science in a broader way than only for the parahydrogen community.

Author reply:

We thank the reviewer for their careful and insightful evaluation. We appreciate the recognition of the significance of our results and the points raised regarding clarity, accessibility, and broader impact.

In response, we have reorganized and revised the manuscript to provide a clearer account of the current state of SABRE hyperpolarization, better contextualize our methods and findings, and highlight broader implications beyond the parahydrogen community. The Results section has been restructured to present a more logical and accessible narrative.

Specific comments are addressed in detail below, with corresponding revisions indicated.

Here are some examples where I felt a need for more clarity in what is established vs new:

1) The abstract states "we introduce a parahydrogen-enhanced, spin-selective NMR method to investigate pyruvate binding", but the introduction does not present what methods were previously established and what is the novelty of the introduced methods.

Answer: A brief introduction of previously used methods for pyruvate binding was added to the text.

Exemplary modified text:

Abstract:

Here, we use a parahydrogen-enhanced,…

Modified main text:

Introduction, p2: Some recent studies in this area have deployed in-field PHIP to improve sensitivity, selective pulses to enhance selectivity and magnetization transfer efficiency, and used long-lived ^{15}N signals to measure lower exchange rates[26,32–38].

Results: Hyperpolarization-enhanced tracking of pyruvate exchange:

…. Previously, a somewhat similar approach was proposed to transfer pH_2 spin order to ^{1}H nuclei of ligands[35], which we cannot employ due to the too narrow chemical shift dispersion of ^{1}H pyruvate signals and rapid relaxation of protons.

2) Paragraph starting with "Given the importance of this area, this paper reports […]": This paragraph which summarizes the main findings of the paper could be clearer. The sentence "It was found that the NMR signals proposed to be associated with 4 [...]" is a surprising way to start the description of the results. As a reader, I want to hear what methods were used/what new methods were introduced, and the key findings they allowed to reach. In the current state, things feel are mixed and blurred.

Answer: We thank the reviewer for pointing this out. This part of the text was reorganized and separated into several paragraphs.

Original text: Previous DFT predictions indicated a 7.6 kJ/mol higher Gibbs free energy for **4** than for **3,** which is higher than the experimentally observed equilibrium would suggest.[40]

Given the importance of this area, this paper reports on a reexamination of the pyruvate SABRE system. It was found that the NMR signals proposed to be associated with **4**, grew into a level that surpassed those of **3** with increasing DMSO concentration. This observation motivated us to collect advanced NMR and chemical exchange data, which confirmed that, in fact, $[Ir(H)_2(IMes)(Cl)(\kappa^1\text{-pyr})(DMSO)_2]$, (**2**), forms instead of **4** (**Fig. 1**), as previously assumed. In addition, we now evaluate the pronounced *intramolecular* hydride exchange that was previously unreported. This involved harnessing the fact that the six hydride ligand protons (IrHH) in species **1**-**3** are chemically inequivalent, and hence can be targeted separately by frequency-selective polarization transfer in NMR experiments to facilitate this process.[41,42] The resulting data enable the determination of the kinetic parameters for ligand exchange in **Fig. 2** with high sensitivity by using pH_2. Previously, this sensitization method enabled measurement of the *J*-coupling constants between hydride and ^{13}C nuclei in bound pyruvate.[40]

These observations led to the formulation of the revised SABRE pyruvate-H_2 exchange scheme, as shown in **Fig. 2**. Further computational analysis was undertaken, revealing a role of Na^+ interactions in stabilizing **2**. Consequently, we present several results that necessitate a reinterpretation of earlier data and suggest that further studies are needed to predict the quantitative outcomes of SABRE if an optimal solution is to be reached.

Modified text:

Introduction

…

These included *bis*-DMSO complex $[Ir(Cl)(H)_2(DMSO)_2(IMes)]$ (**1**),[42] and $[Ir(H)_2(\kappa^1\text{-}\kappa^2\text{-pyr})(DMSO)(IMes)]$ which was initially proposed[21] and then followed[43] to exist as two isomers where pyruvate binds in the equatorial plane (**3**) or bridges equatorial-axial sites (**4**) (**Fig. 1**).

However, previous density functional theory (DFT) predictions indicated that isomer **4** has a Gibbs free energy 7.6 kJ/mol higher than isomer **3**.[44] Such a significant energy difference implies much lower equilibrium concentrations of **4** than of **3**, which is in contradiction with our experimental observations. In particular, we have found in this work that the ^{1}H and ^{13}C NMR signals, previously associated with complex **4** in the literature,[22,27] can grow to levels surpassing those of **3** with increasing DMSO concentration or with decreasing the temperature, highlighting the significant flaws in the existing mechanistic model.

Given the significant level of contradiction and the importance of hyperpolarizing pyruvate using a cost-efficient SABRE method, we herein report a comprehensive reexamination of the mechanistic transformations of the pyruvate SABRE system. Importantly, by collecting 2D NMR and chemical-exchange data, we have shown that the previously proposed complex **4** (**Fig. 1**) is not observed and should be excluded from the list of key intermediates in the pyruvate SABRE process. Instead, intermediate $[Ir(H)_2(IMes)(Cl)(\kappa^1\text{-pyr})(DMSO)_2]$ (**2**), forms in the system. Consequently, the corresponding ^{1}H and ^{13}C NMR resonances previously associated with **4**, in fact, belong to complex **2**. Additionally, we observed a pronounced *intramolecular* hydride ligand exchange in complexes **1-3**, which was previously unreported but can significantly limit the observable ^{13}C hyperpolarization levels. Furthermore, we undertook DFT computational analysis of the proposed mechanism, revealing a role for Na^+ interactions in stabilizing **2,** explaining the previous misassignment. Altogether, we present several crucial results that necessitate a reinterpretation of earlier data and suggest that further studies are needed to predict the quantitative outcomes of SABRE if an optimal solution is to be reached.

Our experimental approach leverages the fact that the hydride ligand protons (IrHH) in species **1-3** do not overlap, and can therefore be targeted separately by frequency-selective polarization transfer in exchange NMR experiments.[45,46] The resulting data enabled the determination of the kinetic parameters for ligand exchange in **Fig. 2** with high sensitivity by using pH_2. Previously, this sensitization method enabled measurement of the *J*-coupling constants between hydride ligands and ^{13}C nuclei of bound pyruvate on SABRE complexes.[44]

3) First section of results and discussion: it is not clear from reading the text if the scheme in Figure 1 exists in the literature or is proposed by the authors. The authors should clarify it and, if it exists in the literature, references must be added.

Answer: The scheme in Figure 1 does not appear in the literature in the form it is presented because complex **2** was considered to be extremely short-lived and apparently NMR-invisible. As we show in our manuscript, the observed ^{1}H and ^{13}C signals of this complex were erroneously attributed to another complex (**4**). This error was repeated in multiple high-level papers in the field. Given that complex **2** is one of the key intermediates of pyruvate SABRE, this is one of the important findings of our article, as we devise the correct structure.

The figure was modified. As stated in the text, the figure illustrates the structures and the most important believed stable intermediates discussed previously, plus complex 2.

In the revised manuscript, we clarify that Figure 1 presents an overview of main species of the pyruvate/H_2 exchange in SABRE, highlighting the revised structural assignments. In addition, Figure 2 provides more detailed reaction pathways and exchange steps.

Modified text:

Figure 1 caption:

Modified text: Given the significant level of contradiction and the importance of hyperpolarizing pyruvate using a cost-efficient SABRE method, we herein report a comprehensive reexamination of the mechanistic transformations of the pyruvate SABRE system. Importantly, by collecting 2D NMR and chemical-exchange data, we have shown that the previously proposed complex **4** (**Fig. 1**) is not observed and should be excluded from the list of key intermediates in the pyruvate SABRE process. Instead, intermediate $[Ir(H)_2(IMes)(Cl)(\kappa^1\text{-pyr})(DMSO)_2]$ (**2**), forms in the system. Consequently, the corresponding ^{1}H and ^{13}C NMR resonances previously associated with **4**, in fact, belong to complex **2**. Additionally, we observed a pronounced *intramolecular* hydride ligand exchange in complexes **1-3**, which was previously unreported but can significantly limit the observable ^{13}C hyperpolarization levels. Furthermore, we undertook DFT computational analysis of the proposed mechanism, revealing a role for Na^+ interactions in stabilizing **2**, explaining the previous misassignment. Altogether, we present several crucial results that necessitate a reinterpretation of earlier data and suggest that further studies are needed to predict the quantitative outcomes of SABRE if an optimal solution is to be reached.

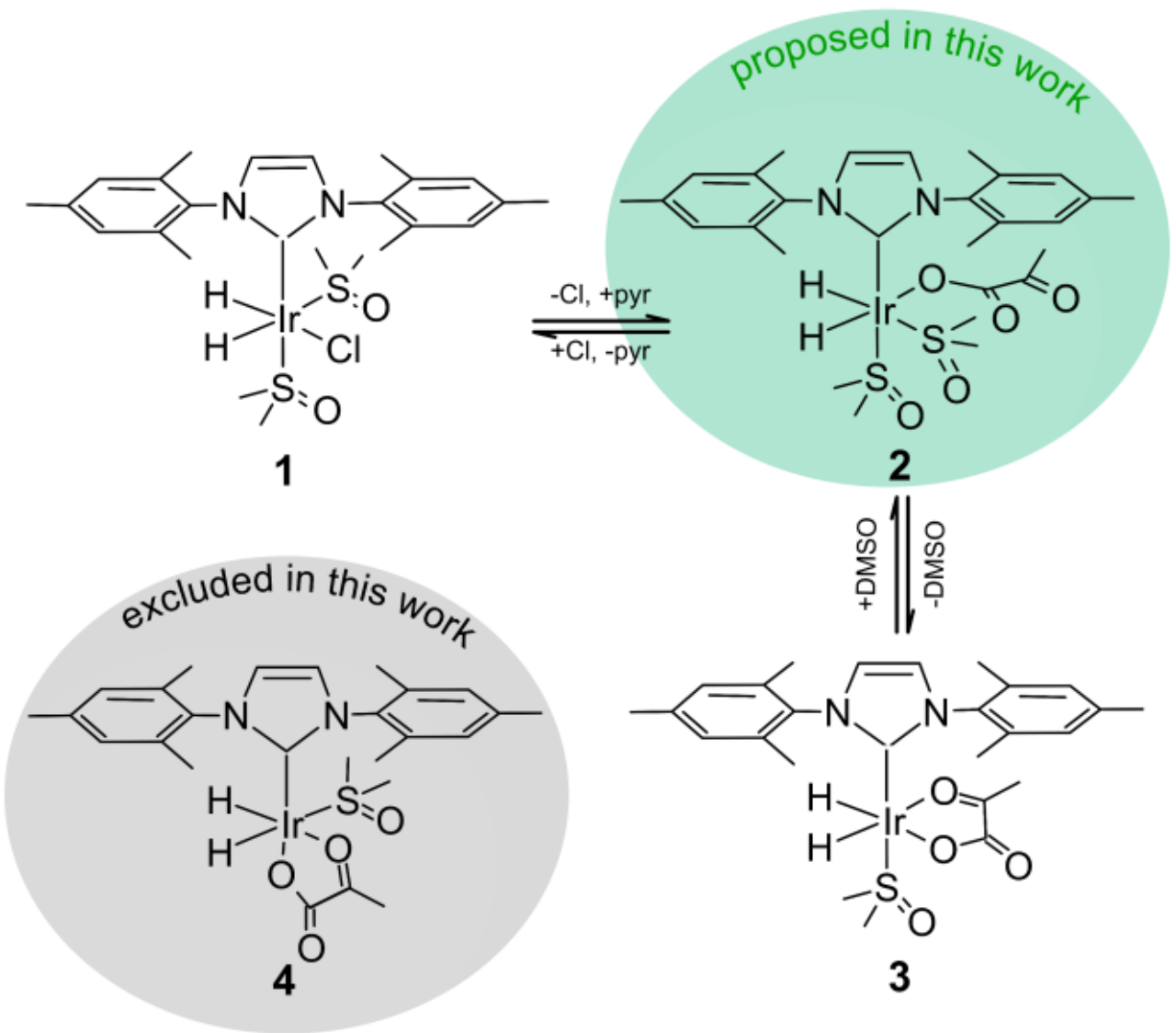

Modified figure

Fig. 1. Revised speciation of Ir complexes in pyruvate SABRE. Structures of complexes **1**-**4**. Complexes **1-3** are formed when IrIMes reacts with DMSO, pyruvate, and H_2 in methanol. Previously, **4** was assumed to be populated to a high level and in exchange with **1** and **3**; however, we show here that it is actually absent and energetically unfavorable compared to its isomer **3**, while **2** is present instead. Therefore, **4** should be excluded from consideration for the discussed pyruvate SABRE composition.

4) I have nothing against the use of the passive form in general but here it often makes it unclear what are previously established findings/hypotheses, and findings/hypotheses of the papers.

Answer: We agree. Since the text was substantially changed, we rewrote many sections accordingly.

5) This manuscript is presumably meant for a large audience as it is submitted to Nat. Comms. However, the authors mention specialized NMR experiments without even specifying the acronyms. If the paper is to be published in Nat. Comms., the acronyms must be detailed. Furthermore, a few words explaining what information each experiment brings would make the paper much more approachable (in particular for EXSY).

Answer:

Thank you for pointing this out. We have now explained acronyms throughout the whole text and clarified many places. The changes are detailed below.

Modified text:

Results:

…

To evaluate the merits of this proposed model, we performed a series of exchange spectroscopy (EXSY) NMR measurements (data in **Figs. 3, 5, and 6**), which allowed us to monitor chemical exchange of free pyruvate, DMSO, and H_2 with their counterparts bound to the iridium complexes. The experiments were undertaken with variations in [H2], [pyr], and [DMSO] concentrations to quantitatively probe the exchange dynamics and support the proposed reaction mechanism. In addition, a rigorous electronic structure analysis was performed through DFT.

Reevaluation of NMR signal assignments and structures in pyruvate SABRE: Previously, the ^{1}H chemical shifts of what we now assign as complex **2** were associated with complex **4** in the literature[22,27,44,47–49]. Our experiments discussed here and below indicate a revised assignment is necessary: the hydride ligands of complex **2** resonate at δ –14.98 and –24.09 ppm (**Fig. 3a**).

The first key evidence came from NOE 2D NMR. We investigated a sample containing IrCl(COD)(IMes), DMSO-h_6, and [2-^{13}C]pyruvate in methanol-d_4 at 253 K after exposure to pH_2. ^{1}H-^{1}H NOE revealed cross peaks between hydride protons of **2** (δ –14.98 and –24.09 ppm) and three methyl groups of DMSO (^{1H}δ 3.37, 2.98, and 2.92 ppm, **ESI**, **Fig. S19**). Due to the low hydride-ligand symmetry, a single DMSO ligand would produce two connections to the hydrides, whereas our measurements with three peaks are indicative of two DMSO ligands.

Then we performed two additional experiments: HMQC (heteronuclear multiple-quantum correlation)[50] ^{1}H-^{13}C correlation 2D NMR (**ESI, Fig. S20**) and ^{1}H-^{13}C SEPP-SPINEPT (frequency-selective excitation of parahydrogen-derived PASADENA polarization followed by frequency-selectively pulsed insensitive nuclei enhanced by polarization transfer, where PASADENA refers to high-field pH_2 experiment – pH_2 and synthesis allow dramatic enhancement of nuclear alignment[51], **ESI**, **Fig. S21**) that transfers polarization from pH_2 to neighboring carbons[44], but now using [U-^{13}C]DMSO. The SEPP-SPINEPT applied to hydrides of **2** yielded four hyperpolarized carbons of this isotopomer of DMSO (**ESI, Fig. S21**). HMQC, on the other hand, provided resonances for the neighboring methyl protons (^{1H}δ-^{13C}δ pairs were 3.37-48.27, 2.98-58.98, 2.92-44.19, and 3.14-50.20 ppm, **ESI, Table S17**). With this we confirmed the presence of two DMSO ligands and assigned four ^{13}C and ^{1}H resonance pairs, establishing that the correct identity of the complex is [Ir(H)$_2$(IMes)(κ^1-pyr)(DMSO)$_2$] (**2**) rather than the previously proposed [Ir(H)$_2$(IMes)(κ^1-κ^2-pyr)(DMSO)], (**4**)[22].

… It is worth noting some limitations of this approach. As with all EXSY-type experiments, it is not always possible to distinguish between nuclear Overhauser effect (NOE) cross-relaxation

and actual chemical exchange; however, in some cases, NOE can lead to a transfer of polarization with the opposite sign, which is equivalent to a negative flux rate constant. Despite this limitation, the exchange rates obtained from the data fitting are of the correct order of magnitude, since chemical exchange processes are faster than NOE cross-relaxation in most of the cases considered here. Therefore, we did not impose any constraints on the flux rates during the fitting process. We observed a consistently negative flux rate constant (indicative of NOE-mediated polarization transfer) when we measured slow intramolecular magnetization exchange between two hydrides in complex **2** (**Fig. 5E, G, I, K**). In all other cases, the flux rates were positive in sign despite an expected but apparently weak negative cross-relaxation contribution.

…Previous density functional theory (DFT) predictions indicated.

Detailed comments

Introduction

6) “Has proven” appears three times in the intro and feels heavy

Answer:

The repeated use of “has proven” in the introduction has been revised to improve readability and stylistic variation. The phrasing was adjusted to use alternative expressions such as “has been,” “has emerged as,” and “has demonstrated great potential,” while preserving the original meaning.

Original text:

1- For more than a decade, [1-^{13}C]pyruvate has proven to be the most prominent tracer for hyperpolarized
2- the dissolution dynamic nuclear polarization (dDNP) hyperpolarization approach has proven to be the most successful method
3- The hydrogenative parahydrogen-induced polarization (PHIP) sidearm hydrogenation variant (PHIP-SAH)[11–13] has proven to be one such alternative, which is underpinned

Modified text:

1- For more than a decade, [1-^{13}C]pyruvate has been the most prominent tracer for hyperpolarized metabolic magnetic resonance imaging[1–3]
2- the dissolution dynamic nuclear polarization (dDNP) hyperpolarization approach has emerged as the most successful translational method.
3- Parahydrogen (pH_2)-based hyperpolarization methods offer faster and more cost-efficient alternatives. Hydrogenative parahydrogen-induced polarization (PHIP), more specifically, its side-arm hydrogenation variant (PHIP-SAH)[11–13] has demonstrated great potential as a viable alternative to dDNP, which is underpinned by the high-yield synthesis of essential precursors[11,12,14–17]. Additionally, it has achieved high ^{13}C polarization levels of pyruvate around 20%[18].

7) Because the introduction focuses precisely on the production of hyperpolarized pyruvate for in vivo MRI, a few words comparing the performance (polarization, concentration, purity, preparation time) of the different methods (dDNP, PHIP, SABRE) would be welcome.

Answer:

We thank the reviewer for the helpful suggestion. We have added a concise comparison of the main hyperpolarization methods (dDNP, PHIP, SABRE) in the introduction, including key performance indicators where they are most informative. However, the field is evolving at such a pace that any statement of the current state of the art risks becoming obsolete almost immediately — *tempora mutantur*. Moreover, our work does not aim to promote or further optimize for example purification methods but to study the underlying catalysis. While specialists may locate detailed performance data in the literature, including extensive site specifics (e.g., manual purification routines) would be too technical for a broad readership and not essential for understanding the scope of our study.

Modified text:

Introduction:

For more than a decade, [1-^{13}C]pyruvate has been the most prominent tracer for hyperpolarized metabolic magnetic resonance imaging[1–3]. The reason for this is a sufficiently long lifetime of the polarization, a sufficiently high applicable dose, rapid in vivo metabolic transformation into lactate, alanine, bicarbonate, and CO_2, and their association with inflammation[3] and tumor malignancy[4,5] as a result of the Warburg effect[6]. To achieve the necessary increase in MRI detectability of pyruvate and its metabolic products, the dissolution dynamic nuclear polarization (dDNP) hyperpolarization approach has emerged as the most successful translational method.[7] This method can produce up to 60% of ^{13}C polarization and deliver millimolar concentrations of pyruvate for in vivo use following a 30 to 60 minute polarization and dissolution process, but it requires costly equipment. However, the hyperpolarization community widely aspires to create swifter and more cost-effective alternatives to hyperpolarize biomolecules,[8] with one prominent route being the use of parahydrogen-based methods[9–11].

Parahydrogen (pH_2)-based hyperpolarization methods offer faster and more cost-efficient alternatives. Hydrogenative parahydrogen-induced polarization (PHIP), more specifically, its side-arm hydrogenation variant (PHIP-SAH)[12–14] has demonstrated great potential as a viable alternative to dDNP, which is underpinned by the high-yield synthesis of essential precursors[12,13,15–18]. Additionally, it has achieved high ^{13}C polarization levels of pyruvate around 20%[19]. The non-hydrogenative variant of PHIP, signal amplification by reversible exchange (SABRE)[20], offers another route to pyruvate hyperpolarization[19,21,22]. In SABRE, a ligand (e.g., pyruvate) and pH_2 are temporarily associated with an Ir complex, whose spin-spin interactions allow the spin alignment of pH_2 to convert into the polarization of a ligand. It enables direct hyperpolarization without altering the substrate, allowing rapid polarization buildup within seconds under mild conditions. However, current implementations typically achieve lower ^{13}C polarization levels of a few percent for pyruvate and require careful control of exchange dynamics to optimize the performance. The strength of the spin-spin interaction and the lifetime of the complex play critical roles in maximizing polarization transfer efficiency[23–25]; the latter is controlled by tuning the temperature[26] or ligand identity[27].

8) "However, the hyperpolarization community widely aspires to create swifter and more cost-effective alternatives to hyperpolarize biomolecules using parahydrogen." This statement sounds a little overstretched. Parahydrogen is not the only way the hyperpolarization community seeks to make the hyperpolarization of biomolecules more widespread or faster. This statement should be toned down, for example saying "one route is SABRE…".

Answer: We thank the reviewer for this valuable suggestion. We revised the sentence.

Modified text: However, the hyperpolarization community widely aspires to create swifter and more cost-effective alternatives to hyperpolarize biomolecules, with one prominent route being the use of parahydrogen-based methods[8–10].

9) "In this case, the measurement of ligand loss rates for the dissociative exchange pathway that SABRE catalysts like [Ir(H)2(IMes)(pyridine)3]Cl follow is standard28–30." By "standard", do the authors mean that it's easy/routinely done?

Answer:

We thank the reviewer for this comment. By "standard," we intended to convey that measuring ligand loss rates for the dissociative exchange pathway in SABRE catalysts is a well-established approach in the field, rather than implying it is necessarily easy or routine. The sentence has been revised in the manuscript to read:

Modified text: For a better understanding of the system, it is desirable to measure ligand loss rates for the dissociative exchange pathway following the SABRE catalysts, such as $[Ir(H)_2(IMes)(pyridine)_3]Cl$[29–31].

10) "[…] as this process typically happens after substrate loss and is complicated by the role a dihydrogen-dihydride complex plays in the exchange mechanism28,31." polarization or molecular exchange?

Answer:

The phrase "this process" refers to chemical exchange of H_2, not polarization transfer. To clarify, we have revised the sentence in the manuscript to read:

Original text: However, few reported studies focus on determining the H_2 exchange rate[31], as this process typically happens after substrate loss and is complicated by the role of a dihydrogen-dihydride.

Modified text: However, few reported studies focus on determining the H_2 exchange rate[32], as this chemical exchange process typically occurs after substrate loss and is complicated by the role that a dihydrogen-dihydride complex plays in the exchange mechanism[29,32].

11) "[…] which is proposed to exist as two isomers where pyruvate binds in the equatorial plane (3) or bridges equatorial-axial sites (4)". Would be good to make it clear in which reference this hypothesis was proposed. 39? 21?

Answer:

The hypothesis of these two isomers was originally proposed in reference Iali et al., ACIE 2019. We have updated the manuscript to explicitly indicate this reference by placing it after "isomers" in the relevant sentence.

Original text: which is proposed to exist as two isomers where pyruvate binds in the equatorial plane (**3**) or bridges equatorial-axial sites

Modified text: These included *bis*-DMSO complex $[Ir(Cl)(H)_2(DMSO)_2(IMes)]$ (**1**),[42] and $[Ir(H)_2(\kappa^1\text{-}\kappa^2\text{-pyr})(DMSO)(IMes)]$, which was initially proposed[22] and then followed (e.g. recently [43]) to exist as two isomers where pyruvate binds in the equatorial plane (**3**) or bridges equatorial-axial sites (**4**) (**Fig. 1**).

12) "Previous DFT predictions indicated a 7.6 kJ/mol higher Gibbs free energy for 4 than for 3, which is higher than the experimentally observed equilibrium would suggest." This argument is difficult to follow as is: equilibrium between which states?

Answer:

We thank the reviewer for pointing this out. The sentence has been revised to clarify that the equilibrium refers to the relative populations of isomer complexes **3** and **4** in solution.

Original text: Previous DFT predictions indicated a 7.6 kJ/mol higher Gibbs free energy for **4** than for **3,** which is higher than the experimentally observed equilibrium would suggest.[40]

Modified text: However, previous density functional theory (DFT) predictions indicated that isomer **4** has a Gibbs free energy 7.6 kJ/mol higher than isomer **3**[44]. Such a significant energy difference implies much lower equilibrium concentrations of **4** than of **3**, contradicting our experimental observations.

13) "[…] [Ir(H)2(IMes)(Cl)(κ1-pyr)(DMSO)2], (2), forms instead of 4 (Fig. 1), as previously assumed." A reference is needed.

Answer: We have added reference reference and revised the text.

Original text: which confirmed that, in fact, $[Ir(H)_2(IMes)(Cl)(\kappa^1\text{-pyr})(DMSO)_2]$, (**2**), forms instead of **4** (**Fig. 1**), as previously assumed.

Modified text: Importantly, by collecting 2D NMR and chemical-exchange data, we have shown that the previously proposed complex **4** (**Fig. 1**) is not observed and should be excluded from the list of key intermediates under normal conditions. Instead, intermediate $[Ir(H)_2(IMes)(Cl)(\kappa^1\text{-pyr})(DMSO)_2]$ (**2**), forms in the system. Consequently, the corresponding ^1H and ^{13}C NMR resonances previously associated with **4**, in fact, belong to complex **2**.

Results:

Chemical exchange models. Several Key features of the pyruvate SABRE system:

14) "Several" in the title is superfluous

Answer:

The word "Several" has been removed from the title.

Original text: Chemical exchange models. Several Key features of the pyruvate SABRE system:

Modified text: Chemical exchange models. Key features of the pyruvate SABRE system:

15) "Hence, dissociative loss of DMSO and chloride in 1 and 2 (Fig. 1) is likely." I understand that the authors are transposing a well-established mechanism of pyridyl-type systems to the pyruvate case. If correct, this could be made clearer.

Answer:

Indeed, we are transposing the dissociative exchange mechanism of pyridyl-type SABRE systems to the pyruvate case. We have clarified the text to make this connection explicit for the reader.

Original text: As was pointed out in the introduction, a relatively well-studied SABRE mechanism for pyridyl-type systems indicates its dissociative loss and associative exchange of H_2. Hence, dissociative loss of DMSO and chloride in **1** and **2** (**Fig. 1**) is likely.

Modified text: As noted in the introduction, a relatively well-studied SABRE mechanism for pyridyl-type systems indicates dissociative loss of pyridine and associative H_2 exchange. Hence, by analogy, dissociative loss of DMSO and chloride in **1** and **2** (**Fig. 1**) is likely.

16) "A role for complex 4 was ruled out through detailed experimental and theoretical evidence, as described later." This is an example where the passive form gets in the way of understanding. Furthermore, the authors are already ruling out a mechanism at the point where they are setting up the work hypothesis.?

Answer:

We thank the reviewer for this comment. We agree that the original phrasing could suggest a conclusion was stated prematurely. In the revised manuscript, we have rephrased this sentence to use active voice and indicate that the absence of a role for complex 4 is a finding supported by the experimental and theoretical evidence presented in our article. This clarifies the logical flow and maintains consistency with the discussion of Figure 2.

Original text: A role for complex **4** was ruled out through detailed experimental and theoretical evidence, as described later (**ESI, Fig. S24-29**).

Modified text: As was noted above, based on the experimental and theoretical evidence supported by DFT calculations (**ESI, Fig. S24–29**), we have found that complex **4** (**Fig. 1**) could not play a significant role in pyruvate SABRE and ruled it out from the list of key intermediates of this system in **Fig. 2**. Instead, we prove the existence of complex **2** as one of the key species for pyruvate SABRE along with complexes **1** and **3**.

17) "Paragraph starting with "The corresponding bimolecular ligand rebinding is indicated […]" This paragraph is confusing: it states that figure 2 shows some rebinding steps, but then each listed element is specified "not shown".

Answer:

We thank the reviewer for pointing this out. The listed bimolecular rebinding steps correspond to species that are not shown in **Figure 2** and were not detected experimentally. **Figure 2** is intended to illustrate the primary observed and hypothesized mechanistic exchange pathways only, while the additional possible intermediates are not part of the observed species. Text was shortened and clarified.

Original text: The corresponding bimolecular ligand rebinding is indicated in **Figure 2** as k_{1+Cl}, k_{1+pyr}, k_{1+DMSO} (this is not shown, and would lead to undetected $[Ir(H)_2(IMes)(DMSO)_3]Cl$, (**5**)), k_{2+Cl} (not shown, leading to undetected $[Ir(H)_2(IMes)(\kappa^1\text{-pyr})(DMSO)Cl]$, (**6**)), k_{2+DMSO} and k_{2+pyr} (not shown, leading to undetected [*cis-cis* $Ir(H)_2(IMes)(\kappa^1\text{-pyr})_2(DMSO)]$, (**7**)).

Modified text:

Figure 2 shows such a hypothesized reaction pathway that allows the equilibration of **1**, **2,** and **3** through a series of dissociative reaction steps involving loss of Cl^- ($k_{1\text{-Cl}}$), DMSO ($k_{2\text{-DMSO}}$), and pyr^- ($k_{2\text{-pyr}}$), or pyr transition from bidentate to monodentate binding ($k_{3\rightarrow6}$) and back ($k_{6\rightarrow3}$). These steps involve the 16-electron intermediates, **5** ($k_{2\text{-DMSO}}$, $k_{3\rightarrow5}$) and $[Ir(H)_2(IMes)(DMSO)_2]^+$ (**6**, $k_{2\text{-pyr}}$ and $k_{1\text{-Cl}}$). These intermediates are expected to bind DMSO, methanol, Cl^-, pyruvate, and water. Unless complexes **1-3** are formed, the resulting species are unobservable in NMR because they are energetically very unfavorable. Care should be taken when viewing this figure, as it shows elementary reaction steps (grey rates with harpoon-like arrows) and transmission rates (not grey rates with regular arrowheads). The difference is that sometimes we can observe only the reagent and the product, not the individual steps.

18) "The required associative bimolecular H2 loss […]" Required for what?

Answer:

The associative bimolecular H_2 loss is required to enable intramolecular hydride exchange via the 16-electron intermediates. We have clarified this point in the revised manuscript.

Original text: The required associative bimolecular H_2 loss is facilitated by first binding H_2 to the corresponding 16-electron intermediate according to $k_{1\text{-H2}}$, $k_{2\text{-H2}}$, and $k_{3\text{-H2}}$. These two intermediates were proposed to play a role in hydride site interchange (i.e., intramolecular hydride exchange) wherein the hydride ligands interchange positions.

Modified text:

These intermediates are expected to bind DMSO, methanol, Cl^-, pyruvate, and water. Unless complexes **1-3** are formed, the resulting species are unobservable in NMR because they are energetically very unfavorable. Care should be taken when viewing this figure, as it shows elementary reaction steps (grey rates with harpoon-like arrows) and transmission rates (not grey rates with regular arrowheads). The difference between them is that, typically, we observe only the overall transmission between reagents and products, not the intermediate steps.

The associative bimolecular H_2 loss occurs via binding of H_2 to **5** and **6** intermediates (**Fig. 2**). We propose that these two intermediates also enable the hydride ligands to interchange positions. The corresponding, now first-order, rearrangement steps are indicated as "hydrogen swap" in **Fig. 2**. The observed hydrogen dissociation transmission rate for **1** is displayed as $k_{1\text{-H2}}$ and involves several steps, while only the initial and final steps are observable.

19) "To evaluate the merits of this proposed model, a series of experimental EXSY-type measurements […]" This section presents the proposed mechanisms, but this part of the paragraph mentions the experiments undertaken to study of the mechanism. This makes the story hard to follow.?

Answer:

We thank the reviewer for this comment. We agree that the original paragraph conflated the proposed mechanism with the experimental approach, which could make the story harder to follow. In the revised manuscript, we have reorganized the text to first introduce the proposed mechanism and then clearly describe the EXSY-type experiments used to evaluate it, including their purpose and the information they provide.

Original text: To evaluate the merits of this proposed model, a series of experimental EXSY-type measurements (data in Fig.s 3, 5, 6) were undertaken as a function of the [H2], [pyr], and [DMSO] concentrations alongside a rigorous electronic structure theory analysis through DFT.

Modified text: In the sections below, we evaluated the validity of the model by performing a series of exchange spectroscopy (EXSY) NMR measurements (data and experimental schemes are in **Figs. 3, 5, and 6**). EXSY allowed us to monitor the chemical exchange of free pyruvate, DMSO, and H_2 with their counterparts bound to the iridium complexes. We repeated experiments with varying concentrations of [H_2], [pyr], and [DMSO] to quantitatively probe the exchange dynamics. Then we discussed the impact of concentrations on rates of chemical transformations in the context of the proposed reaction mechanisms here or previously. In addition, we performed a rigorous electronic-structure analysis using DFT

20) "Figure 2: the arrows in the conversion from and to 1 shows +pyr and -pyr by pyruvate isn't present in the product and reagent.

Answer: You are right, figure was modified accordingly.

21) "Figure 2: The arrows leading to H2 (from 2 and 3) do not follow the same logic as the rest of the figure. Instead of linking complexes, they link a complex to H2 and the complex disappears. Although I think I understand the point the authors are making, I find this is a little confusing.

Answer: We redesigned the figure, changed the figure caption and wrote some explanation in the text. We hope that now the figure is more self-explanatory.

Modified figure (Fig. 2):

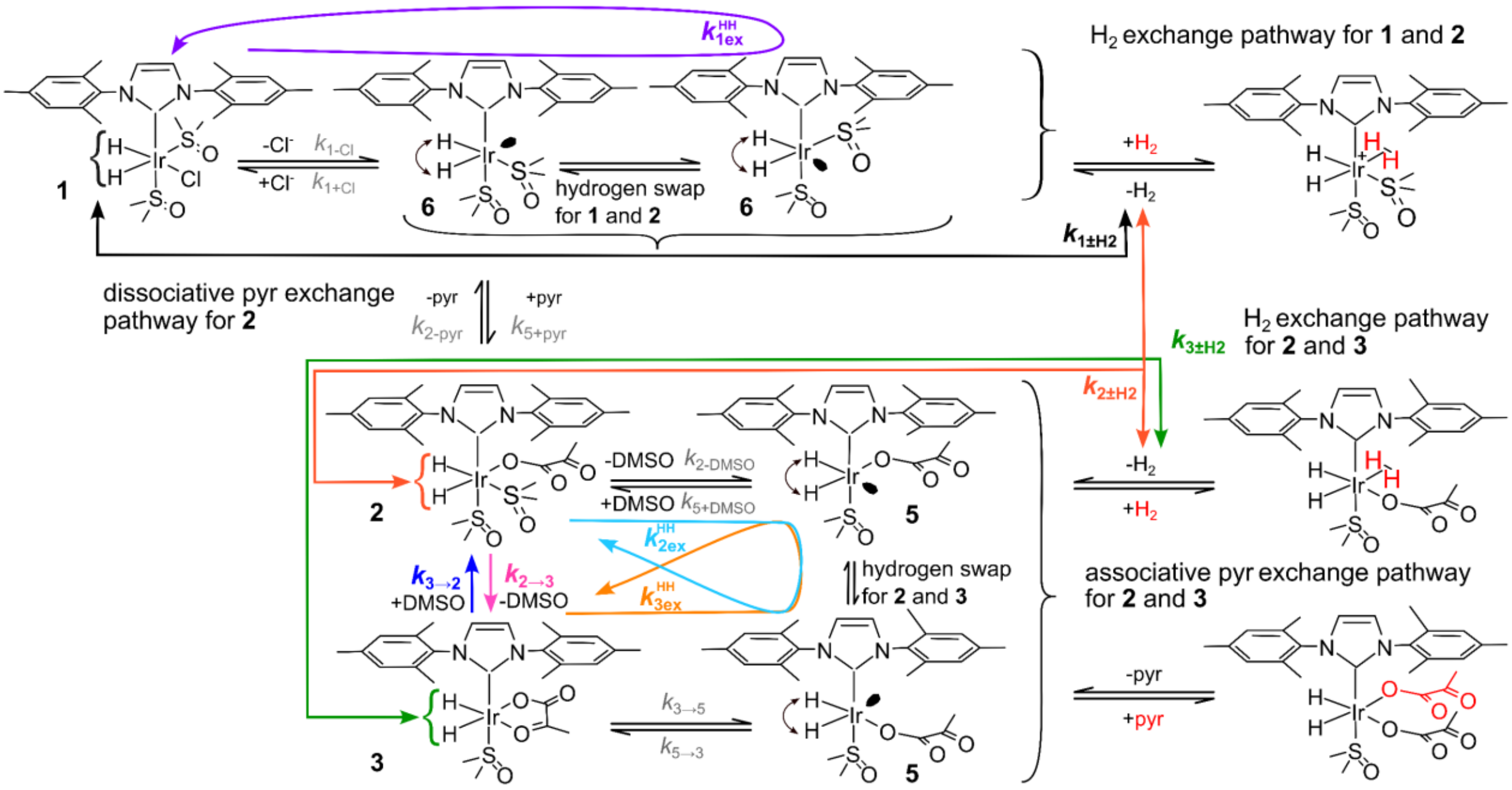

Fig. 2. Schematic of pyruvate, DMSO, and H_2 exchange, which explains the observed experimental kinetic dependencies. Critical elements of the exchange: (1) H_2 and pyruvate exchange on a transition between **1** and **2**, where "+pyr" and "–pyr" indicate coordination from

or dissociation to the solution phase; (2) intramolecular H-H exchange happens during rearrangement between **2** and **3** and **1** and **2**; (3) transformation from **3** to **2** is [DMSO] dependent. Elementary reaction steps are indicated with grey colored rates and harpoon-like arrows. In contrast, transmission rates are indicated with colors and regular arrowheads. For example, transmission $k_{3\mathrm{ex}}^{\mathrm{HH}}$ involves multiple transformations going from **3** to **5** and back to **3**. In practice, it is notable that species **2** and **3** are stabilized in solution by Na^+ interactions associated with the oxygen centers in the pyruvate ligand. Note that for the sake of simplicity $k_{2\mathrm{ex}}^{\mathrm{HH}}$ is indicated only in the cycle **2**-**5**-**2**, while it could also occur in **2**-**6**-**2**. The transmission $k_{3-\mathrm{pyr}}$ is not indicated but goes from **3** to **5** to associative pyr exchange. Transmission $k_{2-\mathrm{pyr}}$ consists of two pathways, one through complex **5** (associative) and the other one through complex **6** (dissociative).

Reevaluation of the SABRE complex structures and NMR signal assignment:

22) "Previously, the 1H chemical shifts of (here) complex 2 were associated with complex 4 instead26,40,43–46." Hard to follow. What signals? I suppose the authors should refer to Fig 3a. Then the rest of the paragraph mixes established facts that the authors challenge, conclusions drawn by the authors, and some results. The structure should be clearer. What is most troubling is that conclusions are drawn before the results are even stated. Then the first results that are mentioned are not even in the main text but it the SI.
23) "Among others, we used NOE of two hydride ligands that […]" We are finally getting to actual results. However, they are mentioned in an intro-like fashion, with no mention of a figure. Regarding NOE, was this H-H NOE? How was the NOE experiment used to draw the authors' conclusion. Looking at Figure S19 (and not S20), I understand that the presence of a cross-peak in the 1H-1H-NOE spectrum led the authors to their conclusion. This should be clearly stated.?
24) "At the same time, connections between the hydrides and Me groups of the IMes ligand were also expected." And so what? Expected by who? This sentence and the rest of the paragraph are unclear.?
25) "The hydride connections to DMSO signals at 3.37 ppm, 2.98 ppm, and 2.92 ppm […]". The hydride connections of what complex?

Answer: Thank you for asking these questions. We revised the logic of this section which is now follow more clear step by step explanation.

Modified text:

Reevaluation of NMR signal assignments and structures in pyruvate SABRE: Our experiments discussed here and below indicate that a revised assignment of SABRE complexes is necessary. In particular, we believe that ^{1}H NMR signals of hydride ligands in complex **2** at δ –14.98 and –24.09 ppm (**Fig. 3a**) were incorrectly ascribed to complex **4** in the literature[22,27,43,44,47–49].

The first key evidence came from NOE 2D NMR. We investigated a sample containing IrCl(COD)(IMes), DMSO-*h*₆, and [2-^{13}C]pyruvate in methanol-*d*₄ at 253 K after exposure to H_2. ^{1}H-^{1}H NOE revealed cross peaks between hydride protons of **2** (δ –14.98 and –24.09 ppm) and three methyl groups of DMSO (^{1H}δ 3.37, 2.98, and 2.92 ppm, **ESI**, **Fig. S19**). Due to the low hydride-ligand symmetry, a single DMSO ligand would produce two connections to the hydrides, whereas our measurements with three peaks are indicative of two DMSO ligands.

Then we performed two additional experiments: HMQC (heteronuclear multiple-quantum correlation)[49] ^{1}H-^{13}C correlation 2D NMR (**ESI, Fig. S20**) and ^{1}H-^{13}C SEPP-SPINEPT

(frequency-selective excitation of parahydrogen-derived PASADENA polarization followed by frequency-selectively pulsed insensitive nuclei enhanced by polarization transfer, where PASADENA refers to high-field pH_2 experiment – pH_2 and synthesis allow dramatic enhancement of nuclear alignment[51]) that transfers polarization from pH_2 to neighboring carbons[44], but now using [U-^{13}C]DMSO. The SEPP-SPINEPT applied to hydrides of **2** yielded four hyperpolarized carbons of this isotopomer of DMSO (**ESI, Fig. S21**). HMQC, on the other hand, provided resonances for the neighboring methyl protons ($^{1H}\delta$-$^{13C}\delta$ pairs were 3.37-48.27, 2.98-58.98, 2.92-44.19, and 3.14-50.20 ppm, **ESI, Table S17**). With this we confirmed the presence of two DMSO ligands and assigned four ^{13}C and ^{1}H resonance pairs, establishing that the correct identity of the complex is $[Ir(H)_2(IMes)(\kappa^1\text{-pyr})(DMSO)_2]$ (**2**) rather than the previously proposed $[Ir(H)_2(IMes)(\kappa^1\text{-}\kappa^2\text{-pyr})(DMSO)]$, (**4**)[22].

Temperature dependency:

26) "Why are the authors undertaking this measurement series? The section should start with a direction. Otherwise, it feels like an internal report-like list of observations. This applies to other sections.

Answer: Agree. The directions were given in the revised version. Generally, temperature is an important parameter influencing populations of the SABRE species and provides energetics insights of the system transformation.

Modified text:

Evaluation of thermodynamic properties of the pyruvate SABRE system: Temperature strongly influences the kinetic parameters and the equilibrium populations of the complexes, affecting the hyperpolarization process[26]. Moreover, it provides insights into the energetics of the intersystem conversion, which can then be compared with ab initio simulations to verify the SABRE mechanism. Therefore, we first measured the fractions of complexes **1**-**3** as a function of temperature by using ^{1}H NMR signals of hydride ligands (**Fig. 3C**).

DMSO concentration dependency:

27) "[…] 1, 3, and 4 are considered to be the primary complexes in SABRE, […]" I don't understand the word "primary" in this context, but it might be a lack of knowledge of mine.

Answer: We clarified this sentence.

Modified text: We have found that when the concentration of DMSO was increased, the relative proportions of the two-DMSO complexes, **2** and **1**, increased, while that of the one-DMSO complex, **3**, decreased (**Fig. 3D**). This behavior cannot be rationalized with the previous assignment of complexes **1**, **3,** and **4**, since in this case, only [**1**] would increase, while [**3**] and [**4**] would decrease.

28) "This data highlights why polarization of high [pyr] could be a challenge." is or could be? Are the others referring to a known challenge? If so, they should add a reference.

Answer: Thank you for your interest. The sentence was changed to clarify it.

Modified text: This data aligns with the previous observations in which polarization yield is negatively correlated with [pyr][49,53].

Computational study:

29) "[…] in agreement with our findings" The section on T dependence gives value for 2<-3 and 1<-3, while the DFT results are for 2<-3 and 2<-4. I don't understand the comparison that is made between DFT and experimental results. A table would help.

Answer:

We thank the reviewer for this suggestion, we actually have a figure with the calculated relative energies in the SI. However, the discussion is complicated by the necessity to treat counter ions and we would like to keep these technical details as part of the SI, rather than extending the manuscript much further. The text is modified.

Original text: Electronic structure calculations at the B3LYP-D4/def2-TZVP CPCM(Methanol) level of theory[47–55] yielded relative Gibbs free energy differences of +7.2 and +21.5 kJ/mol for **3** and **4** with respect to **2** (**Fig. 4** and **ESI, Section 18**), in agreement with our findings (using the ORCA 6 program package[56,57], see ESI for details).

Modified text: Computational study: Electronic structure calculations at the B3LYP-D4/def2-TZVP CPCM(Methanol) level of theory[54–62] yielded relative Gibbs free energy differences of +7.2 and +21.5 kJ/mol for **3** and **4** with respect to **2** (**Fig. 4** and **ESI, Section 18**; using the ORCA 6 program package[63,64], see ESI for details). This is in agreement with the experimentally evaluated thermodynamic parameters presented above, which indicate that **2** is favorable over **3** in energy by about 5 kJ/mol. The computational findings and the DMSO concentration dependence could not be explained if **4** was a thermodynamically accessible species.

30) "In addition to this fact, SABRE works only with sodium pyruvate and not with pyruvic acid, which is another clear indication of the critical role Na+-pyr--pairing plays in complex stabilization." Add reference to literature or data or specify "not shown".

Answer: Yes, this should be clarified.

Modified text: In addition, SABRE presumably works only with sodium pyruvate as originally proposed, not with pyruvic acid, for which SABRE has not been shown to work so far, which is another clear indication of the critical role Na^+-pyr pairing plays in complex stabilization.

Hyperpolarization-enhanced observation of proton exchange:

31) "We acknowledge that a Thrippleton-Keeler zero-quantum filter based on a chirp pulse with a gradient […]" Do I understand correctly that Fig 5A shows a pulse sequence that was not used? That's a surprising choice. I think it would be more appropriate to show in the figure the exact pulse sequence that used.

Answer: We were thinking it would be useful for followers, but since it is confusing, we removed it from the figure and modified the text.

Modified text:

A simple two-step phase cycle and dephasing gradients were used to improve background signal suppression, with a 180° phase shift of the first pulse and the receiver to suppress signals unaffected by the frequency-selective first pulse (**Fig. S2B**). No additional filters[66] were used to enable the observation of the early exchange, and because remaining zero-quantum terms do not contribute to the total integral[37] (**Fig. 5B)**. It is also important that relaxation is

taken into account in the data fitting process, as the T_1's of the interchanging sites may be significantly different from each other.

32) "It is worth noting some limitations of this approach. As with all EXSY-like experiments, one cannot distinguish between NOE cross-relaxation and chemical exchange;" It is positive that the authors state this limitation. However, they should probably discuss how this affects the interpretation of the data (and possibly refer the reader to the SI).

Answer:

We thank the reviewer for highlighting this point. This is a rather well-known effect for all sorts of Exchange spectroscopy in NMR. We now clarify in the manuscript how the limitations of EXSY-like experiments affect data interpretation.

Modified text:

It is worth noting some limitations of this approach. As with all EXSY-type experiments, it is not always possible to distinguish between nuclear Overhauser effect (NOE) cross-relaxation and actual chemical exchange; however, in some cases, NOE can lead to a transfer of polarization with the opposite sign, which is equivalent to a negative flux rate constant. Despite this limitation, the exchange rates obtained from the data fitting are of the correct order of magnitude, since chemical exchange processes are faster than NOE cross-relaxation in most of the cases considered here. Therefore, we did not impose any constraints on the flux rates during the fitting process. We observed a consistently negative flux rate constant (indicative of NOE-mediated polarization transfer) when we measured slow intramolecular magnetization exchange between two hydrides in complex **2** (**Fig. 5E, G, I, K**). In all other cases, the flux rates were positive in sign despite an expected but apparently weak negative cross-relaxation contribution.

Hydrogen site intramolecular exchange

33) "readily evident in all of these NMR spectra." What are "these"?

Answer:

We thank the reviewer for pointing this out. "These NMR spectra" refers specifically to the ^{1}H NMR spectra presented in Figure 5B, with the corresponding integrals shown in Figure 5C. We have clarified this in the revised manuscript.

Modified text: Hydrogen site *intramolecular* exchange in 3: The additional process of hydride site exchange in **3**, flux k^{HH}_{3ex}, was readily evident in all of the ^{1}H NMR spectra shown in **Fig.5B**, with directly analogous behavior seen regardless of which hydride ligand was selected, alongside the slightly slower transfer of the magnetization into that of free H_2 (integrals shown in **Fig. 5C**).

34) "The fact that we always observed uniform conversion from, e.g., Ha excited state to Ha and Hb of the same complex 3 or complex 2 indicates that kHHex flux >>[DMSO]*k2+DMSO, and, k3-κ1-κ2-pyr." This sentence is very hard to follow.

Answer:

We thank the reviewer for pointing this out. We have revised the sentence for clarity. The updated text explicitly states that the uniform conversion of an excited hydride to both H^a and H^b indicates that the intramolecular hydride exchange rate (k^{HH}_{ex} flux) is much faster than competing processes such as DMSO binding or pyruvate rearrangement, and we clarify how this is modulated by [DMSO] and [H_2].

Modified text: Moreover, the observed k^{HH}_{3ex} flux decreased gradually with increasing [DMSO], which is consistent with possible interception in the κ^1 intermediate **5** at k_{2+DMSO} step. Notably, the symmetric conversion observed from an excited hydride (e.g., H^a) to both H^a and H^b within the same complex **3** or **2** indicates that the intramolecular hydride exchange is much faster than competing processes, such as DMSO binding ([DMSO]·k_{5+DMSO}), or pyruvate rearrangement ($k_{5\rightarrow 3}$). Furthermore, the dissociation of H_2 via **3** occurs much faster than via **2**, and can be inhibited by increasing [DMSO] or accelerated by increasing [H_2] through competitive trapping of **5** (**Fig. 5H, I**).

Hydrogen-observed intramolecular exchange in 2

35) "We observed the polarization buildup of the negative sign" Polarization buildup of what?

Answer:

We thank the reviewer for this comment. The "polarization buildup of the negative sign" refers to the non-excited hydride signal within complex **2**. We have clarified this in the revised version.

Modified text: In contrast to intramolecular exchange in **3**, when the two hydride ligand signals of **2** were excited separately in two separate experiments, no hydride site interchange is evident on the same reaction timescale. Instead, we observed the buildup of negative polarization of the non-excited hydride, indicating a cross-polarization effect within the complex. Accordingly, the fitted conversion rate appears negative (**Fig. 5E, G, I, K**). This shows that polarization is first transferred with opposite sign to the other hydride before dissociation to free H_2.

Reviewer: 2

Assaf and colleagues describe a study of the kinetics of iridium complex formation with hydrogen and pyruvate used in SABRE hyperpolarization of 13C-labelled pyruvate. With extensive characterization of the dependence on temperature and concentration and of the interconversion of the various complex complexes, this study sheds fresh light on the mechanisms of this promising hyperpolarization technology and points to potential routes for its improvement.

Overall, the manuscript is well written and clearly presented. My main concern is that the data presented consists of many details that, while very important for the SABRE community, may be lost on the general readership of Nature Communications. It may potentially find a more receptive audience in a specialized magnetic resonance or chemistry journal.

Author reply:

We thank the reviewer for their careful evaluation and positive remarks on the clarity and presentation of our work. We appreciate the concern regarding the accessibility of some of the detailed SABRE-specific data for a broader readership.

In response, we have revised the manuscript to improve readability for a general audience, providing clearer explanations of the key concepts, experimental approaches, and broader implications of our findings. These revisions aim to highlight the significance of our study beyond the specialized SABRE community, while retaining the essential mechanistic insights and experimental details.

In any case, there are also several apparent errors or omissions that should be addressed:

1) (page 6) Figure 4. The depicted calculated structure of complex 2 appears to only have one DMSO ligand and may therefore be incorrect. (The optimized structure of complex 2 in Fig. S26 does however, correspond more closely to the structure for 2 shown in Figs 1 & 2.

Answer: We thank the reviewer for carefully noting this point. The structure originally shown for *complex 2* in Figure 2 was indeed incorrect, it corresponded to the previously assigned *complex 4* rather than the intended bis-DMSO *complex 2*. We have now corrected Figure 2 to display the proper calculated structure of *complex 2*, consistent with the optimized geometry shown in Figure S26.

Modified figure:

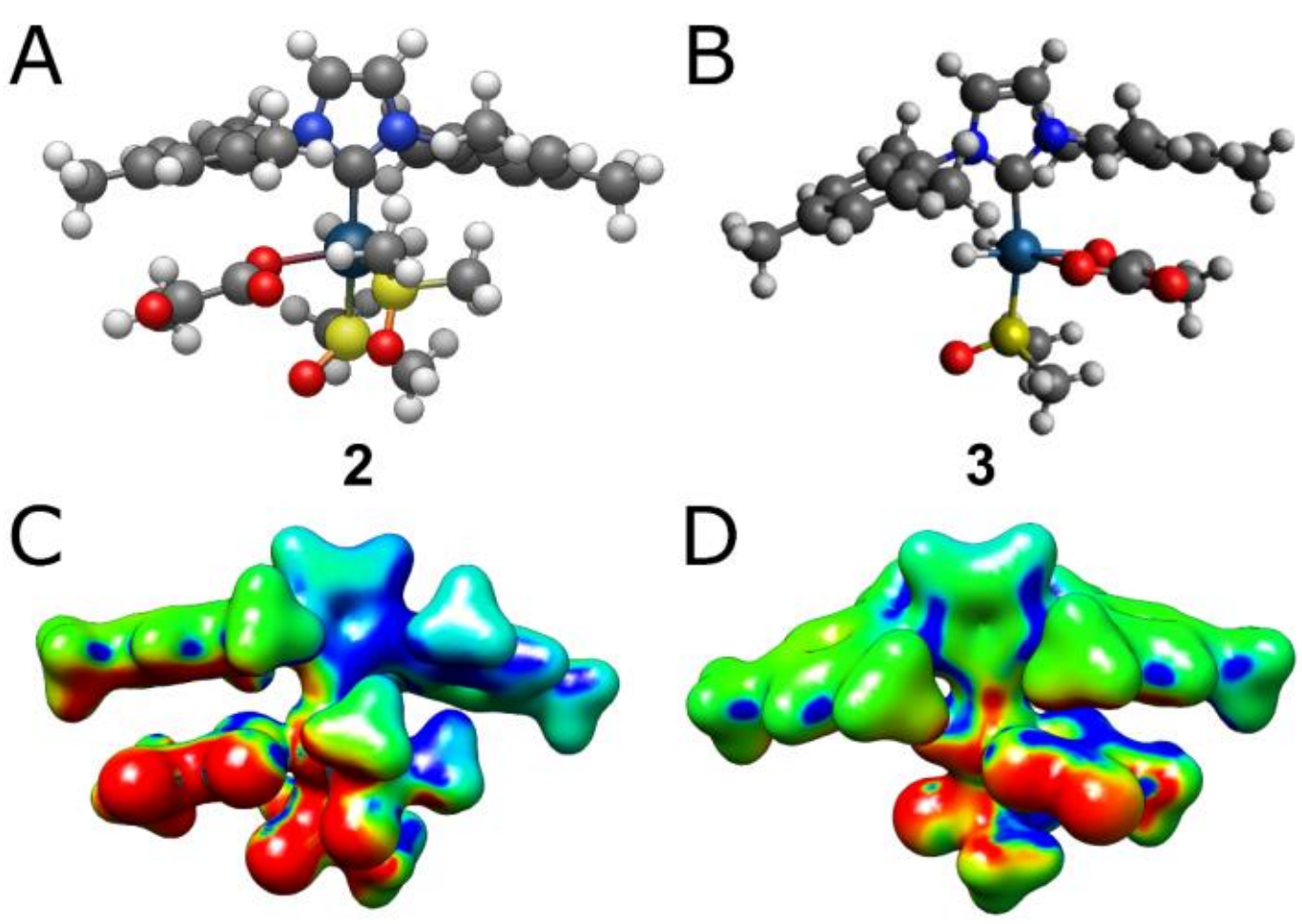

2) (page 3) "...transition from bidentate to monodentate binding (k3-κ1-κ2-pyr)" does not agree with the definitions in Figure 2. Please either swap the bi- and mono- or change the rate to k3-κ2-κ1-pyr.

Answer: We thank the reviewer for bringing this mistake to our attention. It was corrected. We also introduced complexes 5 and 6 which should simplify the flow.

Original text: pyr transition from bidentate to monodentate binding (k_3-κ^1-κ^2-pyr).

Modified text: or pyr transition from bidentate to monodentate binding ($k_{3\to6}$) and back ($k_{6\to3}$).

3) (page 6) "We acknowledge that a Thrippleton-Keeler zero-quantum filter based on a chirp pulse with a gradient (Fig. 5A) (ref61) could be used to improve coherence selection efficiency further". Fig. 5A appears to show such a filter with a chirp pulse and simultaneous GPZ0 gradient in the SEPP-SABRE sequence. Is the depicted sequence correct, and was a Thrippleton-Keeler indeed used?

Answer: We were thinking it would be useful for followers, but since it is confusing, we removed it from the figure and modified the text.

Modified text:

A simple two-step phase cycle and dephasing gradients were used to improve background signal suppression, with a 180° phase shift of the first pulse and the receiver to suppress signals unaffected by the frequency-selective first pulse (**Fig. S2B**). No additional filters[66] were used to enable the observation of the early exchange, and because remaining zero-quantum terms do not contribute to the total integral[37] (**Fig. 5B)**. It is also important that relaxation is taken into account in the data fitting process, as the T_1's of the interchanging sites may be significantly different from each other.

4) A similar effect of [DMSO] on the formation of complexes 2 and 3 has recently been described in Mamone et al. (doi:10.1039/D5CP01773D), albeit with the old assignment of complex 4 for complex 2. It would be worth acknowledging and citing this prior work.

Answer: We thank the reviewer for this helpful observation. We could not know about this work as it was published after manuscript was submitted. Still, the work is cited now in several places. We guess that the reviewer is referring to this passage of the work "By increasing the DMSO equivalents from 3 to 20, we observe a decrease in the population of the equatorial form (**3b**) and a corresponding increase in the axial form (**3a**) for both IMes (**1a**) and IPr (**1b**) catalysts across the temperature range of 240–310 K (see Fig. SI-18). " If you check Fig SI-18, then you would see that it is hyperpolarization yield weighted data, so populations and obtained signals are not easily correlated.

Therefore, we have now added a citation to Mamone *et al.* in the relevant section and clarified how their observations relate to, but do not duplicate, the findings presented here.

Modified text: Thus, we further confirm the revised identity of **2**, whose hydride ligand chemical shift signals were found not to change with increasing [DMSO] (**ESI**, **Fig. S19**). A related DMSO-dependent change in observed ^{13}C hyperpolarized signal amplitudes was independently reported by Mamone et al.[43], although without analysis of equilibrium populations or reassignment of complex identities. Given the modified structures and

associated chemical exchange diagrams (**Fig. 2**), the dependency on [DMSO] becomes self-evident (**ESI, Section 6**).

5) While evidence is shown to support complex 2 containing two DMSO ligands, does the 1-13C pyruvate chemical shift of complex 2 also support this new assignment of complex 2 instead of complex 4? Given that complex 2/4 has been assigned by the 13C chemical shift, it would further strengthen the new assignment.

Answer: Our current evidence indeed supports that complex **2** contains two DMSO ligands rather than one. The same then applies to 1- and 2-13C signals of pyruvate, which were previously assigned to 4, and should be assigned to 2, as evidenced by their prior correlation with the corresponding hydride protons.

If we understand the question correctly, you are asking if we have any ideas on the chemical shifts of 4. The answer is no. DFT is still not quantitative for 13C chemical shift prediction, and we still lack experimental evidence for 4.

6) While Na+ is attributed to have a substantial effect on the relative free energies of the complexes, is there any experimental evidence for this beyond the fact that SABRE works with sodium pyruvate but not with pyruvic acid?

Answer: Experimental analysis forced us to revise of DFT calculations and the structures, which were then aligned only after Na+ was explicitly added. In SI, we discuss different compositions of the complex. There is an example of the addition of NaCl to the solution, which was detrimental to polarization yield, but we are convinced that it is simply because of overpopulation of complex 1 instead of due to the excess presence of Cl in the solution.

Modified text:

Electronic structure calculations at the B3LYP-D4/def2-TZVP CPCM(Methanol) level of theory[54–62] yielded relative Gibbs free energy differences of +7.2 and +21.5 kJ/mol for **3** and **4** with respect to **2** (**Fig. 4** and **ESI, Section 18**; using the ORCA 6 program package[63,64], see ESI for details). This is in agreement with the experimentally evaluated thermodynamic parameters presented above, which indicate that **2** is favorable over **3** in energy by about 5 kJ/mol. The computational findings and the DMSO concentration dependence could not be explained if **4** was a thermodynamically accessible species. However, upon studying the relative energies and electronic structure of the complexes, it was found that the experimental trends in stability can only be reproduced when the computational models explicitly include Na^+ as a counterion. Note that implicit solvation models are known to exhibit larger errors in the case of strong specific interactions with solvent molecules or counterions. In this case, the electrostatic potential of **2** (**Fig. 4C**) especially indicates strong counterion interactions. In addition, SABRE presumably works only with sodium pyruvate as originally proposed, not with pyruvic acid, for which SABRE has not been shown to work so far, which is another clear indication of the critical role Na^+-pyr pairing plays in complex stabilization.

Minor points:

7) (page 1) The abbreviation pH2 is introduced without being defined.

Answer: Thank you. The missing word was added.

Modified text: Parahydrogen (pH_2)-based hyperpolarization methods offer faster and more cost-efficient alternatives.

8) (page 4) "were revealed using HMQC for DMSO with naturally abundant 13C (ESI, Fig. S19)". Fig. S19 shows a 1H-1H NOESY spectrum rather than HMQC.

Answer: We thank the reviewer for bringing this mistake to our attention. It was corrected. The right reference is figure S20 instead of figure S19

Modified text: The first key evidence came from NOE 2D NMR. We investigated a sample containing IrCl(COD)(IMes), DMSO-h_6, and [2-^{13}C]pyruvate in methanol-d_4 at 253 K after exposure to H_2. ^{1}H-^{1}H NOE revealed cross peaks between hydride protons of **2** (δ –14.98 and –24.09 ppm) and three methyl groups of DMSO (^{1H}δ 3.37, 2.98, and 2.92 ppm, **ESI**, **Fig. S19**). Due to the low hydride-ligand symmetry, a single DMSO ligand would produce two connections to the hydrides, whereas our measurements with three peaks are indicative of two DMSO ligands.

Then we performed two additional experiments: HMQC (heteronuclear multiple-quantum correlation)[50] ^{1}H-^{13}C correlation 2D NMR (**ESI, Fig. S20**) and ^{1}H-^{13}C SEPP-SPINEPT (frequency-selective excitation of parahydrogen-derived PASADENA polarization followed by frequency-selectively pulsed insensitive nuclei enhanced by polarization transfer, where PASADENA refers to high-field pH_2 experiment – pH_2 and synthesis allow dramatic enhancement of nuclear alignment[51], **ESI**, **Fig. S21**) that transfers polarization from pH_2 to neighboring carbons[44], but now using [U-^{13}C]DMSO.

9) (page 5) "The role of Na+ ... which is described in the next chapter". Perhaps "section" is more appropriate than "chapter".

Answer: We thank the reviewer for bringing this suggestion. The suggested word was added.

Modified text: which is described in the next section, may explain this effect.

10) (page 7) "...rate of conversion to 1 were found to reduce as [H2] increased...". "to decrease" or the like instead of "to reduce".

Answer: We thank the reviewer for bringing this suggestion. The suggested word was added.

Modified text: the rate of conversion to **1** were found to decrease as [H_2] increased

11) (page 8) "Interestingly, flux $k\mathbf{3}\rightarrow\mathbf{2}$ growed rapidly with [DMSO] ". "grew", or better, "increased" instead of "growed".

Answer: We thank the reviewer for bringing this mistake to our attention. It was corrected.

Modified text: Interestingly, flux $k_{\mathbf{3}\rightarrow\mathbf{2}}$ increased rapidly with

Reviewer 3:

In this article, the authors report novel understanding of an iridium SABRE catalyst's role in hyperpolarization of pyruvate through a combination of thermal equilibrium NMR experiments, hyperpolarized NMR, and computational studies using DFT calculations. In their hyperpolarization experiments, the authors use frequency-selective excitation sequences to perform chemical exchange measurements of protons. The manuscript reports a number of new findings. The authors convincingly show that a previously-described catalyst species is in fact not present in solution, and provide a wealth of new data on ligand and complex exchange dynamics. The authors performed electronic structure calculations using the ORCA 6 program package to support their experimental findings. These computational studies have revealed the importance of sodium ions for catalyst stability, which was not previously known. A key conclusion of this work is that the catalyst system, while effective, is not suitable for polarizing high concentrations of pyruvate. This is an important revelation, since the goal in many hyperpolarization experiments and applications is to maximize the concentration of hyperpolarized target in solution.
The only weakness is that the new knowledge that has been acquired is not then applied to improve the polarization level or concentration of polarized pyruvate. For example, by use of alternative ligands, or inclusion of additional/alternative counterions for catalyst stability. No concrete suggestions are given, and it is left up to future studies to optimize these outcomes. Nevertheless, this is a fantastic piece of work and I congratulate the authors on this carefully-conducted and thorough study that will be important for the research field.

Author reply:
We thank the reviewer for their detailed and encouraging assessment of our work. We greatly appreciate the recognition of the novelty and thoroughness of our study.
We acknowledge the comment regarding the application of our findings to improve polarization levels or pyruvate concentration. While the current study was focused on elucidating the mechanistic details of the catalyst system, our results provide a foundation for future work to optimize hyperpolarization efficiency, including the exploration of alternative ligands. We have clarified this point in the revised Discussion. At the same time, we are convinced that by spreading the information, methodology, and key features of the system, together with other research groups, we could find a more optimal ligand–co–ligand configuration.

I have a few specific suggestions for the authors to consider:

1) The authors show that there is rapid intramolecular hydride exchange in complex 3, and in the conclusions, they say that rapid exchange of the hydride ligands averages J-couplings hinders polarization transfer in complex 3. But Fig. S21 that is pointed to, does not seem to show that. If the authors have quantitative measurements of intramolecular hydride exchange (Fig. 5), is it not possible to quantitatively predict the effect on pyruvate 13C polarization via simulations? Showing that this mechanism limits pyruvate polarization would be an important result in the manuscript, and if it is a negligible effect, that would still be worth showing.

Answer: We thank the reviewer for pointing this out. The correct figure reference is Fig. S22 (not S21). In the SI, we explicitly simulate the effect of intramolecular hydride exchange on SLIC-SABRE polarization of [1-13C]pyruvate. These simulations show that intramolecular exchange of the two hydride protons in complex **3** reduces polarization transfer efficiency. The model is greatly simplified and includes only the most essential steps.

The model assumes that only H_2 exchanges while the substrate remains bound, which is typical for **3** at low temperatures. Therefore, the detrimental effect of hydride exchange on pyruvate polarization is quantitatively demonstrated in Fig. S22. The true impact can be calculated when each step is included in the simulation, but currently, some exchange steps are not yet quantified.

Modified text: In the extreme, the two become equal; the pH_2 spin order cannot be converted into polarization of the target nucleus (**ESI**, **Section 16**, **Fig. S22**).

Section 16, SI:

For example, for k_d = 10 s^{-1}, predicted polarization is falling from 67.2% to 43.7% (or by 35%) when intramolecular exchange increases from 0 to 10 s^{-1}. For 3, the k^{HH}_{3ex} ranged between 2 and 7 s^{-1} in the studied temperature range.

2) The title is over-reaching and vague, and should be modified: this article has certainly shed new light on this catalyst and provides important new results, but the title should reflect more precisely the work reported in the manuscript.

Answer: Thank you for your interest. Yes, we followed the recommendation and

Original title: Reshaping the understanding of magnetic resonance signal amplification by reversible exchange of pyruvate

Modified title: Reshaping and quantifying inter- and intramolecular exchange in signal amplification by reversible exchange of pyruvate

3) In the "DMSO concentration dependency" subsection, I found the discussion regarding the "revised identity of 2" to be confusing, and this pops up a few times in the paper. As I understand, the identity of complex 2 is not revised, but rather the authors have determined its existence, and excluded the existence of complex 4.

Answer: Yes, you are correct. We revised the text substantially; hopefully current state is more transparent.

Example of Modified text:

As was noted above, based on the experimental and theoretical evidence supported by DFT calculations (**ESI, Fig. S24–29**), we have found that complex **4** (**Fig. 1**) could not play a significant role in pyruvate SABRE and ruled it out from the list of key intermediates of this system in **Fig. 2**. Instead, we prove the existence of complex **2** as one of the key species for pyruvate SABRE along with complexes **1** and **3**.

4) In the "Pyruvate concentration dependency" subsection the authors say that the increasing concentration of 3 relative to 2 is not expected from the chemical model, but it's not obvious to me why that is. Further clarification would be helpful here.

Answer: Thank you for raising this question. It is not expected, as both complexes contain one pyruvate ligand each. Increasing the pyruvate concentration, therefore, should not favor the formation of **3** over **2**.

Original text:

Surprisingly, the concentration of **3** also increases relative to **2** as a function of [pyr], which was not expected based on the presented chemical model (**Fig. 2**).

Modified text:

Surprisingly, the concentration of **3** also increases relative to **2** as a function of [pyr], which was not expected based on the presented chemical model (**Fig. 2**). This is because, according to their chemical structures, both **2** and **3** contain one pyr ligand; hence, changes of [pyr] should not favor one of them. The observed trend, therefore, indicates that the system behaves more intricately than the simple model predicts.

5) The authors state that complex 3 has low stability in solution because the sample degrades, but data is not included to show this. In parallel, they have shown that complex 3 is the most important catalyst species for polarization of pyruvate, so quantification of its degradation would be useful data to include.

Answer: Thank you for raising this question. Likely you are referring to this passage: "Utilization of pH_2 and SEPP allowed us to enhance, for example, H^a or H^b proton signals of **1** at 265 K by ~8060-fold at 9.4 T ([DMSO]= 75 mM, [Ir] = 4 mM, [pyr] = 40 mM), dramatically accelerating data collection proved to be critical because of the low stability of **3** (the samples degrades after preparation).[64] " We did not study this ourselves as we used low temperatures where degradation is much slower and therefore cited the work. Instead, we tried to keep the sample at a low temperature and freshly prepared to reduce the potential impact from sample degradation.

Comments of the reviewers and corresponding answers

Reviewer #1 (Remarks to the Author)

I wish to thank the authors for taking my feedback into account. I found the manuscript significantly improved. The motivation is clearer and the introduction reads better in the new version.

However, I still believe that the second half of the results section is not properly justified. The reported data are surely relevant and useful for the parahydrogen community, but how do they contribute to the story of this paper? As an example, the section on H2 exchange starts with "The effect of [H2] on these processes was evaluated over the range of 1 to 8.5 bar." Why was this measurement performed? How does it contribute to reshaping the understanding of SABRE? Although the manuscript was improved, the question of why a measurement is needed still needs to be answered in multiple places.

Author reply:

We thank the reviewer for this valuable comment. We agree that the motivation for varying specific experimental parameters may not have been sufficiently explicit in the original manuscript.

To elucidate the chemical mechanism of the SABRE process, it is necessary to systematically vary key parameters influencing SABRE kinetics. As SABRE is inherently a multiparametric process, we explored a range of conditions to disentangle the contributions of the different factors involved. In this context, the variation of H_2 pressure was performed to probe the role of hydrogen concentration on SABRE kinetics.

Therefore, we are convinced that all sections are justified. One can make an educated guess in some cases, but we do not think that it is better than experiments, e.g., as the experiments in our case (see DMSO study), and always bring surprises.

Modified text:

H_2 exchange: The effect of [H_2] on these processes was evaluated by changing pH_2 pressure over the range of 1 to 8.5 bar. This pressure change, and as a result [H_2] change, led to a progressive increase in the degree of visible H_2 loss, flux $k_{3\text{-}H2}$ from **3** (**Fig. 5F**), thereby confirming that these exchange processes occur by H_2 addition to **5** (i.e. also S_N2 mechanism as with [Ir(H)$_2$(IMes)(pyridine)$_3$]Cl-type systems), accessed by κ^2-pyr to κ^1-pyr rearrangement.

Detailed comments

1) The choice of tenses of verbs needs to be improved. A common choice is the present tense in the introduction and methods and the past tense in the results. In the current manuscript, it goes back and forth between tenses creating a confusion regarding where the authors describe their overall methods, past results, and their current results. Example: In the introduction paragraph that starts with "Given the significant level of contradiction and the importance of […]", the authors use "we have shown" and "we observe". I believe that the present tense would make it clearer to the reader that this summarizes the findings of this paper.

Answer: The introductory section was checked for the improvements of tenses.

Original text:

1- we have shown that the previously proposed complex 4 (Fig. 1) is not observed and should be excluded
2- we observed a pronounced *intramolecular* hydride ligand exchange in
3- we undertook DFT computational analysis of
4- The resulting data enabled the determination of

Modified text:

1- we show that the previously proposed complex 4 (Fig. 1) is not observed and should be excluded
2- we observe a pronounced *intramolecular* hydride ligand exchange in
3- we undertake DFT computational analysis of
4- The resulting data enable the determination of

2) "However, previous density functional theory (DFT) predictions indicated that isomer 4 has a Gibbs free energy 7.6 kJ/mol higher than isomer 344. Such a significant energy difference implies much lower equilibrium concentrations of 4 than of 3, contradicting our experimental observations." Am I correct in saying that the authors state a contradiction with results they haven't presented yet? This makes this paragraph difficult to follow.

Answer: We thank the reviewer for this valuable comment. We agree that presenting detailed experimental observations in the Introduction may not be clear. We have therefore revised this paragraph to improve the logical flow by removing specific experimental details and instead providing a forward reference to the Results section.

Original text: However, previous density functional theory (DFT) predictions[43] indicated that isomer 4 has a Gibbs free energy 7.6 kJ/mol higher than isomer 3. Such a significant energy difference implies much lower equilibrium concentrations of 4 than of 3, contradicting our experimental observations**.**

Modified text: (now in two different paragraphs)

…These included *bis*-DMSO complex [Ir(Cl)$(H)_2(DMSO)_2$(IMes)] (**1**),[42] and [Ir$(H)_2$(κ^1-κ^2-pyr)(DMSO)(IMes)], which was initially proposed[22] to exist as two isomers where pyruvate binds in the equatorial plane (**3**) or bridges equatorial-axial sites (**4**) (**Fig. 1**). Previous density functional theory (DFT) predictions[43] indicated that isomer **4** has a Gibbs free energy 7.6 kJ/mol higher than isomer **3**. Such a significant energy difference implies much lower equilibrium concentrations of **4** than of **3**.

Herein, we show that our experimental results clearly contradict the previous assumptions, revealing significant shortcomings in the existing mechanistic model. In particular, we found that the ^{1}H and ^{13}C NMR signals, previously associated with complex **4** in the literature,[22,27] can grow to levels exceeding those of **3** as the DMSO concentration increases or as the temperature decreases, highlighting the significant flaws in the existing mechanistic model.

Given the contradiction and ….

3) The SEPP-SPINEPT applied to hydrides of 2 yielded four hyperpolarized carbons of this isotopomer of DMSO" I see more than 4 hyperpolarized 13C signals in the SI figure

Answer: Thank you for this comment. In the SI figure, only four ^{13}C signals correspond to the hyperpolarized DMSO isotopomer obtained after applying SEPP-SPINEPT to the hydrides of complex **2**. These signals appear in antiphase, as expected for SEPP-SPINEPT transfer. The additional resonances observed in the spectrum arise from methanol and free DMSO present in the sample. To avoid confusion, the hyperpolarized signals as well as the free DMSO contribution have now been explicitly marked in the revised Supporting Information.

Modified SI Figure:

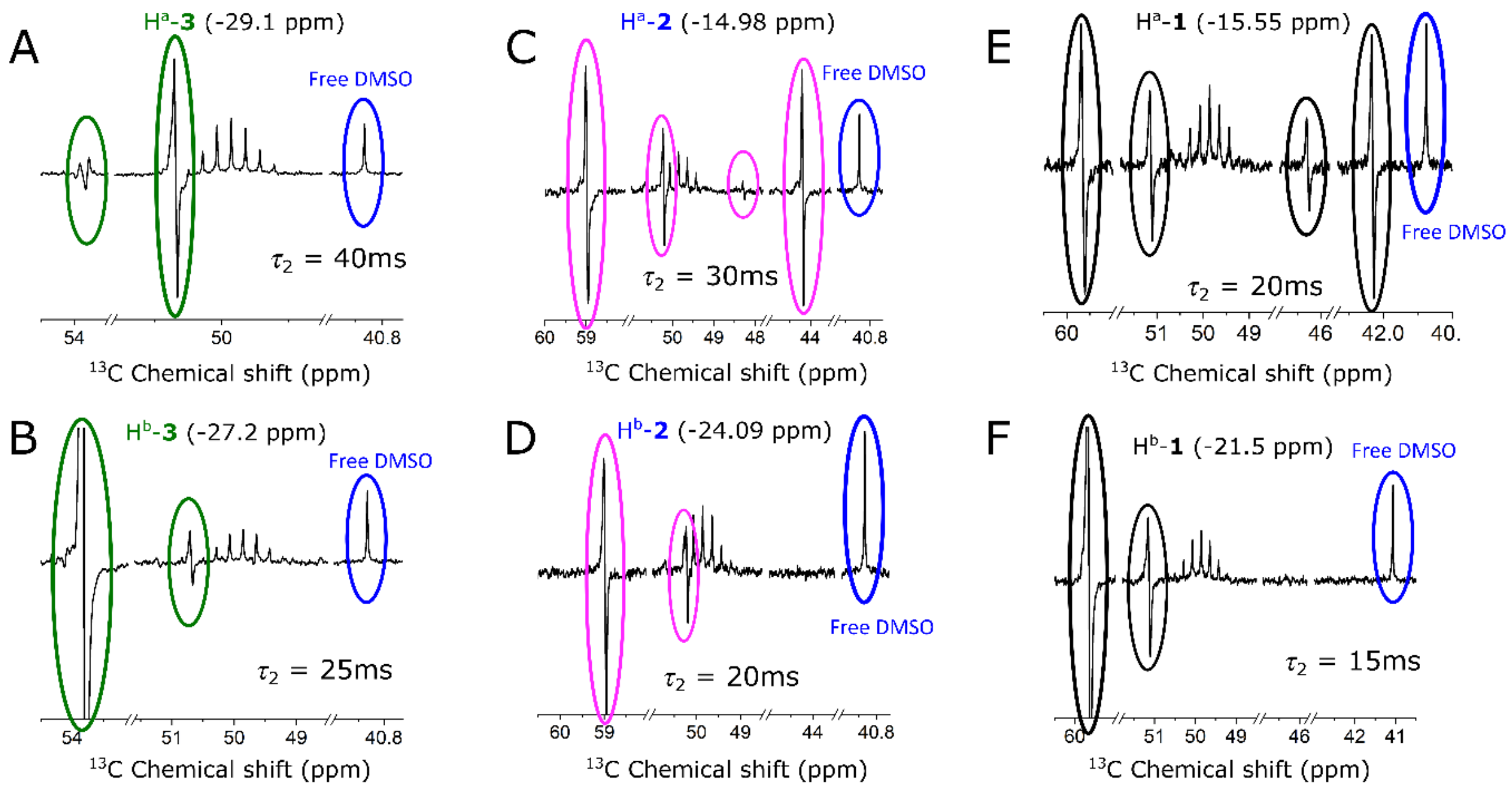

Modified caption: Phased NMR spectra at a maximum of polarization of the absolute intensity ^{13}C NMR signals of bound DMSO after SEPP-SPINEPT at 263K. (A) This NMR spectrum shows polarization transferred from H^a-**3** (-29.1 ppm) to the bound ^{13}C-DMSO of **3,** while (B) shows polarization transferred from H^b-**3** (-27.2 ppm) to the bound ^{13}C-DMSO of **3.** (C) The spectrum shows polarization transferred from H^a-**2** (-14.98 ppm) to the bound ^{13}C-DMSO of **2,** and (D) shows polarization transferred from H^b-**2** (-24.09 ppm) to the bound ^{13}C-DMSO of **2.** (E) The spectrum shows polarization transferred from H^a-**1** (-15.55 ppm) to the bound ^{13}C-DMSO of **1,** and (F) shows polarization transferred from H^b-**1** (-21.5 ppm) to the bound ^{13}C-DMSO of **1.** The peak at 41 ppm corresponds to free DMSO in the sample. The multiplet (septet) at 50 ppm is assigned to the methanol.

4) The observed hydrogen dissociation transmission rate for 1 is displayed as k1-H2 and involves several steps, while only the initial and final steps are observable." Initial reagent and final product might be easier to understand.

Answer: We thank the reviewer for the suggestion. We have revised the sentence to improve clarity by specifying the "initial reagent" and "final product" instead of "initial and final steps."

Original text: The observed hydrogen dissociation transmission rate for **1** is displayed as $k_{1\text{-}H2}$ and involves several steps, while only the initial and final steps are observable.

Modified text: The observed hydrogen dissociation transmission rate for **1** is displayed as $k_{1\text{-}H2}$ and involves several steps, while only the initial reagent and final product are observable.

5) (Remarks on code availability)

The link does not contain any code. In contains experimental data (together with pulse sequence files). It does not contain a readme file.

Answer: Thank you for this remark. We apologize for the oversight. The code has now been uploaded to Zenodo, along with a README file describing its contents and usage. The DOI has been updated accordingly in both the manuscript and the Supporting Information. The new DOI is **10.5281/zenodo.18449765**, which replaces the previous incomplete upload (**10.5281/zenodo.16746641**).

Reviewer #2 (Remarks to the Author)

The authors have addressed my comments, and, in addressing the critique of Reviewer 1, the manuscript is much improved in its presentation and narrative structure. Some of the changes should, however, be further revised to make the manuscript suitable for publication.

Author reply: We thank the reviewer for their careful reading and positive feedback on the improvements made to the manuscript's presentation and narrative. We have carefully addressed all remaining suggestions to further improve clarity, and believe the revised manuscript is suitable for publication.

1) The revised title makes less sense than before. With "Reshaping ... exchange", how does one reshape exchange? Perhaps the comment from Reviewer 3 can again be taken in consideration to reformulate a title that more accurately summarizes the work and its significance.

Answer: We thank the referee for this suggestion and agree that the original title may not have been optimal. We hope that the revised title is more representative of the scope of our work.

Modified title: Mechanistic Insights into Chemical Exchange during the Signal Amplification by Reversible Exchange Sensitization of Pyruvate

2) The revised Figure 2 is busier and less clear than the prior version. The additional species shown are helpful for understanding the various pathways, but less so for the the long coloured arrows and especially the crowding. Perhaps more space can be used and/or the repetitive presence of the IMes ligand omitted or abbreviated to make the key pathways easier for the reader to understand.

Answer: We thank the reviewer for this helpful suggestion. To improve clarity and reduce crowding in Figure 2, we have replaced the full structure of the IMes ligand with the abbreviation “IMes” throughout the figure. Some long arrows we find still important, as unfortunately, we do not always measure the rates, but transmission rates/fluxes across many elementary steps. Altogether, this retains the chemical information while making the key pathways and colored arrows easier to follow.

3) Additionally, should complex 6 have a net positive charge, and should the unnumbered complex with two pyruvates have a net negative charge?

Answer: We thank the reviewer for this comment. The formal charges of the complexes have been carefully reviewed and updated. Complex **6** has a net positive charge, and the unnumbered complex with two pyruvates has a net negative charge, consistent with the chemical composition and coordination environment of each species.

Original Figure:

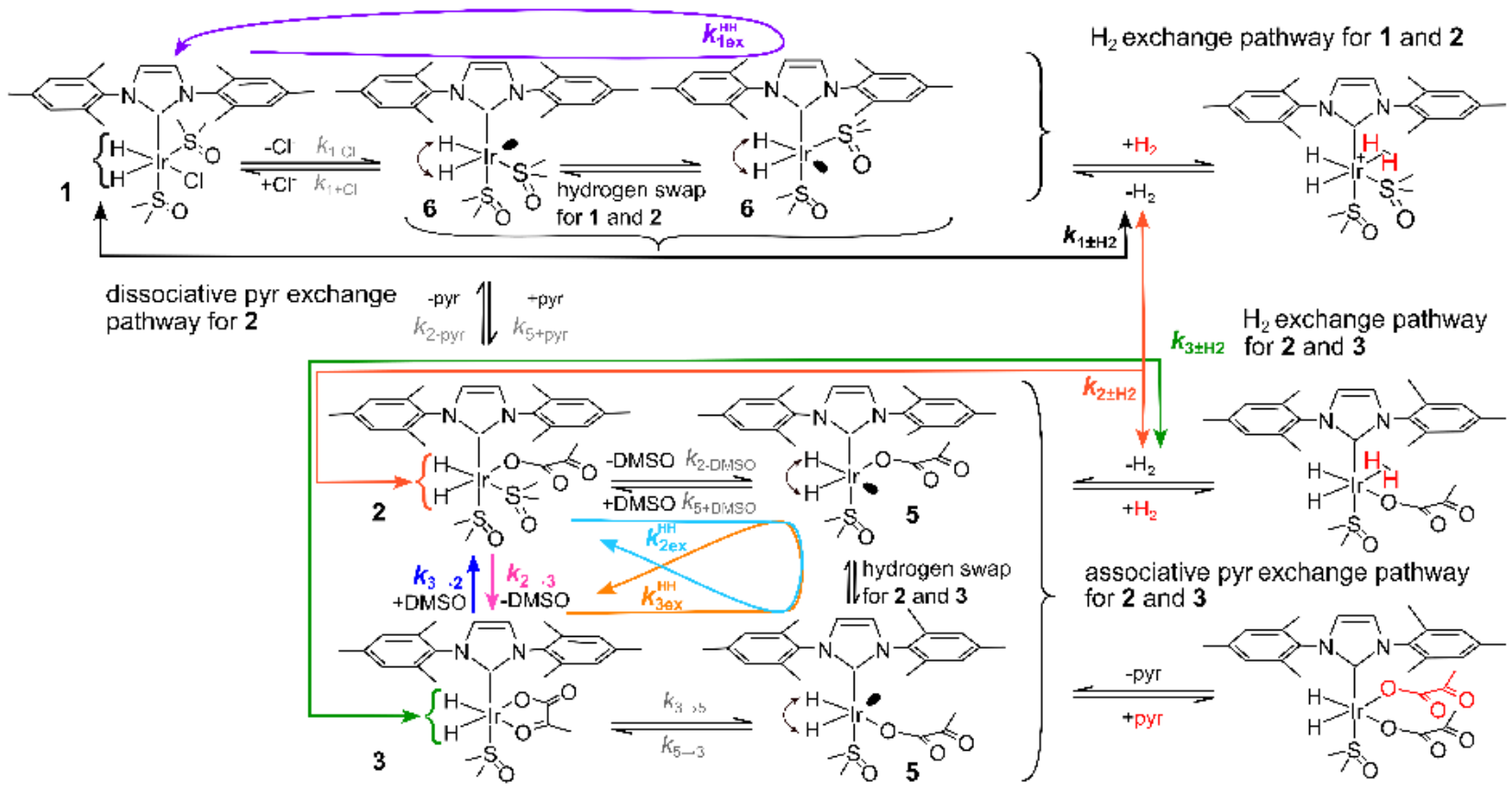

Modified Figure:

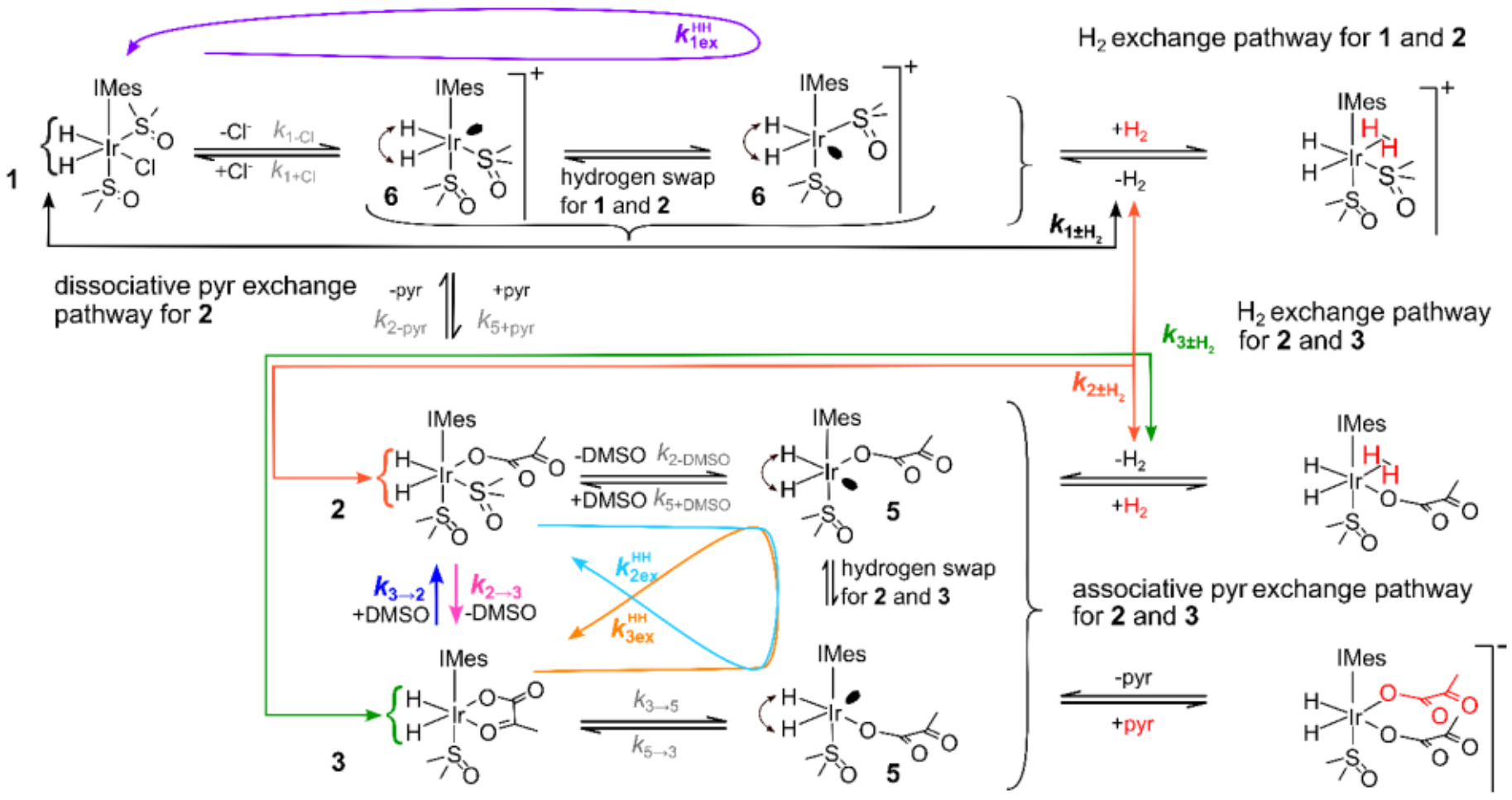

4) "Single-barbed", "equilibrium" or "half-headed", rather than "harpoon-like", are more conventional descriptions of the arrows for reversible chemical reactions. (both in Fig. 2 caption and main text)

Answer: We thank you for pointing this out. Fig. 2 caption and the main text were corrected by replacing “harpoon-like” with “single-barbed”.

Original text:

1. (Fig 2 caption) Elementary reaction steps are indicated with grey colored rates and harpoon-like arrows.
2. (main text) Care should be taken when viewing this figure, as it shows elementary reaction steps (grey rates with harpoon-like arrows) and transmission rates (not grey rates with regular arrowheads).

Modified text:

1. (Fig 2 caption) Elementary reaction steps are indicated with grey colored rates and single-barbed arrows.
2. (main text) Care should be taken when viewing this figure, as it shows elementary reaction steps (grey rates with single-barbed arrows) and transmission rates (not grey rates with regular arrowheads).

Minor points:

5) page 7: "dissociation of H2 via 3 occurs much faster than via 2, and can be inhibited by increasing [DMSO] or accelerated by increasing [H2] through competitive trapping of 5 (Fig. 5H, I)." Should that be Fig. 5F, rather than 5I?

Answer: We thank the reviewer for pointing out these minor grammatical issues. The manuscript has been carefully revised to correct them throughout the text.

Original text: Furthermore, the dissociation of H_2 via **3** occurs much faster than via **2**, and can be inhibited by increasing [DMSO] or accelerated by increasing [H_2] through competitive trapping of **5 (Fig. 5H, I**).

Modified text: Furthermore, the dissociation of H_2 via **3** occurs much faster than via **2**, and can be accelerated by increasing [H_2] (**Fig. 5F, G**) or inhibited by increasing [DMSO] (**Fig. 5H, I**) through competitive trapping of **5.**

The revised text also contains some minor grammatical or syntactical errors, but the following may particularly cause some confusion:

6) page 2: "which was initially proposed22 and then followed (e.g., recently43) to exist as two isomers". "followed ... to exist" sounds very strange; perhaps "and then followed (e.g., recently43)" can be removed if that citation is not essential here.

Answer: We thank the reviewer for pointing out these minor grammatical issues. The manuscript has been carefully revised to correct them throughout the text.

Original text: which was initially proposed[22] and then followed (e.g., recently[43]) to exist as two isomers where pyruvate binds in the equatorial plane (3) or bridges equatorial-axial sites (4) (Fig. 1).

Modified text: which was initially proposed to exist as two isomers[22] where pyruvate binds in the equatorial plane (**3**) or bridges equatorial-axial sites (**4**) (**Fig. 1**).

7) page 7: "Utilization of pH2 and SEPP allowed us to enhance, for example, Ha or Hb proton signals of 1 at 265 K by ~8060-fold at 9.4 T ([DMSO]=75 mM, [Ir] = 4 mM, [pyr] = 40 mM), dramatically accelerating data collection proved to be critical because of the low stability of 3 (the samples can degrade after preparation65)." Run-on sentence.

Answer: We thank the reviewer for pointing out these minor grammatical issues. The manuscript has been carefully revised to correct them throughout the text.

Original text: Utilization of pH_2 and SEPP allowed us to enhance, for example, H^a or H^b proton signals of 1 at 265 K by ~8060-fold at 9.4 T ([DMSO]= 75 mM, [Ir] = 4 mM, [pyr] = 40 mM), dramatically accelerating data collection proved to be critical because of the low stability of 3 (the samples can degrade after preparation[65]).

Modified text: Utilization of pH_2 and SEPP allowed us to enhance, for example, H^a or H^b proton signals of **1** at 265 K by ~8060-fold at 9.4 T ([DMSO]= 75 mM, [Ir] = 4 mM,

[pyr] = 40 mM), which dramatically accelerated data collection and proved critical for the low stability of **3** (the samples can degrade after preparation[65]).

8) page 7: "No additional filters66 were used to enable the observation of the early exchange, and because remaining zero-quantum terms do not contribute to the total integral37 (Fig. 5B)." Sentence fragment.

Answer: We thank the reviewer for pointing out these minor grammatical issues. The manuscript has been carefully revised to correct them throughout the text.

Original text: No additional filters[66] were used to enable the observation of the early exchange, and because remaining zero-quantum terms do not contribute to the total integral[37] (**Fig. 5B**)

Modified text: No additional filters[66] were used to enable the observation of the early exchange, because remaining zero-quantum terms do not contribute to the total integral[37] (**Fig. 5B**)

9) page 9: "...was also quantified at high [DMSO] and again very close to the value measured via pyruvate polarization." – ...and was again very close...?

Answer: We thank the reviewer for pointing out this sentence. The paragraph was redesigned for simplicity.

Original text: the $k_{2\to3}$ flux values measured by probing hydride or pyruvate ligands were close to those observed here within errors; flux $k_{3\to2}$, measured by hydride ligand excitation, was also quantified at high [DMSO] and again very close to the value measured via pyruvate polarization. Increased [DMSO] proved to inhibit $2 \to 3$ conversion as expected through the [DMSO] dependent k_{5+DMSO} step.

Modified text:

Pyruvate-observed *inter*complex exchange: For the low [DMSO] of 20 mM, the flux $k_{3\to2}$ was 6-8 times lower than that of flux $k_{2\to3}$. At high [DMSO] (hundreds of mM), $k_{3\to2}$≈$k_{2\to3}$ (**Fig. 6H,I**). Both fluxes proved to be temperature-dependent, as expected. Interestingly, flux $k_{3\to2}$ grew proportionally to [DMSO], while flux $k_{2\to3}$ decayed slowly because high [DMSO] prevented pyruvate from binding back to form **3**. Hence, increased [DMSO] proved to inhibit $2 \to 3$ conversion as expected through the [DMSO] dependent k_{5+DMSO} step. This aligned with the observations made by SEPP-SABRE applied to hydrides and supported the identity of **2**: $k_{2\to3}$ and $k_{3\to2}$ flux values measured by probing hydride were within error of those observed here by probing pyruvate.

10) page 9: "...exchange between 2 and 3, is mediated by 5, is much slower..." - ...and is much slower...?

Answer: We agree that the original text was not sufficiently clear. The text has been revised accordingly to avoid misunderstandings.

Modified text (paragraph order was changed):

Pyruvate dissociation rate: The observed pyruvate dissociation flux at studied temperatures was always slower than the flux of *inter*complex exchange between **2** and **3** mediated by **5**. For example, at [Ir] = 4 mM, [DMSO] = 20 mM, [pyr] = 40 mM at 265 K, the fluxes $k_{2-\mathrm{pyr}}$ and $k_{3-\mathrm{pyr}}$ were (0.042 ± 0.004) s^{-1}, and (0.016 ± 0.001) s^{-1}, respectively, whereas the interconversion fluxes were $k_{3\to 2}$ = (0.121 ± 0.006) s^{-1} and $k_{2\to 3}$ = (0.630 ± 0.007) s^{-1}.

Pyruvate exchange can go via dissociative exchange and complex **6**, or via associative exchange and complex **5**. When it goes through **5**, the complex [Ir(H)$_2$(IMes)(κ^1-pyr)$_2$(DMSO)] is generated as a subsequent step; thus, the observed exchange should be [pyr]-dependent. This is in accordance with the observation of the acceleration of $k_{3-\mathrm{pyr}}$ with the increase of [pyr] (**ESI, Fig. S17G**). Hence, the addition of pyr could accelerate pyr exchange. At the same time, $k_{2-\mathrm{pyr}}$ is less impacted by [pyr] (**ESI, Fig. S17H**) than $k_{3-\mathrm{pyr}}$, indicating that for **2**, the dissociative exchange has a greater contribution than the associative exchange at 265 K and [pyr] up to 175 mM.

11)(Remarks on code availability)

Answer: Thank you for this remark. We apologize for the oversight. The code has now been uploaded to Zenodo, along with a README file describing its contents and usage. The DOI has been updated accordingly in both the manuscript and the Supporting Information. The new DOI is **10.5281/zenodo.18449765**, which replaces the previous incomplete upload (**10.5281/zenodo.16746641**).

Reviewer #3 (Remarks to the Author)

The authors have addressed all of my suggestions, and I recommend publication. (Remarks on code availability)

Author reply: Thank you for this remark. We apologize for the oversight. The code has now been uploaded to Zenodo, along with a README file describing its contents and usage. The DOI has been updated accordingly in both the manuscript and the Supporting Information. The new DOI is **10.5281/zenodo.18449765**, which replaces the previous incomplete upload (**10.5281/zenodo.16746641**).

Reviewer #4 - Remarks to Author

This work of Dr. Pravdivtsev and co-workers presents new insights on the formation of the iridium complex used in SABRE hyperpolarization of 13C-labelled pyruvate through NMR experiments and DFT calculations. Two key points of this study are the characterization of a key intermediate of the reaction pathway and the elucidation of the role of a sodium counterion in the stabilization of the Iridium complex intermediates in the reaction media. The overall manuscript is well written, and the findings are presented with clarity.

I focused my attention on the DFT results presented in this study, as the NMR experiments go beyond my expertise and have been thoroughly evaluated by the previous reviewers. The used computational method (B3Lyp-D4/de2tzvp) is adequate (and properly cited) and the general approach is suitable for the scope of the presented work, consisting in the calculation and comparison of Gibbs free energy of different structures to provide insight on the most stable species. Within the scope of this study, the presented DFT results play a key role in supporting the experimental observations. It is required for the computational data to be thorough and robust in order to assure the presented results are not derived from artifacts of the computational model, especially taken into account the explicit inclusion of Na+ in the model presented in this study. For this reason, I strongly believe that some additional calculations are required and that this work can become suitable for publication in Nature Communications if the following points are addressed:

Author reply: We sincerely thank the reviewer for the thorough and constructive evaluation of our manuscript, and for the detailed and fair criticism provided in the report. We would like to offer a brief general comment on the role of the DFT calculations within the broader context of this work. The experimental evidence remains the primary and most robust foundation of our findings, supported by 2D NMR, kinetic data, and concentration-dependent population measurements. We are confident in the solidity of these results. The DFT calculations were included to provide a rationalization of our experimental observations, particularly because the structures suggested by experiment were inconsistent with previously reported simulations. The explicit inclusion of Na^+ emerged as one plausible explanation for this discrepancy.

However, we fully acknowledge the reviewer's concern: the explicit modeling of Na^+ introduces additional complexity, and the system may indeed be too intricate for the used computational treatment to reliably assess the relative energetics of all relevant species. We agree that a more comprehensive computational investigation would be needed to definitively establish these energetics, and we have taken care to ensure that both the revised manuscript and the extended Supporting Information convey this limitation transparently, without overstating the conclusions drawn from the DFT results.

We are grateful for the reviewer's suggestions, which have meaningfully improved the rigor and clarity of the manuscript.

1. In the computational study section of the main text, the authors start by comparing the relative Gibbs free energies of structures 2, 3 and 4:

"Computational study: Electronic structure calculations at the B3LYP-D4/def2-TZVP CPCM(Methanol) level of theory54–62 yielded relative Gibbs free energy differences of +7.2 and +21.5 kJ/mol for 3 and 4 with respect to 2 (Fig. 4 and ESI, Section 18; using the ORCA 6 program package63,64, see ESI for details" However, in this section the authors do not present the relative energies of complexes 2, 3 and 4 shown in Figure 1. These energies correspond to the results obtained after the inclusion of a sodium ion in the model, which is presented in Figure S26 of the ESI. One issue is that the adaptation of the computational model by inclusion of a sodium ion is only discussed in detail in the ESI, and these structures containing a sodium ion are not shown at all in Figure 4. Another is that the relative energies of the structures presented in Figure 4-A,B are only mentioned in the figure's caption and not properly discussed in the main text. Therefore, the obtained results should be presented with more clarity and Figure 4 should be modified to show the structures matching the "Gibbs free energy differences of +7.2 and +21.5 kJ/mol for 3 and 4 with respect to 2" described in the text.

Answer: We thank the reviewer for this remark and agree that the computed free energies were presented with insufficient context and in a suboptimal manner in the original manuscript. In the revised version, we have restructured the computational section for clarity, and the relevant free energy differences are now featured prominently within a revised and extended Figure 4. We note that no energy differences involving Na^+ are discussed in the main text.

The rationale for its potential inclusion and the associated problems are discussed in the main text and ESI. The associated structural and energetic analysis are addressed in detail in the ESI, Section 18.2 ("Structure and Energetics of Compounds 2–4") and the surrounding subsections of Section 18.

The necessity of Na^+ was removed from the text, or the statements were softened.

Modified text (Energetics, main text):

Electronic structure calculations at the B3LYP-D4/def2-TZVP CPCM (Methanol) level of theory[54–62] using the ORCA 6 program package[63,64] yielded relative Gibbs free energy differences ($\Delta G_{50\%}$,[65–67] see **ESI, Section 18** for details, **Table S18**) of +9.1 and +15.8 kJ/mol for **3** and **4** with respect to **2** (**Fig. 4**). Within the error bars of DFT, this is consistent with the experimentally evaluated thermodynamic parameters presented above, which indicated that **2** is energetically favorable over **3** by about 5 kJ/mol.

Modified text (Na^+, main text):

Upon studying the relative energies and electronic structures of the complexes, we found that the electrostatic potentials of **2**, **3**, and **4** (**Fig. 4**) are particularly indicative of potentially strong counterion interactions. We found that their relative stabilities are strongly influenced by the explicit addition of species such as Na^+. Note that implicit solvation models are known to exhibit larger errors in the case of strong

specific interactions with solvent molecules or counterions. We also checked the impact of Na^+ because SABRE presumably works with sodium pyruvate, as originally proposed, rather than with pyruvic acid, for which SABRE has not yet been shown to work, providing another experimental indication of the possible role Na^+-pyr pairing plays in complex stabilization. Here, we found that the complex-counterion interaction with Na^+ is strongest for **2** (ΔE = 144.1 kJ/mol), followed by **3** (ΔE = 116.8 kJ/mol) and **4** (ΔE = 113.0 kJ/mol), in agreement with the electrostatic potential assessment. For a series of simple adduct models (complex with Na^+, methanol, or DMSO, **ESI**, **Fig. S26**), this can lead to changes in the relative free energies of more than 10 kJ/mol. While these preliminary results should not be regarded as quantitative, they hint at the great potential of this influence, and further detailed experimental and computational studies of the ions' effects are underway in our group.

Added text in ESI:

From the resulting free energies, the pyruvate complex **3** is the lowest energy structure - in contrast to what is found in experiment ($\Delta G^0_{2\leftarrow 3}$ = -5.3 kJ/mol in favor of **2**) - and the most stable compared to the other species, implying that **2** and **4** should not be observed at all (see ΔG in **Fig. S24**). For the electronic energies (**ΔE** in **Fig. S24**), however, **2,** which contains an additional DMSO, appears much more stable than **3** and **4**. This situation is often observed for Gibbs free energies of non-unimolecular reactions in solution. In the literature, it is well documented that in these cases the common entropy corrections contained in Gibbs free energies strongly overestimate the translational entropy [65FaliveneMolCat]. We will refer to this computational scheme as **CS1**.

Therefore, two additional computational schemes (**CS2** and **CS3**) have been applied in computing the relative free energies of **2**, **3**, and **4**, following the suggestions of Falivene et al. Ref [65FaliveneMolCat].

CS2: We computed the $\Delta G_{50\%}$ values, in which the entropy correction for all species was scaled by 0.5 (Ref. 66GarzaJCTC, 67YuJACS).

CS3: We computed the ΔG_{assoc} values, for which the standard entropy corrections were used, but the dissociating DMSO molecule was kept associated with the reacting complex in the second coordination sphere (see Fig. S25 for geometries). For the latter, translational entropy corrections mostly cancel.

The results with respect to compound **2** are shown in **Table S18**.

2. In Figure 4, the presented electrostatic potentials (computed for C and D) must contain the corresponding colour-coded scale. This scale is also missing in Figure S25 in the ESI.

Answer: We thank the reviewer for pointing this out and have added the scale bar to all electrostatic potentials shown in the manuscript. As all 3 complexes are now in the main text, the corresponding figure from ESI was removed.

Modified Figure:

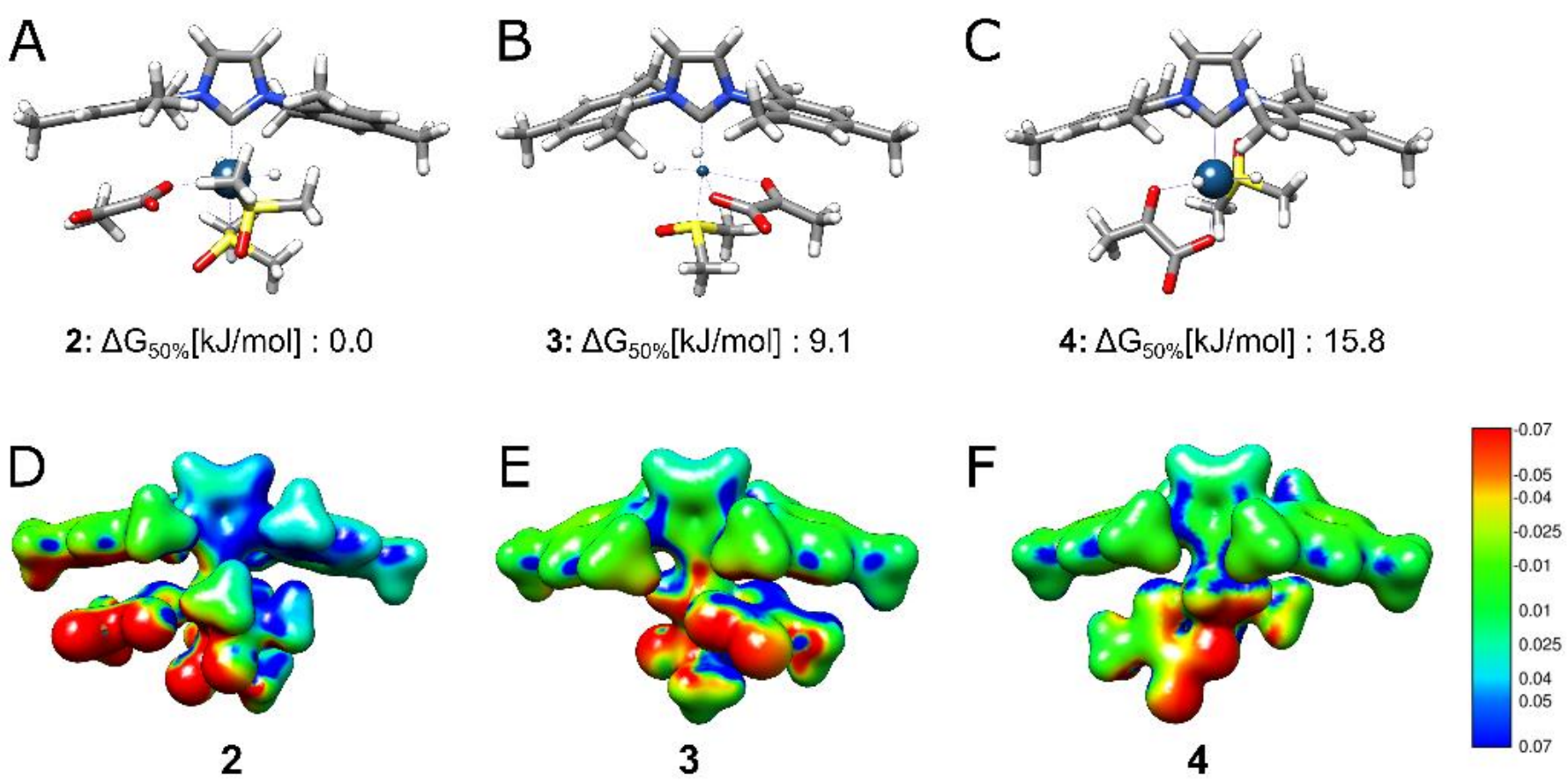

3. In the "Electronic Structure Calculations" of the ESI, there is no mention of conformational search by the authors. While the studied Iridium complexes might be somewhat rigid, the explicit inclusion of Na+ in the computational model requires a conformational search of the system, so the most stable interaction of the Iridium complex with the cation can be obtained as a global minimum (which could require the rotation of Ir-S(DMSO), Ir-O(pyr) and C-C(pyr) bonds).

Answer:

Indeed, the complex is fairly rigid and while we have not carried out an extensive conformational search of the system with algorithms like CREST or GOAT due to the required computational effort (and the deficiencies of xtb for transition metal complexes) we have carried preliminary calculations assessing different structures and choosing the most stable one for further optimizations. This is now mentioned explicitly in the computational details in the SI.

4. The authors present in the ESI a comparison of Gibbs free energies between 2, 3 and 4 (Figure S24) and the observation that 4 is less stable than 3 is in line with the cited literature (ref. 44). However, the authors discuss that 2 is 20.1 kJ/mol less stable than 3 (and is also less stable than 4), contrarily to experimental findings. I believe the relative instability of 2 can be over-estimated in this case. While 3 and 4 contain a single DMSO ligand, 2 contains two DMSO ligands. As a result, the presented energies are obtained by comparing G(2) to G(3 or 4) + G(free DMSO molecule). This approach tends to over-estimate the entropy of the free DMSO molecule. I would therefore strongly suggest the authors to compute the structures of 3 and 4 with a second explicit DMSO molecule interacting in the second coordination sphere of the complex. This adaptation of the computational model would allow a direct comparison between G(2) and G(3 with a second explicit DMSO) and can decrease the initially obtained 20.1kJ/mol difference.

Answer:

We completely agree that entropy corrections tend to introduce larger errors when treating cases with separating sub-systems. However, one also has to note that for a calculation with DMSO in the second coordination sphere, the interaction energy of DMSO with the complex will skew the corresponding result. While there is no perfect solution to this dilemma, we have carried out calculations with DMSO in the second coordination sphere to assess these effects. By comparing energies rather than free energies we can analyze the effects of entropy and interaction energies separately and have added the results and the corresponding discussion to the ESI. We would like to emphasize that a common scheme to compensate for the overestimation is the calculation of $\Delta G_{50\%}$ values in which the entropy correction is scaled by 0.5. But whether the relative free energies are computed with DMSO or via $\Delta G_{50\%}$ - the referee is absolutely correct with the remark that this effect accounts for the overstabilization of **2**. The best agreement with experiment is indeed found for the $\Delta G_{50\%}$ values which now confirm **2** to be most stable, **3** to be accessible within DFT error bars and **4** to be unlikely and we now describe this accordingly and report and assess the $\Delta G_{50\%}$ values in the manuscript.

A new section was added in ESI: for example subsection of Section 18 “Additional influences on the relative stabilities of 2, 3, and 4”

5. The tables in this section of the ESI are not labelled.

Answer: The tables in this section of the ESI have now been clearly labelled as Tables S18–S21 to improve clarity and ease of reference.

6. Chemical drawings should be included in the ESI figures of this section. Especially due to the bulkiness of the IMes ligand, 3D structures alone are not enough to clearly show the metal-ligand bonds, namely in figures S26, which includes Na+.

Answer: The original figure S26 was removed as not necessary (Fig 4 was updated). The structures of complexes **1-4** are used uniformly and when states with additions, for example, Fig S27 **1** and **1**+Na^+ structures. We think that this should be now clear, but the addition of Lewis structures to each 3D structure would not bring much, as they are all almost identical.

7. The XYZ coordinates of the computed structures are available in the domain presented in the ESI (https://doi.org/10.5281/zenodo.16746641). However, this webpage references an outdated title of the current manuscript and JACS as the source journal. I would like to remind the authors to update the details of this page.

Answer: The Zenodo repository has now been fully updated. The previous incomplete upload (DOI: 10.5281/zenodo.16746641) has been replaced with a complete version (DOI: 10.5281/zenodo.18449765), which includes the corrected manuscript title and journal reference, the new XYZ coordinates, and accompanying Readme files for guidance.

8. A functional benchmark is included in the ESI. However, since all other calculations were performed with def2-TZVP basis set, I don't understand why the authors chose to decrease the level of theory to a double-zeta basis set for this study. Also, performing single-point energy calculations with a different functional than the one used for the optimization is often not recommended. This is because the minimum energy structure can be significantly different from functional to functional, especially when considering: a) functionals that belong to different classes (in this benchmark: GGA, hybrid GGA and meta-GGA), b) hybrid functionals that contain different contributions of HF% in the exchange or present range-correction; c) the studied systems contain transition metal complexes. Therefore, I would recommend the authors to either re-compute the benchmark by optimizing structures 3 and 4 with the tested functionals (instead of computing a single point on a structure optimized with B3LYP-D4/def2-SVP) or remove this section entirely. I also noticed that the XZY coordinates of the structures of 3 and 4 used for this benchmark are not included in the available DFT data.

Answer:

We thank the referee for this comment – we have double checked all results and found that this is actually a typo, all geometries that had been used were obtained at the B3LYP-D4/def2-TZVP, CPCM, level of theory, and we corrected this in the revised version. However, the referee is certainly correct that the choice of a functional for the geometry optimization should also be assessed. We have added further results, in which we have re-optimized each structure at the given level of theory (PBE, PBE0, TPSS, ωB97x) and discuss the results in the SI (new subsection "Further DFT functional benchmarks").

9. Finally, the introduction of Na+ in the model must be further explored with computations. It was observed by the authors in the section "Formation energy of complex 2 from complex 1" of the ESI that computing the Gibbs free energy difference between 2 and 1 is an issue due to the stabilization of Cl-, which needs to dissociate from 1. In the table below Figure S27, it is even possible to observe large discrepancies of computed relative energies, depending on the computational model and, in particular, how the Cl- is stabilized. For this reason, I find surprising that the authors did not apply the same approach to the inclusion of Na+ in the computational model.

Answer:

We fully agree that the influence of Na^+ warrants deeper investigation, and we appreciate the reviewer raising this point. However, a thorough computational treatment of this effect — including proper benchmarking against experimental data from systems with different counter-cations (M^+) — goes beyond the scope of the present manuscript and is currently the subject of an ongoing study in our group.
In the revised manuscript, rather than presenting an incomplete computational picture, we have taken a more measured approach: we explicitly discuss which factors (including Na^+, Cl^-, DMSO coordination, and entropy corrections) may influence the

relative stabilities of the complexes, and we caution the reader that several of the computed relative energies carry significant uncertainty. We believe this framing is valuable in its own right, as it demonstrates that previous DFT analyses of related systems — which omitted these contributions — cannot be considered fully reliable, since any of these effects can individually alter, or even invert, the computed energetic ordering of the complexes.
We are confident that this more cautious and transparent presentation is appropriate for the current stage of the work, and that the full computational study, supported by additional experimental benchmarks, will be reported in a dedicated follow-up publication.

The results presented in the main text show that A is 7.2 kJ/mol more stable than B and are computed as shown in Scheme 1 (provided as attachment).

While examining the computed 3D structures, it is possible to observe that the form of chelation of sodium is different from A to B. As a result, the established O–Na bonds present a length 2.20 and 2.30 Å in A, while in B they are longer (both O–Na bonds in B are 2.31 Å) and therefore correspond to weaker interactions, leading to a weaker stabilization of the sodium ion. Given that this energy comparison is a key argument for the proposed characterization of complex 2 within this work, it is imperative to show strong evidence that the relative stability of A is obtained from a significant comparison to B and not due to an artifact of a computational model that is unable to promote the stabilization of the introduced cation.
Therefore, the computations including the sodium cation must be expanded to consider further stabilization of this species. If the trend in relative energies remains (2 being more stable than 3), the computational results would indeed be strong evidence for both the presented assignment of 2 as a key species in the SABRE reactivity and the role of sodium in the system.
I would like to provide the authors with two suggestions on how to approach the stabilization of the sodium ion. As I mentioned in point 4, B has a fewer DMSO molecule than A in the model, which could easily coordinate to the sodium ion. As such, one possible approach would be to consider the interaction of the dissociated DMSO with sodium, as depicted in Scheme 2 (provided as attachment). Alternatively, the solvent (MeOH) can be included explicitly in the computational model to account for the interactions with Na+, as shown in Scheme 3 (provided as attachment).

Answer: We perfectly agree with the doubts of the referee - there are many influences that will change computed interaction energies. However, for the discussion of the influence of Na^+ we would like the role of theory in this work to be understood as a tool for identifying possible influences, rather than yielding accurate numbers, and we now mention this in the revised manuscript.

Note that – as discussed for DMSO above - an explicit inclusion of a loosely bound solvent molecule would only represent one configuration of a possible solvent interaction. Even if a conformational search would be carried out, maybe a dozen low-energy configurations might be identified. However, in liquid phase, the system will

sample many orders of magnitude more configurations. The only appropriate treatment (as also recommended in the literature) would be large-scale, long-duration ab-initio molecular dynamics that include ions and counter ions. While we are currently working on similar approaches (machine learning potential MD based on foundation models) for further studies, this is not within the scope of this work.

In general, the focus of the paper is rather the experimental finding, not the computational protocol – we basically just propose a possible interpretation of the experimental values using simple models for the computation of the observed phenomena.

However, as the referee correctly points out, understanding and assessing the error bars and approximations of the computational model is essential. Hence, we have carried out the proposed calculations with Na^+ included adding DMSO in the second coordination sphere (see above) and adding one explicit methanol molecule representing an explicit interaction with a solvent molecule. While adding DMSO to system **3** and **4** does not change the stability with respect to **2** significantly, the relative stability of **3** and **4** is altered. As expected, one additional methanol changes the relative energies quite a bit, while **3** and **4** are still close in energy, they are more stable in this particular interaction. All of these results have been included in the SI, so that the inclined reader can assess the expected limited accuracy of the values given.

Comments of the reviewers and corresponding answers

Reviewer #1 (Remarks to the Author)

I thank the authors for taking my comments into account. I believe the manuscript is improved and can be published as is

Author reply:

Thank you for your constructive feedback throughout the review process. We sincerely appreciate the time and effort you invested in evaluating our manuscript and for recognizing the improvements made. Your comments have been valuable in helping us strengthen the quality of the work, and we are grateful for your support and recommendation for publication.

Reviewer #2 (Remarks to the Author)

The further revised manuscript is significantly improved, and the authors have addressed the changes I suggested and points raised. The critiques of Reviewer #4 on the computational data raise further questions about its reliability, and in my opinion the role of the Na+ cation is best addressed with experimental evidence, as alluded to in the original round of review. With the various caveats now noted in the computational study section, it is more suitable for publication in its current form.

Author reply:
Thank you for your thoughtful evaluation and for recognizing the significant improvements made in the revised manuscript. We sincerely appreciate your constructive comments throughout the review process, which have helped us strengthen the quality and clarity of our work.

1) One minor comment concerning the revised text for Figure 3: "The spectra were hyperpolarized using the SEPP-SPINEPT-SABRE sequence." Perhaps that can be rephrased, unless it really was the spectra and not the samples that were hyperpolarized

Answer: We thank the reviewer for the suggestion. We have revised the sentence to improve clarity.

Original text: The spectra were hyperpolarized using the SEPP-SPINEPT-SABRE sequence.

Modified text: The spectra were acquired using the SEPP-SPINEPT-SABRE sequence.

<u>Reviewer #4 -</u> Remarks to Author

The authors addressed my main concerns regarding the viability of the computational model by performing additional calculations and I am pleased to see that the new results reveal a much clearer agreement with the experiment. The presented computational data no longer requires an explicit interaction with Na+ to achieve a match between the relative Gibbs free energies of intermediate species and the experimental observations.

Author reply:

Thank you for your careful reassessment of our revised manuscript. We sincerely appreciate your constructive feedback throughout the review process, which has been invaluable in strengthening this work. We are pleased that the additional calculations addressed your main concerns regarding the viability of the computational model and that the new results show clearer agreement with the experimental observations. We also appreciate your recognition that the updated computational data no longer requires an explicit interaction with Na^{+} to reproduce the relative Gibbs free energies of the intermediate species consistent with experiment. Your insightful comments have greatly contributed to improving the quality of the manuscript.

I believe the manuscript can be accepted after the authors address the following minor details:

1) In Figure 4, the size of the iridium nucleus is inconsistent in B (very small in comparison to A and C).

Answer: We thank the reviewer for the suggestion.

Original figure:

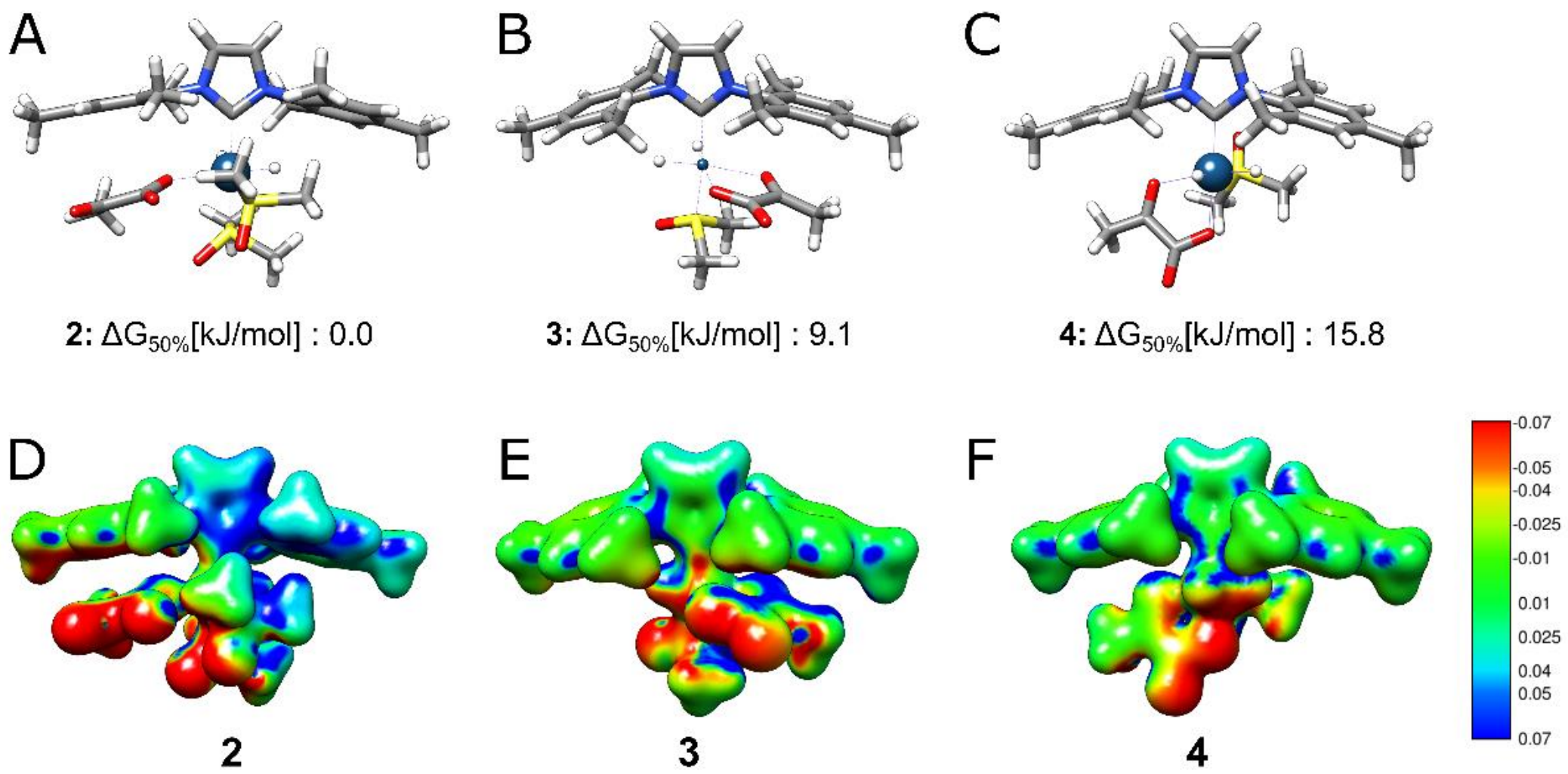

Modified text:

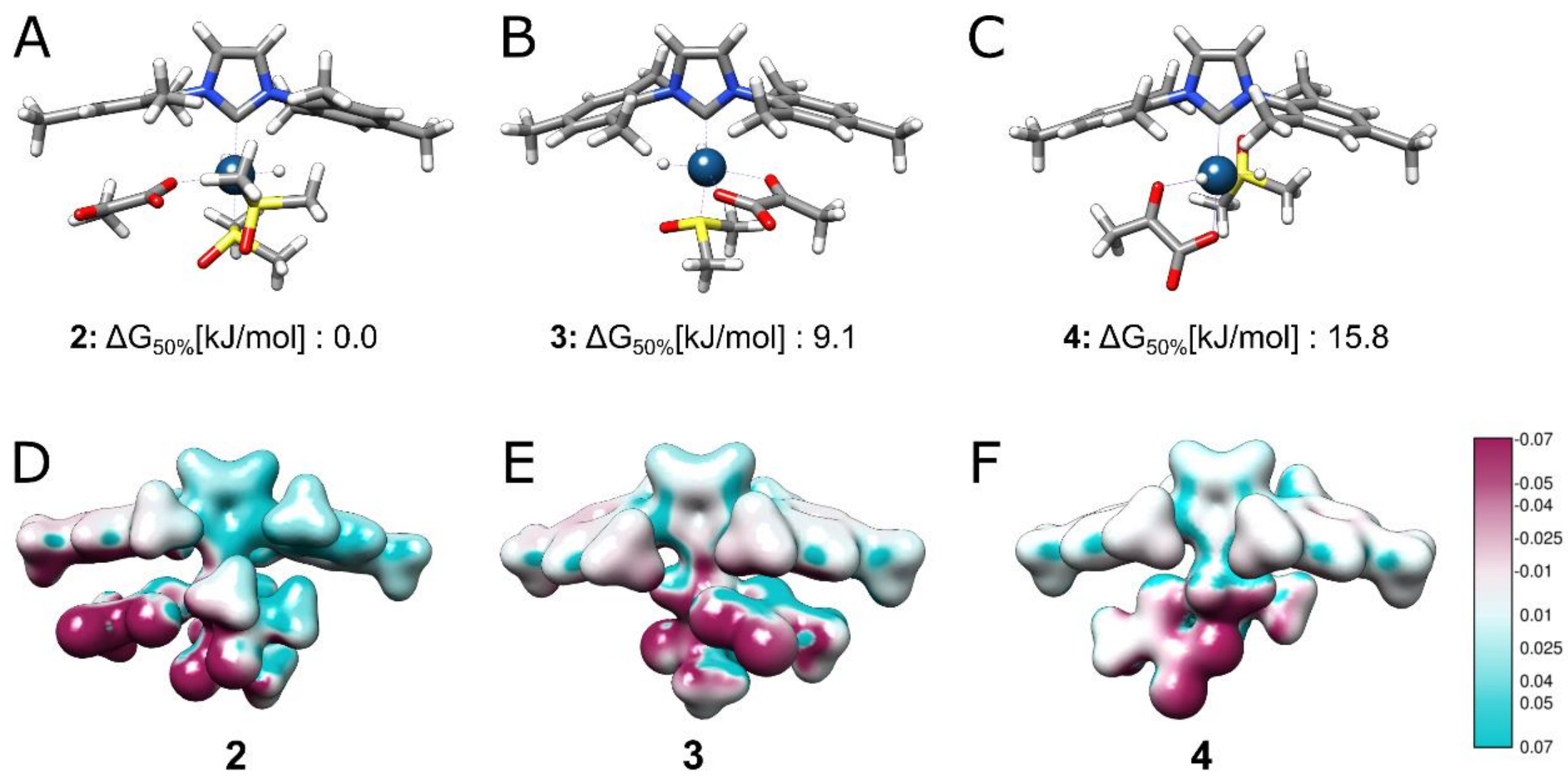

2) In the main text, I would suggest rephrasing “Within the error bars of DFT” to “within the accuracy of the level of theory” or “computational method”, since error bars were not explicitly calculated for this system.

Answer: We thank the reviewer for the suggestion.

Original text: Within the error bars of DFT, this is consistent with the experimentally evaluated thermodynamic parameters presented above.

Modified text: Within the accuracy of the level of theory, this is consistent with the experimentally evaluated thermodynamic parameters presented above.

3) In the Figure S26 of the ESI I would insist on the authors to either include the Lewis structures of each computed complex or to explicitly display in the 3D figure the bonds to Na so the reader can easily see the different considered interactions.

Answer: Thank you for this helpful suggestion. We appreciate your attention to clarity in the presentation of the computational results. We agree that improving the readability of Figure S26 is important for the reader to better understand the different interaction motifs considered in the study. In the revised Supporting Information, we will accordingly update Figure S26 by either including the corresponding Lewis structures of each computed complex or explicitly highlighting the Na^{+} coordination interactions within the 3D representations, so that the differences between the considered binding modes are immediately clear.

Modified text:

We add Figure S26(2) containing only Lewis structures.

A

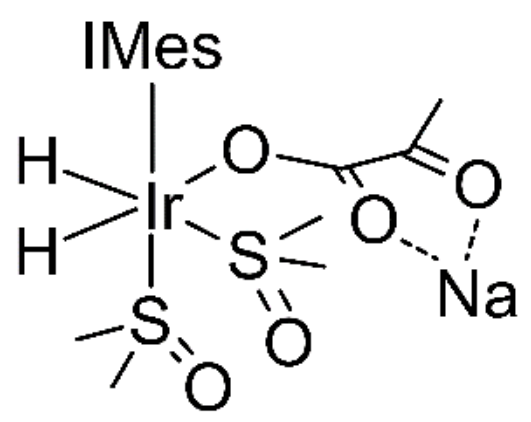

2: ΔE[kJ/mol] : 0.0
ΔG[kJ/mol] : 0.0
$\Delta G_{50\%}$[kJ/mol] : 0.0

B

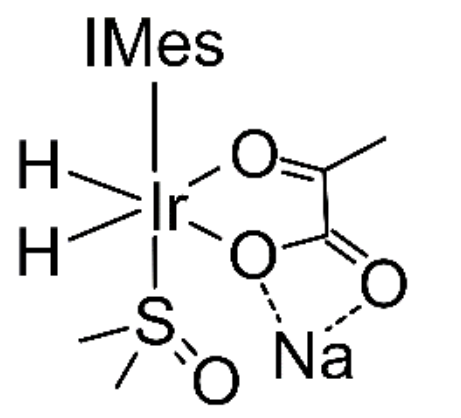

3: ΔE[kJ/mol] : 79.7
ΔG[kJ/mol] : 7.2
$\Delta G_{50\%}$[kJ/mol] : 39.2

C

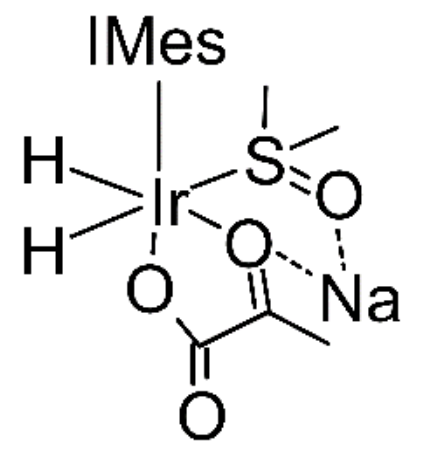

4: ΔE[kJ/mol] : 66.7
ΔG[kJ/mol] : 2.0
$\Delta G_{50\%}$[kJ/mol] : 30.9

D

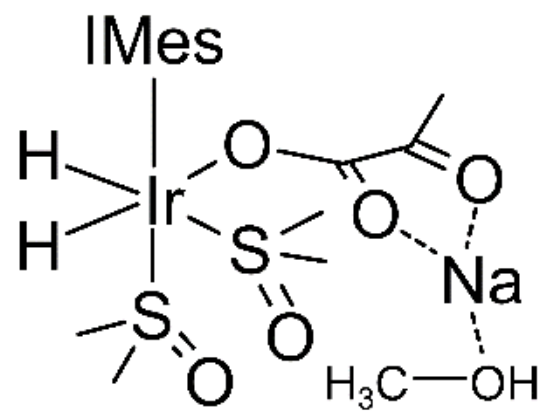

2: ΔE[kJ/mol] : 0.0
ΔG[kJ/mol] : 0.0
$\Delta G_{50\%}$[kJ/mol] : 0.0

E

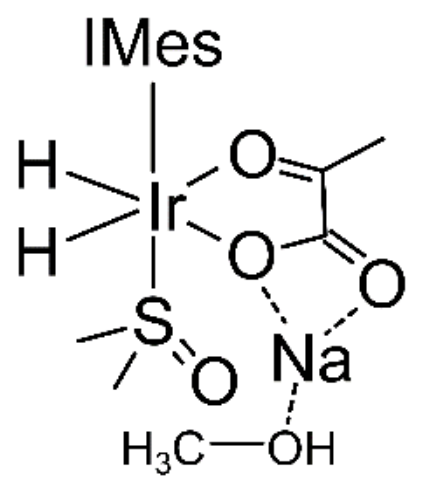

3: ΔE[kJ/mol] : 68.3
ΔG[kJ/mol] : 2.5
$\Delta G_{50\%}$[kJ/mol] : 31.2

F

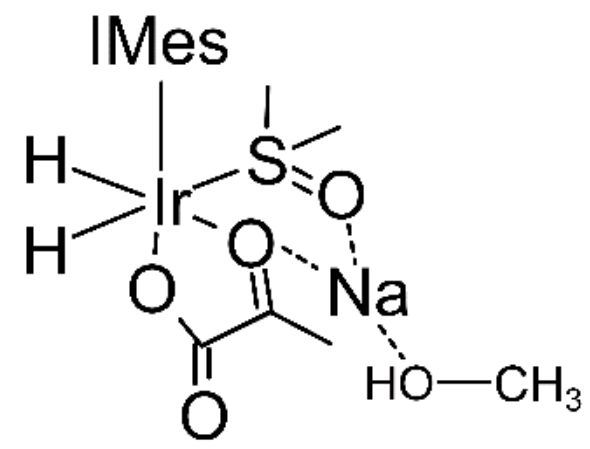

4: ΔE[kJ/mol] : 64.9
ΔG[kJ/mol] : 3.1
$\Delta G_{50\%}$[kJ/mol] : 30.9

G

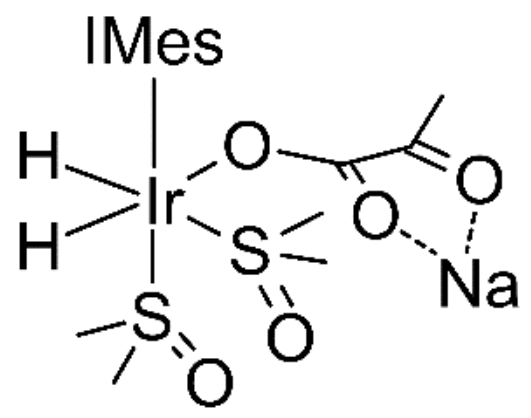

2: ΔE[kJ/mol] : 0.0
ΔG[kJ/mol] : 0.0
$\Delta G_{50\%}$[kJ/mol] : 0.0

H

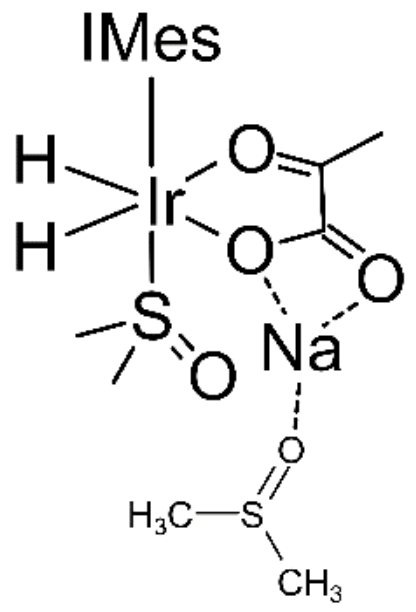

3: ΔE[kJ/mol] : - 4.8
ΔG[kJ/mol] : - 19.6
$\Delta G_{50\%}$[kJ/mol] : - 14.7

I

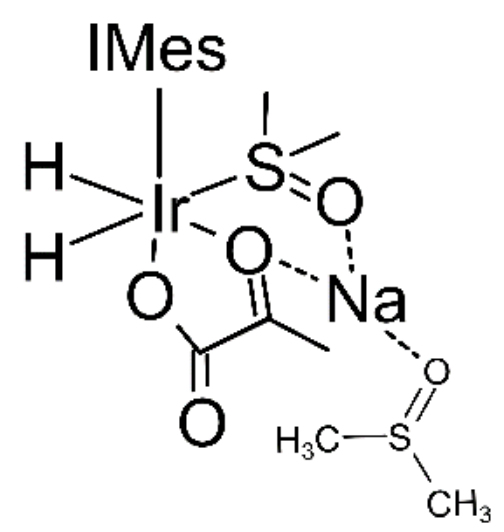

4: ΔE[kJ/mol] : - 11.4
ΔG[kJ/mol] : - 14.6
$\Delta G_{50\%}$[kJ/mol] : - 14.2

4) Table S20 of the ESI must contain a typo: the entries of E3 and E4, reoptimized at the DFT-D4/def2-TZVP CPCM level of theory in the column of B3LYP (last column, last 3 rows) have to be exactly the same as the ones in the same column, first 3 rows: -1924.707791733 for E3 and -1924.705312465 for E4.

Answer: Thank you for carefully identifying this issue in Table S20 of the ESI. We appreciate your close examination of the data. You are correct that these entries contained a typographical error. The values for E3 and E4 in the B3LYP column (last column, last rows) have now been corrected to match the reoptimized DFT-D4/def2-TZVP CPCM results. The revised table has been updated accordingly in the Supporting Information.

Scheme 1

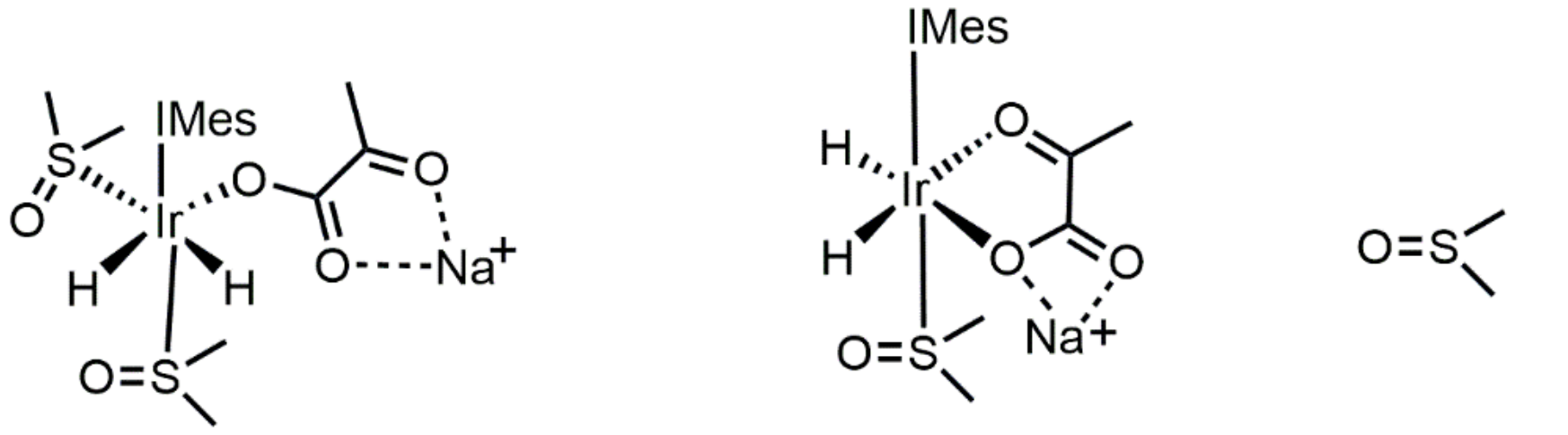

A in S24 B in S24 Free DMSO

$\Delta G(A \rightarrow B) = (\Delta G(B) + \Delta G(DMSO)) - \Delta G(A)$

Scheme 2

Example 1) Direct interaction with dissociated DMSO

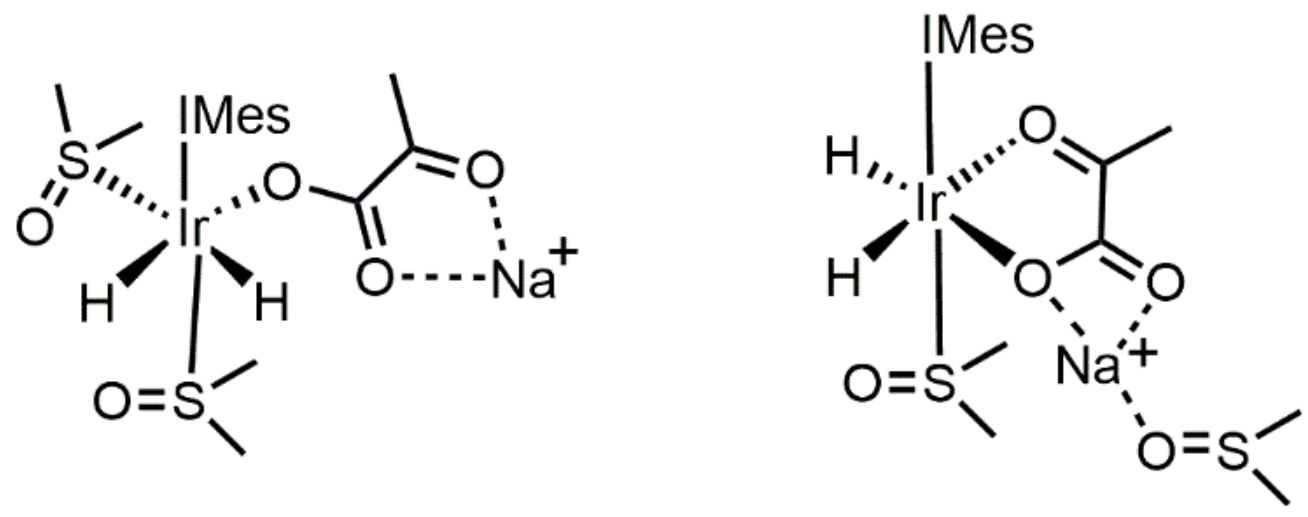

A in S24 B With explicit DMSO

$\Delta G(A \rightarrow B) = (\Delta G(B \text{ with DMSO}) - \Delta G(A)$

Scheme 3

Example 2) Solvation by MeOH

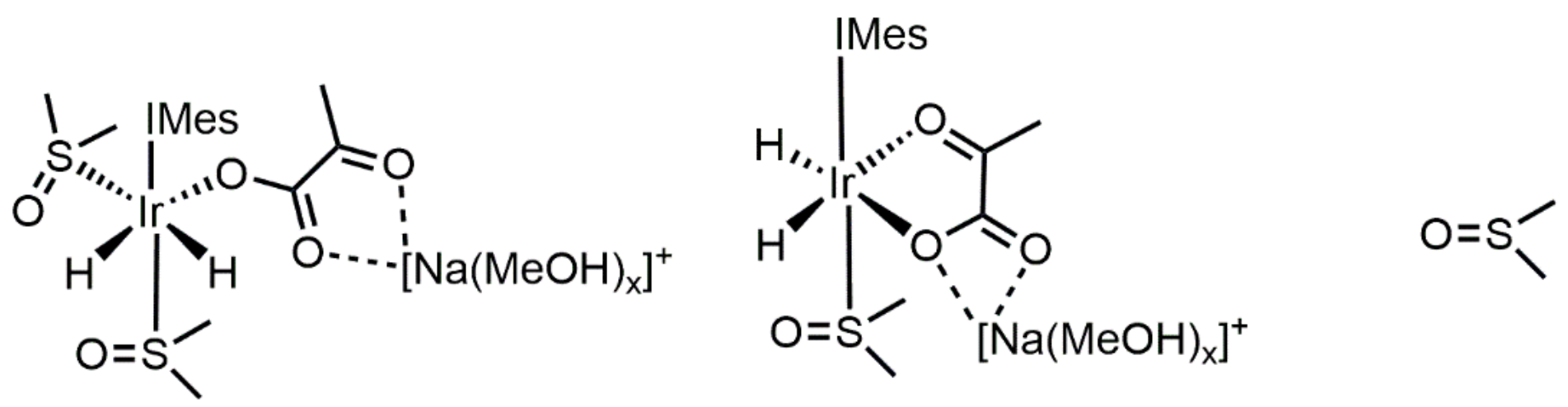

A with solvated Na B with solvated Na Free DMSO

$\Delta G(A \rightarrow B) = (\Delta G(B) + \Delta G(DMSO)) - \Delta G(A)$
